# Periodic Hirshfeld Atom Refinement

**DOI:** 10.1021/acs.jpclett.5c03918

**Published:** 2026-02-27

**Authors:** Kanghyun Chu, Dylan Jayatilaka, Lorraine A. Malaspina, Alessandro Genoni, Georgia Cametti, Stefan Mebs, Dieter Lentz, Hans-Beat Bürgi, Sergey V. Churakov, Simon Grabowsky

**Affiliations:** † Department of Chemistry, Biochemistry and Pharmaceutical Sciences, 27210 University of Bern, Freiestrasse 3, 3012 Bern, Switzerland; ‡ Research Group for Structural Biochemistry and Mechanisms, Max-Planck Institute for Multidisciplinary Sciences, Am Fassberg 11, 37077 Göttingen, Germany; ¶ School of Molecular Sciences, University of Western Australia, 35 Stirling Highway, Crawley, Western Australia 6009, Australia; § Department of Chemistry, Materials and Chemical Engineering “Giulio Natta”, 18981 Politecnico di Milano, Via Bassini, 20133 Milano, Italy; ∥ Institute of Geological Sciences, 27210 University of Bern, Baltzerstrasse 1+3, 3012 Bern, Switzerland; ⊥ Department of Physics, 9166 Free University of Berlin, Arnimallee 14, 14195 Berlin, Germany; # Institute of Chemistry and Biochemistry, 9166 Free University of Berlin, Fabeckstr. 34/36, 14195 Berlin, Germany; @ PSI Center for Nuclear Engineering and Sciences, Paul Scherrer Institute, Forschungsstrasse 111, 5232 Villigen PSI, Switzerland

## Abstract

Hirshfeld Atom Refinement (HAR) is a quantum crystallographic method for analyzing single-crystal X-ray diffraction data, providing accurate and precise structural parameters. Despite its success in predicting hydrogen-atom parameters, the application of HAR is fundamentally limited to molecular crystals. Inspired by two recently developed HAR versions that employ periodic-boundary conditions, here we introduce a new variant of periodic HAR (pHAR) that is applicable to any periodic-network structure while remaining compatible with conventional HAR by using atom-centered Gaussian orbitals with a Bloch wave formalism. pHAR was tested against high-quality single-crystal diffraction data for boranes and borates comprising N–H and B–H bonds in different chemical environments. The results demonstrate a close agreement of X–H bond lengths with reference data from neutron-diffraction experiments with improved precision. Using pHAR, this study has nearly doubled the previously available body of reliable experimental structural data on B–H bonds.

## Graphical abstract

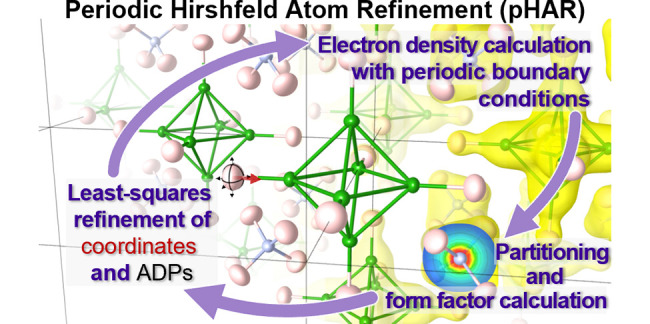

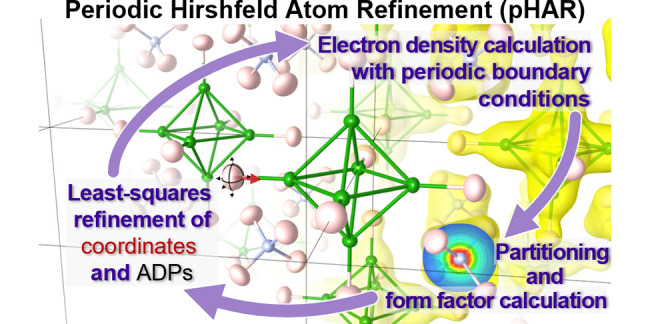

Single-crystal X-ray diffraction is the leading technique for atomically resolved structure elucidation. The technique places models of atomic electron densities in the crystal unit cell and optimizes their positions and mean-square displacements (atomic displacement parameters, ADPs) with a least-squares procedure to best fit the experimental diffraction pattern. The simplest and most widely adopted model is the Independent Atom Model (IAM).^1^ It represents the crystal electron density with spherically symmetric, noninteracting atomic densities calculated quantum mechanically; their Fourier transforms are the so-called spherical atomic form factors.^2^ This approach neglects bonding-induced anisotropy, leading to a bias in atomic positions and ADPs. This is especially important for hydrogen atoms with their highly polarized single valence electrons.^3,4^ X-H bond lengths in light-atom structures obtained with an IAM are typically 0.1 Å shorter than corresponding values obtained by neutron diffraction.^5^

Two modern techniques of quantum crystallography overcome these limitations: the multipole model (MM) and Hirshfeld Atom Refinement (HAR).^6–8^ The MM complements spherical atomic densities with weighted spherical harmonic functions representing electric dipole, quadrupole, octupole, and hexadecapole moments (deformation functions).^9–11^ The weight factors (multipole populations) are either refined against experimental X-ray diffraction data (together with atomic positions and ADPs)^12–14^ or taken from databases derived from experiments^15^ or from quantum chemical calculations.^16,17^ The additional parameters can account for chemical bonding and crystal-field effects; they improve the hydrogen atom parameters significantly, all without much loss in computational efficiency.^18,19^

HAR models are based on tailor-made, quantum-chemically calculated electron densities of unit-cell building blocks, i.e. molecules or clusters of molecules.^20–23^ The electron densities of the blocks are partitioned into general nonspherical Hirshfeld atomic densities of all symmetry-unique atoms. Their Fourier transforms are used in conventional least-squares refinement of the atomic positions and ADPs. The electron densities are recalculated with the refined atomic positions and the process is iteratively cycled until the atomic parameters and the quantum chemical energy converge. HAR is far more computationally expensive than MM but can yield more accurate structure models because a high-quality and rigorous quantum-chemical model of the underlying electron density is used.^24–26^ For light atom molecular structures, hydrogen-atom positions can attain precision and accuracy comparable to those from neutron diffraction.^27,28^

The various models in the HAR family differ in several aspects (see the references for details).

I Choice of the atomic assembly for the quantum-chemical calculation: isolated molecule, or a molecule embedded in a dielectric medium;^20,22,29^ molecule surrounded by a cluster of molecules represented by self-consistent point charges and dipoles simulating an approximate crystal field;^24,30^ explicit cluster of molecules around a central unit;^22,31^ content of complete unit cell with crystal periodicity.^32,33^

II Representation of the electron density: plane-wave basis;^32^ local atomic basis functions;^20,21^ pseudopotential descriptions of core electrons;^33,34^ projections onto multipole functions.^35,36^

III Hamiltonian: Hartree–Fock;^20,37^ Post Hartree–Fock;^38^ Density Functional Theory;^21,30,39,40^ Semiempirical.^41^
IV Partitioning scheme: fragmentation Hirshfeld atom approaches;^42–44^ atomic partitioning alternative to Hirshfeld atoms.^45,46^

Here, we focus on the most general choice of atomic assembly, namely a periodic electron-density distribution. This allows treating infinite network compounds without arbitrarily cutting interatomic (covalent or long-range Coulomb) interactions at the boundaries of the unit cell. Such approaches have been tested by Wall,^32^ Ruth et al. (XHARPy)^33^ and Patzer and Lehmann (ReCrystal).^35^

For our version of periodic HAR (pHAR), we avoid the use of a plane-wave basis (Wall^32^). While it is well suited to describe valence electron densities, a plane-wave basis becomes impractical for modeling highly localized core-level electrons which represent an important part of the atomic scattering density. A comparison between plane-wave- and Gaussian-basis-set-derived electron densities can be found in ref 47. Pseudopotential descriptions were excluded because they cannot account for core polarization (Ruth^33^).^48–50^ For heavy elements, the use of effective core potentials can be useful in HAR, but it requires correction functions.^34^ We also decided not to consider an approach in which the quantum chemical electron density is first projected onto multipole functions followed by a multipole refinement (Patzer and Lehmann^35^). In our pHAR method, we treat all electrons—both core and valence—on an equal footing. Electronic wave functions are expressed in terms of Bloch functions of atom-centered Gaussian orbitals. This choice allows meaningful comparisons with results from traditional quantum chemistry and conventional HAR on finite atomic assemblies provided Hamiltonians and basis sets are the same. It also provides density matrices for complementary chemical bonding analysis.^51^

The pHAR procedure is visualized in more detail in Figure 1. Starting from a tentative crystal structure (e.g., IAM), Crystal23 ^52^ calculates its electronic wavefunction under periodic-boundary conditions imposing the correct crystallographic symmetry. The software Tonto^53^ reads the Crystal23 density matrix, calculates the electron density ρ­(**r**) and partitions it into nonspherical atomic densities ρ_*i*_(**r**) which are Fourier transformed into nonspherical atomic form factors *f*_*i*_(**h**). If the initial and refined parameters do not agree within the predefined tolerances, the refined structure becomes the new tentative structure and the procedure is repeated until convergence. Crystal23 and Tonto are interfaced via lamaGOET.^54^ More details of the implementation are given in the section Experimental and Computational Methods and in the Supporting Information.

**Figure 1:**
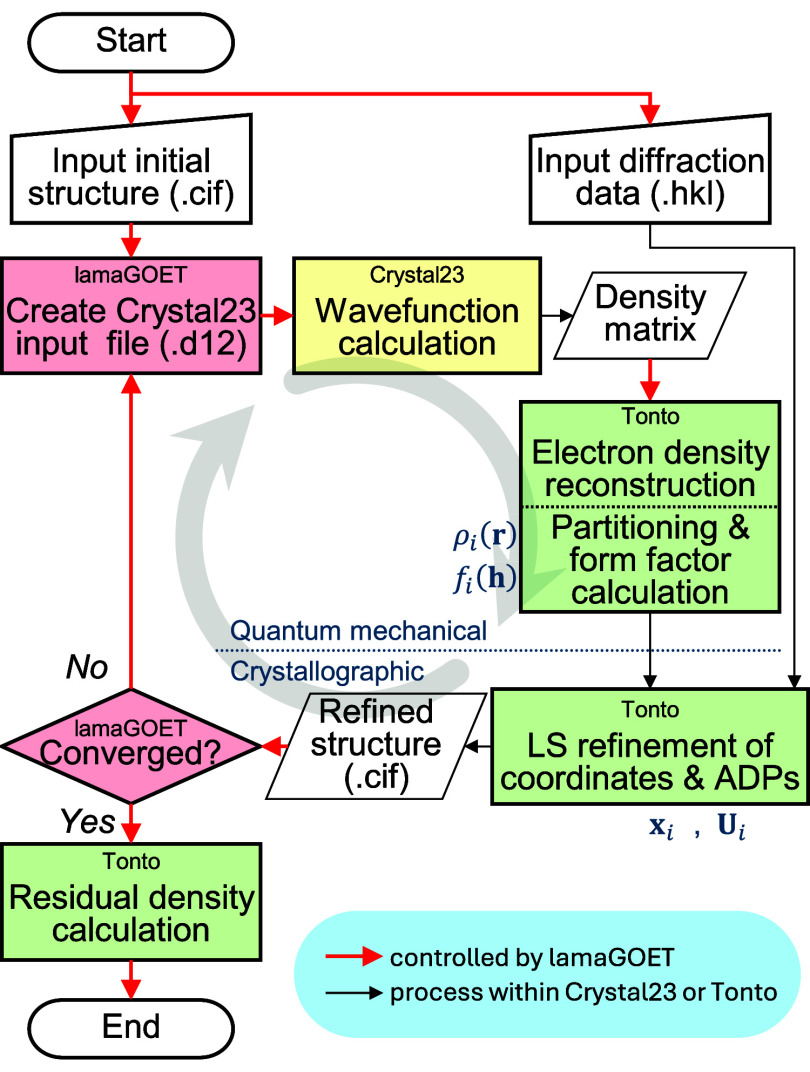
Schematic diagram of pHAR. Above the dotted line: quantum mechanical calculation of nonspherical Hirshfeld atom electron densities ρ_*i*_(**r**) and their Fourier transforms *f*_*i*_(**h**) (nonspherical atomic form factors). Below: crystallographic least-squares refinement for updating atomic positions **x**_*i*_ and ADPs **U**_*i*_. The red arrows indicate the automated control by lamaGOET.

The performance of the newly developed pHAR was tested with high-quality single-crystal data for molecules with X-H bonds (X=B, C, N, O). Here, we concentrate primarily on B–H and N–H bonds which have been less studied than C–H or O–H bonds. There are only three published HAR studies of boranes/borates: diborane, B_2_H_6_, in ref 28; bis­(ammonium) closo-hexaborate(6), (NH_4_)_2_B_6_H_6_, in ref 22; and m-terphenylhydridoborates in ref 55. There are no studies of bigger cage structures and no systematic comparisons of the refined structures obtained with HAR or with neutron-diffraction data. We have included six neutral borane and ammonium borate compounds for which high-quality data sets are available and which were previously studied with MMs.^56–58^ We also included bis­(ammonium) closo-hexaborate(6), (NH_4_)_2_B_6_H_6_, from a previous HAR study, which consists of highly symmetric, small molecular ions.^22^ Ammonia and diborane are included as small neutral parent compounds for the N–H and B–H bonds, respectively. The single crystal X-ray diffraction data of ammonia (160 K) and of diborane (94 K) are taken from the literature.^59,60^ Figure 2 shows all the B–H and N–H compounds included in this study after pHAR treatment. It covers a wide variety of chemical environments. The compounds contain B–H bonds in both terminal (t) and bridging (b) positions, in anionic and neutral species, as well as N–H bonds, with or without hydrogen bonding, in cationic and neutral species.

**Figure 2:**
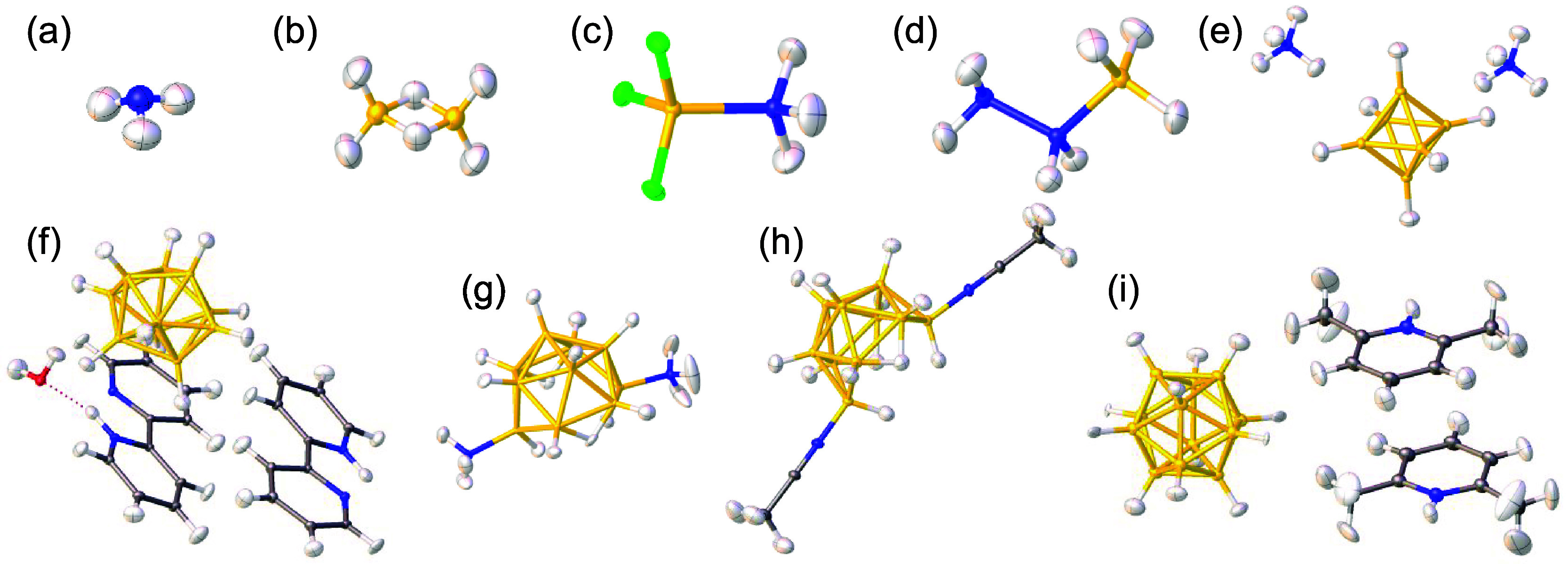
Structures of B–H and N–H compounds refined with pHAR: (a) ammonia,^59^ NH_3_; (b) diborane(6),^60^ B_2_H_6_; (c) ammonia trifluoroborane,^56^ NH_3_BF_3_; (d) hydrazine borane,^56^ N_2_H_4_BH_3_; (e) bis­(ammonium) closo-hexaborate(6),^22^ (NH_4_)_2_B_6_H_6_; (f) bis­(2,2′-bipyridinium) closo-decaborate(10) hydrate,^57,58^ (C_10_H_9_N_2_)_2_B_10_H_10_·H_2_O; (g) bis­(ammonia) arachno-decaborane(12),^57,58^ (NH_3_)_2_B_10_H_12_; (h) bis­(acetonitrile) arachno-decaborane(12),^57,58^ (CH_3_CN)_2_B_10_H_12_; (i) bis­(2,6-lutidinium) closo-dodecaborate(12),^57,58^ (C_7_H_10_N)_2_B_12_H_12_. The ADP ellipsoids are scaled to represent a 50% probability, representing atomic vibrations. Each element is colored as follows: H, white; B, yellow; C, gray; N, blue; O, red; F, green.

The refined bond distances are compared with values from a compilation of corresponding distances obtained by neutron diffraction. For B–H, distances are compared with the mean (and standard uncertainty) of the distribution of terminal and bridging B–H bond distances determined with neutron diffraction at various temperatures: d­(B–H_t_) = 1.185(18)­Å and d­(B–H_b_) = 1.338(12)­Å.^5^ The N–H distances chosen as references are bond-length populations coming from neutron-diffraction experiments: d­(N^+^–H) = 1.036(16) Å, d­(Z–N–H) = 1.015(16) Å, and d­(Z_2_–N–H) = 1.027(16) Å.^5^ Note that these uncertainties do not reflect the precision of individual refinements, but are sample standard deviations representing both measurement uncertainty and the variance of chemical environments across neutron diffraction measurements. It is also worth noting that the number of observations in ref 5 is much smaller for B–H bonds than for N–H bonds: 27 and 10 for B–H_t_ and B–H_b_, respectively, compared with 187, 233, and 74 for N^+^–H, Z–N–H, and Z_2_–N–H.

In a few cases, structural data are available from both X-ray and neutron diffraction, measured on the same molecule or its isotopologues at approximately the same temperature, thus allowing the most conclusive test on the performance of pHAR. Ammonia (NH_3_) at 160 K is compared with ND_3_ studied by powder neutron diffraction at 180 K (d­(N–D) = 0.989(5) Å).^61^ Note that, due to the isotope effect, d­(N–H) > d­(N–D) by ∼0.004 Å (as estimated from a gas-phase electron-diffraction experiment).^62^ The comparison for diborane is with values from a gas phase electron diffraction experiment interpreted with spherical atomic electrostatic potentials: d­(B–H_t_) = 1.196(8) Å, d­(B–H_b_) = 1.339(6) Å.^63^ Note, though, that X–H distances from single-crystal electron-diffraction experiments interpreted with spherical form factors tend to be too long.^64,65^

First, the performance of various models (including IAM) was investigated for ammonia^59^ varying the basis sets and the ways of simulating the crystal field (Table 1). The R values decrease as the level of basis set sophistication increases and the simulated crystal fields become more realistic. The maximum, minimum, and root-mean-square (rms) residual densities follow the same trend, with three minor exceptions. The corresponding three-dimensional maps for IAM, HAR, and pHAR confirm this finding (Figure 3): the residual density becomes progressively smaller and smoother, as apparent from the shrinking isosurfaces and the reduced undulation of the contour lines. The density at the bond sites is also reduced.

**Table 1:** Refinement Statistics (R factor), Maximum/Minimum/Root-Mean-Square (rms) Residual Densities (e Å^–3^), N–H Bond Lengths (Å), and H–N–H Angles (deg) for Ammonia^a^

Model	Basis set	R	Δρ_max_	Δρ_min_	Δρ_rms_	Length	Angle
IAM		0.0130	0.0474	–0.0585	0.0130	0.825(8)	108.5(5)
HAR	STO-3G	0.0234	0.0543	–0.1025	0.0214	0.952(13)	106.7(8)
HAR	6-311G(d,p)	0.0090	0.0292	–0.0296	0.0078	0.953(6)	108.5(4)
HAR	pob-TZVP-rev2	0.0085	0.0310	–0.0233	0.0079	0.933(6)	109.1(4)
HAR/CC	STO-3G	0.0233	0.0542	–0.1018	0.0212	0.956(13)	106.8(8)
HAR/CC	6-311G(d,p)	0.0083	0.0252	–0.0258	0.0074	0.965(5)	108.2(3)
HAR/CC	pob-TZVP-rev2	0.0078	0.0280	–0.0187	0.0072	0.945(5)	108.8(3)
pHAR	STO-3G	0.0225	0.0509	–0.1010	0.0207	0.957(13)	106.8(8)
pHAR	6-311G(d,p)	0.0074	0.0242	–0.0215	0.0067	0.968(4)	108.1(2)
pHAR	pob-TZVP-rev2	0.0069	0.0230	–0.0161	0.0065	0.947(4)	108.6(2)
HAR/CC (HF)^21^	6-311++G(2d,2p)	0.0092				0.987(5)	107.9(3)
HAR/CC (HF)^54^	def2-TZVP	0.0073				1.002(5)	108.0(3)
Neutron diffraction (ND_3_)^61^						0.989(5)	107.8(4)

a HAR denotes refinement with no crystal field, HAR/CC with cluster charges within a radius of 8 Å, and pHAR with periodic-boundary conditions. All calculations were performed using the B3LYP method.

**Figure 3:**
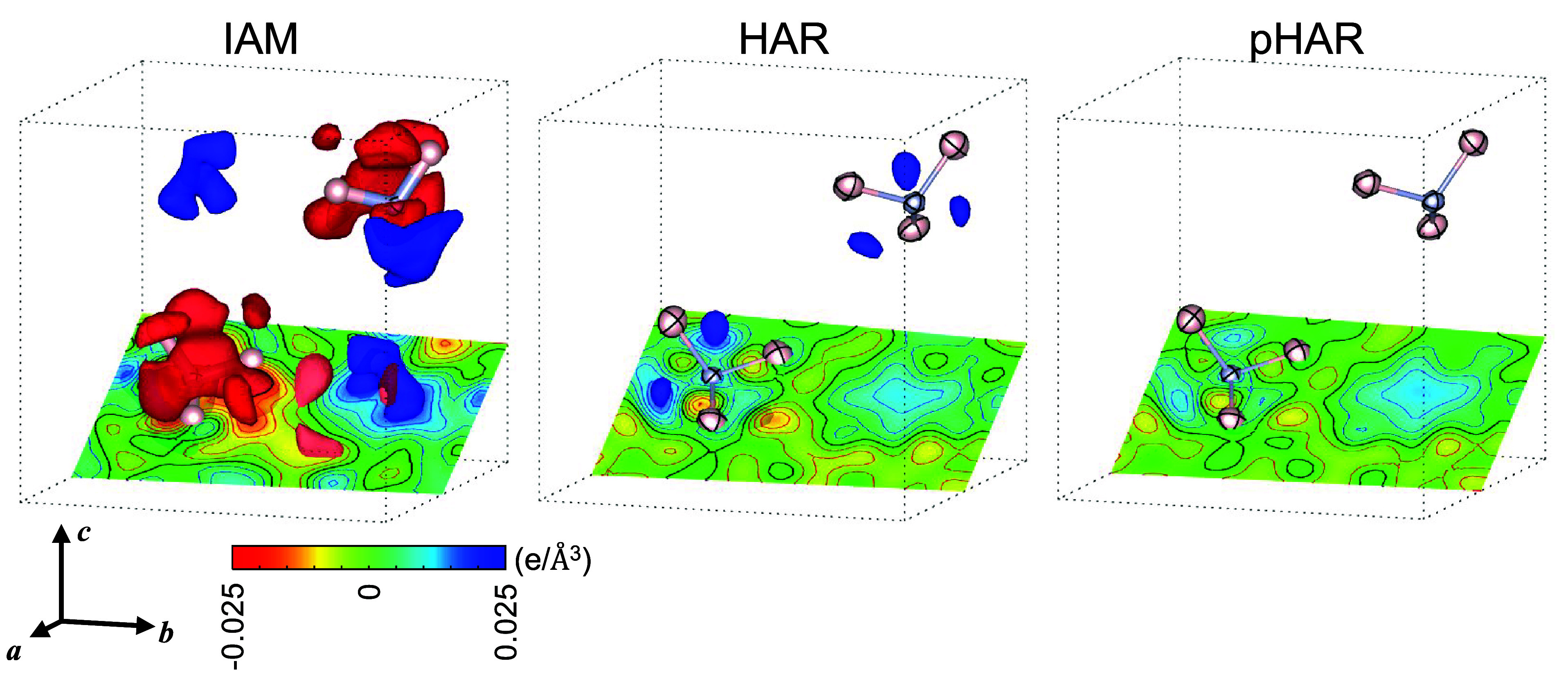
Residual electron-density maps of ammonia for IAM, HAR, and pHAR. The dotted lines indicate the cubic unit cell of *a* = 5.1305 Å. Two ammonia molecules in the rear half of the cell are shown (0 ≤ *x* ≤ 1/2). Cross-sectional contour maps on the planes defined by the H–N–H bonds are shown with 0.005 e/Å^3^ per step. The isosurface level is at ±0.025 e/Å^3^ (positive blue; negative red). The ADPs are shown at the 10% probability level. The HAR and pHAR results are obtained using the pob-TZVP-rev2 basis set and the B3LYP hybrid functional.

The refined bond lengths increase for every basis set as the crystal field model becomes more realistic, approaching the neutron ND_3_ reference value of 0.989(5)­Å. Likewise, agreement with the neutron bond angle improves progressively from HAR to HAR/CC to pHAR. However, there is no obvious correlation between R value and bond length. The best match in N–H distance (pHAR, 6-311G­(d,p), d = 0.968 Å, R = 0.0074) is not associated with the lowest R value (pHAR, pob-TZVP-rev2, d = 0.947 Å, R = 0.0069). For STO-3G, the electron-density prediction is extremely poor—even worse than IAM—yet the structure prediction is not nearly as bad. Earlier refinements with a basis set containing diffuse functions or the simpler Hartree–Fock Hamiltonian showed better geometrical agreement, although with slightly larger R values.^21,54^ Superior results with the simpler HF method compared to DFT methods were also found recently in a study that benchmarked methods and basis sets for HAR.^37^ The authors ascribed this finding to a cancellation of errors. The different behavior of R and d­(N–H) for ammonia in our study indicates that, even with the present sophistication of modeling electron density using pHAR, the hydrogen-atom position still depends on how accurately the quantum-mechanical model describes the electron density in the vicinity of H and of N. This suggests that the choice of the basis set and Hamiltonian influences the geometric refinement results, but not necessarily in a foreseeable way. For ammonia, their effect appears to be larger than the effect due to the different models of the crystal field.

This conclusion is further substantiated by a comparison of pHAR with the best XHARPy results using two test examples reported in the literature.^33^ It is found that pHAR showed lower performance for most but not all investigated parameters (see the Supporting Information, section 5). E.g., for the five O–H bonds in the molecule xylitol the average absolute difference between neutron and X-ray derived O–H distances from pHAR with the B3LYP/pob-TZVP-rev2 level of theory is 0.043(9)­Å, whereas it is 0.011(7)­Å from XHARPy with the revSCAN functional. The highly polar O–H bonds are most susceptible to the choice of method and basis set as we will discuss below. Considering that valence electrons in XHARPy are described by plane-wave basis functions—which are by definition completely delocalized—and in view of the data in Table 1, the choice of basis set should be carefully examined to achieve the best structural agreement with neutron data. Currently, the use of basis sets with diffuse functions is limited in Crystal23 and thus in pHAR, because, as it is well-known, they generally cause numerical instabilities in periodic calculations with atom-centered Gaussian orbitals.

The borane/borate compounds were refined with IAM, conventional HAR, and pHAR, using the B3LYP/pob-TZVP-rev2 level of theory for both HAR and pHAR (more details are given in the section Experimental and Computational Methods). Table 2 reports the R factors and residual electron densities for each compound. Consistent improvements are observed from IAM to HAR/pHAR across all the eight compounds. However, the differences between HAR and pHAR in terms of R values and residual densities are not significant. It suggests that the polarization due to the crystal field for these molecular crystals has no measurable impact on these metrics. There are some cases that exhibit significant residual electron density (Δρ_max_ > 0.5 e Å^–3^) even after HAR or pHAR refinements. Therefore, we have compared the residual densities between HAR, pHAR and the original multipole models^56,57^ in the Supporting Information, section 6. For some cases, the high residual densities are a chemical and not a model effect, for some cases HAR/pHAR show lower values, for some cases higher values than MM. Overall, a fair comparison between MM and HAR/pHAR is difficult because only merged data are available, the numbers of reflections used in MM and HAR/pHAR are not the same, and the multipole populations in MM are adjusted to the experimental data during the refinement, whereas the charge distributions in HAR/pHAR are fixed during the refinement.

**Table 2:** Refinement Statistics (R Factor) and Residual Electron Densities (e Å^–3^) for All Borane and Borate Compounds

**Name**, Formula		IAM	HAR	pHAR
**Diborane(6)**	R	0.0374	0.0318	0.0320
B_2_H_6_	Δρ_max_	0.1210	0.0866	0.0859
	Δρ_min_	–0.1788	–0.1758	–0.1777
	Δρ_rms_	0.0247	0.0192	0.0192
**Ammonia trifluoroborane**	R	0.0249	0.0202	0.0205
NH_3_BF_3_	Δρ_max_	0.6843	0.5671	0.5830
	Δρ_min_	–0.2192	–0.1188	–0.1214
	Δρ_rms_	0.0929	0.0683	0.0681
**Hydrazine borane**	R	0.0295	0.0220	0.0220
N_2_H_4_BH_3_	Δρ_max_	0.6623	0.5533	0.5442
	Δρ_min_	–0.1323	–0.1027	–0.1025
	Δρ_rms_	0.0611	0.0458	0.0455
**Bis(ammonium)**	R	0.0188	0.0180	0.0183
**closo-hexaborate(6)**	Δρ_max_	0.1724	0.1153	0.0962
(NH_4_)_2_B_6_H_6_	Δρ_min_	–0.2199	–0.2401	–0.2540
	Δρ_rms_	0.0312	0.0262	0.0269
**Bis(2,2**′**-bipyridinium)**	R	0.0365	0.0239	0.0239
**closo-decaborate(10) hydrate**	Δρ_max_	0.9586	0.6389	0.6294
(C_10_H_9_N_2_)_2_B_10_H_10_·H_2_O	Δρ_min_	–0.5758	–0.4115	–0.4178
	Δρ_rms_	0.1243	0.1155	0.1156
**Bis(ammonia)**	R	0.0297	0.0232	0.0230
**arachno-decaborane(12)**	Δρ_max_	0.3176	0.1963	0.2012
(NH_3_)_2_B_10_H_12_	Δρ_min_	–0.2653	–0.1724	–0.1791
	Δρ_rms_	0.0439	0.0345	0.0345
**Bis(acetonitrile)**	R	0.0358	0.0212	0.0217
**arachno-decaborane(12)**	Δρ_max_	0.9682	0.3502	0.3549
(CH_3_CN)_2_B_10_H_12_	Δρ_min_	–0.3328	–0.1360	–0.1311
	Δρ_rms_	0.0546	0.0298	0.0301
**Bis(2,6-lutidinium)**	R	0.0442	0.0321	0.0325
**closo-dodecaborate(12)**	Δρ_max_	0.8145	0.7873	0.8005
((CH_3_)_2_C_5_H_4_N)_2_B_12_H_12_	Δρ_min_	–0.2666	–0.3301	–0.3338
	Δρ_rms_	0.0619	0.0404	0.0410

As seen in the ammonia case, improvements in R-statistics do not necessarily ensure closer agreement of bond lengths with the reference data, and vice versa. Therefore, we examine trends in the N–H and B–H bond distances in more detail. Figure 4 shows the distribution of refined d­(N–H) and d­(B–H) for each kind of molecule in the unit cells. Only (a) ammonia and (e) bis­(ammonium) closo-hexaborate(6) exhibit differences in individual bond lengths between HAR and pHAR beyond a single standard uncertainty. On the other hand, aggregate analysis shows that the averages of the estimated standard deviations (esds) of d­(X–H) gradually decrease from IAM to HAR to pHAR for all compounds (Figure 5). Although the reduction from HAR to pHAR may be as small as 0.001 Å, the trend is unambiguous, with a reduction ratio of roughly 10%, thereby improving statistical inferences on X–H bonds.

**Figure 4:**
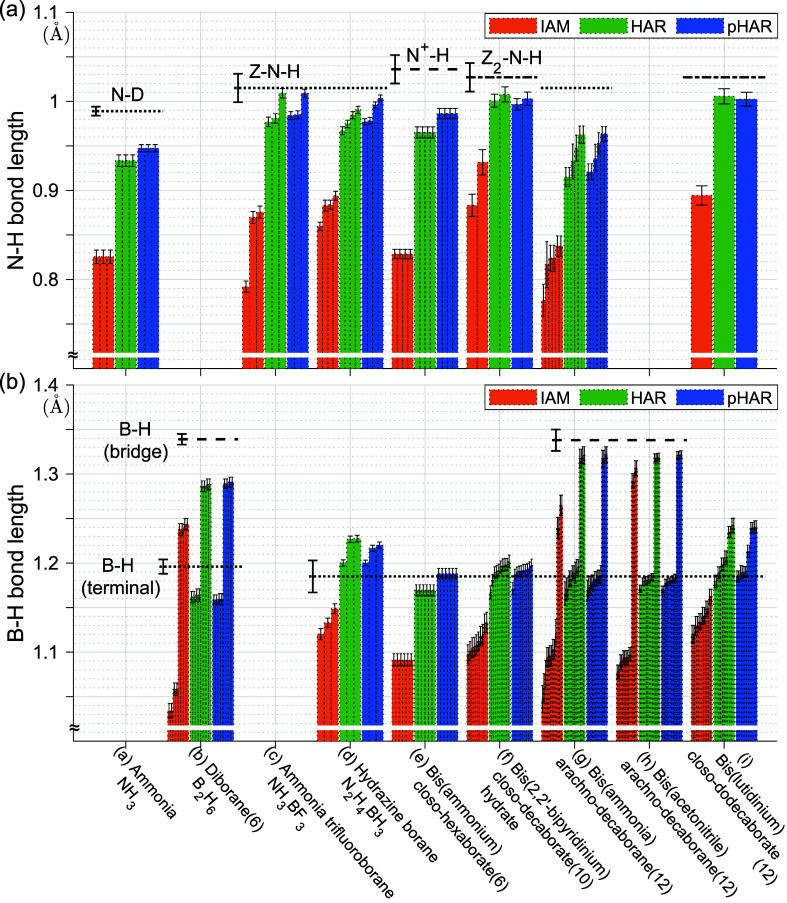
Refined (a) N–H and (b) B–H bond distances for ammonia and borane/borate systems. Error bars represent standard uncertainties derived from the variance-covariance matrix during refinement. Horizontal dotted or dashed lines indicate reference values from neutron diffraction (except for diborane, taken from electron diffraction). In boranes (b), (g), and (h), there are both terminal and bridging H atoms which require a different reference value each. For ammonia and diborane, the reference values stem from measurements of the same compounds, and the error bars corresponds to standard uncertainties from refinement.^61,63^ For the other compounds, the reference values are average lengths taken from ref 5, and the error bars represent sample standard deviations. See Figure S10 for a similar graphic for C–H and O–H bonds, mostly based on the xylitol refinement.

**Figure 5:**
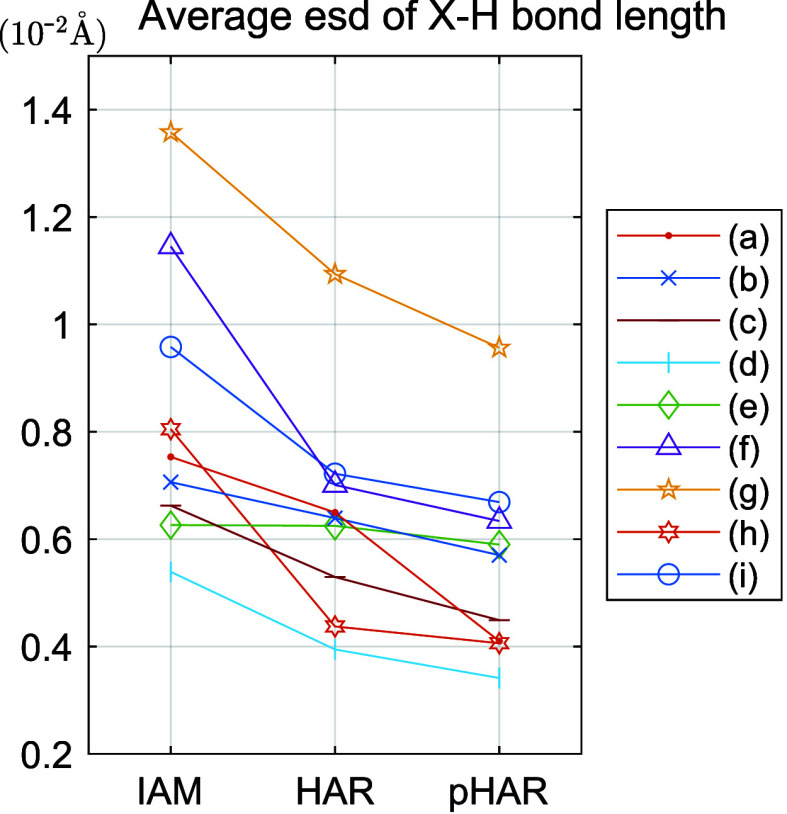
Estimated standard deviation (esd) values averaged over all B–H and N–H bonds of each system with respect to different refinement schemes. The order of the compounds (a) to (i) corresponds to that in Figure 2. See Figure S10 for a similar graphic for C–H and O–H bonds, mostly based on the xylitol refinement, showing the same trend.

Comparison with the neutron reference values (horizontal dotted/dashed lines in Figure 4) reveals that the refined N–H bond lengths are systematically underestimated, whereas most terminal B–H bond lengths agree with the reference values within a single standard uncertainty. This motivated us to extend the analysis to C–H and O–H bonds, thereby completing the B, C, N, and O series. Among the nine compounds in this study, 28 independent C–H bonds but only two O–H bonds are found. To enable aggregate analysis of the X–H bond series, we therefore added xylitol, which contains five O–H as well as seven C–H bonds, and has both X-ray and neutron diffraction data available (see the Supporting Information, section 8).^66,67^ Analysis of these bond lengths refined with HAR shows the same trend as N–H and B–H: O–H bond lengths are clearly underestimated, whereas C–H bond lengths match the reference values.^28^

Table 3 shows the average absolute deviations, Δ_X‑H_, relative to neutron references—which can be regarded as an estimate of accuracy^26^—for X = B, C, N, and O. For both HAR and pHAR, the underestimation of d­(X–H) becomes more pronounced from B to O as the electronegativity increases. We speculate that the observed differences among bond types are related to bond polarity, which is low for B–H/C–H bonds and high for N–H/O–H bonds. Since Hirshfeld atoms are based on neutral spherical atoms, this may be a source of this trend. At the same time, there is a consistent reduction in Δ_X‑H_ from HAR to pHAR, which is more apparent for more electronegative atoms that are more involved in hydrogen bonding and, thus, benefit more from the accurate treatment of the periodic environment. Although the difference in individual refined bond lengths are insignificant, the aggregate analysis reveals closer agreement with the neutron reference for pHAR than for HAR for every bond type.

**Table 3:** Average Absolute Deviations (Å) of Refined Bond Lengths with Respect to Neutron Reference Data^a^

	Δ_B–H_	Δ_C–H_	Δ_N–H_	Δ_O–H_
HAR	0.018	0.022	0.052	0.067
pHAR	0.016	0.018	0.044	0.056
Δ_X‑H_ = average(|d_(X‑H)_^refined^ – d_(X‑H)_^neutron^|)				

a Δ_B–H_ and Δ_N–H_ are obtained from boranes/borates, whereas Δ_C–H_ and Δ_O–H_ are obtained from xylitol and the cocrystallized molecules in borate compounds (see the Supporting Information, section 8).

An interesting, clearly significant chemical feature can be observed for bis­(2,6-lutidinium) closo-dodecaborate(12) in Figure 4. The 12 terminal B–H bonds in the dodecahedron are not identical. Six bonds match the literature neutron-diffraction data for terminal B–H bonds, whereas six are clearly overestimated by about 0.05 Å. Upon closer inspection of the crystal packing, four overestimated bonds are those that are in contact with N–H bonds in a B–H···H–N dihydrogen interaction, since the borane hydrogen atoms are hydridic but the ammonium N^+^-H hydrogen atoms are protic/acidic. This phenomenon has already been described for small Lewis acid–base adducts by several authors of this study elsewhere.^56^ For the other two overestimated bonds, H atoms point toward the regions with high residual density, which could be attributed to a possible minor substitutional disorder with a halogen atom. Using the newly developed pHAR method, such subtle chemical features can now be detected in terms of accurate and precise B–H bond distances from X-ray diffraction.

## Concluding Remarks

A new periodic Hirshfeld atom refinement variant, pHAR for short, has been developed. This technique can now be applied to periodic network compounds, which were previously beyond the reach of HAR (compare ref 68). Since pHAR is a natural extension of HAR, changes in chemical features induced by crystal fields can now be consistently explored at the same level of theory.

pHAR was tested on nine different borane, borate, ammonia, and ammonium systems—molecular crystals or molecular salts—thereby expanding the range of available compounds containing accurate structural information on B–H bonds. pHAR yields significant improvements in refinement statistics and residual densities over IAM, but only marginal gains compared with conventional HAR. Since the impact of the basis set—especially the use of diffuse functions—appears to be more important than the method used to simulate the crystal field, further basis set optimization is needed to ensure the utility of pHAR.

Although the influence of periodic-boundary conditions on individual X–H bond lengths is insignificant for the tested systems, aggregate analysis shows a clear decrease in average absolute deviations from neutron-diffraction values and in estimated standard deviations. The underestimation of X–H bond lengths—more pronounced with increasing bond polarity—is also mitigated in pHAR. The more polar a bond type is, the more it benefits from the correct periodic description of the environment in pHAR.

## Experimental and Computational Methods

pHAR combines Crystal23 ^52^ and Tonto.^53^ Crystal23 performs quantum-chemical calculations with periodic-boundary conditions using atom-centered Gaussian-type atomic orbitals. Tonto carries out electron-density partitioning and least-squares refinement of structural parameters. In this study, the self-consistent field (SCF) convergence threshold for the total energy was set to be 10^–7^ Hartree. The irreducible part of the Brillouin zone was sampled using the Pack-Monkhorst method with a shrinking factor of 6 for the construction of the Hamiltonian matrix. The corresponding numbers of sampling points in the Brillouin zone for triclinic, monoclinic, orthorhombic, simple cubic and face-centered cubic systems were 112, 80, 64, 24 and 16, respectively. The resulting supercell required for calculating the electron density of the reference cell typically had a radius of about 20 Å to absorb electron-density tails of the neighboring cells into the reference cell.

The data exchange between the two packages Crystal23 and Tonto was implemented at the level of density matrices. The keyword *CRYAPI*_*OUT* of Crystal23 is used to print density matrices in XML format. Tonto reads those density matrices and reproduces the electron density for each atom-centered grid system^69^ of unique atoms in the unit cell. To improve computational efficiency in calculating electron density from density matrices within Tonto, the calculation of the product of two basis functions is skipped in two cases: when the corresponding density-matrix element is zero, or when the distance between the basis-function centers is sufficiently large so that their tails fall below the threshold value of *e* ^–20^ (≈2 × 10^–9^) at the center of their counterpart. The validity of the reproduced electron density was tested by comparing density maps produced by Tonto and Crystal23 on identical grids, and agreement to at least five decimal places at every grid point was confirmed.

After reproducing electron density within Tonto, stockholder partitioning^70^ is performed to obtain Hirshfeld atoms, and the corresponding atomic form factors are calculated by Fourier transform. Structural least-squares refinement is then carried out. The CIF output from Tonto and the output file from Crystal23 are processed by lamaGOET^54^ for convergence testing. The thresholds are set to be 10^–5^ hartree for the energy change and 0.01 for the ratio parameter shift/standard uncertainty. The pHAR cycle is repeated until the output passes the convergence test. Once the iteration has converged, the residual density is calculated from the final CIF. The overall procedure is illustrated in Figure 1. Detailed instructions and known issues are provided in the Supporting Information, sections 1 and 2.

All data used in this study stem from the literature; references are given in the caption of Figure 2. The temperatures of the determinations were between 9 and 100 K, and resolutions between 1.0 and 1.3 Å^–1^. For most compounds, only merged data sets were available, and the merging procedures are described in the original publications. For IAM in Tonto, hydrogen-atom ADPs were set to be isotropic. For HAR and pHAR, all structural parameters of hydrogen atoms were refined freely, including their anisotropic ADPs. Unless otherwise specified, HAR and pHAR were performed by using the pob-TZVP-rev2 basis set^71^ and the B3LYP hybrid functional,^72,73^ which is motivated by their widespread use. For HAR, the smallest neutral formula unit was chosen, and no cluster charges were used for the simulation of the crystal environment. For the cocrystal compounds, whose molecular configurations of neutral formula units are not unique, the geometries for the electron-density calculation are provided in the Supporting Information, section 4.

HAR in Tonto requires atomic form factors that respect the site symmetries for atoms in special positions. For pHAR, this is now automatically given; and was part of the motivation for this method development. However, for conventional HAR, when molecules are selected for electron-density calculations without preserving each atom’s site symmetry, the corresponding atomic form factors become asymmetric, often causing problems in the refinement of high-symmetry systems. A practical procedure for the symmetrization of the Hirshfeld atom density was suggested in ref 25, which stated in section 2.4 that “details of the efficient implementation of this important procedure in Tonto will be reported elsewhere in due course”. In this study, we have now equipped Tonto with a subroutine for form-factor symmetrization. For all atoms in special crystallographic positions, atomic form factors are averaged over their site symmetries. It makes conventional HAR applicable to structures with atoms in special positions irrespective of their environment. Only with this implementation, all the comparisons between HAR and pHAR in this paper have become possible. Meanwhile, for pHAR, this procedure enhances the numerical stability of least-squares matrices (see the Supporting Information, section 3).

Similarly, ADP tensors are symmetrized by averaging over their site symmetry. This restores any broken symmetry that may have occurred during the rounding process of ADPs when recording in CIF, and reinforces the numerical stability.

## Supplementary Material





## Data Availability

The crystallographic information files including lists of measured structure factors for pHAR are available from the Cambridge Structural Database under deposition numbers 2481159–2481170. They can be obtained free of charge from https://www.ccdc.cam.ac.uk/structures.

## Notes

Beginner’s guide for pHAR, details about symmetrization of atomic form factors, geometries for the DFT calculations of HAR, comparison with XHARPy, residual-density comparison with the multipole model, absolute values of Figure 4, and xylitol refinement for O–H and C–H bonds (PDF)
Relevant crystallographic information files including IAM refinement, conventional HAR, and periodic HAR (ZIP)
