# Supplementary material for: Periodic Hirshfeld Atom Refinement
Source: J Phys Chem Lett. 2026 Feb 27;17(11):3170–9. doi: 10.1021/acs.jpclett.5c03918 (PMC13007019; doi:10.1021/acs.jpclett.5c03918)
Supplement: Supplementary file 1 [file jz5c03918_si_001.pdf]

Periodic Hirshfeld Atom Refinement Supporting Information

Kanghyun Chu,[†] Dylan Jayatilaka,[‡,¶] Lorraine A. Malaspina,[†] Alessandro Genoni,[§] Georgia Cametti,[‖] Stefan Mebs,[⊥] Dieter Lentz,[#] Hans-Beat Bürgi,[†] Sergey V. Churakov,[‖,@] and Simon Grabowsky*,[†]

†*Department of Chemistry, Biochemistry and Pharmaceutical Sciences, University of Bern, Freiestrasse 3, 3012 Bern, Switzerland*

‡*Research Group for Structural Biochemistry and Mechanisms, Max-Planck Institute for Multidisciplinary Sciences, Am Fassberg 11, 37077 Göttingen, Germany;*

¶*School of Molecular Sciences, University of Western Australia, 35 Stirling Highway, Crawley WA 6009, Australia*

§*Department of Chemistry, Materials and Chemical Engineering "Giulio Natta", Politecnico di Milano, Via Bassini, 20133 Milano, Italy*

‖*Institute of Geological Sciences, University of Bern, Baltzerstrasse 1+3, 3012 Bern, Switzerland*

⊥*Department of Physics, Free University of Berlin, Arnimallee 14, 14195 Berlin, Germany*

#*Institute of Chemistry and Biochemistry, Free University of Berlin, Fabeckstr. 34/36, 14195 Berlin, Germany*

@*PSI Center for Nuclear Engineering and Sciences, Paul Scherrer Institute, Forschungsstrasse 111, 5232 Villigen PSI, Switzerland*

E-mail: simon.grabowsky@unibe.ch

1. pHAR setup instruction

Here, we provide instructions for setting up and running the program for periodic Hirshfeld atom refinement (pHAR). As mentioned in the main text, pHAR is based on three core software packages: Tonto, lamaGOET, and Crystal23. pHAR is optimized for a Linux environment.

1.1 Installing Tonto

To download and compile Tonto, type the following lines in your Linux command prompt:

```
> cd ~
> git clone -b release-no-ptr –recursive https://github.com/dylan-jayatilaka/tonto.git
> cd ~/tonto
> mkdir build
> cd build
> cmake ..
> make -j
```

The executable file will be created at the following path: "~/tonto/build/tonto". For more details, check the following link:

https://github.com/dylan-jayatilaka/tonto/tree/release-no-ptr?tab=readme-ov-file

1.2 Installing lamaGOET

Type the following command into the prompt to download lamaGOET.

```
> cd ~
> git clone -b periodic https://github.com/lomalaspina/lamaGOET.git
```

There is no need to compile. Please refer to the supplementary material of ref. 1 for more details.

1.3 Installing Crystal23

Crystal23 is a commercial software program. A license can be purchased under https://www.crystal.unito.it. Instructions for installing Crystal23 are found in the Crystal23 manual. See:

https://www.crystal.unito.it/include/manuals/CRYSTAL23_installation.html

Prepare a shortcut for executing Crystal23, for example, *runcry23*.

1.4 Launching pHAR

Before starting pHAR, prepare a CIF and a Tonto-compatible hkl file. For the hkl file format, Tonto supports both structure factor magnitudes F^2 and F. For hkl files in F^2, refer to Table S1 for the file format. For those in F, modify the second line of the table to "keys= { h= k= l= F_exp= F_sigma }". In both cases, the symmetry-equivalent reflections must be merged in advance, which can be done using packages such as WinGX*, xprep or others. Refinement in Tonto is always carried out in F, regardless of the input hkl format.

Start pHAR by typing *lamaGOET* into the command prompt. A graphical user interface is provided, as shown in Figure S1. Select **Crystal23** in the radio button menu at the top[1)]. Enter the shortcuts or paths of Tonto and Crystal23[2),3)]. Enter the name of your project.

*https://www.chem.gla.ac.uk/~louis/software/wingx/

Table S1: The format of a Tonto-compatible hkl file

```
reflection_data= {
    keys= { h= k= l= I_exp= I_sigma }
    data= {
        0  0  1  300.00  5.00        % Data block here
        0  1  0  256.00  4.00
                  ⋮
        9  9  9    1.00  0.10
    }
}
REVERT
```

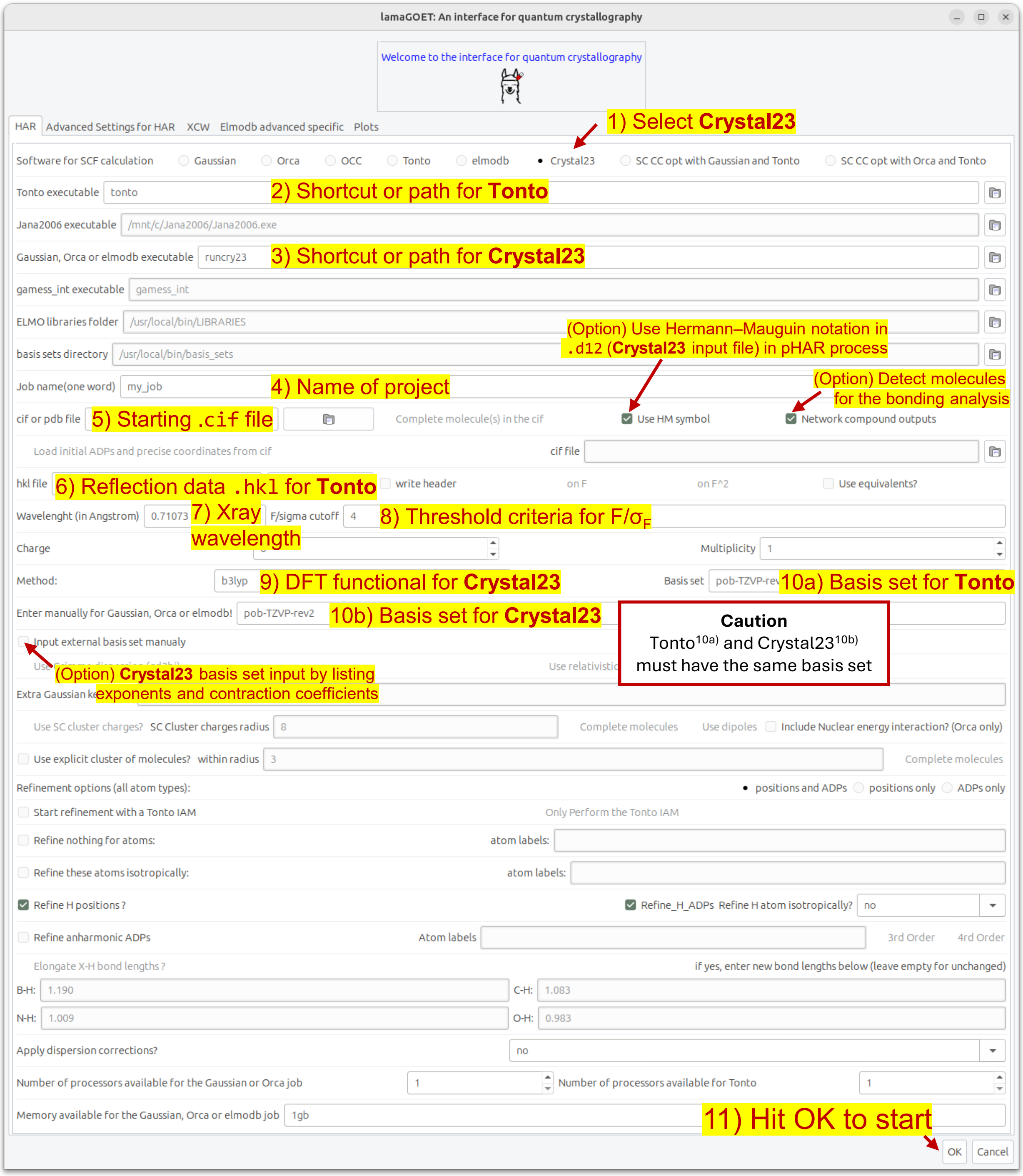

Figure S1: lamaGOET GUI with brief instructions for pHAR

This name will be the name of the output files[4)]. Select the initial CIF and hkl files[5),6)]. Enter the wavelength of the X-radiation used in the experiment[7)]. Set a rejection criterion for the reflection data, $F_{\mathrm{obs}}/\sigma_F$ [8)]. For beginners, a value of 4 is recommended, which is comparable

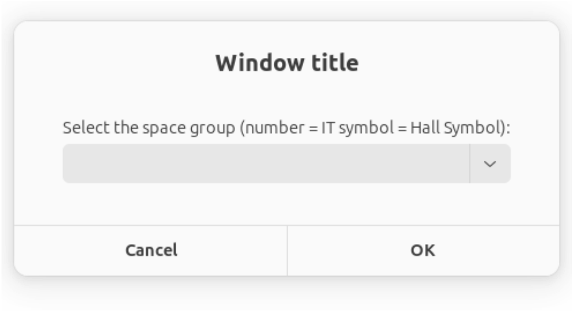

Figure S2: Pop-up window for space group

to $I_{\mathrm{obs}}/\sigma_I < 2$. Select an exchange-correlation functional for the DFT calculation[9)], B3LYP is recommended, or select Hartree-Fock (HF). Select a basis set for Tonto by using the drop-down list[10a)]. It is recommended to use STO-3G for testing and pob-TZVP-rev2 for production runs. Type the name of the chosen basis set for use in Crystal23[10b)]. For advanced users wanting to use different basis sets, see section 2.2. Hit "OK" to start pHAR[11)].

If the project is launched for the first time, a pop-up window will appear asking for a space group (Figure S2). Hit "OK" without choosing an option from the drop-down menu to automatically detect the space group in the CIF and proceed with pHAR. If more than one origin or axes setting is associated with your space group number, the auto-detection may fail. In this case, choose the correct origin or axes setting from the menu to override automatic space group selection. The pop-up window will create "spacegroup.txt", and will never appear again when you repeat the project, unless the file has been removed.

Once the pHAR process is complete, the command prompt will be returned and you can find a summary of the result in the file named "*project_ name*.lst". This file contains the initial geometry, the evolution of the statistics over each iteration step, the final geometry, residual density, etc. The refined structural parameters, structure factors (calculated and observed), and the corresponding residual density data in a cube file format are provided in the files "*project_ name*.archive.cif", "*project_ name*.archive.fcf"(or .fco), and "*project_ name*.residual_density,cell.cube", respectively.

2. Known issues, cautions, and solutions

2.1 Choice between the space group number and the Hermann-Mauguin (HM) symbol

It is recommended to enable the "use HM symbol" checkbox (right side of Figure S1[5)]) when your system has multiple settings for origins and axes ordering (e.g. monoclinic or orthorhombic). A typical example is space group #14, which has two popular axes settings out of nine possible ones: $P\ 2_1/c$ and $P\ 2_1/n$. If the checkbox is not enabled, space group #14 will always be interpreted as $P\ 2_1/c$, and pHAR will fail if $P\ 2_1/n$ is the setting in the input files.

For cubic space groups #221-230, **uncheck** the "use HM symbol" checkbox, because Crystal23 accepts only 3 but not $\bar{3}$ as a valid space group identifier for those cubic systems. Therefore, $\bar{3}$ in the HM symbol will cause Crystal23 to crash. The problem is avoided by using the space group number instead of the HM symbols. Please contact the authors if you find any other inconsistencies in the interpretation of the space groups between Tonto and Crystal23.

2.2 Using custom basis sets and the ordering issue

Custom basis sets are supported. If the basis set of your choice is not available in the presets of either Tonto or Crystal23 or both, follow the steps below. At present, only the basis sets STO-3G and pob-TZVP-rev2 are available as default basis sets common to both software programs.

To add a custom basis set in Tonto, create a basis set file in the directory: "~/tonto/basis_set/". The easiest way is to make a copy of a pre-existing basis set file, rename it, and edit it. The newly introduced basis set will not appear automatically in the drop-down menu of Figure S1[10a)]. Instead, type its name into the field. Once the new basis set file has been created, it is available for repeated use.

To employ a custom basis set for Crystal23, enable the checkbox "Input external basis

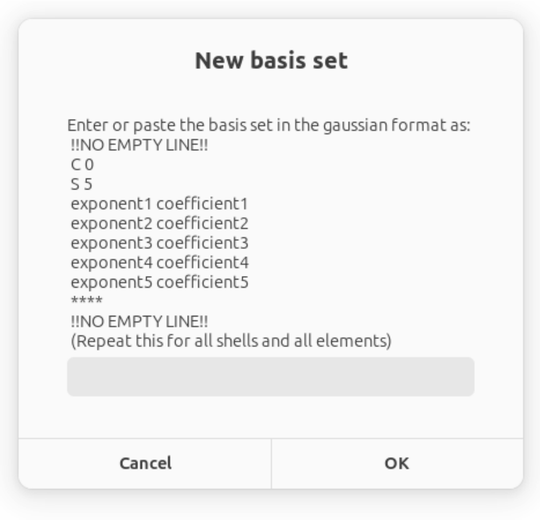

Figure S3: Pop-up window for Crystal23 basis set input

set manually” below “10b) Basis set for **Crystal23**” in Figure S1. After confirming with “OK” in Figure S1[11)], a pop-up window will appear as shown in Figure S3. Prepare a basis set block for the Crystal23 input file, and paste it into the provided text field. The formatted basis sets are available via the following link: https://www.crystal.unito.it/basis_sets.html. If the basis set was downloaded from the Basis Set Exchange (https://www.basissetexchange.org), refer to the Crystal23 manual and edit the initial occupation number of each orbital. Paste the copied text block into the text input field in Figure S3. Do not include any blank lines or the end-of-basis-set indicator, “99 0”. The text block will be saved in a file named “basis_gen.txt” and automatically inserted into the basis set block of every d12 file created by lamaGOET. The pop-up window will not reappear unless the file has been deleted from the working folder.

For pHAR to work correctly, the **basis set ordering** in Crystal23 must match that in Tonto. If it does not, the pHAR result will be incorrect as may be seen from the R-values which become significantly worse than those of IAM. It is recommended to always compare the basis set ordering in Crystal23 with that in Tonto. The ordering used by Tonto can be found in “∼/tonto/basis_set/”. To obtain a corresponding basis set for Crystal23, copy the desired basis set from the folder and relabel the orbital identifiers according to the Crystal23

Table S2: 6-311G(d,p) basis function of nitrogen for Crystal23

Basis set from the Crystal23 library			Reordered to be Tonto-compatible	
7 5			7 8	
0 0 6 2 1			0 0 6 2 1	
6293.48	0.00196979		6293.48	0.00196979
949.044	0.0149613		949.044	0.0149613
218.776	0.0735006		218.776	0.0735006
63.6916	0.248937		63.6916	0.248937
18.8282	0.60246		18.8282	0.60246
2.72023	0.256202		2.72023	0.256202
0 1 3 5 1			0 0 3 2 1	
30.6331	0.111906	0.0383119	30.63310	0.111906
7.02614	0.921666	0.237403	7.026140	0.921666
2.11205	-0.00256919	0.817592	2.112050	-0.00256919
0 1 1 0 1			0 0 1 0 1	
0.684009	1.00	1.00	0.684009	1.00
0 1 1 0 1			0 0 1 0 1	
0.200878	1.00	1.00	0.200878	1.00
0 3 1 0 1			0 2 3 3 1	
0.913	1.00		30.6331	0.0383119
			7.02614	0.237403
			2.11205	0.817592
			0 2 1 0 1	
			0.684009	1.00
			0 2 1 0 1	
			0.200878	1.00
			0 3 1 0 1	
			0.913	1.00

manual.

An example of a non-matching basis set is 6-311G(d,p) as exemplified for nitrogen in Table S2. The problem can be resolved by reordering the basis functions. Specifically, each sp-block should be separated into two distinct blocks and the orbital identifier should be adjusted accordingly. Do not load the 6-311G(d,p) basis set by putting its name in Figure S1[10b)]. Instead, enable the “Input external basis set manually" checkbox below the label “10b)”, and paste the reordered basis set into the text field of Figure S3.

2.3 Ordering of ADPs

The off-diagonal ADP tensor components in the final CIFs generated by Tonto are ordered lexicographically as U_{12}, U_{13}, U_{23}, rather than U_{23}, U_{13}, U_{12} as in Voigt notation.

2.4 Updating "_refine_diff_density" in final CIF manually

For each least-squares cycle, Tonto creates a CIF of an intermediate structure, and lamaGOET conducts a convergence test. The final CIF does not contain residual density information. However, this information can be found in the files named "stdout" and "*project_name*.lst", in both files specifically in the section:

```
=====================
Residual density data
=====================
.  Grid has voxels less than 0.1 A per side
.  Length units are in Angstrom
        ⋮
```

Copy the values of "Maximum ..." , "Minimum ..." , "RMS ...", and paste them into the fields "_refine_diff_density_max" , "_refine_diff_density_min" , "_refine_diff_density_rms" of the final CIF, respectively.

2.5 Defining molecules for the DFT calculation of HAR

It is not unusual in conventional HAR that the cluster of molecules needed for a meaningful molecular wavefunction calculation exceeds the asymmetric unit atoms. In order to specify the molecular cluster for electron-density calculation, all the atoms comprising those molecules must be included in the input CIF. Any inconsistency in fractional coordinates of symmetry-related atoms will cause Tonto to crash.

3. Symmetrization of atomic form factors

For a given space group symmetry, some structural parameters for atoms in positions with symmetry (special positions) may be constrained by the symmetry itself (stabilizer group). Such constraints take particularly simple forms if described in the respective crystal coordinate system (See International Tables for Crystallography, Vol. A for atomic coordinates, and International Tables for Crystallography (2013), Vol. D ch. 1.9, pp. 231-245 for ADPs). In Tonto, HAR and pHAR use Cartesian coordinate systems throughout. Hence, the formulation of the constraints is more complicated, especially for the non-orthogonal triclinic, monoclinic, rhombohedral and hexagonal crystal systems. Constraints are therefore introduced indirectly by diagonalizing the (singular) least-squares matrix computed with all structural parameters, constrained and unconstrained, and inverting the non-singular part. To achieve the required singularity, it is important that the atomic form factors of atoms in special positions obey their site symmetry.

There are two situations where this is not the case:

1) The atomic arrangement chosen for the quantum chemical calculation around the atoms with site symmetry may not obey the respective site symmetry as is often the case for HARs (see following section on 'Geometry for the DFT calculation of HAR' for examples) but is not a problem for pHAR by definition of the periodic environment.

2) Atomic electron densities are sampled on a Becke grid (with cubic symmetry m3m of the grid points, but not of the density values). Problems arise if the grid is not oriented such that (some of) its symmetry operations match the full site symmetry in question.

As a consequence of 1) and 2), here we have derived and implemented a procedure in which the form factors of atoms in special positions are always symmetrized. This also provides a quantitative measure of the deviation from symmetry (RSS), as described below.

Consider a partitioned electron density ρ_i, which is the product of the electron density obtained from the DFT calculations and the Hirshfeld partitioning function. ρ_i is positive definite with a finite integral and it has its main features around the position of atom i. The

atomic form factor of i is the Fourier transform[†] of ρ_i, *i.e.*

$$f_i(\mathbf{K}) = \int_V \rho_i(\mathbf{r}) \exp(\mathrm{i}\mathbf{K} \cdot \mathbf{r})\, \mathrm{d}\mathbf{r}. \tag{1}$$

$\mathbf{K}$ denotes a wave vector in reciprocal space, represented as a 1-by-3 row vector; $\mathbf{r}$ denotes a position vector in direct space, represented as a 3-by-1 column vector.

If i is at a special position, ρ_i must be symmetric with respect to the site symmetry group $\mathbf{S}$ (stabilizer group). As mentioned above, the practical details of calculating ρ_i may lead to smaller or larger deviations from a symmetric atomic form factor implying that $\rho_i(\mathbf{r}) \neq \rho_i(S_s\mathbf{r})$, or equivalently $f_i(\mathbf{K}) \neq f_i(\mathbf{K}S_s^T)$.[‡] S_s is the 3-by-3 rotation or rotoinversion matrix for the s-th stabilizer symmetry operator. In order to guarantee the correct number of zero eigenvalues of the least-squares matrix, the symmetrized atomic electron density and the corresponding atomic form factor ($\rho_i^{\mathrm{sym}}(\mathbf{r})$ and $f_i^{\mathrm{sym}}(\mathbf{K})$ must be invariant under every symmetry operation S_s of $\mathbf{S}$, *i.e.*

$$\rho_i^{\mathrm{sym}}(S_s\mathbf{r}) = \rho_i^{\mathrm{sym}}(\mathbf{r}) \quad \text{or equivalently} \quad f_i^{\mathrm{sym}}\big(\mathbf{K}S_s^T\big) = f_i^{\mathrm{sym}}(\mathbf{K}). \tag{2}$$

Also, the symmetrization must not change the total charge, *i.e.*

$$\int_V \rho_i(\mathbf{r})\, d\mathbf{r} = \int_V \rho_i^{\mathrm{sym}}(\mathbf{r})\, d\mathbf{r} \quad \text{or equivalently} \quad f_i(\mathbf{0}) = f_i^{\mathrm{sym}}(\mathbf{0}), \tag{3}$$

where $\mathbf{0}$ is the zero vector in reciprocal space.

The average of the symmetry-transformed atomic form factors obtained with all stabilizer symmetry operators satisfies the conditions (2) and (3).[§] Therefore, the symmetrized atomic

[†]We adhere to the crystallographic convention that the kernel of the forward transform is $\exp(\mathrm{i}\mathbf{K} \cdot \mathbf{r})$.

[‡]See *Proof1* on page 14.

[§]See *Proof2* on page 14.

form factor f_i^{sym} is given by

$$f_i^{\text{sym}}(\mathbf{K}) = \frac{1}{|\mathbf{S}|}\sum_{s=1}^{|\mathbf{S}|} f_i\big(\mathbf{K}S_s^T\big). \tag{4}$$

$|\mathbf{S}|$ is the order of $\mathbf{S}$. Figure S4 shows symmetrization of a non-symmetric form factor for an atom at a site with 4-fold symmetry.

Before examining the computational details of Equation 4, it is worth mentioning that, in practice, an integration over the entire space is approximated by a summation over grid points, as follows:

$$\int_V g(\mathbf{r})\; d\mathbf{r} \simeq \sum_n g(\mathbf{r}_n)\,\Delta V_n \tag{5}$$

where $g(\mathbf{r})$ is an arbitrary function such as the integrand in Equation 1, $\mathbf{r}_n$ and ΔV_n are the position and the volume associated with the n-th grid point. Expressed in terms of partitioned electron densities, Equation 4 becomes

$$\begin{aligned} f_i^{\text{sym}}(\mathbf{K}) = \frac{1}{|\mathbf{S}|}\Bigg(&\Big(\sum_n \rho_i(\mathbf{r}_n) e^{\mathbf{iKS}_1^{\mathrm{T}}\mathbf{r}_\mathrm{n}}\;\Delta V_n\Big) + \Big(\sum_n \rho_i(\mathbf{r}_n) e^{\mathbf{iKS}_2^{\mathrm{T}}\mathbf{r}_\mathrm{n}}\;\Delta V_n\Big) + \cdots \\ &+ \Big(\sum_n \rho_i(\mathbf{r}_n) e^{\mathbf{iKS}_{(|\mathbf{S}|-1)}^{\mathrm{T}}\mathbf{r}_\mathrm{n})}\;\Delta V_n\Big) + \Big(\sum_n \rho_i(\mathbf{r}_n) e^{\mathbf{iKS}_{|\mathbf{S}|}^{\mathrm{T}}\mathbf{r}_\mathrm{n}}\;\Delta V_n\Big)\Bigg). \end{aligned} \tag{6}$$

By convention, S_1^T is the identity matrix, which is always a stabilizer symmetry operator. The partitioned electron density ρ_i is evaluated only at the grid points $\mathbf{r}_n$ of ρ_i, and an expensive calculation on the rotated grids $S_s\mathbf{r}_n$ is unnecessary. Instead, f_i is evaluated in the rotated reciprocal spaces $\mathbf{K}S_s^T$.

The influence of symmetrization on the atomic form factor is quantified by the residual sum of squares (RSS),

$$\text{RSS} = \sum_{\mathbf{K}} \Big| f_i(\mathbf{K}) - f_i^{\text{sym}}(\mathbf{K}) \Big|^2. \tag{7}$$

In Tonto, this summation is performed over the domain specified in the hkl file, not over the entire reciprocal space. The RSS values are given in the Tonto output file.

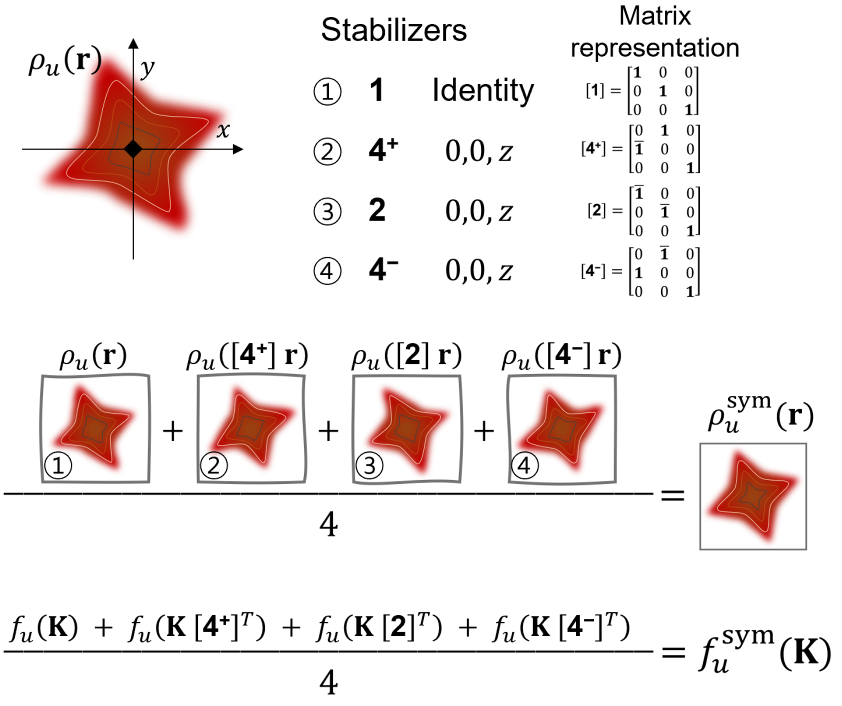

Figure S4: Schematic representation of the symmetrization of a partitioned atomic electron density $\rho_i(\mathbf{r})$ sitting on a 4-fold symmetry axis. Neither $\rho_i(\mathbf{r})$ nor the corresponding atomic form factor $f_i(\mathbf{K})$ obey the site symmetry. The symmetrized objects, $\rho_i^{\mathrm{sym}}(\mathbf{r})$ and $f_i^{\mathrm{sym}}(\mathbf{K})$, are obtained by averaging over all the symmetry-transformed objects.

Proof1. **Fourier transform of $\rho_i(S\mathbf{r})$**

It is enough to show that the Fourier transform of $\rho_i(S\mathbf{r})$ is $f_i(\mathbf{K}S^T)$.

$$\begin{aligned}\int_V \rho_i(S\ \mathbf{r}) \exp(\mathrm{i}\mathbf{K}\cdot\mathbf{r})\ \mathrm{d}\mathbf{r} &= \int_V \rho_i(S\ \mathbf{r}) \exp\Big(\mathrm{i}\mathbf{K}\cdot(\mathrm{S}^{-1}\mathrm{S}\ \mathbf{r})\Big)\ \mathrm{d}\mathbf{r} \\ &= \int_V \rho_i(\mathbf{r}') \exp\Big(\mathrm{i}\mathbf{K}\cdot\mathrm{S}^{\mathrm{T}}(\mathrm{S}\mathbf{r})\Big)\left\|\frac{\partial\mathbf{r}}{\partial\mathbf{r}'}\right\|\ \mathrm{d}\mathbf{r}' \\ &= \int_V \rho_i(\mathbf{r}') \exp\Big(\mathrm{i}(\mathbf{K}\mathrm{S}^{\mathrm{T}})\cdot\mathbf{r}'\Big)\ \mathrm{d}\mathbf{r}' \\ &= f_i\big(\mathbf{K}S^T\big)\end{aligned} \tag{8}$$

Note that S is an orthogonal matrix (a real unitary matrix) satisfying $S^{-1} = S^T$, since it represents a rotation or rotoinversion operation. $\left\|\frac{\partial\mathbf{r}}{\partial\mathbf{r}'}\right\|$ is the Jacobian determinant, which equals $\left|\det(S^T)\right|$. For rotation or rotoinversion matrices, its value is 1.

Proof2. **Equation 4 satisfies Equations 2 and 3**

For Equation 2, applying any stabilizer symmetry operation to $f_i^{\mathrm{sym}}(\mathbf{K})$ yields an invariant result due to the closure property of the group operation.

$$\begin{aligned}f_i^{\mathrm{sym}}\big(\mathbf{K}S^T\big) &= \frac{1}{|\mathbf{S}|}\sum_{s=1}^{|\mathbf{S}|} f_i\Big(\big(\mathbf{K}S^T\big)S_s^T\Big) \\ &= \frac{1}{|\mathbf{S}|}\sum_{s=1}^{|\mathbf{S}|} f_i\Big(\mathbf{K}\big(S^TS_s^T\big)\Big) \\ &= \frac{1}{|\mathbf{S}|}\sum_{s'=1}^{|\mathbf{S}|} f_i(\mathbf{K}S_{s'}^T) \qquad \text{(permutation of terms only)} \\ &= f_i^{\mathrm{sym}}(\mathbf{K})\end{aligned} \tag{9}$$

For Equation 3, let us evaluate $f_i^{\mathrm{sym}}(\mathbf{0})$, and it can be shown that $f_i^{\mathrm{sym}}(\mathbf{0}) = f_i(\mathbf{0})$.

$$f_i^{\mathrm{sym}}(\mathbf{0}) = \frac{1}{|\mathbf{S}|}\sum_{s=1}^{|\mathbf{S}|} f_i\big(\mathbf{0}\cdot S_s^T\big) = \frac{1}{|\mathbf{S}|}\sum_{s=1}^{|\mathbf{S}|} f_i(\mathbf{0}) = \frac{1}{|\mathbf{S}|}|\mathbf{S}|f_i(\mathbf{0}) = f_i(\mathbf{0}) \tag{10}$$

4. Geometry for the DFT calculation of HAR

In this work, conventional HAR is performed with isolated molecules or single formula units. For salt compounds and cocrystals, there is no unique way to arrange the ionic or molecular components in such a way that the resulting configurations are charge-neutral. Here, we provide the molecular configurations for the ionic compounds (e),(f), and (i) (Figure S5).

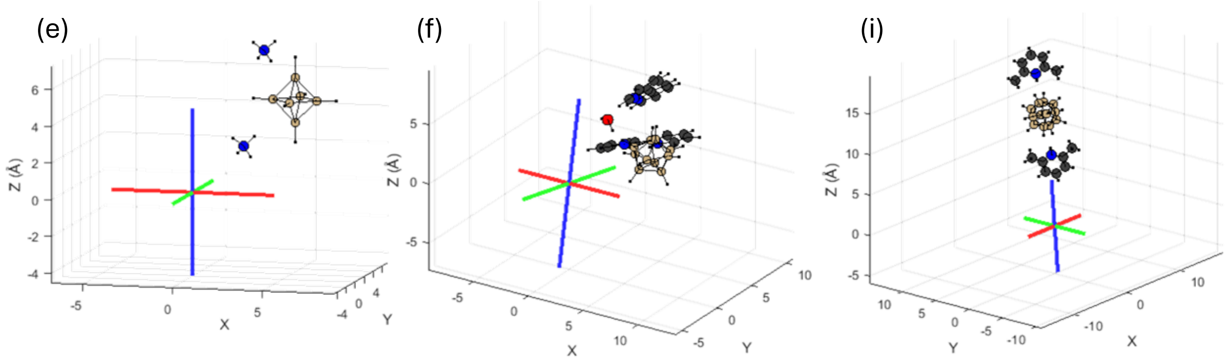

Figure S5: The geometry for the electron-density calculation in HAR. (e) Bis(ammonium) closo-hexaborate(6), (f) bis(2,2'-bipyridinium) closo-decaborate(10) hydrate, (i) bis(2,6-lutidinium) closo-dodecaborate(12)

Each partitioned electron density and its form factor are symmetrized with respect to the site symmetry. RSS values[¶] for each asymmetric unit atom are reported in *stdout*. Table S3 shows the RSS from HAR for (e) obtained from 403 independent reflections.[‖] In contrast, the RSS values obtained from pHAR for this compound are all below 10^{-6}, indicating that the crystal symmetry is preserved in the electron-density calculation.

Table S3: RSS values of atomic form factors from HAR of $(NH_4)_2B_6H_6$

Atom	site symmetry	Number of stabilizers	RSS	Remark
N	$\bar{4}3m$	24	0.009521	
B	$4m.m$	8	0.007910	
H	$4m.m$	8	0.001674	belonging to $(B_6H_6)^{2-}$
H1A	$.3m$	6	0.004686	belonging to NH_4^+

[¶]Residual Sum of Squares. See Equation 7 in the previous section.

[‖]Refer to the fields "_refine_ls_number_reflns" or "_reflns_number_gt" in the provided CIF.

5. Comparison with XHARPy

The pHAR results for L-alanine (at 23 K) and magnesium bis(hydrogen maleate) hexahydrate (at 12 K) are compared with the XHARPy results by P. N. Ruth *et al.*.[2] The following metrics were analyzed: the least-squares agreement factor $wR_2(F^2)$, the bond length deviation $|\Delta r|$, and the ADP overlap S_{12}.[3] For this comparison, we performed pHAR using the same XRD data as in Ruth's study and evaluated the results against the neutron reference data for L-alanine and magnesium bis(hydrogen maleate) hexahydrate.

In our work, least-squares optimization was performed on F (the structure factor amplitude), whereas in Ruth's work it was performed on F^2. This leads to differences in the calculation of wR. For pHAR, wR is $wR_2(F)$ with $w = \frac{1}{(\sigma_F)^2}$. In contrast, for XHARPy, wR corresponds to $wR_2(F^2)$ with $w = \frac{1}{(\sigma_{F^2})^2}$.

Overall, XHARPy with the SCAN functional yields more accurate results for most parameters than pHAR with the pob-TZVP-rev2 basis set and the B3LYP exchange functional. Note that pHAR still has room for optimizing in both its basis sets and exchange–correlation functionals.

5.1 L-alanine at 23 K

We compared the following CIFs: "pHAR_A23K.cif" and "pHAR_A23K_Fcut3.cif" from our supporting materials; CCDC 1917813 by L. A. Malaspina[4] (neutron reference); and the "data_A23_xharpy_SCAN" and "data_A23_xharpy_SCAN_iovs_cut" CIF data blocks from the supplementary materials of ref. 2.** For pHAR and XHARPy, the first CIF was refined with a rejection threshold of $F/\sigma_F < 4.0$, the second with $F/\sigma_F < 3.0$. The neutron data are used as reference in both pHAR and XHARPy.

The atom labels in the CIFs are inconsistent; Table S4 provides the correspondence among them. Table S5, S6 and S7 show the refinement statistics, the bond length analysis, and the ADP overlap, respectively.

**https://doi.org/10.1107/S2052252522001385/fc5060sup1.cif

Table S4: L-alanine atom label conversion table

pHAR	N	C1	C2	C3	H1	H2	H3	H4
neutron	N1	C3	C1	C2	H4	H1	H2	H3
XHARPy	N1	C3	C1	C2	H4	H1	H2	H3

The labels not shown here are consistent.

Table S5: L-alanine refinement statistics, unit of ρ is e Å^{-3}

	R	wR	$\rho_{\max}$	$\rho_{\min}$	ρ_{rms}
pHAR_A23K	0.0190	0.0161	0.2052	-0.1802	0.0405
pHAR_A23K_Fcut3	0.0194	0.0161	0.2180	-0.1856	0.0413
A23_xharpy_SCAN	0.0185	0.0303	0.1700	-0.1874	0.0428
A23_xharpy_SCAN_iovs_cut	0.0183	0.0300	0.1644	-0.1750	0.0400

Table S6: L-alanine bond length analysis

pHAR (pHAR_A23K)			Neutron		XHARPy (A23K_xharpy_SCAN)		
	...	..._Fcut3	(reference)			...	..._iovs_cut
O1-C1	1.2464(3)	1.2464(3)	O1-C3	1.2479(9)	O1-C3	1.2473(4)	1.2473(4)
O2-C1	1.2651(4)	1.2651(4)	O2-C3	1.2661(8)	O2-C3	1.2663(4)	1.2663(4)
N-C2	1.4898(4)	1.4898(4)	N1-C1	1.4891(7)	N1-C1	1.4896(4)	1.4897(4)
N-H2	0.992(6)	0.992(6)	N1-H1	1.0351(16)	N1-H1	1.015(6)	1.016(6)
N-H3	1.012(6)	1.012(6)	N1-H2	1.0439(16)	N1-H2	1.024(6)	1.027(6)
N-H4	1.052(6)	1.052(6)	N1-H3	1.0534(15)	N1-H3	1.054(7)	1.058(7)
C1-C2	1.5346(4)	1.5347(4)	C1-C3	1.5360(7)	C1-C3	1.5348(4)	1.5348(4)
C2-C3	1.5267(4)	1.5267(4)	C1-C2	1.5266(8)	C1-C2	1.5264(4)	1.5264(4)
C2-H1	1.094(5)	1.094(5)	C1-H4	1.0993(15)	C1-H4	1.100(5)	1.101(5)
C3-H5	1.094(6)	1.094(6)	C2-H5	1.0919(19)	C2-H5	1.097(6)	1.100(7)
C3-H6	1.079(6)	1.079(6)	C2-H6	1.0945(18)	C2-H6	1.088(6)	1.090(7)
C3-H7	1.083(5)	1.083(5)	C2-H7	1.095(2)	C2-H7	1.088(6)	1.090(6)
$\sum \lvert\Delta r\rvert_{\text{all}}$	0.1160	0.1159			$\sum \lvert\Delta r\rvert_{\text{all}}$	0.0626	0.0627
$\sum \lvert\Delta r\rvert_{\textbf{X-H}}$	0.1113	0.1113			$\sum \lvert\Delta r\rvert_{\textbf{X-H}}$	0.0599	0.0599

(Unit: Å)

Table S7: ADPs and the ADP overlap S_{12} of L-alanine. The labels in the brackets are the ones from the neutron CIF and the XHARPy CIF. (Refer to Table S4)

(Unit: Å^2)		pHAR	...Fcut3	Neutron	XHARPy	...iovs_cut
O1	U11	0.00666(7)	0.00667(7)	0.00683(19)	0.00681(6)	0.00681(7)
	U22	0.00689(7)	0.00689(7)	0.0073(2)	0.00722(6)	0.00722(6)
	U33	0.00549(9)	0.00549(9)	0.00660(18)	0.00584(8)	0.00584(8)
	U12	0.00235(6)	0.00235(6)	0.00243(16)	0.00246(6)	0.00246(6)
	U13	-0.00057(6)	-0.00057(6)	-0.00065(15)	-0.00061(6)	-0.00061(6)
	U23	0.00048(6)	0.00048(6)	0.00043(15)	0.00051(6)	0.00051(6)
	S_{12}	0.248%	0.247%	0	0.105%	0.105%
O2	U11	0.00740(7)	0.00740(7)	0.0078(2)	0.00763(6)	0.00764(7)
	U22	0.00733(7)	0.00733(7)	0.0083(2)	0.00764(6)	0.00764(6)
	U33	0.00383(8)	0.00383(8)	0.00475(17)	0.00406(7)	0.00407(7)
	U12	0.00195(6)	0.00195(6)	0.00197(15)	0.00200(6)	0.00201(6)
	U13	0.00050(7)	0.00051(6)	0.00051(15)	0.00056(6)	0.00056(6)
	U23	-0.00048(6)	-0.00048(6)	-0.00070(14)	-0.00057(5)	-0.00057(6)
	S_{12}	0.421%	0.422%	0	0.218%	0.214%
N	U11	0.00557(8)	0.00557(7)	0.00591(12)	0.00562(7)	0.00563(7)
(N1)	U22	0.00598(7)	0.00598(7)	0.00692(13)	0.00599(7)	0.00600(7)
	U33	0.00413(9)	0.00413(9)	0.00534(11)	0.00417(8)	0.00418(8)
	U12	0.00007(7)	0.00007(7)	0.00001(9)	0.00005(6)	0.00005(6)
	U13	0.00046(7)	0.00045(7)	0.00061(9)	0.00046(6)	0.00047(6)
	U23	-0.00008(7)	-0.00008(7)	0.00000(9)	-0.00008(6)	-0.00007(6)
	S_{12}	0.569%	0.569%	0	0.529%	0.517%
C1	U11	0.00485(8)	0.00486(8)	0.00498(15)	0.00480(7)	0.00481(7)
(C3)	U22	0.00429(7)	0.00428(7)	0.00501(16)	0.00427(7)	0.00427(7)
	U33	0.00328(9)	0.00328(9)	0.00453(13)	0.00324(8)	0.00326(9)
	U12	0.00025(7)	0.00025(7)	0.00036(13)	0.00035(6)	0.00034(7)
	U13	-0.00033(7)	-0.00033(7)	-0.00020(12)	-0.00030(7)	-0.00030(7)
	U23	0.00004(7)	0.00004(7)	-0.00035(12)	0.00001(6)	0.00001(6)
	S_{12}	0.911%	0.915%	0	0.951%	0.924%
C2	U11	0.00505(8)	0.00505(8)	0.00497(16)	0.00500(8)	0.00502(8)
(C1)	U22	0.00459(7)	0.00460(7)	0.00555(18)	0.00459(7)	0.00460(7)
	U33	0.00354(10)	0.00354(10)	0.00456(14)	0.00347(9)	0.00348(9)
	U12	0.00045(7)	0.00044(7)	0.00045(11)	0.00043(6)	0.00043(6)
	U13	-0.00030(7)	-0.00030(7)	-0.00033(11)	-0.00029(7)	-0.00028(7)
	U23	0.00005(7)	0.00005(6)	0.00006(11)	0.00003(6)	0.00003(6)
	S_{12}	0.629%	0.623 %	0	0.691%	0.676%
C3	U11	0.00576(9)	0.00576(9)	0.00584(18)	0.00573(8)	0.00575(8)
(C2)	U22	0.00878(9)	0.00878(9)	0.00937(19)	0.00875(8)	0.00876(8)
	U33	0.00601(11)	0.00601(11)	0.00754(17)	0.00597(10)	0.00599(10)
	U12	-0.00128(8)	-0.00128(8)	-0.00136(13)	-0.00131(7)	-0.00132(7)

(Unit: Å^2)		pHAR	...Fcut3	Neutron	XHARPy	...iovs_cut
	U13	-0.00061(8)	-0.00061(8)	-0.00054(13)	-0.00060(7)	-0.00060(7)
	U23	-0.00038(8)	-0.00038(8)	-0.00050(15)	-0.00041(8)	-0.00041(8)
	S_{12}	0.361%	0.361%	0	0.385%	0.374%
H1	U11	0.022(3)	0.021(3)	0.0198(5)	0.023(3)	0.023(3)
(H4)	U22	0.014(3)	0.014(3)	0.0120(4)	0.014(3)	0.014(3)
	U33	0.019(4)	0.019(4)	0.0192(5)	0.018(4)	0.019(4)
	U12	0.009(3)	0.009(3)	0.0037(4)	0.009(3)	0.009(3)
	U13	0.005(3)	0.005(3)	-0.0006(4)	0.005(3)	0.005(3)
	U23	0.003(3)	0.003(3)	0.0005(4)	0.005(3)	0.005(3)
	S_{12}	2.117%	2.325%	0	2.448%	2.310%
H2	U11	0.018(3)	0.018(3)	0.0211(6)	0.022(4)	0.022(4)
(H1)	U22	0.023(3)	0.023(3)	0.0137(5)	0.023(3)	0.023(4)
	U33	0.013(4)	0.013(4)	0.0243(6)	0.019(5)	0.020(5)
	U12	-0.001(3)	-0.001(3)	0.0057(4)	0.003(3)	0.004(3)
	U13	0.005(3)	0.005(3)	0.0034(5)	0.008(4)	0.008(4)
	U23	-0.004(3)	-0.004(3)	0.0024(5)	-0.000(3)	-0.000(3)
	S_{12}	8.299%	8.299%	0	4.317%	3.852%
H3	U11	0.009(3)	0.009(3)	0.0131(5)	0.012(3)	0.013(3)
(H2)	U22	0.018(3)	0.018(3)	0.0216(6)	0.023(3)	0.023(3)
	U33	0.016(4)	0.016(4)	0.0211(5)	0.021(5)	0.021(5)
	U12	0.002(3)	0.002(3)	-0.0057(4)	0.000(3)	0.000(3)
	U13	0.002(3)	0.002(3)	0.0009(4)	0.007(3)	0.007(4)
	U23	0.002(3)	0.002(3)	-0.0016(5)	0.003(3)	0.003(3)
	S_{12}	4.871%	4.871%	0	4.364%	4.051%
H4	U11	0.023(4)	0.023(4)	0.0203(6)	0.025(4)	0.026(4)
(H3)	U22	0.012(3)	0.012(3)	0.0238(7)	0.019(4)	0.019(4)
	U33	0.004(4)	0.004(4)	0.0113(4)	0.008(4)	0.008(4)
	U12	-0.007(3)	-0.007(3)	0.0002(5)	-0.003(3)	-0.003(4)
	U13	0.004(3)	0.004(3)	-0.0008(4)	0.006(3)	0.006(3)
	U23	-0.003(3)	-0.003(3)	0.0004(4)	-0.005(3)	-0.005(3)
	S_{12}	17.327%	17.327%	0	7.668%	7.608%
H5	U11	0.023(4)	0.023(4)	0.0252(8)	0.022(4)	0.023(4)
(H5)	U22	0.045(4)	0.045(4)	0.0323(9)	0.046(4)	0.046(5)
	U33	0.011(4)	0.011(4)	0.0168(5)	0.011(4)	0.011(4)
	U12	-0.009(3)	-0.009(3)	-0.0055(6)	-0.011(3)	-0.012(3)
	U13	-0.011(3)	-0.011(3)	-0.0085(5)	-0.010(3)	-0.010(3)
	U23	0.008(3)	0.008(3)	0.0031(5)	0.007(3)	0.007(3)
	S_{12}	6.572%	6.572%	0	5.100%	4.712%
H6	U11	0.013(3)	0.013(3)	0.0173(6)	0.015(3)	0.015(3)
(H6)	U22	0.032(4)	0.032(4)	0.0337(9)	0.035(4)	0.036(4)
	U33	0.029(5)	0.029(5)	0.0225(6)	0.031(5)	0.033(5)
	U12	-0.003(3)	-0.003(3)	-0.0030(6)	-0.002(3)	-0.002(3)

(Unit: Å^2)		pHAR	...Fcut3	Neutron	XHARPy	...iovs_cut
	U13	0.008(3)	0.008(3)	0.0076(5)	0.004(4)	0.005(4)
	U23	0.002(3)	0.002(3)	-0.0049(6)	0.003(4)	0.003(4)
	S_{12}	2.121%	2.121%	0	2.409%	2.536%
H7	U11	0.032(4)	0.032(4)	0.0250(7)	0.032(5)	0.033(5)
(H7)	U22	0.013(3)	0.013(3)	0.0155(6)	0.016(3)	0.016(3)
	U33	0.034(5)	0.034(5)	0.0335(9)	0.037(5)	0.038(5)
	U12	-0.005(3)	-0.005(3)	-0.0008(5)	-0.006(3)	-0.006(3)
	U13	0.003(4)	0.003(4)	-0.0020(7)	0.004(4)	0.004(4)
	U23	-0.003(3)	-0.003(3)	-0.0024(6)	-0.003(3)	-0.003(3)
	S_{12}	1.361%	1.361%	0	1.319%	1.409%
Mean $S_{12,\mathbf{H}}$		6.095%	6.125%		3.946%	3.783%

5.2 Magnesium bis(hydrogen maleate) hexahydrate (HMA-Mg) at 12 K

We compared the following CIFs: “HMA-Mg_pHAR.cif" from our supporting materials; CCDC-1538846 by L. A. Malaspina[5] (neutron reference); and the data block “data_HMa-Mg_xharpy_SCAN" from the supplementary materials of Ref. 2.[††] The neutron data are used as reference in both pHAR and XHARPy.

Table S8 provides the correspondence of atoms. The primed atoms($'$) are generated by an inversion operation at the Mg ion. They are not presented explicitly in the neutron and XHARPy CIFs, but are included here for ease of comparison with pHAR CIF.

We provide the symmetry operators for converting coordinates of the unique molecule among the CIFs.

From pHAR to neutron: $x, -y + \frac{1}{2}, z + \frac{1}{2}$.

From pHAR to XHARPy: $x, y, z + 1$.

Table S9 and S10 show the refinement statistics and the bond length analysis. Table S11 shows the ADP overlap, where the ADP tensors are transformed by the symmetry operators to match the orientation of the unique molecule in the neutron CIF.

[††]https://doi.org/10.1107/S2052252522001385/fc5060sup1.cif

Table S8: HMA-Mg atom label conversion table

pHAR	Mg	O5	O6	O7	O8	O9	O10	H4<br>H10	H5<br>H11	H6<br>H12	H7<br>H13	H8<br>H14	H9<br>H15
neutron	Mg1	O7	O7′	O5	O6	O5′	O6′	H5<br>H4	H6<br>H7	H8<br>H4′	H9<br>H5′	H8′<br>H6′	H9′<br>H7′
XHARPy	Mg1	O6′	O6	O5	O7′	O5′	O7	H5<br>H4	H8′<br>H9′	H6′<br>H4′	H7′<br>H5	H6<br>H8	H7<br>H9

The labels not shown here are consistent.

Table S9: HMA-Mg refinement statistics.

	R	wR	ρ_{max}	ρ_{min}	ρ_{rms}
HMA-Mg_pHAR	0.0162	0.0172	0.6486	-0.5646	0.0366
HMa-Mg_xharpy_SCAN	0.0148	0.0284	0.7099	-0.5182	0.0378

Table S10: HMA-Mg bond length analysis. The primed atoms(′) are generated by an inversion operation centered at Mg to ensure comparability.

pHAR (HMA-Mg_pHAR)		Neutron		HMa-Mg_xharpy_SCAN	
Mg-O5	2.06024(8)	Mg1-O7	2.0613(10)	Mg1-O6′	2.0600(7)
Mg-O6	2.06024(8)	Mg1-O7′	2.0613(10)	Mg1-O6	2.0600(7)
Mg-O7	2.04292(8)	Mg1-O5	2.0435(7)	Mg1-O5	2.0429(4)
Mg-O8	2.07531(9)	Mg1-O6	2.0776(9)	Mg1-O7	2.0752(5)
Mg-O9	2.04292(8)	Mg1-O5′	2.0435(7)	Mg1-O5′	2.0429(4)
Mg-O10	2.07531(9)	Mg1-O6′	2.0776(9)	Mg1-O7′	2.0752(5)
O1-C1	1.27929(11)	O1-C1	1.2820(8)	O1-C1	1.27974(20)
O2-C4	1.28701(11)	C4-O2	1.2883(8)	O2-C4	1.2873(2)
O3-C1	1.24758(12)	O3-C1	1.2466(8)	O3-C1	1.2484(3)
O4-C4	1.23583(12)	C4-O4	1.2354(9)	O4-C4	1.2366(3)
O1-H1	1.194(4)	O1-H1	1.1874(16)	O1-H1	1.191(4)
O2-H1	1.213(4)	O2-H1	1.2180(16)	O2-H1	1.214(4)
O5-H6	0.915(3)	O7-H8	0.9568(15)	O6′-H6′	0.953(3)
O5-H7	0.928(3)	O7-H9	0.9710(15)	O6′-H7′	0.961(3)
O6-H8	0.915(3)	O7′-H8′	0.9568(15)	O6-H6	0.953(3)
O6-H9	0.928(3)	O7′-H9′	0.9710(15)	O6-H7	0.961(3)
O7-H4	0.925(3)	O5-H5	0.9677(15)	O5-H5	0.960(3)
O7-H10	0.925(3)	O5-H4	0.9734(15)	O5-H4	0.963(3)
O8-H5	0.926(3)	O6-H6	0.9625(15)	O7′-H8′	0.962(3)
O8-H11	0.937(3)	O6-H7	0.9743(14)	O7′-H9′	0.969(3)
O9-H12	0.925(3)	O5′-H4′	0.9734(15)	O5′-H4′	0.963(3)
O9-H13	0.925(3)	O5′-H5′	0.9677(15)	O5′-H5′	0.960(3)
O10-H14	0.926(3)	O6′-H6′	0.9625(15)	O7-H8	0.962(3)
O10-H15	0.937(3)	O6′-H7′	0.9743(14)	O7-H9	0.969(3)
C1-C2	1.49733(13)	C2-C1	1.4957(7)	C1-C2	1.4971(2)
C2-C3	1.34713(14)	C2-C3	1.3478(8)	C2-C3	1.3474(3)
C3-C4	1.50049(13)	C3-C4	1.5003(8)	C3-C4	1.5003(3)
C2-H2	1.076(2)	C2-H2	1.0897(14)	C2-H2	1.091(2)
C3-H3	1.069(3)	C3-H3	1.0910(14)	C3-H3	1.082(2)
$\sum \lvert\Delta r\rvert_{\text{all}}$	0.5625			$\sum \lvert\Delta r\rvert_{\text{all}}$	0.1100
$\sum \lvert\Delta r\rvert_{\textbf{X-H}}$	0.5467			$\sum \lvert\Delta r\rvert_{\textbf{X-H}}$	0.0933

(Unit: Å)

Table S11: The ADPs and the ADP overlap S_{12} of HMA-Mg. The "as CIF" columns list the values reported in the CIFs, while the "sym. operated" columns correspond to the symmetry-transformed ADP tensor obtained using the symmetry operator that maps the atomic coordinates of one atom onto the other in each CIF. The atom labels in brackets refer to those from the neutron CIF and the XHARPy CIF. (See Table S8)

(Unit: Å^2)		pHAR		Neutron	XHARPy	
		as CIF	sym. operated	as CIF	sym. operated	as CIF
Mg	U11	0.003938(18)	0.003938(18)	0.0044(3)	0.004132(15)	0.004132(15)
(Mg1)	U22	0.004772(15)	0.004772(15)	0.0039(3)	0.004960(12)	0.004960(12)
(Mg1)	U33	0.006694(18)	0.006694(18)	0.0046(3)	0.006866(14)	0.006866(14)
	U12	-0.000144(12)	0.000144(12)	0.0001(2)	0.000136(9)	-0.000136(9)
	U13	0.001495(13)	0.001495(13)	0.0007(3)	0.001541(10)	0.001541(10)
	U23	-0.000379(13)	0.000379(13)	0.0002(2)	0.000375(10)	-0.000375(10)
	S_{12}		1.394%	0	1.529%	
O1	U11	0.00505(2)	0.00505(2)	0.0047(2)	0.005127(18)	0.005127(18)
	U22	0.005312(18)	0.005312(18)	0.00413(18)	0.005326(14)	0.005326(14)
	U33	0.01326(3)	0.01326(3)	0.0112(2)	0.01333(2)	0.01333(2)
	U12	-0.000280(15)	0.000280(15)	0.00063(18)	0.000351(12)	-0.000351(12)
	U13	0.001992(18)	0.001992(18)	0.00177(17)	0.001996(15)	0.001996(15)
	U23	0.000070(18)	-0.000070(18)	-0.00008(16)	-0.000069(14)	0.000069(14)
	S_{12}		0.755%	0	0.761%	
O2	U11	0.00497(2)	0.00497(2)	0.0041(2)	0.005053(18)	0.005053(18)
	U22	0.005888(19)	0.005888(19)	0.00451(19)	0.005838(15)	0.005838(15)
	U33	0.01552(3)	0.01552(3)	0.0124(2)	0.01563(2)	0.01563(2)
	U12	-0.000506(16)	0.000506(16)	0.00097(17)	0.000494(13)	-0.000494(13)
	U13	0.001975(19)	0.001975(19)	0.00109(17)	0.001967(16)	0.001967(16)
	U23	-0.000005(19)	0.000005(19)	-0.00006(15)	0.000006(15)	-0.000006(15)
	S_{12}		1.338%	0	1.378%	
O3	U11	0.00448(2)	0.00448(2)	0.0035(2)	0.004463(18)	0.004463(18)
	U22	0.00702(2)	0.00702(2)	0.00561(19)	0.007086(16)	0.007086(16)
	U33	0.01302(3)	0.01302(3)	0.0104(2)	0.01305(2)	0.01305(2)
	U12	-0.000299(16)	0.000299(16)	0.00011(18)	0.000303(13)	-0.000303(13)
	U13	0.001830(18)	0.001830(18)	0.00130(16)	0.001807(15)	0.001807(15)
	U23	0.000174(18)	-0.000174(18)	0.00006(15)	-0.000181(15)	0.000181(15)
	S_{12}		0.993%	0	1.018%	
O4	U11	0.00504(2)	0.00504(2)	0.0046(2)	0.004998(18)	0.004998(18)
	U22	0.00775(2)	0.00775(2)	0.00594(19)	0.007819(17)	0.007819(17)
	U33	0.01308(3)	0.01308(3)	0.0104(2)	0.01307(2)	0.01307(2)
	U12	0.000868(17)	-0.000868(17)	-0.00095(18)	-0.000934(13)	0.000934(13)
	U13	0.001326(19)	0.001326(19)	0.00057(17)	0.001288(15)	0.001288(15)
	U23	-0.000821(19)	0.000821(19)	0.00084(16)	0.000822(15)	-0.000822(15)
	S_{12}		0.931%	0	0.931%	

(Unit: Å^2)		pHAR		Neutron	XHARPy	
		as CIF	sym. operated	Neutron	sym. operated	as CIF
O5,O6	U11	0.00971(3)	0.00971(3)	0.0085(3)	0.00975(2)	0.00975(2)
(O7,O7′)	U22	0.01210(3)	0.01210(3)	0.0091(2)	0.01214(2)	0.01214(2)
(O6′,O6)	U33	0.01132(3)	0.01132(3)	0.0081(2)	0.01132(2)	0.01132(2)
	U12	0.00408(2)	-0.00408(2)	-0.0031(2)	-0.004098(17)	0.004098(17)
	U13	-0.00266(2)	-0.00266(2)	-0.00176(19)	-0.002701(17)	-0.002701(17)
	U23	-0.00429(2)	0.00429(2)	0.00287(16)	0.004323(17)	-0.004323(17)
	S_{12}		1.191%	0	1.203%	
O7,O9	U11	0.00651(3)	0.00651(3)	0.0063(2)	0.006498(20)	0.006498(20)
(O5,O5′)	U22	0.005613(19)	0.005613(19)	0.0048(2)	0.005626(15)	0.005626(15)
(O5,O5′)	U33	0.01476(3)	0.01476(3)	0.0108(2)	0.01482(2)	0.01482(2)
	U12	-0.000260(16)	0.000260(16)	-0.00010(18)	0.000269(13)	-0.000269(13)
	U13	0.00134(2)	0.00134(2)	0.00086(18)	0.001327(16)	0.001327(16)
	U23	0.000255(19)	-0.000255(19)	-0.00051(15)	-0.000265(15)	0.000265(15)
	S_{12}		0.838%	0	0.859%	
O8,O10	U11	0.01068(3)	0.01068(3)	0.0079(2)	0.01077(2)	0.01077(2)
(O6,O6′)	U22	0.00838(2)	0.00838(2)	0.0068(2)	0.008399(18)	0.008399(18)
(O7′,O7)	U33	0.01390(3)	0.01390(3)	0.0096(2)	0.01396(2)	0.01396(2)
	U12	0.001631(18)	-0.001631(18)	-0.00100(17)	-0.001664(15)	0.001664(15)
	U13	0.00776(2)	0.00776(2)	0.00512(19)	0.007832(19)	0.007832(19)
	U23	0.00156(2)	-0.00156(2)	-0.00114(15)	-0.001583(16)	0.001583(16)
	S_{12}		1.393%	0	1.432%	
C1	U11	0.00437(3)	0.00437(3)	0.00316(18)	0.00424(2)	0.00424(2)
	U22	0.00552(2)	0.00552(2)	0.00418(16)	0.005375(18)	0.005375(18)
	U33	0.00748(3)	0.00748(3)	0.00571(17)	0.00733(2)	0.00733(2)
	U12	-0.000119(18)	0.000119(18)	0.00007(15)	0.000138(15)	-0.000138(15)
	U13	0.001423(19)	0.001423(19)	0.00040(13)	0.001402(15)	0.001402(15)
	U23	0.000075(19)	-0.000075(19)	-0.00034(12)	-0.000078(15)	0.000078(15)
	S_{12}		1.745%	0	1.502%	
C2	U11	0.00504(3)	0.00504(3)	0.00435(18)	0.00492(2)	0.00492(2)
	U22	0.00532(2)	0.00532(2)	0.00433(16)	0.005196(18)	0.005196(18)
	U33	0.00942(3)	0.00942(3)	0.00737(18)	0.00928(2)	0.00928(2)
	U12	-0.000332(19)	0.000332(19)	0.00006(16)	0.000327(15)	-0.000327(15)
	U13	0.00156(2)	0.00156(2)	0.00096(14)	0.001544(17)	0.001544(17)
	U23	-0.00005(2)	0.00005(2)	0.00012(13)	0.000021(16)	-0.000021(16)
	S_{12}		0.793%	0	0.665%	
C3	U11	0.00524(3)	0.00524(3)	0.00481(18)	0.00508(2)	0.00508(2)
	U22	0.00531(2)	0.00531(2)	0.00397(16)	0.005190(18)	0.005190(18)
	U33	0.00982(3)	0.00982(3)	0.00776(18)	0.00968(2)	0.00968(2)
	U12	0.000028(19)	-0.000028(19)	-0.00019(15)	-0.000022(15)	0.000022(15)
	U13	0.00161(2)	0.00161(2)	0.00073(14)	0.001566(17)	0.001566(17)
	U23	-0.00004(2)	0.00004(2)	-0.00017(13)	0.000030(16)	-0.000030(16)
	S_{12}		1.029%	0	0.891%	

(Unit: Å^2)		pHAR		Neutron	XHARPy	
		as CIF	sym. operated	Neutron	sym. operated	as CIF
C4	U11	0.00447(3)	0.00447(3)	0.0044(2)	0.00430(2)	0.00430(2)
	U22	0.00612(2)	0.00612(2)	0.00447(17)	0.005970(18)	0.005970(18)
	U33	0.00768(3)	0.00768(3)	0.00552(18)	0.00755(2)	0.00755(2)
	U12	0.000113(19)	-0.000113(19)	0.00004(14)	-0.000115(15)	0.000115(15)
	U13	0.00169(2)	0.00169(2)	0.00114(15)	0.001632(16)	0.001632(16)
	U23	-0.00030(2)	0.00030(2)	0.00020(11)	0.000281(16)	-0.000281(16)
	S_{12}		1.302%	0	1.159%	
H1	U11	0.047(3)	0.047(3)	0.0200(6)	0.047(3)	0.047(3)
	U22	0.0097(17)	0.0097(17)	0.0139(4)	0.0144(17)	0.0144(17)
	U33	0.017(2)	0.017(2)	0.0226(5)	0.0215(20)	0.0215(20)
	U12	-0.0059(19)	0.0059(19)	0.0034(4)	0.0055(18)	-0.0055(18)
	U13	0.011(2)	0.011(2)	0.0051(4)	0.0113(19)	0.0113(19)
	U23	-0.0030(16)	0.0030(16)	0.0007(4)	0.0031(15)	-0.0031(15)
	S_{12}		6.575%	0	4.687%	
H2	U11	0.0140(14)	0.0140(14)	0.0152(5)	0.0151(13)	0.0151(13)
	U22	0.0143(13)	0.0143(13)	0.0125(4)	0.0149(12)	0.0149(12)
	U33	0.0340(17)	0.0340(17)	0.0344(7)	0.0356(16)	0.0356(16)
	U12	-0.0058(11)	0.0058(11)	0.0060(4)	0.0064(9)	-0.0064(9)
	U13	0.0019(12)	0.0019(12)	0.0036(5)	0.0023(11)	0.0023(11)
	U23	-0.0049(12)	0.0049(12)	0.0011(4)	0.0044(11)	-0.0044(11)
	S_{12}		0.862%	0	0.669%	
H3	U11	0.0168(14)	0.0168(14)	0.0179(6)	0.0218(14)	0.0218(14)
	U22	0.0131(13)	0.0131(13)	0.0100(4)	0.0126(12)	0.0126(12)
	U33	0.0377(18)	0.0377(18)	0.0363(7)	0.0386(17)	0.0386(17)
	U12	0.0055(11)	-0.0055(11)	-0.0020(4)	-0.0046(10)	0.0046(10)
	U13	0.0027(13)	0.0027(13)	0.0044(5)	0.0049(12)	0.0049(12)
	U23	-0.0010(12)	0.0010(12)	0.0003(4)	-0.0012(11)	0.0012(11)
	S_{12}		1.158%	0	0.715%	
H4,H13	U11	0.0126(15)	0.0126(15)	0.0169(6)	0.0153(15)	0.0153(15)
(H5,H5′)	U22	0.0208(15)	0.0208(15)	0.0171(5)	0.0222(15)	0.0222(15)
(H5,H5′)	U33	0.032(2)	0.032(2)	0.0318(7)	0.038(2)	0.038(2)
	U12	-0.0064(12)	0.0064(12)	0.0055(5)	0.0080(12)	-0.0080(12)
	U13	0.0033(13)	0.0033(13)	0.0010(5)	0.0059(14)	0.0059(14)
	U23	0.0013(14)	-0.0013(14)	0.0003(5)	-0.0003(15)	0.0003(15)
	S_{12}		1.474%	0	1.665%	
H5,H14	U11	0.0281(17)	0.0281(17)	0.0298(7)	0.0306(19)	0.0306(19)
(H6,H6′)	U22	0.0189(15)	0.0189(15)	0.0179(5)	0.0231(15)	0.0231(15)
(H8′,H8)	U33	0.028(2)	0.028(2)	0.0229(6)	0.0290(18)	0.0290(18)
	U12	-0.0003(13)	0.0003(13)	-0.0005(5)	-0.0005(14)	0.0005(14)
	U13	0.0182(14)	0.0182(14)	0.0106(5)	0.0169(14)	0.0169(14)
	U23	0.0053(15)	-0.0053(15)	-0.0069(4)	-0.0083(14)	0.0083(14)
	S_{12}		2.289%	0	1.281%	

(Unit: Å^2)		pHAR as CIF	pHAR sym. operated	Neutron Neutron	XHARPy sym. operated	XHARPy as CIF
H6,H8	U11	0.0215(17)	0.0215(17)	0.0274(7)	0.034(2)	0.034(2)
(H8,H8′)	U22	0.0262(17)	0.0262(17)	0.0241(6)	0.0303(18)	0.0303(18)
(H6′,H6)	U33	0.0244(17)	0.0244(17)	0.0190(5)	0.0253(17)	0.0253(17)
	U12	-0.0010(13)	0.0010(13)	-0.0015(5)	-0.0015(15)	0.0015(15)
	U13	0.0037(13)	0.0037(13)	0.0020(5)	0.0031(14)	0.0031(14)
	U23	-0.0133(14)	0.0133(14)	0.0099(4)	0.0144(15)	-0.0144(15)
	S_{12}		0.878%	0	0.969%	
H7,H9	U11	0.0223(17)	0.0223(17)	0.0177(6)	0.0306(19)	0.0306(19)
(H9,H9′)	U22	0.0196(16)	0.0196(16)	0.0223(5)	0.0231(15)	0.0231(15)
(H7′,H7)	U33	0.0256(18)	0.0256(18)	0.0275(6)	0.0290(18)	0.0290(18)
	U12	0.0105(14)	-0.0105(14)	-0.0099(5)	-0.0005(14)	0.0005(14)
	U13	0.0020(13)	0.0020(13)	0.0006(5)	0.0169(14)	0.0169(14)
	U23	-0.0001(14)	0.0001(14)	0.0013(5)	-0.0083(14)	0.0083(14)
	S_{12}		0.628%	0	0.849%	
H10,H12	U11	0.0143(16)	0.0143(16)	0.0161(6)	0.0161(15)	0.0161(15)
(H4,H4′)	U22	0.0193(16)	0.0193(16)	0.0176(5)	0.0240(16)	0.0240(16)
(H4,H4′)	U33	0.0297(18)	0.0297(18)	0.0292(6)	0.0315(19)	0.0315(19)
	U12	0.0019(12)	-0.0019(12)	-0.0039(5)	-0.0055(12)	0.0055(12)
	U13	-0.0001(13)	-0.0001(13)	-0.0014(5)	0.0006(13)	0.0006(13)
	U23	-0.0003(14)	0.0003(14)	-0.0043(4)	0.0007(15)	-0.0007(15)
	S_{12}		1.067%	0	1.586%	
H11,H15	U11	0.0173(15)	0.0173(15)	0.0249(6)	0.0209(17)	0.0209(17)
(H7,H7′)	U22	0.0183(14)	0.0183(14)	0.0156(5)	0.0224(15)	0.0224(15)
(H9′,H9)	U33	0.0273(18)	0.0273(18)	0.0266(6)	0.0292(18)	0.0292(18)
	U12	0.0006(12)	-0.0006(12)	-0.0041(5)	-0.0011(13)	0.0011(13)
	U13	0.0094(13)	0.0094(13)	0.0110(5)	0.0104(13)	0.0104(13)
	U23	0.0004(14)	-0.0004(14)	0.0030(4)	0.0043(14)	-0.0043(14)
	S_{12}		2.468%	0	1.608%	
Mean $S_{12,\mathbf{H}}$			1.747%		1.466%	

6. Residual electron density analysis

6.1 Examined compounds

The cases in which pHAR yielded residual electron densities $\Delta\rho_{\max} > 0.5$ eÅ^{-3} are examined in detail and compared with the results refined with the multipole model (MM). Besides residual-density values, we also compare the atomic charges and dipole moments obtained from HAR and MM. In the current version of Tonto, Hirshfeld atom analysis is available only for conventional HAR, not pHAR. Since the HAR and pHAR results are qualitatively identical and quantitatively similar, we compare HAR with MM instead of pHAR and MM. For HAR, the total charge of a Hirshfeld atom is given by the nuclear charge minus the integral of its partitioned electron density. For MM, we used the nuclear charge minus the sum of core electrons and valence electrons. Note that the number of valence electrons is a refinable parameter and can be found in the following CIF field: **_atom_rho_multipole_Pv**. The refined dipole moment parameters are found in **_atom_rho_multipole_P11**, ...**_P1-1**, and ...**_P10**, which correspond to the negative x-, y-, and z-dipole components, respectively. The dipole moment μ is calculated by the following formula.[6]

$$\mu_x = -\frac{20}{3\kappa'\zeta}P_{11+} \quad \mu_y = -\frac{20}{3\kappa'\zeta}P_{11-} \quad \mu_z = -\frac{20}{3\kappa'\zeta}P_{10} \tag{11}$$

The units of residual electron density (eÅ^{-3}), dipole moment (Debye), and charge (e) are used throughout. The direction of the dipole moment follows the convention from positive to negative charge. When comparing HAR with MM, we have to be careful when discussing absolute values because different numbers of reflections were used in the published multipole models and our new HAR refinements. All refinements are based on merged data.

6.1.1 pHAR of ammonia trifluoroborane

Ammonia trifluoroborane refined with pHAR yields $\Delta\rho_{\max}$= 0.58, whereas the multipole model (MM) refinement yields $\Delta\rho_{\max}$= 0.29[7] (see jp100995n_si_001.cif). As shown in Figure

S6, significant residual electron density remains near the F atoms, which is also observed in the IAM result (inset in Figure S6 (b)). This indicates that the electron density in the vicinity of the F atoms predicted by the *ab initio* calculation is underestimated relative to the experimental measurement. Apart from these regions, $|\Delta\rho| < 0.3$. More specifically, the next highest maximum in $\Delta\rho$ is 0.24 near the N atoms.

In (Table S12), the projected dipole moments of each Hirshfeld atom onto its local z-axis are shown, while the dipoles are aligned up with the local z-axes within 3.2 degrees. For this compound, only ..._**P10** parameters in MM are non-zero and shown in the table.

6.1.2 pHAR of hydrazine borane

Hydrazine borane refined with pHAR yields $\Delta\rho_{\mathrm{max}}= 0.54$, whereas the MM refinement yields $\Delta\rho_{\mathrm{max}}= 0.24$[7] (see jp100995n_si_002.cif). As shown in Figure S7, significant residual electron density remains near the N atoms along the N–N bond axis. This tendency is also observed in the IAM result (inset in (b)). Apart from these regions, $|\Delta\rho| < 0.3$, and the next highest maximum in $\Delta\rho$ is 0.26 near the B atoms.

Table S13 shows the charges and projected dipole moments of each pseudo-atom in the same manner as in the previous subsection. The local axes are defined according to the MM CIF. For the N atoms, dummy atoms (DUM1 and DUM2) are introduced to define the local xy-plane; DUM1 and DUM2 are located at the midpoints between H1 and H2, and between

Table S12: Charges and dipole moments of pseudo-atoms from HAR refinement and multipole refinement for *ammonia trifluoroborane*. The dipole moments of the MM atoms are calculated from the P10 parameters.

Label HAR	HA charge	z-dipole	Label MM	MM charge	z-dipole	Local z-axis
B	0.273	-0.156	B(1)	0.20(3)	-0.49(8)	B–N
F1	-0.257	-0.469	F(1)	-0.273(11)	-0.07(1)	F1–B
F2	-0.258	-0.468	F(2)	-0.278(11)	-0.07(1)	F2–B
F3	-0.255	-0.466	F(3)	-0.288(11)	-0.07(1)	F3–B
N	-0.002	0.064	N(1)	-0.12(3)	0.19(3)	N–B
H1	0.170	0.446	H(1)	0.246(13)	1.13(5)	H1–N
H2	0.163	0.446	H(2)	0.323(11)	1.08(5)	H2–N
H3	0.165	0.446	H(3)	0.196(13)	1.34(5)	H3–N

Table S13: Charges and projected dipoles of pseudo-atoms from HAR refinement and multipole refinement for *hydrazine borane*. The dipole moments of the MM atoms are calculated from the P11, P1-1, and P10 parameters.

Label HAR	HA charge	dipole	Label MM	MM charge	dipole	Local axes
N1	0.059	x: -0.113	N(1)	0.01(3)	x: -0.29(2)	x: N1–N2
		y: -0.057			y: -0.35(2)	y: N1–DUM1
N2	-0.132	x: -0.396	N(2)	0.08(2)	x: -0.37(2)	x: N2–N1
		y: -0.562			y: -0.79(3)	y: N2–DUM2
B	-0.050	z: -0.297	B(1)	0.30(4)	z: -0.11(8)	z: B–N1
H1	0.148	z: 0.436	H(4)	0.011(10)	z: 1.48(5)	z: H1–N1
H2	0.138	z: 0.414	H(5)	0.002(10)	z: 1.57(5)	z: H2–N1
H3	0.099	z: 0.419	H(6)	-0.058(13)	z: 1.83(6)	z: H3–N2
H4	0.110	z: 0.410	H(7)	0.045(11)	z: 1.80(5)	z: H4–N2
H5	-0.126	z: 0.049	H(1)	-0.171(12)	z: 1.26(8)	z: H5–B
H6	-0.121	z: 0.074	H(3)	-0.065(11)	z: 1.26(7)	z: H6–B
H7	-0.126	z: 0.047	H(2)	-0.160(12)	z: 1.19(7)	z: H7–B

H3 and H4, respectively. The dipoles of each Hirshfeld atom are projected onto its local axes. The angles between the dipoles of N atoms and the local xy-planes are less than 1.8 degrees. The dipole moments of the B atom and the H atoms bonded to nitrogen (H1, H2, H3, and H4) align with the local z-axis within 7.5 degrees. However, the dipoles of the H atoms bonded to boron (H5, H6, and H7) do not align well with the local z-axes; the angles between them are 40, 32, and 45 degrees, respectively.

6.1.3 pHAR of bis(2,2′-bipyridinium) closo-decaborate(10) hydrate

Bis(2,2′-bipyridinium) closo-decaborate(10) hydrate refined with pHAR yields $\Delta\rho_{\mathrm{max}} = 0.63$, whereas the MM refinement yields $\Delta\rho_{\mathrm{max}} = 0.38$.[8] Figure S8 shows the residual electron density maps. Significant residual electron density remains mostly on the bipyridinium molecule labeled ‘b’ (Figure S8(e)). In addition, elongated regions approximately 3.7 Å wide along the a-axis with $0.3 < \Delta\rho < 0.35$ are observed between two bipyridinium molecules labeled ‘b’; these regions are located near each corner of the unit cell (Figure S8(a)).

6.1.4 pHAR of bis(2,6-lutidinium) closo-dodecaborate(12)

Bis(2,6-lutidinium) closo-dodecaborate(12) refined with pHAR yields $\Delta\rho_{\text{max}}$= 0.80. The MM refinement also reports a singular positive residual electron density of $\Delta\rho_{\text{max}}$= 0.68 located between the molecules.[8] Figure S9(b) clearly shows this feature. Apart from this region and $\Delta\rho_{\text{min}}$ near C7, $|\Delta\rho| < 0.3$. The next highest maximum in $\Delta\rho$ is 0.25 near the N atoms.

6.2 Discussion of residual electron density

In the MM refinement, the number of valence electrons for each atom is adjusted to the experimental structure factors, providing the model with the flexibility to account for charge transfer between atoms. In contrast, in HAR and pHAR, the electron density is determined by the Hamiltonian and the basis functions; thus, the magnitude of charge transfer and the corresponding charge of each atom are not adjusted to the experimental data. Consequently, pHAR may yield larger residual electron densities near atoms than MM does. This is observed in the cases of *ammonia trifluoroborane* and *hydrazine borane.* In other words, in Table S12 and S13, the HA charges and dipoles are not refinable, although their values may differ depending on the partitioning scheme, but all the MM charges and dipoles are refined.

In *ammonia trifluoroborane*, the F atoms, which are most affected by residual density features, show a smaller charge for HAR than for MM in Table S12, but a much larger magnitude of the dipole moment. In contrast, the affected N atoms in *hydrazine borane* show much more extreme Hirshfeld charges in Table S13 than in MM, but a smaller difference in the dipole moment projections. This means that there are significant differences in charges and dipole moments between the models. This reflects on the distribution of the valence electron density which, in turn, could cause differences in residual-density features. However, there is no correlation or clear causal relationship between them. It will be very interesting to see how the HAR model will change when the wavefunction parameters are allowed to adjust to the experimental diffraction data, as it is done in X-ray constrained wavefunction (XCW) fitting.[9] We will pursue this idea in a follow-up study once the periodic version of

XCW fitting will be available, which is being developed by some of us at present.

Chemical disorder in the samples may also lead to large residual electron densities. In HAR and pHAR, each site is assumed to be fully occupied by a single atom. Any disorder not accounted for in the model manifests as residual electron density. Unlike MM, which can absorb the effects of disorder by adjusting many model parameters and thereby reducing the residual electron density, HAR and pHAR reveal the presence of disorder by exposing unmodeled electron density. In the case of *bis(2,2′-bipyridinium) closo-decaborate(10) hydrate*, Figure S8 reveals positional disorder of one of the bipyridine groups, but not the other. The non-disordered one is presumably fixated by a hydrogen bond in the crystal packing which the disordered one does not possess. This minor disorder was revealed by using pHAR, but not treated, because disoreder modelling is presently not possible in the software Tonto. In *bis(2,6-lutidinium) closo-dodecaborate(12)*, there is substitutional disorder which causes the large residual density of 0.80. In agreement with reference 8, we hypothesize that this could be a halogen atom with a very low occupancy that was used during the synthesis.

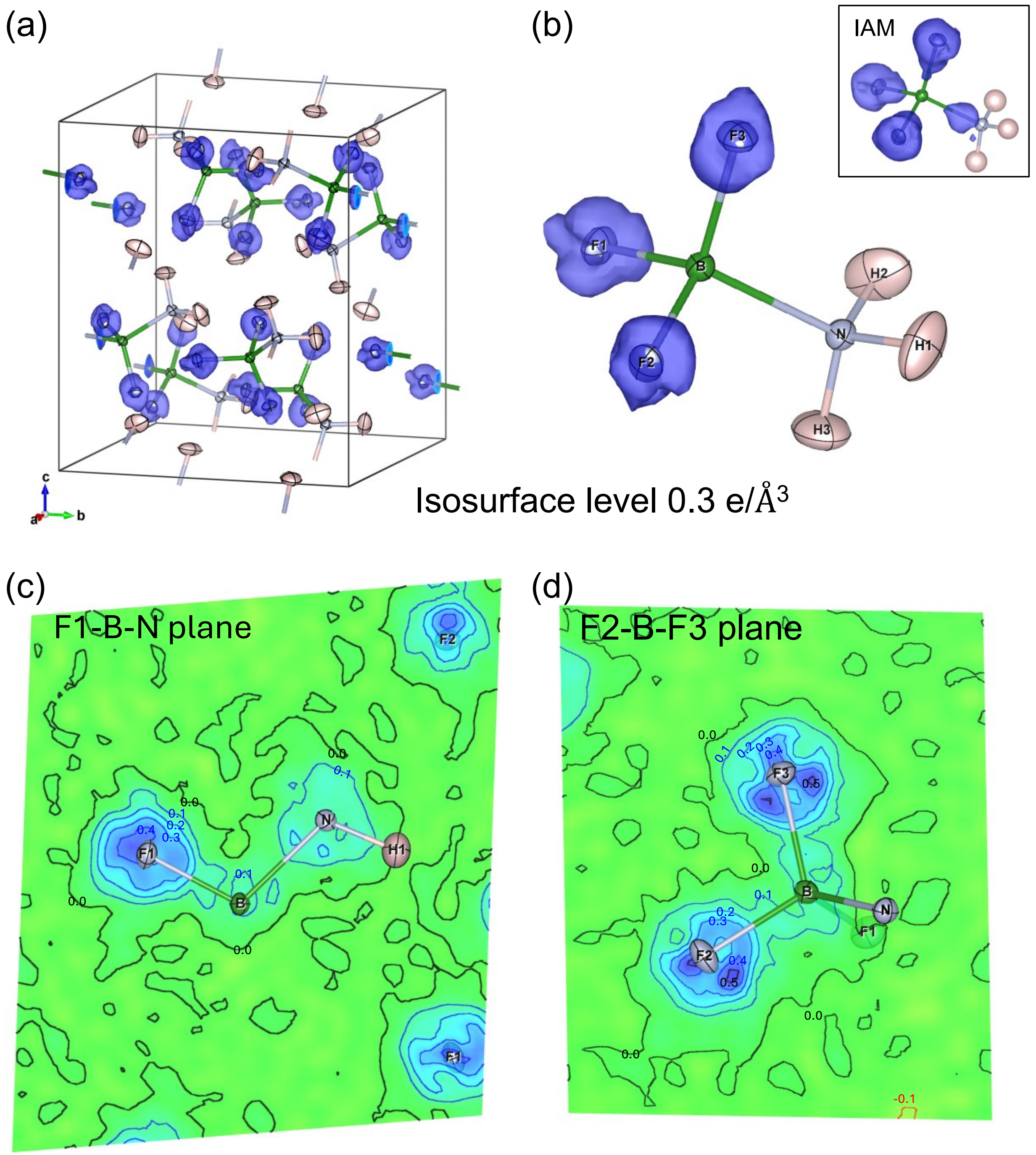

Figure S6: Residual electron density of ammonia trifluoroborane refined with pHAR: (a) unit cell, (b) single molecule, (c) F1-B-N plane, and (d) F2-B-F3 plane. The isosurface level is 0.3 e/Å^3, and the contour interval is 0.1 e/Å^3. Inset in (b) shows the IAM refinement.

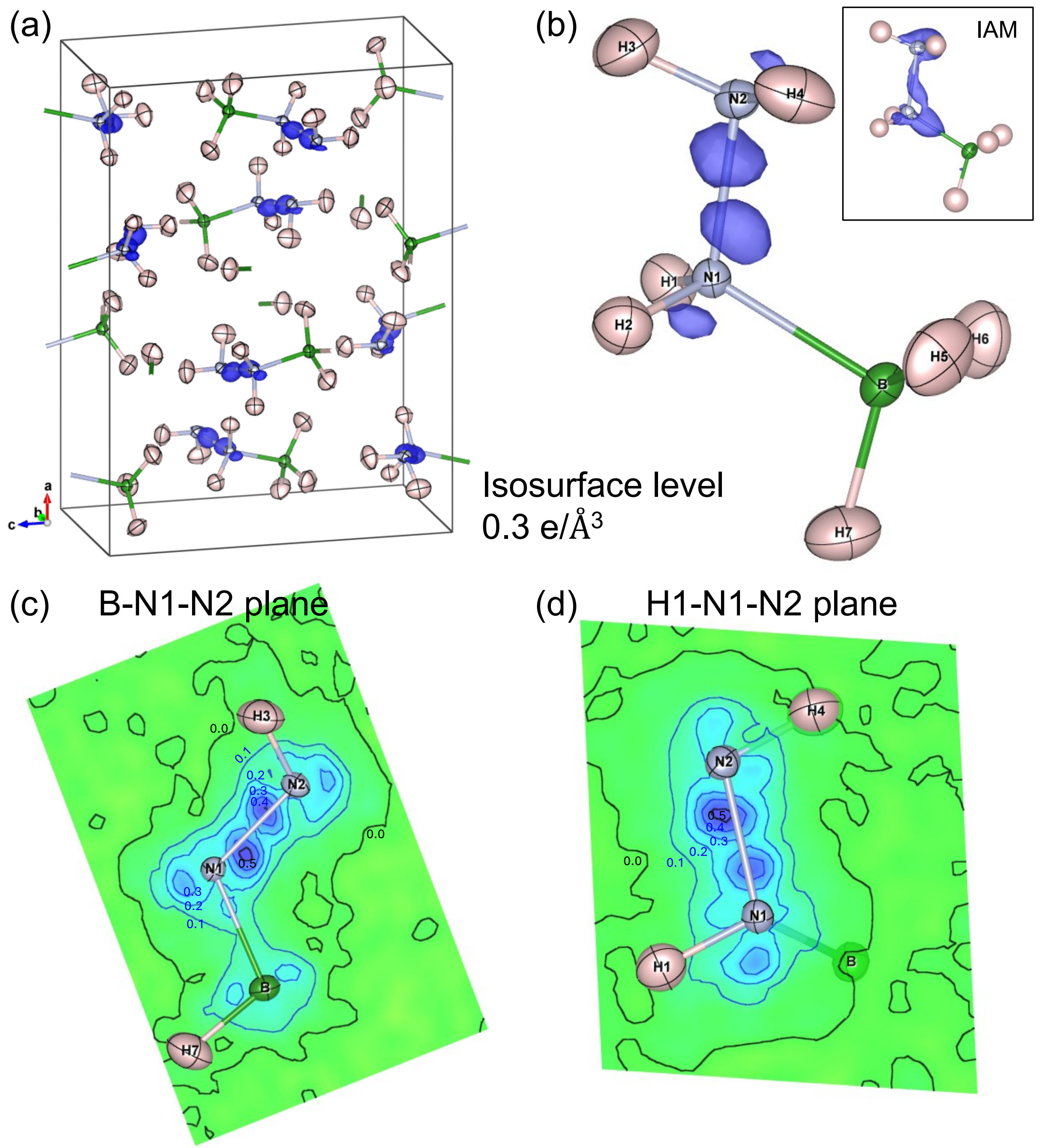

Figure S7: Residual electron density of hydrazine borane refined with pHAR: (a) unit cell, (b) single molecule, (c) N1-N2-H3 plane, and (d) H1-N1-N2 plane. The isosurface level is 0.3 e/Å^3, and the contour interval is 0.1 e/Å^3. Inset in (b) shows the IAM refinement.

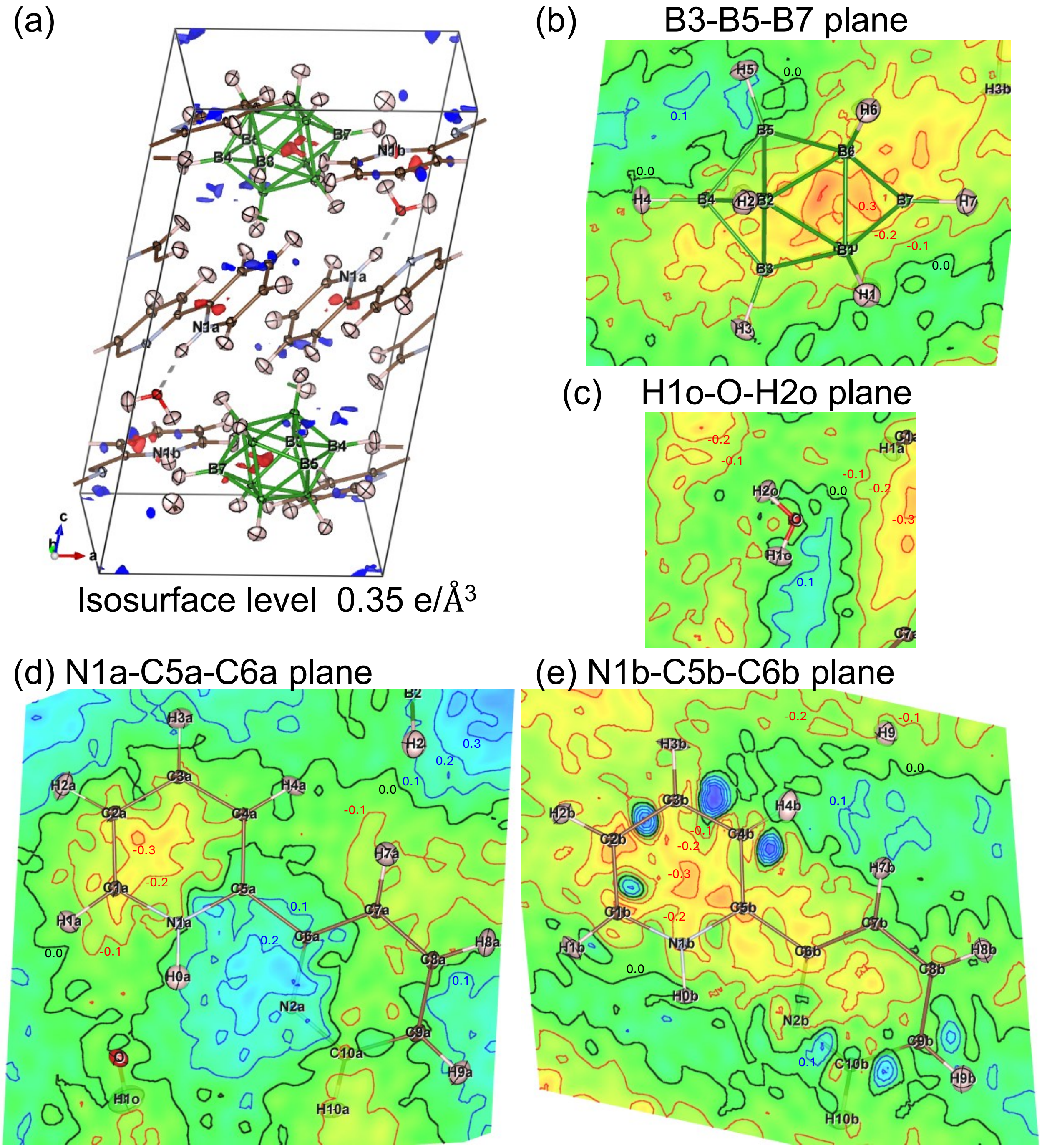

Figure S8: Residual electron density of Bis(2,2′-bipyridinium) closo-decaborate(10) hydrate refined with pHAR: (a) unit cell, (b) closo-decaborane(10), B3-B5-B7 plane (c) water, (d) bipyridinium, N1a-C5a-C6a plane, and (e) bipyridinium, N1b-C5b-C6b plane. The isosurface level is 0.35 e/Å^3, and the contour interval is 0.1 e/Å^3.

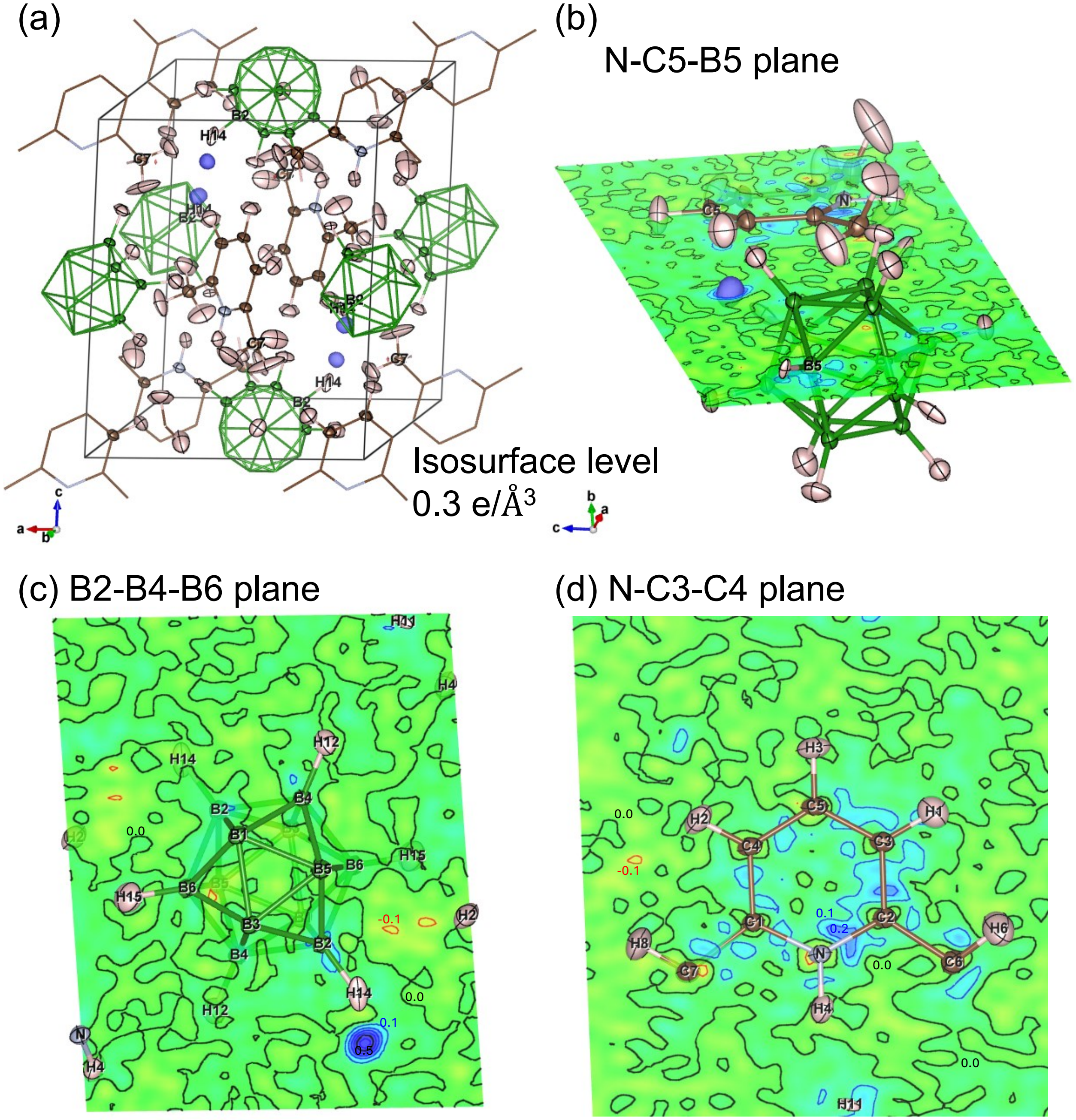

Figure S9: Residual electron density of bis(2,6-lutidinium) closo-dodecaborate(12) refined with pHAR: (a) unit cell, (b) singular positive residual electron density located between the lutidinium and the dodecaborate molecules (c) closo-dodecaborate(12), B2-B4-B6 plane, and (d) 2,6-lutidinium, N-C3-C4 plane. The isosurface level is 0.3 e/Å^3, and the contour interval is 0.1 e/Å^3.

7. Tabulated numerical data for Figure 4

HAR and pHAR were performed using the pob-TZVP-rev2 basis set and the B3LYP hybrid functional. All bond lengths in the molecules are reported, including the symmetry-equivalent bonds.

(a) Ammonia, NH_3 (Unit:Å)

	IAM	HAR	pHAR
N-H1	0.825(8)	0.933(6)	0.947(4)
N-H2	0.825(8)	0.933(6)	0.947(4)
N-H3	0.825(8)	0.933(6)	0.947(4)

Molecular symmetry in crystal: **3**

(b) Diborane, B_2H_6

	IAM	HAR	pHAR
B1-H1*	1.244(6)	1.286(6)	1.289(5)
B1-H2	1.034(8)	1.164(7)	1.159(6)
B1-H3*	1.238(7)	1.288(6)	1.291(6)
B1-H4	1.058(7)	1.162(6)	1.159(6)
B2-H1*	1.238(7)	1.288(6)	1.291(6)
B2-H3*	1.244(6)	1.286(6)	1.289(5)
B2-H5	1.034(8)	1.164(7)	1.159(6)
B2-H6	1.058(7)	1.162(6)	1.159(6)

Mol. sym. in cry.: **-1** * B-H-B bridges

(c) Ammonia Trifluoroborane, NH_3BF_3

	IAM	HAR	pHAR
N-H1	0.792(6)	1.009(5)	1.009(5)
N-H2	0.876(7)	0.977(5)	0.984(4)
N-H3	0.870(7)	0.981(5)	0.985(4)

Mol. sym. in cry.: **1**

(d) Hydrazine borane, $N_2H_4BH_3$

	IAM	HAR	pHAR
N1-H1	0.860(5)	0.984(4)	1.004(3)
N1-H2	0.894(5)	0.990(4)	0.996(3)
N2-H3	0.883(6)	0.967(4)	0.977(4)
N2-H4	0.884(5)	0.975(4)	0.978(4)
B-H5	1.149(5)	1.227(4)	1.220(3)
B-H6	1.133(5)	1.200(3)	1.200(3)
B-H7	1.120(6)	1.227(4)	1.217(3)

Mol. sym. in cry.: **1**

(e) Bis(ammonium) closo-hexaborate(6)

$(NH_4)_2\ B_6H_6$

	IAM	HAR	pHAR
N-H1	0.829(5)	0.965(6)	0.986(6)
N-H2	0.829(5)	0.965(6)	0.986(6)
N-H3	0.829(5)	0.965(6)	0.986(6)
N-H4	0.829(5)	0.965(6)	0.986(6)
B1-H1	1.091(7)	1.169(6)	1.188(6)
B2-H2	1.091(7)	1.169(6)	1.188(6)
B3-H3	1.091(7)	1.169(6)	1.188(6)
B4-H4	1.091(7)	1.169(6)	1.188(6)
B5-H5	1.091(7)	1.169(6)	1.188(6)
B6-H6	1.091(7)	1.169(6)	1.188(6)

Mol. sym. in cry.: NH_4^+ $\mathbf{\bar{4}3m}$, $B_6H_6^{2-}$ $\mathbf{m\bar{3}m}$

(f) Bis(2,2′-bipyridinium) closo-decaborate(10) hydrate

$(C_{10}H_9N_2)_2\ B_{10}H_{10} \cdot H_2O$

	IAM	HAR	pHAR
N1a-H0a	0.932(14)	1.008(9)	1.003(7)
N1b-H0b	0.883(12)	1.001(7)	0.997(6)
B1-H1	1.098(10)	1.181(6)	1.187(6)
B2-H2	1.118(11)	1.198(7)	1.198(6)
B3-H3	1.108(11)	1.189(7)	1.192(6)
B4-H4	1.133(11)	1.198(7)	1.191(6)
B5-H5	1.106(11)	1.196(7)	1.191(6)
B6-H6	1.132(11)	1.198(7)	1.195(6)
B7-H7	1.105(11)	1.167(7)	1.171(6)
B8-H8	1.101(11)	1.192(7)	1.190(6)
B9-H9	1.112(11)	1.202(7)	1.193(6)
B10-H10	1.120(11)	1.190(7)	1.192(7)

Mol. sym. in cry.: All **1**

(g) Bis(ammonia) arachno-decaborane(12)

$(NH_3)_2B_{10}H_{12}$

	IAM	HAR	pHAR
N1-H6	0.837(12)	0.962(10)	0.963(8)
N1-H10	0.776(18)	0.947(15)	0.953(12)
N1-H13	0.837(12)	0.962(10)	0.963(8)
N2-H11	0.82(3)	0.93(2)	0.936(16)
N2-H12	0.824(14)	0.915(11)	0.921(9)
N2-H14	0.824(14)	0.915(11)	0.921(9)
B1-H5	1.076(15)	1.166(13)	1.169(12)
B2-H7*	1.265(12)	1.321(10)	1.322(9)
B2-H8	1.103(11)	1.189(8)	1.185(7)
B3-H1	1.121(15)	1.189(12)	1.186(11)
B4-H3	1.094(11)	1.195(9)	1.182(8)

(Unit: Å)

(g) continued	IAM	HAR	pHAR
B5-H2	1.096(12)	1.182(9)	1.179(8)
B5-H15*	1.239(12)	1.317(11)	1.317(9)
B6-H9	1.047(15)	1.171(12)	1.175(11)
B7-H4	1.060(16)	1.171(13)	1.173(11)
B8-H16	1.094(11)	1.195(9)	1.182(8)
B9-H7*	1.239(12)	1.317(11)	1.317(9)
B9-H17	1.096(12)	1.182(9)	1.179(8)
B10-H15*	1.265(12)	1.321(10)	1.322(9)
B10-H18	1.103(11)	1.189(8)	1.185(7)

Mol. sym. in cry.: **m** * B-H-B bridges

(h) Bis(acetonitrile) arachno-decaborane(12)

$(CH_3CN)_2B_{10}H_{12}$

	IAM	HAR	pHAR
B1-H1	1.089(8)	1.184(4)	1.181(4)
B2-H3	1.078(8)	1.171(4)	1.170(4)
B2-H10*	1.307(8)	1.319(5)	1.321(4)
B3-H4	1.097(8)	1.182(4)	1.178(4)
B3-H9*	1.292(8)	1.318(5)	1.322(4)
B4-H2	1.094(8)	1.179(4)	1.180(4)
B5-H8	1.094(8)	1.180(4)	1.183(4)
B6-H11	1.089(8)	1.184(4)	1.181(4)
B7-H10*	1.292(8)	1.318(5)	1.322(4)
B7-H12	1.097(8)	1.182(4)	1.178(4)
B8-H13	1.094(8)	1.179(4)	1.180(4)
B9-H9*	1.307(8)	1.319(5)	1.321(4)
B9-H14	1.078(8)	1.171(4)	1.170(4)
B10-H15	1.094(8)	1.180(4)	1.183(4)

Mol. sym. in cry.: **2** * B-H-B bridges

(i) Bis(2,6-lutidinium) closo-dodecaborate(12)

$((CH_3)_2C_5H_4N)_2\ B_{12}H_{12}$

	IAM	HAR	pHAR
N-H4	0.894(11)	1.006(9)	1.002(8)
B1-H13	1.134(9)	1.189(7)	1.190(6)
B2-H14	1.148(9)	1.242(8)	1.240(7)
B3-H16	1.120(10)	1.179(7)	1.185(6)
B4-H12	1.141(10)	1.199(7)	1.213(7)
B5-H11	1.162(8)	1.235(6)	1.240(6)
B6-H15	1.129(11)	1.206(8)	1.190(7)
B7-H17	1.120(10)	1.179(7)	1.185(6)
B8-H18	1.162(8)	1.235(6)	1.240(6)
B9-H19	1.134(9)	1.189(7)	1.190(6)
B10-H20	1.129(11)	1.206(8)	1.190(7)
B11-H21	1.141(10)	1.199(7)	1.213(7)
B12-H22	1.148(9)	1.242(8)	1.240(7)

Mol. sym. in cry.: $((CH_3)_2C_5H_4N)^+$ **1**, $B_{12}H_{12}{}^{2-}$ **-1**

8. Xylitol: O-H and C-H bond lengths

We conducted IAM, HAR, and pHAR on xylitol using the data from ref. 10 to further test the performance of pHAR for O–H and C–H bonds. The refined structures are compared with the neutron diffraction data of the same compound.[11] The O–H and C–H bonds in the boranes/borates are also included in the test (Figure S10). The refined C–H bonds show better agreement with the reference data than the O–H bonds. A detailed discussion of this phenomenon, in comparison with B–H and N–H bonds, is given in the main manuscript.

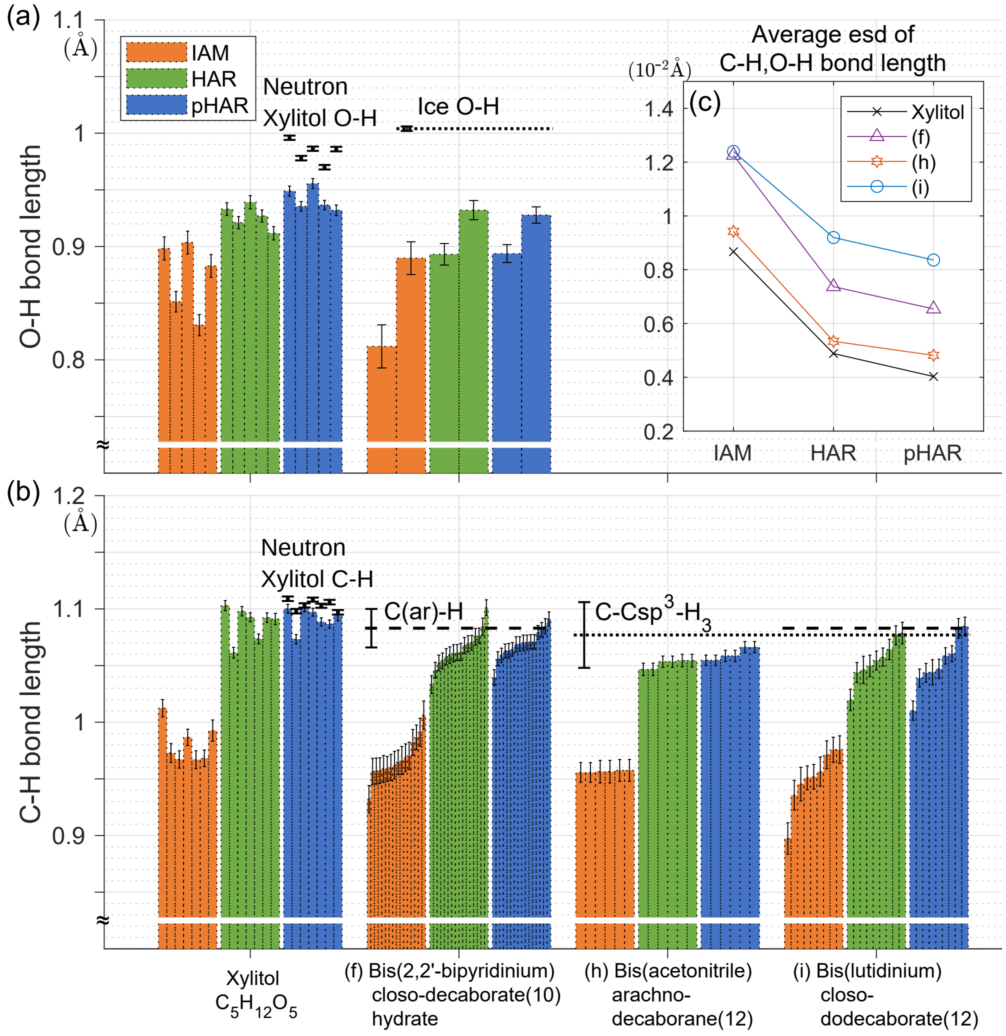

Figure S10: (a) O–H and (b) C–H bond lengths of xylitol and borane/borate systems containing such bonds. The O–H and C–H bonds of xylitol are compared with the neutron diffraction data from ref. 11. For the other compounds, the dotted and dashed lines indicate the mean bond lengths determined by neutron diffraction for C-H[12] and for O-H.[13] (c) Average esd of C–H and O–H bond lengths.

Table S14: Refined C-H and O-H bond lengths of xylitol and $\Delta r/\sigma$

Xylitol, $C_5H_{12}O_5$

Length (Å) $\Delta r/\sigma$	IAM	HAR	pHAR	Neutron, Ref. 11
O1-H11	0.898(10)	0.933(6)	0.949(4)	0.9960(19)
	-9.4	-10.7	-9.7	-
O2-H12	0.851(9)	0.921(5)	0.935(4)	0.978(2)
	-13.6	-10.0	-9.1	-
O3-H13	0.904(10)	0.939(6)	0.956(4)	0.9863(19)
	-8.1	-7.7	-6.4	-
O4-H14	0.831(9)	0.927(5)	0.937(4)	0.970(2)
	-14.6	-7.6	-7.2	-
O5-H15	0.883(10)	0.912(6)	0.932(5)	0.986(2)
	-10.1	-12.0	-10.7	-
C1-H1A	1.012(8)	1.103(4)	1.100(4)	1.109(2)
	-12.3	-1.3	-2.0	-
C1-H1B	0.973(8)	1.061(5)	1.073(4)	1.098(2)
	-14.7	-7.1	-5.4	-
C2-H2	0.967(8)	1.098(4)	1.101(4)	1.1031(19)
	-17.2	-1.1	-0.5	-
C3-H3	0.987(7)	1.093(4)	1.097(3)	1.1082(18)
	-16.4	-3.6	-2.9	-
C4-H4	0.967(8)	1.074(4)	1.089(4)	1.1028(18)
	-16.7	-6.3	-3.5	-
C5-H5B	0.968(7)	1.092(4)	1.087(4)	1.106(2)
	-18.2	-2.9	-4.7	-
C5-H5A	0.993(9)	1.091(5)	1.094(4)	1.097(2)
	-10.9	-1.1	-0.7	-
Average				
$\lvert\Delta r_{\text{O-H}}\rvert$	0.121(24)	0.058(13)	0.043(9)	-
$\lvert\Delta r_{\text{C-H}}\rvert$	0.128(22)	0.018(10)	0.015(6)	-

For the evaluation of $\Delta r/\sigma$, each σ was calculated using the formula, $\sigma_{X+Y} = \sqrt{\sigma_X^2 + \sigma_Y^2}$.

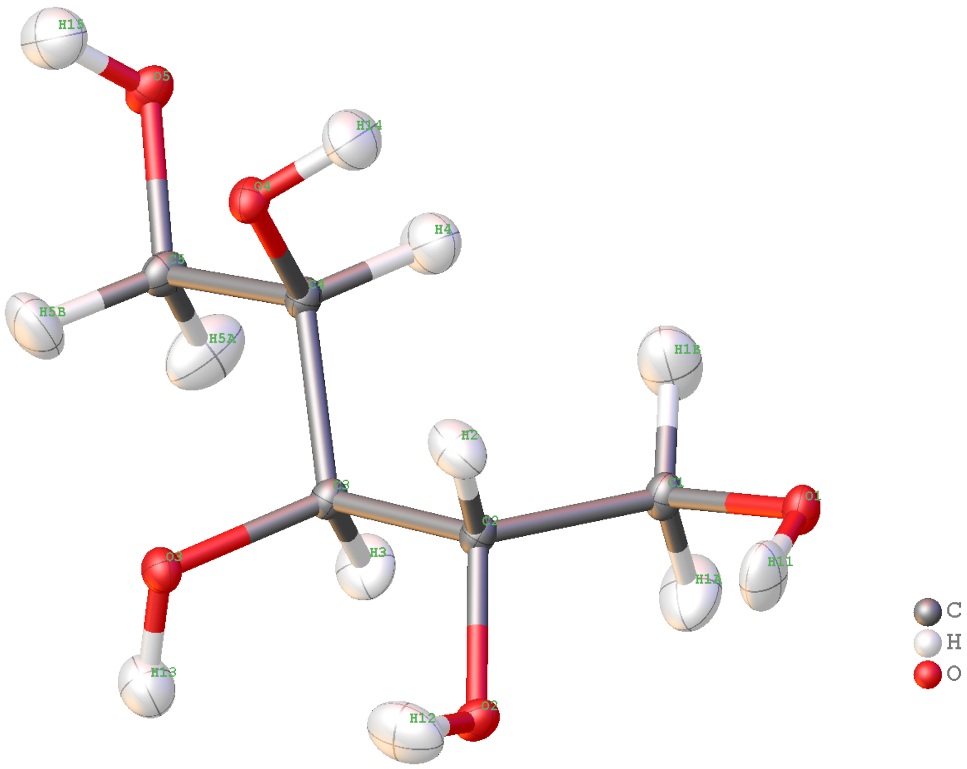

Figure S11: Xylitol refined with pHAR

References

(1) Malaspina, L. A.; Genoni, A.; Grabowsky, S. *lamaGOET*: an interface for quantum crystallography. *J. Appl. Crystallogr.* **2021**, *54*, 987–995.

(2) Ruth, P. N.; Herbst-Irmer, R.; Stalke, D. Hirshfeld atom refinement based on projector augmented wave densities with periodic boundary conditions. *IUCrJ* **2022**, *9*, 286–297.

(3) Whitten, A. E.; Spackman, M. A. Anisotropic displacement parameters for H atoms using an ONIOM approach. *Acta Crystallogr., Sect. B* **2006**, *62*, 875–888.

(4) Malaspina, L. A.; Wieduwilt, E. K.; Bergmann, J.; Kleemiss, F.; Meyer, B.; Ruiz-López, M. F.; Pal, R.; Hupf, E.; Beckmann, J.; Piltz, R. O.; Edwards, A. J.; Grabowsky, S.; Genoni, A. Fast and Accurate Quantum Crystallography: From Small to Large, from Light to Heavy. *J. Phys. Chem. Lett.* **2019**, *10*, 6973–6982.

(5) Malaspina, L. A.; Edwards, A. J.; Woińska, M.; Jayatilaka, D.; Turner, M. J.; Price, J. R.; Herbst-Irmer, R.; Sugimoto, K.; Nishibori, E.; Grabowsky, S. Predicting the Position of the Hydrogen Atom in the Short Intramolecular Hydrogen Bond of the Hydrogen Maleate Anion from Geometric Correlations. *Cryst. Growth Des.* **2017**, *17*, 3812–3825.

(6) Coppens, P. *X-Ray Charge Densities and Chemical Bonding*; Oxford University Press, 1997; Chapter 7.

(7) Mebs, S.; Grabowsky, S.; Förster, D.; Kickbusch, R.; Hartl, M.; Daemen, L. L.; Morgenroth, W.; Luger, P.; Paulus, B.; Lentz, D. Charge Transfer via the Dative N –B Bond and Dihydrogen Contacts. Experimental and Theoretical Electron Density Studies of Small Lewis Acid- Base Adducts. *J. Phys. Chem. A* **2010**, *114*, 10185–10196.

(8) Mebs, S.; Kalinowski, R.; Grabowsky, S.; Förster, D.; Kickbusch, R.; Justus, E.; Morgenroth, W.; Paulmann, C.; Luger, P.; Gabel, D.; Lentz, D. Real-space indicators for

chemical bonding. Experimental and theoretical electron density studies of four deltahedral boranes. *Inorg. Chem.* **2011**, *50*, 90–103.

(9) Jayatilaka, D.; Grimwood, D. J. Wavefunctions derived from experiment. I. Motivation and theory. *Acta Crystallogr., Sect. A* **2001**, *57*, 76–86.

(10) Madsen, A. Ø.; Sørensen, H. O.; Flensburg, C.; Stewart, R. F.; Larsen, S. Modeling of the nuclear parameters for H atoms in X-ray charge-density studies. *Acta Crystallogr., Sect. A* **2004**, *60*, 550–561.

(11) Madsen, A. Ø.; Mason, S.; Larsen, S. A neutron diffraction study of xylitol: derivation of mean square internal vibrations for H atoms from a rigid-body description. *Acta Crystallogr., Sect. B* **2003**, *59*, 653–663.

(12) Allen, F. H.; Bruno, I. J. Bond lengths in organic and metal-organic compounds revisited: *X*—H bond lengths from neutron diffraction data. *Acta Crystallogr., Sect. B* **2010**, *66*, 380–386.

(13) Kuhs, W. F.; Lehmann, M. S. The structure of the ice Ih by neutron diffraction. *J. Phys. Chem.* **1983**, *87*, 4312–4313.
