# Supplementary material for: Periodic Hirshfeld Atom Refinement
Source: J Phys Chem Lett. 2026 Feb 27;17(11):3170–9. doi: 10.1021/acs.jpclett.5c03918 (PMC13007019; doi:10.1021/acs.jpclett.5c03918)

checkCIF/PLATON report

Structure factors have been supplied for datablock(s) Bisbipyridinium_closo-decaborate_hydrate__IAM

THIS REPORT IS FOR GUIDANCE ONLY. IF USED AS PART OF A REVIEW PROCEDURE FOR PUBLICATION, IT SHOULD NOT REPLACE THE EXPERTISE OF AN EXPERIENCED CRYSTALLOGRAPHIC REFEREE.

No syntax errors found.   CIF dictionary   Interpreting this report

Datablock: Bisbipyridinium_closo-decaborate_hydrate__IAM

```
Bond precision:   C-C = 0.0008 A          Wavelength=0.51660

Cell:            a=9.2320(18)      b=9.4370(19)      c=14.613(3)
                 alpha=86.66(3)    beta=83.88(3)     gamma=72.83(3)
Temperature:     9 K

                 Calculated                 Reported
Volume           1209.0(5)                  1209.0(5)
Space group      P -1                       P -1
Hall group       -P 1                       -P 1
Moiety formula   2(C10 H9 N2), B10 H10, H2 O 2(C10 H9 N2), B10 H10, H2 O
Sum formula      C20 H30 B10 N4 O           B10 C20 H30 N4 O
Mr               450.58                     450.60
Dx,g cm-3        1.238                      1.238
Z                2                          2
Mu (mm-1)        0.042                      0.036
F000             472.0                      472.0
F000'            471.96
h,k,lmax         18,19,29                   18,19,29
Nref             21521                      13011
Tmin,Tmax        0.993,0.995
Tmin'            0.993

Correction method= Not given

Data completeness= 0.605          Theta(max)= 31.800

                                                       wR2(reflections)=
R(reflections)= 0.0365( 13011)                         wR= 0.0453(
                                                       13011)
S = 1.058                  Npar= 436
```

The following ALERTS were generated. Each ALERT has the format
test-name_ALERT_alert-type_alert-level.
Click on the hyperlinks for more details of the test.

Alert level A

```
PLAT703_ALERT_1_A Torsion Calc    -0.23(6), Rep     0.00(5), Dev..       3.83 Sigma
              B1  -B2  -B5  -B8    1_555  1_555  1_555  1_555      #  264 Check
```

Author Response: Very small torsion angles are regarded as 0 degree. For example, the torsion angle for B1-B2-B5-B8 is equal to arccos(0.9999916). Considering the significant digit in the cif file, the value 0.9999916 was rounded to be 1.0000, resulting in arccos(1.0) = 0 deg.

Alert level B

```
PLAT029_ALERT_3_B _diffrn_measured_fraction_theta_full value Low .      0.954 Why?
```

Author Response: Refer to Stefan Mebs, et al., Inorganic Chemistry 2011 50 (1), 90-103 (DOI: 10.1021/ic1013158).

```
PLAT097_ALERT_2_B Large Reported Max.  (Positive) Residual Density       0.96 eA-3
```

Author Response: C-C bonds near N atom are poorly modeled in the IAM.

```
PLAT703_ALERT_1_B Torsion Calc    -0.23(9), Rep     0.00(8), Dev..       2.56 Sigma
              C2B -C3B -C4B -C5B   1_555  1_555  1_555  1_555      #   56 Check
```

Author Response: Very small torsion angles are regarded as 0 degree. For example, the torsion angle for B1-B2-B5-B8 is equal to arccos(0.9999916). Considering the significant digit in the cif file, the value 0.9999916 was rounded to be 1.0000, resulting in arccos(1.0) = 0 deg.

```
PLAT703_ALERT_1_B Torsion Calc    -0.15(5), Rep     0.00(4), Dev..       3.00 Sigma
              B6  -B1  -B10 -B8    1_555  1_555  1_555  1_555      #  118 Check
```

Author Response: Very small torsion angles are regarded as 0 degree. For example, the torsion angle for B1-B2-B5-B8 is equal to arccos(0.9999916). Considering the significant digit in the cif file, the value 0.9999916 was rounded to be 1.0000, resulting in arccos(1.0) = 0 deg.

```
PLAT703_ALERT_1_B Torsion Calc     0.15(5), Rep     0.00(4), Dev..       3.00 Sigma
              B6  -B8  -B10 -B1    1_555  1_555  1_555  1_555      #  142 Check
```

Author Response: Very small torsion angles are regarded as 0 degree. For example, the torsion angle for B1-B2-B5-B8 is equal to arccos(0.9999916). Considering the significant digit in the cif file, the value 0.9999916 was rounded to be 1.0000, resulting in arccos(1.0) = 0 deg.

```
PLAT703_ALERT_1_B Torsion Calc    -0.15(5), Rep     0.00(4), Dev..       3.00 Sigma
              B1  -B6  -B8  -B10   1_555  1_555  1_555  1_555      #  257 Check
```

Author Response: Very small torsion angles are regarded as 0 degree. For example, the torsion angle for B1-B2-B5-B8 is equal to arccos(0.9999916). Considering the significant digit in the cif file, the value 0.9999916 was rounded to be 1.0000, resulting in arccos(1.0) = 0 deg.

```
PLAT703_ALERT_1_B Torsion Calc    -0.13(6), Rep     0.00(5), Dev..       2.17 Sigma
              B1  -B3  -B9  -B8    1_555  1_555  1_555  1_555      #  281 Check
```

Author Response: Very small torsion angles are regarded as 0 degree. For example, the torsion angle for B1-B2-B5-B8 is equal to arccos(0.9999916). Considering the significant digit in the cif file, the value 0.9999916 was rounded to be 1.0000, resulting in arccos(1.0) = 0 deg.

```
PLAT703_ALERT_1_B Torsion Calc     0.15(5), Rep     0.00(4), Dev..       3.00 Sigma
              B10 -B1  -B6  -B8    1_555  1_555  1_555  1_555      #  367 Check
```

Author Response: Very small torsion angles are regarded as 0 degree. For example, the torsion angle for B1-B2-B5-B8 is equal to arccos(0.9999916). Considering the significant digit in the cif file, the value 0.9999916 was rounded to be 1.0000, resulting in arccos(1.0) = 0 deg.

```
PLAT919_ALERT_3_B Reflection # Likely Affected by the Beamstop ...          1 Check
                1 -1  1,
```

Author Response: Refer to Stefan Mebs, et al., Inorganic Chemistry 2011 50 (1), 90-103 (DOI: 10.1021/ic1013158).

Alert level C

```
CELLK01_ALERT_1_C  Check that the cell measurement temperature is in Kelvin.
            Value of measurement temperature given =      9.000
DIFMX02_ALERT_1_C  The maximum difference density is > 0.1*ZMAX*0.75
            The relevant atom site should be identified.
REFLE01_ALERT_3_C  The _reflns_threshold_multiplier given is >= 4
            Premultiplier =    4.01
REFLE01_ALERT_3_C  The _reflns_threshold_multiplier given is >= 4
            Premultiplier =    4.01
PLAT041_ALERT_1_C Calc. and Reported SumFormula    Strings  Differ     Please Check
```

```
             Calc: C20 H30 B10 N4 O
             Rep.: B10 C20 H30 N4 O
PLAT703_ALERT_1_C Torsion Calc     0.11(9), Rep     0.00(8), Dev..       1.22 Sigma
             C6A -N2A -C10A-C9A   1_555  1_555  1_555  1_555      #   24 Check
```

Author Response: Very small torsion angles are regarded as 0 degree. For example, the torsion angle for B1-B2-B5-B8 is equal to arccos(0.9999916). Considering the significant digit in the cif file, the value 0.9999916 was rounded to be 1.0000, resulting in arccos(1.0) = 0 deg.

```
PLAT703_ALERT_1_C Torsion Calc    0.19(10), Rep     0.00(9), Dev..       1.90 Sigma
             C1A -C2A -C3A -C4A   1_555  1_555  1_555  1_555      #   55 Check
```

Author Response: Very small torsion angles are regarded as 0 degree. For example, the torsion angle for B1-B2-B5-B8 is equal to arccos(0.9999916). Considering the significant digit in the cif file, the value 0.9999916 was rounded to be 1.0000, resulting in arccos(1.0) = 0 deg.

```
PLAT703_ALERT_1_C Torsion Calc    -0.09(5), Rep     0.00(5), Dev..       1.80 Sigma
             B2  -B6  -B8  -B9    1_555  1_555  1_555  1_555      #  155 Check
```

Author Response: Very small torsion angles are regarded as 0 degree. For example, the torsion angle for B1-B2-B5-B8 is equal to arccos(0.9999916). Considering the significant digit in the cif file, the value 0.9999916 was rounded to be 1.0000, resulting in arccos(1.0) = 0 deg.

```
PLAT703_ALERT_1_C Torsion Calc     0.06(5), Rep     0.00(5), Dev..       1.20 Sigma
             B3  -B1  -B6  -B5    1_555  1_555  1_555  1_555      #  304 Check
```

Author Response: Very small torsion angles are regarded as 0 degree. For example, the torsion angle for B1-B2-B5-B8 is equal to arccos(0.9999916). Considering the significant digit in the cif file, the value 0.9999916 was rounded to be 1.0000, resulting in arccos(1.0) = 0 deg.

```
PLAT913_ALERT_3_C Missing # of Very Strong Reflections in FCF ....          6 Note
              -2 -1  2,  -2  0  2,  -2  0  3,   0  1  3,  -2  0  4,  -1  0  4,
```

Alert level G

```
ABSMU01_ALERT_1_G  Calculation of _exptl_absorpt_correction_mu
               not performed for this radiation type.
PLAT005_ALERT_5_G No Embedded Refinement Details Found  in the CIF     Please Do !
PLAT154_ALERT_1_G The s.u.'s on the Cell Angles are Equal ..(Note)       0.03 Degree
PLAT415_ALERT_2_G Short Inter D-H..H-X       H1O       ..H4       .       2.07 Ang.
                                                -1+x,y,z  =      1_455 Check
PLAT720_ALERT_4_G Number of Unusual/Non-Standard Labels .........          2 Note
             H0A     H0B
PLAT808_ALERT_5_G No Parseable SHELXL Style Weighting Scheme Found     Please Check
PLAT883_ALERT_1_G Absent Datum for _atom_sites_solution_primary ..     Please Do !
```

```
PLAT910_ALERT_3_G Missing FCF Reflection(s) Below Theta(Min)[Deg]=       1.64 Note
                0  0  1,
PLAT911_ALERT_3_G Missing FCF Refl Between Thmin & STh/L=    0.600        864 Report
               -7  1  0,  -5  1  0,   9  1  0,  10  1  0,  -9  2  0,  -8  2  0,
               -7  2  0,  -6  2  0,   6  2  0,  -8  3  0,   8  3  0,   9  3  0,
               -6  4  0,   3  4  0,   7  4  0,   9  4  0,  -7  5  0,   2  5  0,
                3  5  0,   8  5  0,   7  6  0,  10  6  0,   0  7  0,  -2  8  0,
               -2  9  0,   5 10  0,  -3-11  1,  -6-10  1,   0-10  1,  -7 -9  1,
                   (  834 More Missing: see the .ckf listing file)
PLAT912_ALERT_4_G Missing # of FCF Reflections Above STh/L=  0.600       7641 Note
PLAT929_ALERT_5_G No Weight Pars,Obs and Calc R1,wR2,S not Checked          ! Info
PLAT961_ALERT_5_G Dataset Contains no Negative Intensities .......     Please Check
PLAT969_ALERT_5_G The 'Henn et al.' R-Factor-gap value ..........      1.093 Note
              Predicted wR2: Based on SigI**2  8.56 or SHELX Weight  8.56
PLAT978_ALERT_2_G Number C-C Bonds with Positive Residual Density.         16 Info
PLAT980_ALERT_1_G No Anomalous Scattering Factors Found in CIF ...     Please Check
PLAT992_ALERT_5_G Repd & Actual _reflns_number_gt Values Differ by          3 Check
```

```
   1 ALERT level A = Most likely a serious problem - resolve or explain
   9 ALERT level B = A potentially serious problem, consider carefully
  10 ALERT level C = Check. Ensure it is not caused by an omission or oversight
  16 ALERT level G = General information/check it is not something unexpected

  18 ALERT type 1 CIF construction/syntax error, inconsistent or missing data
   3 ALERT type 2 Indicator that the structure model may be wrong or deficient
   7 ALERT type 3 Indicator that the structure quality may be low
   2 ALERT type 4 Improvement, methodology, query or suggestion
   6 ALERT type 5 Informative message, check
```

It is advisable to attempt to resolve as many as possible of the alerts in all categories. Often the minor alerts point to easily fixed oversights, errors and omissions in your CIF or refinement strategy, so attention to these fine details can be worthwhile. In order to resolve some of the more serious problems it may be necessary to carry out additional measurements or structure refinements. However, the purpose of your study may justify the reported deviations and the more serious of these should normally be commented upon in the discussion or experimental section of a paper or in the "special_details" fields of the CIF. checkCIF was carefully designed to identify outliers and unusual parameters, but every test has its limitations and alerts that are not important in a particular case may appear. Conversely, the absence of alerts does not guarantee there are no aspects of the results needing attention. It is up to the individual to critically assess their own results and, if necessary, seek expert advice.

Publication of your CIF in IUCr journals

A basic structural check has been run on your CIF. These basic checks will be run on all CIFs submitted for publication in IUCr journals (*Acta Crystallographica*, *Journal of Applied Crystallography*, *Journal of Synchrotron Radiation*); however, if you intend to submit to *Acta Crystallographica Section C* or *E* or *IUCrData*, you should make sure that full publication checks are run on the final version of your CIF prior to submission.

Publication of your CIF in other journals

Please refer to the *Notes for Authors* of the relevant journal for any special instructions relating to CIF submission.

PLATON version of 04/06/2025; check.def file version of 30/05/2025

Datablock Bisbipyridinium_closo-decaborate_hydrate__IAM - ellipsoid plot

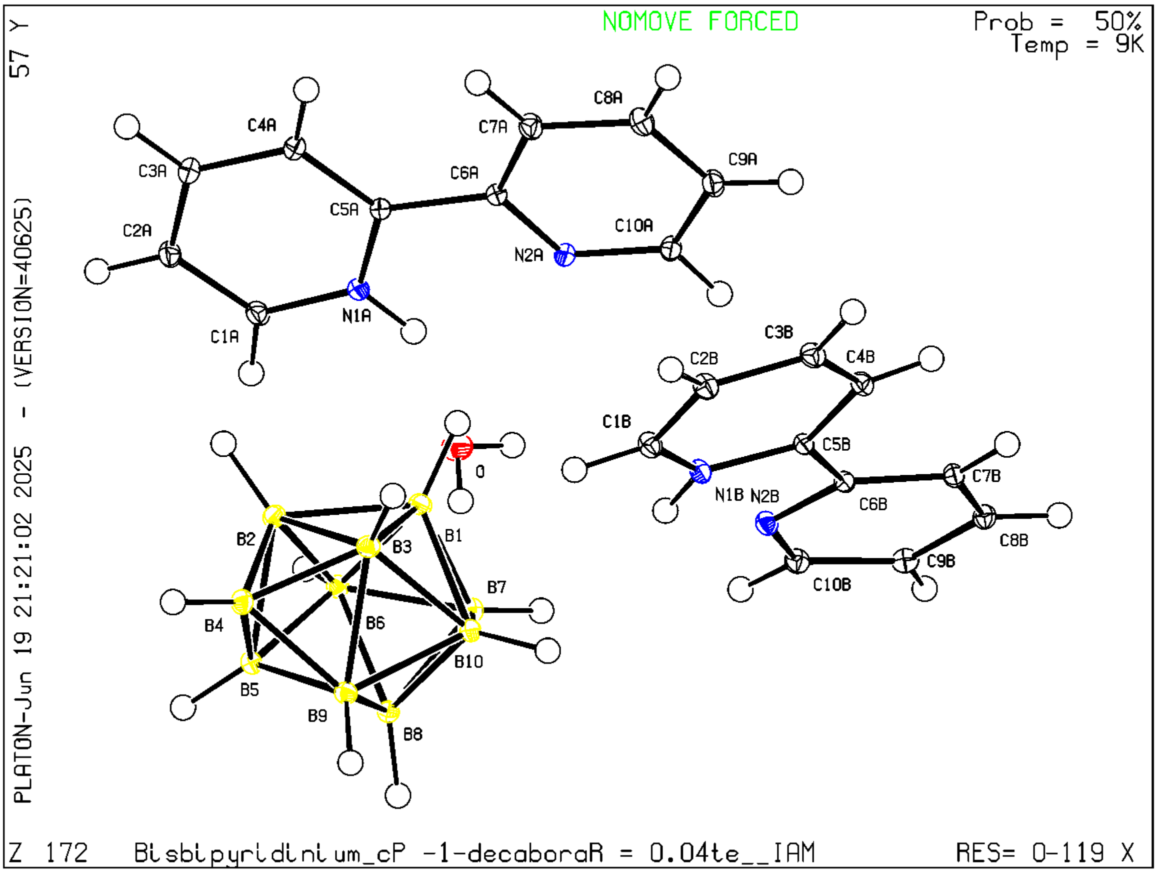

Supplement: Supplementary file 2 [file jz5c03918_si_002.zip › F_bi22py-closo-decaborate(10)/Bis(2,2'-bipyridinium)_closo-decaborate(10)_hydrate__IAM.pdf]
