# Supplementary material for: Periodic Hirshfeld Atom Refinement
Source: J Phys Chem Lett. 2026 Feb 27;17(11):3170–9. doi: 10.1021/acs.jpclett.5c03918 (PMC13007019; doi:10.1021/acs.jpclett.5c03918)

checkCIF/PLATON report

Structure factors have been supplied for datablock(s) Bisacetonitrile_arachno-decaborane12_HAR

THIS REPORT IS FOR GUIDANCE ONLY. IF USED AS PART OF A REVIEW PROCEDURE FOR PUBLICATION, IT SHOULD NOT REPLACE THE EXPERTISE OF AN EXPERIENCED CRYSTALLOGRAPHIC REFEREE.

No syntax errors found. CIF dictionary Interpreting this report

Datablock: Bisacetonitrile_arachno-decaborane12_HAR

```
Bond precision:    C-C = 0.0005 A           Wavelength=0.71073

Cell:              a=15.187(4)     b=11.1265(15)      c=7.7617(14)
                   alpha=90        beta=112.66(1)     gamma=90
Temperature:       95 K

                 Calculated                  Reported
Volume           1210.3(4)                   1210.3(4)
Space group      C 2/c                       C 1 2/c 1
Hall group       -C 2yc                      -C 2yc
Moiety formula   C4 H18 B10 N2               C4 H18 B10 N2
Sum formula      C4 H18 B10 N2               C4 H18 B10 N2
Mr               202.30                      202.31
Dx,g cm-3        1.110                       1.110
Z                4                           4
Mu (mm-1)        0.054                       0.054
F000             424.0                       424.0
F000'            424.04
h,k,lmax         34,25,17                    34,25,17
Nref             7710                        5143
Tmin,Tmax        0.978,0.981
Tmin'            0.973

Correction method= Not given

Data completeness= 0.667           Theta(max)= 54.800

                                                       wR2(reflections)=
R(reflections)= 0.0212( 5143)                          wR= 0.0201( 5143)

S = 0.815                  Npar= 154
```

The following ALERTS were generated. Each ALERT has the format
test-name_ALERT_alert-type_alert-level.
Click on the hyperlinks for more details of the test.

Alert level C

```
REFLE01_ALERT_3_C  The _reflns_threshold_multiplier given is >= 4
            Premultiplier =    4.01
REFLE01_ALERT_3_C  The _reflns_threshold_multiplier given is >= 4
            Premultiplier =    4.01
PLAT029_ALERT_3_C _diffrn_measured_fraction_theta_full value Low .      0.963 Why?
PLAT303_ALERT_2_C Full Occupancy Atom H9        with # Connections       2.00 Check
PLAT711_ALERT_1_C BOND    Unknown or Inconsistent Label .........          N2 Check
                    N2          C3
PLAT711_ALERT_1_C BOND    Unknown or Inconsistent Label .........          N2 Check
                    N2          B10
PLAT711_ALERT_1_C BOND    Unknown or Inconsistent Label .........          C3 Check
                    C3          C4
PLAT711_ALERT_1_C BOND    Unknown or Inconsistent Label .........          C4 Check
                    C4          H16
PLAT711_ALERT_1_C BOND    Unknown or Inconsistent Label .........          C4 Check
                    C4          H17
PLAT711_ALERT_1_C BOND    Unknown or Inconsistent Label .........          C4 Check
                    C4          H18
PLAT711_ALERT_1_C BOND    Unknown or Inconsistent Label .........          B6 Check
                    B1          B6
PLAT711_ALERT_1_C BOND    Unknown or Inconsistent Label .........          B7 Check
                    B1          B7
PLAT711_ALERT_1_C BOND    Unknown or Inconsistent Label .........          B8 Check
                    B1          B8
PLAT711_ALERT_1_C BOND    Unknown or Inconsistent Label .........          B7 Check
                    B2          B7
PLAT711_ALERT_1_C BOND    Unknown or Inconsistent Label .........          B6 Check
                    B3          B6
PLAT711_ALERT_1_C BOND    Unknown or Inconsistent Label .........          B9 Check
                    B3          B9
PLAT711_ALERT_1_C BOND    Unknown or Inconsistent Label .........          B6 Check
                    B4          B6
PLAT711_ALERT_1_C BOND    Unknown or Inconsistent Label .........          B6 Check
                    B6          B8
PLAT711_ALERT_1_C BOND    Unknown or Inconsistent Label .........          B6 Check
                    B6          B9
PLAT711_ALERT_1_C BOND    Unknown or Inconsistent Label .........          B7 Check
                    B7          B8
PLAT711_ALERT_1_C BOND    Unknown or Inconsistent Label .........          B7 Check
                    B7          B10
PLAT711_ALERT_1_C BOND    Unknown or Inconsistent Label .........          B8 Check
                    B8          B9
PLAT711_ALERT_1_C BOND    Unknown or Inconsistent Label .........          B8 Check
                    B8          B10
PLAT711_ALERT_1_C BOND    Unknown or Inconsistent Label .........          B9 Check
                    B9          B10
PLAT711_ALERT_1_C BOND    Unknown or Inconsistent Label .........         H10 Check
                    B2          H10
PLAT711_ALERT_1_C BOND    Unknown or Inconsistent Label .........          B6 Check
                    B6          H11
```

```
PLAT711_ALERT_1_C BOND    Unknown or Inconsistent Label .........        B7 Check
                  B7          H10
PLAT711_ALERT_1_C BOND    Unknown or Inconsistent Label .........        B7 Check
                  B7          H12
PLAT711_ALERT_1_C BOND    Unknown or Inconsistent Label .........        B8 Check
                  B8          H13
PLAT711_ALERT_1_C BOND    Unknown or Inconsistent Label .........        B9 Check
                  B9          H9
PLAT711_ALERT_1_C BOND    Unknown or Inconsistent Label .........        B9 Check
                  B9          H14
PLAT711_ALERT_1_C BOND    Unknown or Inconsistent Label .........       B10 Check
                  B10         H15
PLAT712_ALERT_1_C ANGLE   Unknown or Inconsistent Label .........        N2 Check
                  N2          C3          C4
PLAT712_ALERT_1_C ANGLE   Unknown or Inconsistent Label .........        N2 Check
                  N2          B10         B7
PLAT712_ALERT_1_C ANGLE   Unknown or Inconsistent Label .........        N2 Check
                  N2          B10         B8
PLAT712_ALERT_1_C ANGLE   Unknown or Inconsistent Label .........        N2 Check
                  N2          B10         B9
PLAT712_ALERT_1_C ANGLE   Unknown or Inconsistent Label .........        N2 Check
                  N2          B10         H15
PLAT712_ALERT_1_C ANGLE   Unknown or Inconsistent Label .........        C3 Check
                  C3          N2          B10
PLAT712_ALERT_1_C ANGLE   Unknown or Inconsistent Label .........        C3 Check
                  C3          C4          H16
PLAT712_ALERT_1_C ANGLE   Unknown or Inconsistent Label .........        C3 Check
                  C3          C4          H17
PLAT712_ALERT_1_C ANGLE   Unknown or Inconsistent Label .........        C3 Check
                  C3          C4          H18
PLAT712_ALERT_1_C ANGLE   Unknown or Inconsistent Label .........        B7 Check
                  B1          B2          B7
PLAT712_ALERT_1_C ANGLE   Unknown or Inconsistent Label .........        B6 Check
                  B1          B4          B6
PLAT712_ALERT_1_C ANGLE   Unknown or Inconsistent Label .........        B6 Check
                  B1          B6          B3
PLAT712_ALERT_1_C ANGLE   Unknown or Inconsistent Label .........        B6 Check
                  B1          B6          B4
PLAT712_ALERT_1_C ANGLE   Unknown or Inconsistent Label .........        B6 Check
                  B1          B6          B8
PLAT712_ALERT_1_C ANGLE   Unknown or Inconsistent Label .........        B6 Check
                  B1          B6          B9
PLAT712_ALERT_1_C ANGLE   Unknown or Inconsistent Label .........        B7 Check
                  B1          B7          B2
PLAT712_ALERT_1_C ANGLE   Unknown or Inconsistent Label .........        B7 Check
                  B1          B7          B8
PLAT712_ALERT_1_C ANGLE   Unknown or Inconsistent Label .........        B7 Check
                  B1          B7          B10
PLAT712_ALERT_1_C ANGLE   Unknown or Inconsistent Label .........        B8 Check
                  B1          B8          B6
PLAT712_ALERT_1_C ANGLE   Unknown or Inconsistent Label .........        B8 Check
                  B1          B8          B7
PLAT712_ALERT_1_C ANGLE   Unknown or Inconsistent Label .........        B8 Check
                  B1          B8          B9
PLAT712_ALERT_1_C ANGLE   Unknown or Inconsistent Label .........        B8 Check
                  B1          B8          B10
PLAT712_ALERT_1_C ANGLE   Unknown or Inconsistent Label .........        B6 Check
```

```
                    B2          B1          B6
PLAT712_ALERT_1_C ANGLE   Unknown or Inconsistent Label .........        B7 Check
                    B2          B1          B7
PLAT712_ALERT_1_C ANGLE   Unknown or Inconsistent Label .........        B8 Check
                    B2          B1          B8
PLAT712_ALERT_1_C ANGLE   Unknown or Inconsistent Label .........        B6 Check
                    B2          B4          B6
PLAT712_ALERT_1_C ANGLE   Unknown or Inconsistent Label .........        B7 Check
                    B2          B7          B8
PLAT712_ALERT_1_C ANGLE   Unknown or Inconsistent Label .........        B7 Check
                    B2          B7          B10
PLAT712_ALERT_1_C ANGLE   Unknown or Inconsistent Label .........        B6 Check
                    B3          B4          B6
PLAT712_ALERT_1_C ANGLE   Unknown or Inconsistent Label .........        B6 Check
                    B3          B6          B4
PLAT712_ALERT_1_C ANGLE   Unknown or Inconsistent Label .........        B6 Check
                    B3          B6          B8
PLAT712_ALERT_1_C ANGLE   Unknown or Inconsistent Label .........        B6 Check
                    B3          B6          B9
PLAT712_ALERT_1_C ANGLE   Unknown or Inconsistent Label .........        B9 Check
                    B3          B9          B6
PLAT712_ALERT_1_C ANGLE   Unknown or Inconsistent Label .........        B9 Check
                    B3          B9          B8
PLAT712_ALERT_1_C ANGLE   Unknown or Inconsistent Label .........        B9 Check
                    B3          B9          B10
PLAT712_ALERT_1_C ANGLE   Unknown or Inconsistent Label .........        B6 Check
                    B4          B1          B6
PLAT712_ALERT_1_C ANGLE   Unknown or Inconsistent Label .........        B7 Check
                    B4          B1          B7
PLAT712_ALERT_1_C ANGLE   Unknown or Inconsistent Label .........        B8 Check
                    B4          B1          B8
PLAT712_ALERT_1_C ANGLE   Unknown or Inconsistent Label .........        B7 Check
                    B4          B2          B7
PLAT712_ALERT_1_C ANGLE   Unknown or Inconsistent Label .........        B6 Check
                    B4          B3          B6
PLAT712_ALERT_1_C ANGLE   Unknown or Inconsistent Label .........        B9 Check
                    B4          B3          B9
PLAT712_ALERT_1_C ANGLE   Unknown or Inconsistent Label .........        B6 Check
                    B4          B6          B8
PLAT712_ALERT_1_C ANGLE   Unknown or Inconsistent Label .........        B6 Check
                    B4          B6          B9
PLAT712_ALERT_1_C ANGLE   Unknown or Inconsistent Label .........        B7 Check
                    B5          B2          B7
PLAT712_ALERT_1_C ANGLE   Unknown or Inconsistent Label .........        B6 Check
                    B5          B3          B6
PLAT712_ALERT_1_C ANGLE   Unknown or Inconsistent Label .........        B9 Check
                    B5          B3          B9
PLAT712_ALERT_1_C ANGLE   Unknown or Inconsistent Label .........        B6 Check
                    B5          B4          B6
PLAT712_ALERT_1_C ANGLE   Unknown or Inconsistent Label .........        B6 Check
                    B6          B1          B7
PLAT712_ALERT_1_C ANGLE   Unknown or Inconsistent Label .........        B6 Check
                    B6          B1          B8
PLAT712_ALERT_1_C ANGLE   Unknown or Inconsistent Label .........        B6 Check
                    B6          B3          B9
PLAT712_ALERT_1_C ANGLE   Unknown or Inconsistent Label .........        B6 Check
                    B6          B8          B7
```

```
PLAT712_ALERT_1_C ANGLE   Unknown or Inconsistent Label .........          B6 Check
                    B6          B8          B9
PLAT712_ALERT_1_C ANGLE   Unknown or Inconsistent Label .........          B6 Check
                    B6          B8          B10
PLAT712_ALERT_1_C ANGLE   Unknown or Inconsistent Label .........          B6 Check
                    B6          B9          B8
PLAT712_ALERT_1_C ANGLE   Unknown or Inconsistent Label .........          B6 Check
                    B6          B9          B10
PLAT712_ALERT_1_C ANGLE   Unknown or Inconsistent Label .........          B7 Check
                    B7          B1          B8
PLAT712_ALERT_1_C ANGLE   Unknown or Inconsistent Label .........          B7 Check
                    B7          B8          B9
PLAT712_ALERT_1_C ANGLE   Unknown or Inconsistent Label .........          B7 Check
                    B7          B8          B10
PLAT712_ALERT_1_C ANGLE   Unknown or Inconsistent Label .........          B7 Check
                    B7          B10         B8
PLAT712_ALERT_1_C ANGLE   Unknown or Inconsistent Label .........          B7 Check
                    B7          B10         B9
PLAT712_ALERT_1_C ANGLE   Unknown or Inconsistent Label .........          B8 Check
                    B8          B6          B9
PLAT712_ALERT_1_C ANGLE   Unknown or Inconsistent Label .........          B8 Check
                    B8          B7          B10
PLAT712_ALERT_1_C ANGLE   Unknown or Inconsistent Label .........          B8 Check
                    B8          B9          B10
PLAT712_ALERT_1_C ANGLE   Unknown or Inconsistent Label .........          B8 Check
                    B8          B10         B9
PLAT712_ALERT_1_C ANGLE   Unknown or Inconsistent Label .........          B9 Check
                    B9          B8          B10
PLAT712_ALERT_1_C ANGLE   Unknown or Inconsistent Label .........         H10 Check
                    B1          B2          H10
PLAT712_ALERT_1_C ANGLE   Unknown or Inconsistent Label .........          B6 Check
                    B1          B6          H11
PLAT712_ALERT_1_C ANGLE   Unknown or Inconsistent Label .........          B7 Check
                    B1          B7          H10
PLAT712_ALERT_1_C ANGLE   Unknown or Inconsistent Label .........          B7 Check
                    B1          B7          H12
PLAT712_ALERT_1_C ANGLE   Unknown or Inconsistent Label .........          B8 Check
                    B1          B8          H13
PLAT712_ALERT_1_C ANGLE   Unknown or Inconsistent Label .........          B7 Check
                    B2          B7          H10
PLAT712_ALERT_1_C ANGLE   Unknown or Inconsistent Label .........          B7 Check
                    B2          B7          H12
PLAT712_ALERT_1_C ANGLE   Unknown or Inconsistent Label .........          B6 Check
                    B3          B6          H11
PLAT712_ALERT_1_C ANGLE   Unknown or Inconsistent Label .........          B9 Check
                    B3          B9          H9
PLAT712_ALERT_1_C ANGLE   Unknown or Inconsistent Label .........          B9 Check
                    B3          B9          H14
PLAT712_ALERT_1_C ANGLE   Unknown or Inconsistent Label .........         H10 Check
                    B4          B2          H10
PLAT712_ALERT_1_C ANGLE   Unknown or Inconsistent Label .........          B6 Check
                    B4          B6          H11
PLAT712_ALERT_1_C ANGLE   Unknown or Inconsistent Label .........         H10 Check
                    B5          B2          H10
PLAT712_ALERT_1_C ANGLE   Unknown or Inconsistent Label .........          B6 Check
                    B6          B1          H1
PLAT712_ALERT_1_C ANGLE   Unknown or Inconsistent Label .........          B6 Check
```

```
                 B6          B3          H4
PLAT712_ALERT_1_C ANGLE   Unknown or Inconsistent Label .........          B6 Check
                 B6          B3          H9
PLAT712_ALERT_1_C ANGLE   Unknown or Inconsistent Label .........          B6 Check
                 B6          B4          H2
PLAT712_ALERT_1_C ANGLE   Unknown or Inconsistent Label .........          B6 Check
                 B6          B8          H13
PLAT712_ALERT_1_C ANGLE   Unknown or Inconsistent Label .........          B6 Check
                 B6          B9          H9
PLAT712_ALERT_1_C ANGLE   Unknown or Inconsistent Label .........          B6 Check
                 B6          B9          H14
PLAT712_ALERT_1_C ANGLE   Unknown or Inconsistent Label .........          B7 Check
                 B7          B1          H1
PLAT712_ALERT_1_C ANGLE   Unknown or Inconsistent Label .........          B7 Check
                 B7          B2          H3
PLAT712_ALERT_1_C ANGLE   Unknown or Inconsistent Label .........          B7 Check
                 B7          B2          H10
PLAT712_ALERT_1_C ANGLE   Unknown or Inconsistent Label .........          B7 Check
                 B7          B8          H13
PLAT712_ALERT_1_C ANGLE   Unknown or Inconsistent Label .........          B7 Check
                 B7          B10         H15
PLAT712_ALERT_1_C ANGLE   Unknown or Inconsistent Label .........          B8 Check
                 B8          B1          H1
PLAT712_ALERT_1_C ANGLE   Unknown or Inconsistent Label .........          B8 Check
                 B8          B6          H11
PLAT712_ALERT_1_C ANGLE   Unknown or Inconsistent Label .........          B8 Check
                 B8          B7          H10
PLAT712_ALERT_1_C ANGLE   Unknown or Inconsistent Label .........          B8 Check
                 B8          B7          H12
PLAT712_ALERT_1_C ANGLE   Unknown or Inconsistent Label .........          B8 Check
                 B8          B9          H9
PLAT712_ALERT_1_C ANGLE   Unknown or Inconsistent Label .........          B8 Check
                 B8          B9          H14
PLAT712_ALERT_1_C ANGLE   Unknown or Inconsistent Label .........          B8 Check
                 B8          B10         H15
PLAT712_ALERT_1_C ANGLE   Unknown or Inconsistent Label .........          B9 Check
                 B9          B3          H4
PLAT712_ALERT_1_C ANGLE   Unknown or Inconsistent Label .........          B9 Check
                 B9          B3          H9
PLAT712_ALERT_1_C ANGLE   Unknown or Inconsistent Label .........          B9 Check
                 B9          B6          H11
PLAT712_ALERT_1_C ANGLE   Unknown or Inconsistent Label .........          B9 Check
                 B9          B8          H13
PLAT712_ALERT_1_C ANGLE   Unknown or Inconsistent Label .........          B9 Check
                 B9          B10         H15
PLAT712_ALERT_1_C ANGLE   Unknown or Inconsistent Label .........         B10 Check
                 B10         B7          H10
PLAT712_ALERT_1_C ANGLE   Unknown or Inconsistent Label .........         B10 Check
                 B10         B7          H12
PLAT712_ALERT_1_C ANGLE   Unknown or Inconsistent Label .........         B10 Check
                 B10         B8          H13
PLAT712_ALERT_1_C ANGLE   Unknown or Inconsistent Label .........         B10 Check
                 B10         B9          H9
PLAT712_ALERT_1_C ANGLE   Unknown or Inconsistent Label .........         B10 Check
                 B10         B9          H14
PLAT712_ALERT_1_C ANGLE   Unknown or Inconsistent Label .........         H10 Check
                 B2          H10         B7
```

```
PLAT712_ALERT_1_C ANGLE   Unknown or Inconsistent Label .........          B9 Check
                    B3          H9          B9
PLAT712_ALERT_1_C ANGLE   Unknown or Inconsistent Label .........         H16 Check
                    H16         C4          H17
PLAT712_ALERT_1_C ANGLE   Unknown or Inconsistent Label .........         H16 Check
                    H16         C4          H18
PLAT712_ALERT_1_C ANGLE   Unknown or Inconsistent Label .........         H17 Check
                    H17         C4          H18
PLAT712_ALERT_1_C ANGLE   Unknown or Inconsistent Label .........         H10 Check
                    H3          B2          H10
PLAT712_ALERT_1_C ANGLE   Unknown or Inconsistent Label .........          B9 Check
                    H9          B9          H14
PLAT712_ALERT_1_C ANGLE   Unknown or Inconsistent Label .........         H10 Check
                    H10         B7          H12
PLAT713_ALERT_1_C TORSION Unknown or Inconsistent Label .........          N2 Check
                    N2          C3          C4          H16
PLAT713_ALERT_1_C TORSION Unknown or Inconsistent Label .........          N2 Check
                    N2          C3          C4          H17
PLAT713_ALERT_1_C TORSION Unknown or Inconsistent Label .........          N2 Check
                    N2          C3          C4          H18
PLAT713_ALERT_1_C TORSION Unknown or Inconsistent Label .........          B7 Check
                    N1          B5          B2          B7
PLAT713_ALERT_1_C TORSION Unknown or Inconsistent Label .........          B6 Check
                    N1          B5          B3          B6
PLAT713_ALERT_1_C TORSION Unknown or Inconsistent Label .........          B9 Check
                    N1          B5          B3          B9
PLAT713_ALERT_1_C TORSION Unknown or Inconsistent Label .........          B6 Check
                    N1          B5          B4          B6
PLAT713_ALERT_1_C TORSION Unknown or Inconsistent Label .........         H10 Check
                    N1          B5          B2          H10
PLAT713_ALERT_1_C TORSION Unknown or Inconsistent Label .........         B10 Check
                    B10         N2          C3          C4
PLAT713_ALERT_1_C TORSION Unknown or Inconsistent Label .........          B7 Check
                    B7          B10         N2          C3
PLAT713_ALERT_1_C TORSION Unknown or Inconsistent Label .........          B8 Check
                    B8          B10         N2          C3
PLAT713_ALERT_1_C TORSION Unknown or Inconsistent Label .........          B9 Check
                    B9          B10         N2          C3
PLAT713_ALERT_1_C TORSION Unknown or Inconsistent Label .........          B7 Check
                    B1          B7          B10         N2
PLAT713_ALERT_1_C TORSION Unknown or Inconsistent Label .........          B8 Check
                    B1          B8          B10         N2
PLAT713_ALERT_1_C TORSION Unknown or Inconsistent Label .........          B7 Check
                    B2          B7          B10         N2
PLAT713_ALERT_1_C TORSION Unknown or Inconsistent Label .........          B9 Check
                    B3          B9          B10         N2
PLAT713_ALERT_1_C TORSION Unknown or Inconsistent Label .........          B6 Check
                    B6          B8          B10         N2
PLAT713_ALERT_1_C TORSION Unknown or Inconsistent Label .........          B6 Check
                    B6          B9          B10         N2
PLAT713_ALERT_1_C TORSION Unknown or Inconsistent Label .........          B7 Check
                    B7          B8          B10         N2
PLAT713_ALERT_1_C TORSION Unknown or Inconsistent Label .........          B8 Check
                    B8          B7          B10         N2
PLAT713_ALERT_1_C TORSION Unknown or Inconsistent Label .........          B8 Check
                    B8          B9          B10         N2
PLAT713_ALERT_1_C TORSION Unknown or Inconsistent Label .........          B9 Check
```

```
                    B9          B8          B10         N2
PLAT713_ALERT_1_C TORSION Unknown or Inconsistent Label ..........         B6 Check
                    B1          B2          B4          B6
PLAT713_ALERT_1_C TORSION Unknown or Inconsistent Label ..........         B7 Check
                    B1          B2          B7          B8
PLAT713_ALERT_1_C TORSION Unknown or Inconsistent Label ..........         B7 Check
                    B1          B2          B7          B10
PLAT713_ALERT_1_C TORSION Unknown or Inconsistent Label ..........         B7 Check
                    B1          B4          B2          B7
PLAT713_ALERT_1_C TORSION Unknown or Inconsistent Label ..........         B6 Check
                    B1          B4          B3          B6
PLAT713_ALERT_1_C TORSION Unknown or Inconsistent Label ..........         B9 Check
                    B1          B4          B3          B9
PLAT713_ALERT_1_C TORSION Unknown or Inconsistent Label ..........         B6 Check
                    B1          B4          B6          B3
PLAT713_ALERT_1_C TORSION Unknown or Inconsistent Label ..........         B6 Check
                    B1          B4          B6          B8
PLAT713_ALERT_1_C TORSION Unknown or Inconsistent Label ..........         B6 Check
                    B1          B4          B6          B9
PLAT713_ALERT_1_C TORSION Unknown or Inconsistent Label ..........         B6 Check
                    B1          B6          B3          B4
PLAT713_ALERT_1_C TORSION Unknown or Inconsistent Label ..........         B6 Check
                    B1          B6          B3          B5
PLAT713_ALERT_1_C TORSION Unknown or Inconsistent Label ..........         B6 Check
                    B1          B6          B3          B9
PLAT713_ALERT_1_C TORSION Unknown or Inconsistent Label ..........         B6 Check
                    B1          B6          B4          B2
PLAT713_ALERT_1_C TORSION Unknown or Inconsistent Label ..........         B6 Check
                    B1          B6          B4          B3
PLAT713_ALERT_1_C TORSION Unknown or Inconsistent Label ..........         B6 Check
                    B1          B6          B4          B5
PLAT713_ALERT_1_C TORSION Unknown or Inconsistent Label ..........         B6 Check
                    B1          B6          B8          B7
PLAT713_ALERT_1_C TORSION Unknown or Inconsistent Label ..........         B6 Check
                    B1          B6          B8          B9
PLAT713_ALERT_1_C TORSION Unknown or Inconsistent Label ..........         B6 Check
                    B1          B6          B8          B10
PLAT713_ALERT_1_C TORSION Unknown or Inconsistent Label ..........         B6 Check
                    B1          B6          B9          B3
PLAT713_ALERT_1_C TORSION Unknown or Inconsistent Label ..........         B6 Check
                    B1          B6          B9          B8
PLAT713_ALERT_1_C TORSION Unknown or Inconsistent Label ..........         B6 Check
                    B1          B6          B9          B10
PLAT713_ALERT_1_C TORSION Unknown or Inconsistent Label ..........         B7 Check
                    B1          B7          B2          B4
PLAT713_ALERT_1_C TORSION Unknown or Inconsistent Label ..........         B7 Check
                    B1          B7          B2          B5
PLAT713_ALERT_1_C TORSION Unknown or Inconsistent Label ..........         B7 Check
                    B1          B7          B8          B6
PLAT713_ALERT_1_C TORSION Unknown or Inconsistent Label ..........         B7 Check
                    B1          B7          B8          B9
PLAT713_ALERT_1_C TORSION Unknown or Inconsistent Label ..........         B7 Check
                    B1          B7          B8          B10
PLAT713_ALERT_1_C TORSION Unknown or Inconsistent Label ..........         B7 Check
                    B1          B7          B10         B8
PLAT713_ALERT_1_C TORSION Unknown or Inconsistent Label ..........         B7 Check
                    B1          B7          B10         B9
```

```
PLAT713_ALERT_1_C TORSION Unknown or Inconsistent Label ..........          B8 Check
                    B1          B8          B6          B3
PLAT713_ALERT_1_C TORSION Unknown or Inconsistent Label ..........          B8 Check
                    B1          B8          B6          B4
PLAT713_ALERT_1_C TORSION Unknown or Inconsistent Label ..........          B8 Check
                    B1          B8          B6          B9
PLAT713_ALERT_1_C TORSION Unknown or Inconsistent Label ..........          B8 Check
                    B1          B8          B7          B2
PLAT713_ALERT_1_C TORSION Unknown or Inconsistent Label ..........          B8 Check
                    B1          B8          B7          B10
PLAT713_ALERT_1_C TORSION Unknown or Inconsistent Label ..........          B8 Check
                    B1          B8          B9          B3
PLAT713_ALERT_1_C TORSION Unknown or Inconsistent Label ..........          B8 Check
                    B1          B8          B9          B6
PLAT713_ALERT_1_C TORSION Unknown or Inconsistent Label ..........          B8 Check
                    B1          B8          B9          B10
PLAT713_ALERT_1_C TORSION Unknown or Inconsistent Label ..........          B8 Check
                    B1          B8          B10         B7
PLAT713_ALERT_1_C TORSION Unknown or Inconsistent Label ..........          B8 Check
                    B1          B8          B10         B9
PLAT713_ALERT_1_C TORSION Unknown or Inconsistent Label ..........          B6 Check
                    B2          B1          B4          B6
PLAT713_ALERT_1_C TORSION Unknown or Inconsistent Label ..........          B6 Check
                    B2          B1          B6          B3
PLAT713_ALERT_1_C TORSION Unknown or Inconsistent Label ..........          B6 Check
                    B2          B1          B6          B4
PLAT713_ALERT_1_C TORSION Unknown or Inconsistent Label ..........          B6 Check
                    B2          B1          B6          B8
PLAT713_ALERT_1_C TORSION Unknown or Inconsistent Label ..........          B6 Check
                    B2          B1          B6          B9
PLAT713_ALERT_1_C TORSION Unknown or Inconsistent Label ..........          B7 Check
                    B2          B1          B7          B8
PLAT713_ALERT_1_C TORSION Unknown or Inconsistent Label ..........          B7 Check
                    B2          B1          B7          B10
PLAT713_ALERT_1_C TORSION Unknown or Inconsistent Label ..........          B8 Check
                    B2          B1          B8          B6
PLAT713_ALERT_1_C TORSION Unknown or Inconsistent Label ..........          B8 Check
                    B2          B1          B8          B7
PLAT713_ALERT_1_C TORSION Unknown or Inconsistent Label ..........          B8 Check
                    B2          B1          B8          B9
PLAT713_ALERT_1_C TORSION Unknown or Inconsistent Label ..........          B8 Check
                    B2          B1          B8          B10
PLAT713_ALERT_1_C TORSION Unknown or Inconsistent Label ..........          B6 Check
                    B2          B4          B1          B6
PLAT713_ALERT_1_C TORSION Unknown or Inconsistent Label ..........          B7 Check
                    B2          B4          B1          B7
PLAT713_ALERT_1_C TORSION Unknown or Inconsistent Label ..........          B8 Check
                    B2          B4          B1          B8
PLAT713_ALERT_1_C TORSION Unknown or Inconsistent Label ..........          B6 Check
                    B2          B4          B3          B6
PLAT713_ALERT_1_C TORSION Unknown or Inconsistent Label ..........          B9 Check
                    B2          B4          B3          B9
PLAT713_ALERT_1_C TORSION Unknown or Inconsistent Label ..........          B6 Check
                    B2          B4          B6          B3
PLAT713_ALERT_1_C TORSION Unknown or Inconsistent Label ..........          B6 Check
                    B2          B4          B6          B8
PLAT713_ALERT_1_C TORSION Unknown or Inconsistent Label ..........          B6 Check
```

```
                   B2          B4          B6          B9
PLAT713_ALERT_1_C TORSION Unknown or Inconsistent Label ..........         B6 Check
                   B2          B5          B3          B6
PLAT713_ALERT_1_C TORSION Unknown or Inconsistent Label ..........         B9 Check
                   B2          B5          B3          B9
PLAT713_ALERT_1_C TORSION Unknown or Inconsistent Label ..........         B6 Check
                   B2          B5          B4          B6
PLAT713_ALERT_1_C TORSION Unknown or Inconsistent Label ..........         B7 Check
                   B2          B7          B1          B4
PLAT713_ALERT_1_C TORSION Unknown or Inconsistent Label ..........         B7 Check
                   B2          B7          B1          B6
PLAT713_ALERT_1_C TORSION Unknown or Inconsistent Label ..........         B7 Check
                   B2          B7          B1          B8
PLAT713_ALERT_1_C TORSION Unknown or Inconsistent Label ..........         B7 Check
                   B2          B7          B8          B6
PLAT713_ALERT_1_C TORSION Unknown or Inconsistent Label ..........         B7 Check
                   B2          B7          B8          B9
PLAT713_ALERT_1_C TORSION Unknown or Inconsistent Label ..........         B7 Check
                   B2          B7          B8          B10
PLAT713_ALERT_1_C TORSION Unknown or Inconsistent Label ..........         B7 Check
                   B2          B7          B10         B8
PLAT713_ALERT_1_C TORSION Unknown or Inconsistent Label ..........         B7 Check
                   B2          B7          B10         B9
PLAT713_ALERT_1_C TORSION Unknown or Inconsistent Label ..........         B6 Check
                   B3          B4          B1          B6
PLAT713_ALERT_1_C TORSION Unknown or Inconsistent Label ..........         B7 Check
                   B3          B4          B1          B7
PLAT713_ALERT_1_C TORSION Unknown or Inconsistent Label ..........         B8 Check
                   B3          B4          B1          B8
PLAT713_ALERT_1_C TORSION Unknown or Inconsistent Label ..........         B7 Check
                   B3          B4          B2          B7
PLAT713_ALERT_1_C TORSION Unknown or Inconsistent Label ..........         B6 Check
                   B3          B4          B6          B8
PLAT713_ALERT_1_C TORSION Unknown or Inconsistent Label ..........         B6 Check
                   B3          B4          B6          B9
PLAT713_ALERT_1_C TORSION Unknown or Inconsistent Label ..........         B7 Check
                   B3          B5          B2          B7
PLAT713_ALERT_1_C TORSION Unknown or Inconsistent Label ..........         B6 Check
                   B3          B5          B4          B6
PLAT713_ALERT_1_C TORSION Unknown or Inconsistent Label ..........         B6 Check
                   B3          B6          B1          B4
PLAT713_ALERT_1_C TORSION Unknown or Inconsistent Label ..........         B6 Check
                   B3          B6          B1          B7
PLAT713_ALERT_1_C TORSION Unknown or Inconsistent Label ..........         B6 Check
                   B3          B6          B1          B8
PLAT713_ALERT_1_C TORSION Unknown or Inconsistent Label ..........         B6 Check
                   B3          B6          B4          B5
PLAT713_ALERT_1_C TORSION Unknown or Inconsistent Label ..........         B6 Check
                   B3          B6          B8          B7
PLAT713_ALERT_1_C TORSION Unknown or Inconsistent Label ..........         B6 Check
                   B3          B6          B8          B9
PLAT713_ALERT_1_C TORSION Unknown or Inconsistent Label ..........         B6 Check
                   B3          B6          B8          B10
PLAT713_ALERT_1_C TORSION Unknown or Inconsistent Label ..........         B6 Check
                   B3          B6          B9          B8
PLAT713_ALERT_1_C TORSION Unknown or Inconsistent Label ..........         B6 Check
                   B3          B6          B9          B10
```

```
PLAT713_ALERT_1_C TORSION Unknown or Inconsistent Label .........          B9 Check
                    B3          B9          B6          B4
PLAT713_ALERT_1_C TORSION Unknown or Inconsistent Label .........          B9 Check
                    B3          B9          B6          B8
PLAT713_ALERT_1_C TORSION Unknown or Inconsistent Label .........          B9 Check
                    B3          B9          B8          B6
PLAT713_ALERT_1_C TORSION Unknown or Inconsistent Label .........          B9 Check
                    B3          B9          B8          B7
PLAT713_ALERT_1_C TORSION Unknown or Inconsistent Label .........          B9 Check
                    B3          B9          B8          B10
PLAT713_ALERT_1_C TORSION Unknown or Inconsistent Label .........          B9 Check
                    B3          B9          B10         B7
PLAT713_ALERT_1_C TORSION Unknown or Inconsistent Label .........          B9 Check
                    B3          B9          B10         B8
PLAT713_ALERT_1_C TORSION Unknown or Inconsistent Label .........          B7 Check
                    B4          B1          B2          B7
PLAT713_ALERT_1_C TORSION Unknown or Inconsistent Label .........          B6 Check
                    B4          B1          B6          B8
PLAT713_ALERT_1_C TORSION Unknown or Inconsistent Label .........          B6 Check
                    B4          B1          B6          B9
PLAT713_ALERT_1_C TORSION Unknown or Inconsistent Label .........          B7 Check
                    B4          B1          B7          B8
PLAT713_ALERT_1_C TORSION Unknown or Inconsistent Label .........          B7 Check
                    B4          B1          B7          B10
PLAT713_ALERT_1_C TORSION Unknown or Inconsistent Label .........          B8 Check
                    B4          B1          B8          B6
PLAT713_ALERT_1_C TORSION Unknown or Inconsistent Label .........          B8 Check
                    B4          B1          B8          B7
PLAT713_ALERT_1_C TORSION Unknown or Inconsistent Label .........          B8 Check
                    B4          B1          B8          B9
PLAT713_ALERT_1_C TORSION Unknown or Inconsistent Label .........          B8 Check
                    B4          B1          B8          B10
PLAT713_ALERT_1_C TORSION Unknown or Inconsistent Label .........          B6 Check
                    B4          B2          B1          B6
PLAT713_ALERT_1_C TORSION Unknown or Inconsistent Label .........          B7 Check
                    B4          B2          B1          B7
PLAT713_ALERT_1_C TORSION Unknown or Inconsistent Label .........          B8 Check
                    B4          B2          B1          B8
PLAT713_ALERT_1_C TORSION Unknown or Inconsistent Label .........          B7 Check
                    B4          B2          B7          B8
PLAT713_ALERT_1_C TORSION Unknown or Inconsistent Label .........          B7 Check
                    B4          B2          B7          B10
PLAT713_ALERT_1_C TORSION Unknown or Inconsistent Label .........          B6 Check
                    B4          B3          B6          B8
PLAT713_ALERT_1_C TORSION Unknown or Inconsistent Label .........          B6 Check
                    B4          B3          B6          B9
PLAT713_ALERT_1_C TORSION Unknown or Inconsistent Label .........          B9 Check
                    B4          B3          B9          B6
PLAT713_ALERT_1_C TORSION Unknown or Inconsistent Label .........          B9 Check
                    B4          B3          B9          B8
PLAT713_ALERT_1_C TORSION Unknown or Inconsistent Label .........          B9 Check
                    B4          B3          B9          B10
PLAT713_ALERT_1_C TORSION Unknown or Inconsistent Label .........          B7 Check
                    B4          B5          B2          B7
PLAT713_ALERT_1_C TORSION Unknown or Inconsistent Label .........          B6 Check
                    B4          B5          B3          B6
PLAT713_ALERT_1_C TORSION Unknown or Inconsistent Label .........          B9 Check
```

```
                    B4          B5          B3          B9
PLAT713_ALERT_1_C TORSION Unknown or Inconsistent Label ..........         B6 Check
                    B4          B6          B1          B7
PLAT713_ALERT_1_C TORSION Unknown or Inconsistent Label ..........         B6 Check
                    B4          B6          B1          B8
PLAT713_ALERT_1_C TORSION Unknown or Inconsistent Label ..........         B6 Check
                    B4          B6          B3          B5
PLAT713_ALERT_1_C TORSION Unknown or Inconsistent Label ..........         B6 Check
                    B4          B6          B3          B9
PLAT713_ALERT_1_C TORSION Unknown or Inconsistent Label ..........         B6 Check
                    B4          B6          B8          B7
PLAT713_ALERT_1_C TORSION Unknown or Inconsistent Label ..........         B6 Check
                    B4          B6          B8          B9
PLAT713_ALERT_1_C TORSION Unknown or Inconsistent Label ..........         B6 Check
                    B4          B6          B8          B10
PLAT713_ALERT_1_C TORSION Unknown or Inconsistent Label ..........         B6 Check
                    B4          B6          B9          B8
PLAT713_ALERT_1_C TORSION Unknown or Inconsistent Label ..........         B6 Check
                    B4          B6          B9          B10
PLAT713_ALERT_1_C TORSION Unknown or Inconsistent Label ..........         B6 Check
                    B5          B2          B1          B6
PLAT713_ALERT_1_C TORSION Unknown or Inconsistent Label ..........         B7 Check
                    B5          B2          B1          B7
PLAT713_ALERT_1_C TORSION Unknown or Inconsistent Label ..........         B8 Check
                    B5          B2          B1          B8
PLAT713_ALERT_1_C TORSION Unknown or Inconsistent Label ..........         B6 Check
                    B5          B2          B4          B6
PLAT713_ALERT_1_C TORSION Unknown or Inconsistent Label ..........         B7 Check
                    B5          B2          B7          B8
PLAT713_ALERT_1_C TORSION Unknown or Inconsistent Label ..........         B7 Check
                    B5          B2          B7          B10
PLAT713_ALERT_1_C TORSION Unknown or Inconsistent Label ..........         B6 Check
                    B5          B3          B4          B6
PLAT713_ALERT_1_C TORSION Unknown or Inconsistent Label ..........         B6 Check
                    B5          B3          B6          B8
PLAT713_ALERT_1_C TORSION Unknown or Inconsistent Label ..........         B6 Check
                    B5          B3          B6          B9
PLAT713_ALERT_1_C TORSION Unknown or Inconsistent Label ..........         B9 Check
                    B5          B3          B9          B6
PLAT713_ALERT_1_C TORSION Unknown or Inconsistent Label ..........         B9 Check
                    B5          B3          B9          B8
PLAT713_ALERT_1_C TORSION Unknown or Inconsistent Label ..........         B9 Check
                    B5          B3          B9          B10
PLAT713_ALERT_1_C TORSION Unknown or Inconsistent Label ..........         B6 Check
                    B5          B4          B1          B6
PLAT713_ALERT_1_C TORSION Unknown or Inconsistent Label ..........         B7 Check
                    B5          B4          B1          B7
PLAT713_ALERT_1_C TORSION Unknown or Inconsistent Label ..........         B8 Check
                    B5          B4          B1          B8
PLAT713_ALERT_1_C TORSION Unknown or Inconsistent Label ..........         B7 Check
                    B5          B4          B2          B7
PLAT713_ALERT_1_C TORSION Unknown or Inconsistent Label ..........         B6 Check
                    B5          B4          B3          B6
PLAT713_ALERT_1_C TORSION Unknown or Inconsistent Label ..........         B9 Check
                    B5          B4          B3          B9
PLAT713_ALERT_1_C TORSION Unknown or Inconsistent Label ..........         B6 Check
                    B5          B4          B6          B8
```

```
PLAT713_ALERT_1_C TORSION Unknown or Inconsistent Label .........          B6 Check
                    B5          B4          B6          B9
PLAT713_ALERT_1_C TORSION Unknown or Inconsistent Label .........          B6 Check
                    B6          B1          B2          B7
PLAT713_ALERT_1_C TORSION Unknown or Inconsistent Label .........          B6 Check
                    B6          B1          B7          B8
PLAT713_ALERT_1_C TORSION Unknown or Inconsistent Label .........          B6 Check
                    B6          B1          B7          B10
PLAT713_ALERT_1_C TORSION Unknown or Inconsistent Label .........          B6 Check
                    B6          B1          B8          B7
PLAT713_ALERT_1_C TORSION Unknown or Inconsistent Label .........          B6 Check
                    B6          B1          B8          B9
PLAT713_ALERT_1_C TORSION Unknown or Inconsistent Label .........          B6 Check
                    B6          B1          B8          B10
PLAT713_ALERT_1_C TORSION Unknown or Inconsistent Label .........          B6 Check
                    B6          B3          B9          B8
PLAT713_ALERT_1_C TORSION Unknown or Inconsistent Label .........          B6 Check
                    B6          B3          B9          B10
PLAT713_ALERT_1_C TORSION Unknown or Inconsistent Label .........          B6 Check
                    B6          B4          B1          B7
PLAT713_ALERT_1_C TORSION Unknown or Inconsistent Label .........          B6 Check
                    B6          B4          B1          B8
PLAT713_ALERT_1_C TORSION Unknown or Inconsistent Label .........          B6 Check
                    B6          B4          B2          B7
PLAT713_ALERT_1_C TORSION Unknown or Inconsistent Label .........          B6 Check
                    B6          B4          B3          B9
PLAT713_ALERT_1_C TORSION Unknown or Inconsistent Label .........          B6 Check
                    B6          B8          B1          B7
PLAT713_ALERT_1_C TORSION Unknown or Inconsistent Label .........          B6 Check
                    B6          B8          B7          B10
PLAT713_ALERT_1_C TORSION Unknown or Inconsistent Label .........          B6 Check
                    B6          B8          B9          B10
PLAT713_ALERT_1_C TORSION Unknown or Inconsistent Label .........          B6 Check
                    B6          B8          B10         B7
PLAT713_ALERT_1_C TORSION Unknown or Inconsistent Label .........          B6 Check
                    B6          B8          B10         B9
PLAT713_ALERT_1_C TORSION Unknown or Inconsistent Label .........          B6 Check
                    B6          B9          B8          B7
PLAT713_ALERT_1_C TORSION Unknown or Inconsistent Label .........          B6 Check
                    B6          B9          B8          B10
PLAT713_ALERT_1_C TORSION Unknown or Inconsistent Label .........          B6 Check
                    B6          B9          B10         B7
PLAT713_ALERT_1_C TORSION Unknown or Inconsistent Label .........          B6 Check
                    B6          B9          B10         B8
PLAT713_ALERT_1_C TORSION Unknown or Inconsistent Label .........          B7 Check
                    B7          B1          B6          B8
PLAT713_ALERT_1_C TORSION Unknown or Inconsistent Label .........          B7 Check
                    B7          B1          B6          B9
PLAT713_ALERT_1_C TORSION Unknown or Inconsistent Label .........          B7 Check
                    B7          B1          B8          B9
PLAT713_ALERT_1_C TORSION Unknown or Inconsistent Label .........          B7 Check
                    B7          B1          B8          B10
PLAT713_ALERT_1_C TORSION Unknown or Inconsistent Label .........          B7 Check
                    B7          B2          B1          B8
PLAT713_ALERT_1_C TORSION Unknown or Inconsistent Label .........          B7 Check
                    B7          B8          B6          B9
PLAT713_ALERT_1_C TORSION Unknown or Inconsistent Label .........          B7 Check
```

```
                    B7          B8          B9          B10
PLAT713_ALERT_1_C TORSION Unknown or Inconsistent Label ..........         B7 Check
                    B7          B8          B10         B9
PLAT713_ALERT_1_C TORSION Unknown or Inconsistent Label ..........         B7 Check
                    B7          B10         B8          B9
PLAT713_ALERT_1_C TORSION Unknown or Inconsistent Label ..........         B7 Check
                    B7          B10         B9          B8
PLAT713_ALERT_1_C TORSION Unknown or Inconsistent Label ..........         B8 Check
                    B8          B1          B6          B9
PLAT713_ALERT_1_C TORSION Unknown or Inconsistent Label ..........         B8 Check
                    B8          B1          B7          B10
PLAT713_ALERT_1_C TORSION Unknown or Inconsistent Label ..........         B8 Check
                    B8          B6          B3          B9
PLAT713_ALERT_1_C TORSION Unknown or Inconsistent Label ..........         B8 Check
                    B8          B6          B9          B10
PLAT713_ALERT_1_C TORSION Unknown or Inconsistent Label ..........         B8 Check
                    B8          B7          B10         B9
PLAT713_ALERT_1_C TORSION Unknown or Inconsistent Label ..........         B9 Check
                    B9          B6          B8          B10
PLAT713_ALERT_1_C TORSION Unknown or Inconsistent Label ..........         B9 Check
                    B9          B8          B7          B10
PLAT713_ALERT_1_C TORSION Unknown or Inconsistent Label ..........         B7 Check
                    B1          B2          B7          H10
PLAT713_ALERT_1_C TORSION Unknown or Inconsistent Label ..........         B7 Check
                    B1          B2          B7          H12
PLAT713_ALERT_1_C TORSION Unknown or Inconsistent Label ..........        H10 Check
                    B1          B4          B2          H10
PLAT713_ALERT_1_C TORSION Unknown or Inconsistent Label ..........         B6 Check
                    B1          B4          B6          H11
PLAT713_ALERT_1_C TORSION Unknown or Inconsistent Label ..........         B6 Check
                    B1          B6          B3          H4
PLAT713_ALERT_1_C TORSION Unknown or Inconsistent Label ..........         B6 Check
                    B1          B6          B3          H9
PLAT713_ALERT_1_C TORSION Unknown or Inconsistent Label ..........         B6 Check
                    B1          B6          B4          H2
PLAT713_ALERT_1_C TORSION Unknown or Inconsistent Label ..........         B6 Check
                    B1          B6          B8          H13
PLAT713_ALERT_1_C TORSION Unknown or Inconsistent Label ..........         B6 Check
                    B1          B6          B9          H9
PLAT713_ALERT_1_C TORSION Unknown or Inconsistent Label ..........         B6 Check
                    B1          B6          B9          H14
PLAT713_ALERT_1_C TORSION Unknown or Inconsistent Label ..........         B7 Check
                    B1          B7          B2          H3
PLAT713_ALERT_1_C TORSION Unknown or Inconsistent Label ..........         B7 Check
                    B1          B7          B2          H10
PLAT713_ALERT_1_C TORSION Unknown or Inconsistent Label ..........         B7 Check
                    B1          B7          B8          H13
PLAT713_ALERT_1_C TORSION Unknown or Inconsistent Label ..........         B7 Check
                    B1          B7          B10         H15
PLAT713_ALERT_1_C TORSION Unknown or Inconsistent Label ..........         B8 Check
                    B1          B8          B6          H11
PLAT713_ALERT_1_C TORSION Unknown or Inconsistent Label ..........         B8 Check
                    B1          B8          B7          H10
PLAT713_ALERT_1_C TORSION Unknown or Inconsistent Label ..........         B8 Check
                    B1          B8          B7          H12
PLAT713_ALERT_1_C TORSION Unknown or Inconsistent Label ..........         B8 Check
                    B1          B8          B9          H9
```

```
PLAT713_ALERT_1_C TORSION Unknown or Inconsistent Label ..........         B8 Check
                   B1          B8          B9          H14
PLAT713_ALERT_1_C TORSION Unknown or Inconsistent Label ..........         B8 Check
                   B1          B8          B10         H15
PLAT713_ALERT_1_C TORSION Unknown or Inconsistent Label ..........         B6 Check
                   B2          B1          B6          H11
PLAT713_ALERT_1_C TORSION Unknown or Inconsistent Label ..........         B7 Check
                   B2          B1          B7          H10
PLAT713_ALERT_1_C TORSION Unknown or Inconsistent Label ..........         B7 Check
                   B2          B1          B7          H12
PLAT713_ALERT_1_C TORSION Unknown or Inconsistent Label ..........         B8 Check
                   B2          B1          B8          H13
PLAT713_ALERT_1_C TORSION Unknown or Inconsistent Label ..........         B6 Check
                   B2          B4          B6          H11
PLAT713_ALERT_1_C TORSION Unknown or Inconsistent Label ..........         B7 Check
                   B2          B7          B1          H1
PLAT713_ALERT_1_C TORSION Unknown or Inconsistent Label ..........         B7 Check
                   B2          B7          B8          H13
PLAT713_ALERT_1_C TORSION Unknown or Inconsistent Label ..........         B7 Check
                   B2          B7          B10         H15
PLAT713_ALERT_1_C TORSION Unknown or Inconsistent Label ..........        H10 Check
                   B3          B4          B2          H10
PLAT713_ALERT_1_C TORSION Unknown or Inconsistent Label ..........         B6 Check
                   B3          B4          B6          H11
PLAT713_ALERT_1_C TORSION Unknown or Inconsistent Label ..........        H10 Check
                   B3          B5          B2          H10
PLAT713_ALERT_1_C TORSION Unknown or Inconsistent Label ..........         B6 Check
                   B3          B6          B1          H1
PLAT713_ALERT_1_C TORSION Unknown or Inconsistent Label ..........         B6 Check
                   B3          B6          B4          H2
PLAT713_ALERT_1_C TORSION Unknown or Inconsistent Label ..........         B6 Check
                   B3          B6          B8          H13
PLAT713_ALERT_1_C TORSION Unknown or Inconsistent Label ..........         B6 Check
                   B3          B6          B9          H9
PLAT713_ALERT_1_C TORSION Unknown or Inconsistent Label ..........         B6 Check
                   B3          B6          B9          H14
PLAT713_ALERT_1_C TORSION Unknown or Inconsistent Label ..........         B9 Check
                   B3          B9          B6          H11
PLAT713_ALERT_1_C TORSION Unknown or Inconsistent Label ..........         B9 Check
                   B3          B9          B8          H13
PLAT713_ALERT_1_C TORSION Unknown or Inconsistent Label ..........         B9 Check
                   B3          B9          B10         H15
PLAT713_ALERT_1_C TORSION Unknown or Inconsistent Label ..........        H10 Check
                   B4          B1          B2          H10
PLAT713_ALERT_1_C TORSION Unknown or Inconsistent Label ..........         B6 Check
                   B4          B1          B6          H11
PLAT713_ALERT_1_C TORSION Unknown or Inconsistent Label ..........         B7 Check
                   B4          B1          B7          H10
PLAT713_ALERT_1_C TORSION Unknown or Inconsistent Label ..........         B7 Check
                   B4          B1          B7          H12
PLAT713_ALERT_1_C TORSION Unknown or Inconsistent Label ..........         B8 Check
                   B4          B1          B8          H13
PLAT713_ALERT_1_C TORSION Unknown or Inconsistent Label ..........         B7 Check
                   B4          B2          B7          H10
PLAT713_ALERT_1_C TORSION Unknown or Inconsistent Label ..........         B7 Check
                   B4          B2          B7          H12
PLAT713_ALERT_1_C TORSION Unknown or Inconsistent Label ..........         B6 Check
```

```
                    B4          B3          B6          H11
PLAT713_ALERT_1_C TORSION Unknown or Inconsistent Label ..........         B9 Check
                    B4          B3          B9          H9
PLAT713_ALERT_1_C TORSION Unknown or Inconsistent Label ..........         B9 Check
                    B4          B3          B9          H14
PLAT713_ALERT_1_C TORSION Unknown or Inconsistent Label ..........        H10 Check
                    B4          B5          B2          H10
PLAT713_ALERT_1_C TORSION Unknown or Inconsistent Label ..........         B6 Check
                    B4          B6          B1          H1
PLAT713_ALERT_1_C TORSION Unknown or Inconsistent Label ..........         B6 Check
                    B4          B6          B3          H4
PLAT713_ALERT_1_C TORSION Unknown or Inconsistent Label ..........         B6 Check
                    B4          B6          B3          H9
PLAT713_ALERT_1_C TORSION Unknown or Inconsistent Label ..........         B6 Check
                    B4          B6          B8          H13
PLAT713_ALERT_1_C TORSION Unknown or Inconsistent Label ..........         B6 Check
                    B4          B6          B9          H9
PLAT713_ALERT_1_C TORSION Unknown or Inconsistent Label ..........         B6 Check
                    B4          B6          B9          H14
PLAT713_ALERT_1_C TORSION Unknown or Inconsistent Label ..........         B7 Check
                    B5          B2          B7          H10
PLAT713_ALERT_1_C TORSION Unknown or Inconsistent Label ..........         B7 Check
                    B5          B2          B7          H12
PLAT713_ALERT_1_C TORSION Unknown or Inconsistent Label ..........         B6 Check
                    B5          B3          B6          H11
PLAT713_ALERT_1_C TORSION Unknown or Inconsistent Label ..........         B9 Check
                    B5          B3          B9          H9
PLAT713_ALERT_1_C TORSION Unknown or Inconsistent Label ..........         B9 Check
                    B5          B3          B9          H14
PLAT713_ALERT_1_C TORSION Unknown or Inconsistent Label ..........        H10 Check
                    B5          B4          B2          H10
PLAT713_ALERT_1_C TORSION Unknown or Inconsistent Label ..........         B6 Check
                    B5          B4          B6          H11
PLAT713_ALERT_1_C TORSION Unknown or Inconsistent Label ..........         B6 Check
                    B6          B1          B2          H10
PLAT713_ALERT_1_C TORSION Unknown or Inconsistent Label ..........         B6 Check
                    B6          B1          B7          H10
PLAT713_ALERT_1_C TORSION Unknown or Inconsistent Label ..........         B6 Check
                    B6          B1          B7          H12
PLAT713_ALERT_1_C TORSION Unknown or Inconsistent Label ..........         B6 Check
                    B6          B1          B8          H13
PLAT713_ALERT_1_C TORSION Unknown or Inconsistent Label ..........         B6 Check
                    B6          B3          B9          H14
PLAT713_ALERT_1_C TORSION Unknown or Inconsistent Label ..........         B6 Check
                    B6          B4          B2          H10
PLAT713_ALERT_1_C TORSION Unknown or Inconsistent Label ..........         B6 Check
                    B6          B8          B7          H10
PLAT713_ALERT_1_C TORSION Unknown or Inconsistent Label ..........         B6 Check
                    B6          B8          B7          H12
PLAT713_ALERT_1_C TORSION Unknown or Inconsistent Label ..........         B6 Check
                    B6          B8          B9          H14
PLAT713_ALERT_1_C TORSION Unknown or Inconsistent Label ..........         B6 Check
                    B6          B8          B10         H15
PLAT713_ALERT_1_C TORSION Unknown or Inconsistent Label ..........         B6 Check
                    B6          B9          B8          H13
PLAT713_ALERT_1_C TORSION Unknown or Inconsistent Label ..........         B6 Check
                    B6          B9          B10         H15
```

```
PLAT713_ALERT_1_C TORSION Unknown or Inconsistent Label ..........         B7 Check
                    B7          B1          B2          H10
PLAT713_ALERT_1_C TORSION Unknown or Inconsistent Label ..........         B7 Check
                    B7          B1          B6          H11
PLAT713_ALERT_1_C TORSION Unknown or Inconsistent Label ..........         B7 Check
                    B7          B1          B8          H13
PLAT713_ALERT_1_C TORSION Unknown or Inconsistent Label ..........         B7 Check
                    B7          B8          B6          H11
PLAT713_ALERT_1_C TORSION Unknown or Inconsistent Label ..........         B7 Check
                    B7          B8          B9          H14
PLAT713_ALERT_1_C TORSION Unknown or Inconsistent Label ..........         B7 Check
                    B7          B8          B10         H15
PLAT713_ALERT_1_C TORSION Unknown or Inconsistent Label ..........         B7 Check
                    B7          B10         B8          H13
PLAT713_ALERT_1_C TORSION Unknown or Inconsistent Label ..........         B7 Check
                    B7          B10         B9          H14
PLAT713_ALERT_1_C TORSION Unknown or Inconsistent Label ..........         B8 Check
                    B8          B1          B2          H10
PLAT713_ALERT_1_C TORSION Unknown or Inconsistent Label ..........         B8 Check
                    B8          B1          B6          H11
PLAT713_ALERT_1_C TORSION Unknown or Inconsistent Label ..........         B8 Check
                    B8          B1          B7          H10
PLAT713_ALERT_1_C TORSION Unknown or Inconsistent Label ..........         B8 Check
                    B8          B1          B7          H12
PLAT713_ALERT_1_C TORSION Unknown or Inconsistent Label ..........         B8 Check
                    B8          B6          B9          H14
PLAT713_ALERT_1_C TORSION Unknown or Inconsistent Label ..........         B8 Check
                    B8          B7          B2          H10
PLAT713_ALERT_1_C TORSION Unknown or Inconsistent Label ..........         B8 Check
                    B8          B7          B10         H15
PLAT713_ALERT_1_C TORSION Unknown or Inconsistent Label ..........         B8 Check
                    B8          B9          B6          H11
PLAT713_ALERT_1_C TORSION Unknown or Inconsistent Label ..........         B8 Check
                    B8          B9          B10         H15
PLAT713_ALERT_1_C TORSION Unknown or Inconsistent Label ..........         B8 Check
                    B8          B10         B7          H10
PLAT713_ALERT_1_C TORSION Unknown or Inconsistent Label ..........         B8 Check
                    B8          B10         B7          H12
PLAT713_ALERT_1_C TORSION Unknown or Inconsistent Label ..........         B8 Check
                    B8          B10         B9          H14
PLAT713_ALERT_1_C TORSION Unknown or Inconsistent Label ..........         B9 Check
                    B9          B3          B6          H11
PLAT713_ALERT_1_C TORSION Unknown or Inconsistent Label ..........         B9 Check
                    B9          B6          B8          H13
PLAT713_ALERT_1_C TORSION Unknown or Inconsistent Label ..........         B9 Check
                    B9          B8          B6          H11
PLAT713_ALERT_1_C TORSION Unknown or Inconsistent Label ..........         B9 Check
                    B9          B8          B7          H12
PLAT713_ALERT_1_C TORSION Unknown or Inconsistent Label ..........         B9 Check
                    B9          B8          B10         H15
PLAT713_ALERT_1_C TORSION Unknown or Inconsistent Label ..........         B9 Check
                    B9          B10         B7          H12
PLAT713_ALERT_1_C TORSION Unknown or Inconsistent Label ..........         B9 Check
                    B9          B10         B8          H13
PLAT713_ALERT_1_C TORSION Unknown or Inconsistent Label ..........        B10 Check
                    B10         B7          B8          H13
PLAT713_ALERT_1_C TORSION Unknown or Inconsistent Label ..........        B10 Check
```

```
                    B10         B8          B7          H12
PLAT713_ALERT_1_C TORSION Unknown or Inconsistent Label ..........        B10 Check
                    B10         B8          B9          H14
PLAT713_ALERT_1_C TORSION Unknown or Inconsistent Label ..........        B10 Check
                    B10         B9          B8          H13
PLAT713_ALERT_1_C TORSION Unknown or Inconsistent Label ..........        H10 Check
                    B1          B2          H10         B7
PLAT713_ALERT_1_C TORSION Unknown or Inconsistent Label ..........         B7 Check
                    B1          B7          H10         B2
PLAT713_ALERT_1_C TORSION Unknown or Inconsistent Label ..........        H10 Check
                    B4          B2          H10         B7
PLAT713_ALERT_1_C TORSION Unknown or Inconsistent Label ..........         B9 Check
                    B4          B3          H9          B9
PLAT713_ALERT_1_C TORSION Unknown or Inconsistent Label ..........        H10 Check
                    B5          B2          H10         B7
PLAT713_ALERT_1_C TORSION Unknown or Inconsistent Label ..........         B9 Check
                    B5          B3          H9          B9
PLAT713_ALERT_1_C TORSION Unknown or Inconsistent Label ..........         B6 Check
                    B6          B3          H9          B9
PLAT713_ALERT_1_C TORSION Unknown or Inconsistent Label ..........        H10 Check
                    B2          H10         B7          B8
PLAT713_ALERT_1_C TORSION Unknown or Inconsistent Label ..........        H10 Check
                    B2          H10         B7          B10
PLAT713_ALERT_1_C TORSION Unknown or Inconsistent Label ..........         B9 Check
                    B3          H9          B9          B6
PLAT713_ALERT_1_C TORSION Unknown or Inconsistent Label ..........         B9 Check
                    B3          H9          B9          B8
PLAT713_ALERT_1_C TORSION Unknown or Inconsistent Label ..........         B9 Check
                    B3          H9          B9          B10
PLAT713_ALERT_1_C TORSION Unknown or Inconsistent Label ..........        H10 Check
                    B2          H10         B7          H12
PLAT713_ALERT_1_C TORSION Unknown or Inconsistent Label ..........         B9 Check
                    B3          H9          B9          H14
PLAT713_ALERT_1_C TORSION Unknown or Inconsistent Label ..........        H15 Check
                    H15         B10         N2          C3
PLAT713_ALERT_1_C TORSION Unknown or Inconsistent Label ..........         B9 Check
                    H9          B9          B10         N2
PLAT713_ALERT_1_C TORSION Unknown or Inconsistent Label ..........        H10 Check
                    H10         B7          B10         N2
PLAT713_ALERT_1_C TORSION Unknown or Inconsistent Label ..........        H12 Check
                    H12         B7          B10         N2
PLAT713_ALERT_1_C TORSION Unknown or Inconsistent Label ..........        H13 Check
                    H13         B8          B10         N2
PLAT713_ALERT_1_C TORSION Unknown or Inconsistent Label ..........        H14 Check
                    H14         B9          B10         N2
PLAT713_ALERT_1_C TORSION Unknown or Inconsistent Label ..........         B7 Check
                    H1          B1          B2          B7
PLAT713_ALERT_1_C TORSION Unknown or Inconsistent Label ..........         B6 Check
                    H1          B1          B4          B6
PLAT713_ALERT_1_C TORSION Unknown or Inconsistent Label ..........         B6 Check
                    H1          B1          B6          B8
PLAT713_ALERT_1_C TORSION Unknown or Inconsistent Label ..........         B6 Check
                    H1          B1          B6          B9
PLAT713_ALERT_1_C TORSION Unknown or Inconsistent Label ..........         B7 Check
                    H1          B1          B7          B8
PLAT713_ALERT_1_C TORSION Unknown or Inconsistent Label ..........         B7 Check
                    H1          B1          B7          B10
```

```
PLAT713_ALERT_1_C TORSION Unknown or Inconsistent Label ..........         B8 Check
                    H1          B1          B8          B6
PLAT713_ALERT_1_C TORSION Unknown or Inconsistent Label ..........         B8 Check
                    H1          B1          B8          B7
PLAT713_ALERT_1_C TORSION Unknown or Inconsistent Label ..........         B8 Check
                    H1          B1          B8          B9
PLAT713_ALERT_1_C TORSION Unknown or Inconsistent Label ..........         B8 Check
                    H1          B1          B8          B10
PLAT713_ALERT_1_C TORSION Unknown or Inconsistent Label ..........         B6 Check
                    H2          B4          B1          B6
PLAT713_ALERT_1_C TORSION Unknown or Inconsistent Label ..........         B7 Check
                    H2          B4          B1          B7
PLAT713_ALERT_1_C TORSION Unknown or Inconsistent Label ..........         B8 Check
                    H2          B4          B1          B8
PLAT713_ALERT_1_C TORSION Unknown or Inconsistent Label ..........         B7 Check
                    H2          B4          B2          B7
PLAT713_ALERT_1_C TORSION Unknown or Inconsistent Label ..........         B6 Check
                    H2          B4          B3          B6
PLAT713_ALERT_1_C TORSION Unknown or Inconsistent Label ..........         B9 Check
                    H2          B4          B3          B9
PLAT713_ALERT_1_C TORSION Unknown or Inconsistent Label ..........         B6 Check
                    H2          B4          B6          B8
PLAT713_ALERT_1_C TORSION Unknown or Inconsistent Label ..........         B6 Check
                    H2          B4          B6          B9
PLAT713_ALERT_1_C TORSION Unknown or Inconsistent Label ..........         B6 Check
                    H3          B2          B1          B6
PLAT713_ALERT_1_C TORSION Unknown or Inconsistent Label ..........         B7 Check
                    H3          B2          B1          B7
PLAT713_ALERT_1_C TORSION Unknown or Inconsistent Label ..........         B8 Check
                    H3          B2          B1          B8
PLAT713_ALERT_1_C TORSION Unknown or Inconsistent Label ..........         B6 Check
                    H3          B2          B4          B6
PLAT713_ALERT_1_C TORSION Unknown or Inconsistent Label ..........         B7 Check
                    H3          B2          B7          B8
PLAT713_ALERT_1_C TORSION Unknown or Inconsistent Label ..........         B7 Check
                    H3          B2          B7          B10
PLAT713_ALERT_1_C TORSION Unknown or Inconsistent Label ..........         B6 Check
                    H4          B3          B4          B6
PLAT713_ALERT_1_C TORSION Unknown or Inconsistent Label ..........         B6 Check
                    H4          B3          B6          B8
PLAT713_ALERT_1_C TORSION Unknown or Inconsistent Label ..........         B6 Check
                    H4          B3          B6          B9
PLAT713_ALERT_1_C TORSION Unknown or Inconsistent Label ..........         B9 Check
                    H4          B3          B9          B6
PLAT713_ALERT_1_C TORSION Unknown or Inconsistent Label ..........         B9 Check
                    H4          B3          B9          B8
PLAT713_ALERT_1_C TORSION Unknown or Inconsistent Label ..........         B9 Check
                    H4          B3          B9          B10
PLAT713_ALERT_1_C TORSION Unknown or Inconsistent Label ..........         B7 Check
                    H8          B5          B2          B7
PLAT713_ALERT_1_C TORSION Unknown or Inconsistent Label ..........         B6 Check
                    H8          B5          B3          B6
PLAT713_ALERT_1_C TORSION Unknown or Inconsistent Label ..........         B9 Check
                    H8          B5          B3          B9
PLAT713_ALERT_1_C TORSION Unknown or Inconsistent Label ..........         B6 Check
                    H8          B5          B4          B6
PLAT713_ALERT_1_C TORSION Unknown or Inconsistent Label ..........         B6 Check
```

```
                    H9          B3          B4          B6
PLAT713_ALERT_1_C TORSION Unknown or Inconsistent Label ..........         B6 Check
                    H9          B3          B6          B8
PLAT713_ALERT_1_C TORSION Unknown or Inconsistent Label ..........         B6 Check
                    H9          B3          B6          B9
PLAT713_ALERT_1_C TORSION Unknown or Inconsistent Label ..........         B9 Check
                    H9          B3          B9          B6
PLAT713_ALERT_1_C TORSION Unknown or Inconsistent Label ..........         B9 Check
                    H9          B3          B9          B8
PLAT713_ALERT_1_C TORSION Unknown or Inconsistent Label ..........         B9 Check
                    H9          B3          B9          B10
PLAT713_ALERT_1_C TORSION Unknown or Inconsistent Label ..........         B9 Check
                    H9          B9          B3          B6
PLAT713_ALERT_1_C TORSION Unknown or Inconsistent Label ..........         B9 Check
                    H9          B9          B6          B8
PLAT713_ALERT_1_C TORSION Unknown or Inconsistent Label ..........         B9 Check
                    H9          B9          B8          B6
PLAT713_ALERT_1_C TORSION Unknown or Inconsistent Label ..........         B9 Check
                    H9          B9          B8          B7
PLAT713_ALERT_1_C TORSION Unknown or Inconsistent Label ..........         B9 Check
                    H9          B9          B8          B10
PLAT713_ALERT_1_C TORSION Unknown or Inconsistent Label ..........         B9 Check
                    H9          B9          B10         B7
PLAT713_ALERT_1_C TORSION Unknown or Inconsistent Label ..........         B9 Check
                    H9          B9          B10         B8
PLAT713_ALERT_1_C TORSION Unknown or Inconsistent Label ..........        H10 Check
                    H10         B2          B7          B10
PLAT713_ALERT_1_C TORSION Unknown or Inconsistent Label ..........        H10 Check
                    H10         B7          B8          B9
PLAT713_ALERT_1_C TORSION Unknown or Inconsistent Label ..........        H10 Check
                    H10         B7          B8          B10
PLAT713_ALERT_1_C TORSION Unknown or Inconsistent Label ..........        H10 Check
                    H10         B7          B10         B9
PLAT713_ALERT_1_C TORSION Unknown or Inconsistent Label ..........        H11 Check
                    H11         B6          B8          B10
PLAT713_ALERT_1_C TORSION Unknown or Inconsistent Label ..........        H11 Check
                    H11         B6          B9          B10
PLAT713_ALERT_1_C TORSION Unknown or Inconsistent Label ..........        H10 Check
                    H1          B1          B2          H10
PLAT713_ALERT_1_C TORSION Unknown or Inconsistent Label ..........         B6 Check
                    H1          B1          B6          H11
PLAT713_ALERT_1_C TORSION Unknown or Inconsistent Label ..........         B7 Check
                    H1          B1          B7          H10
PLAT713_ALERT_1_C TORSION Unknown or Inconsistent Label ..........         B7 Check
                    H1          B1          B7          H12
PLAT713_ALERT_1_C TORSION Unknown or Inconsistent Label ..........         B8 Check
                    H1          B1          B8          H13
PLAT713_ALERT_1_C TORSION Unknown or Inconsistent Label ..........        H10 Check
                    H2          B4          B2          H10
PLAT713_ALERT_1_C TORSION Unknown or Inconsistent Label ..........         B6 Check
                    H2          B4          B6          H11
PLAT713_ALERT_1_C TORSION Unknown or Inconsistent Label ..........         B7 Check
                    H3          B2          B7          H10
PLAT713_ALERT_1_C TORSION Unknown or Inconsistent Label ..........         B7 Check
                    H3          B2          B7          H12
PLAT713_ALERT_1_C TORSION Unknown or Inconsistent Label ..........         B6 Check
                    H4          B3          B6          H11
```

```
PLAT713_ALERT_1_C TORSION Unknown or Inconsistent Label ..........          B9 Check
                    H4          B3          B9          H9
PLAT713_ALERT_1_C TORSION Unknown or Inconsistent Label ..........          B9 Check
                    H4          B3          B9          H14
PLAT713_ALERT_1_C TORSION Unknown or Inconsistent Label ..........         H10 Check
                    H8          B5          B2          H10
PLAT713_ALERT_1_C TORSION Unknown or Inconsistent Label ..........          B6 Check
                    H9          B3          B6          H11
PLAT713_ALERT_1_C TORSION Unknown or Inconsistent Label ..........          B9 Check
                    H9          B3          B9          H14
PLAT713_ALERT_1_C TORSION Unknown or Inconsistent Label ..........          B9 Check
                    H9          B9          B6          H11
PLAT713_ALERT_1_C TORSION Unknown or Inconsistent Label ..........          B9 Check
                    H9          B9          B8          H13
PLAT713_ALERT_1_C TORSION Unknown or Inconsistent Label ..........          B9 Check
                    H9          B9          B10         H15
PLAT713_ALERT_1_C TORSION Unknown or Inconsistent Label ..........         H10 Check
                    H10         B2          B7          H12
PLAT713_ALERT_1_C TORSION Unknown or Inconsistent Label ..........         H10 Check
                    H10         B7          B8          H13
PLAT713_ALERT_1_C TORSION Unknown or Inconsistent Label ..........         H10 Check
                    H10         B7          B10         H15
PLAT713_ALERT_1_C TORSION Unknown or Inconsistent Label ..........         H11 Check
                    H11         B6          B8          H13
PLAT713_ALERT_1_C TORSION Unknown or Inconsistent Label ..........         H11 Check
                    H11         B6          B9          H14
PLAT713_ALERT_1_C TORSION Unknown or Inconsistent Label ..........         H12 Check
                    H12         B7          B8          H13
PLAT713_ALERT_1_C TORSION Unknown or Inconsistent Label ..........         H12 Check
                    H12         B7          B10         H15
PLAT713_ALERT_1_C TORSION Unknown or Inconsistent Label ..........         H13 Check
                    H13         B8          B9          H14
PLAT713_ALERT_1_C TORSION Unknown or Inconsistent Label ..........         H13 Check
                    H13         B8          B10         H15
PLAT713_ALERT_1_C TORSION Unknown or Inconsistent Label ..........         H14 Check
                    H14         B9          B10         H15
PLAT713_ALERT_1_C TORSION Unknown or Inconsistent Label ..........         H10 Check
                    H3          B2          H10         B7
PLAT713_ALERT_1_C TORSION Unknown or Inconsistent Label ..........          B9 Check
                    H4          B3          H9          B9
```

Alert level G

```
PLAT005_ALERT_5_G No Embedded Refinement Details Found  in the CIF     Please Do !
PLAT128_ALERT_4_G Alternate Setting for Input Space Group     C2/c       I2/a Note
PLAT710_ALERT_4_G Delete 1-2-3 or 2-3-4 Linear Torsion Angle ... #          1 Do !
              N1  -C1  -C2  -H5      3.90  1.40   1_555   1_555   1_555   1_555
PLAT710_ALERT_4_G Delete 1-2-3 or 2-3-4 Linear Torsion Angle ... #          2 Do !
              N1  -C1  -C2  -H6   -116.10  1.30   1_555   1_555   1_555   1_555
PLAT710_ALERT_4_G Delete 1-2-3 or 2-3-4 Linear Torsion Angle ... #          3 Do !
              N1  -C1  -C2  -H7    123.70  1.40   1_555   1_555   1_555   1_555
PLAT710_ALERT_4_G Delete 1-2-3 or 2-3-4 Linear Torsion Angle ... #         22 Do !
              C1  -N1  -B5  -B2     96.50  1.90   1_555   1_555   1_555   1_555
PLAT710_ALERT_4_G Delete 1-2-3 or 2-3-4 Linear Torsion Angle ... #         23 Do !
              C1  -N1  -B5  -B3    -29.10  1.90   1_555   1_555   1_555   1_555
PLAT710_ALERT_4_G Delete 1-2-3 or 2-3-4 Linear Torsion Angle ... #         24 Do !
              C1  -N1  -B5  -B4     33.80  1.90   1_555   1_555   1_555   1_555
```

```
PLAT710_ALERT_4_G Delete 1-2-3 or 2-3-4 Linear Torsion Angle ... #         25 Do !
              C1  -N1  -B5  -H8   -147.10  1.90   1_555   1_555   1_555   1_555
PLAT710_ALERT_4_G Delete 1-2-3 or 2-3-4 Linear Torsion Angle ... #         26 Do !
              C2  -C1  -N1  -B5     18.00  3.00   1_555   1_555   1_555   1_555
PLAT808_ALERT_5_G No Parseable SHELXL Style Weighting Scheme Found     Please Check
PLAT883_ALERT_1_G Absent Datum for _atom_sites_solution_primary ..     Please Do !
PLAT910_ALERT_3_G Missing FCF Reflection(s) Below Theta(Min)[Deg]=       3.22 Note
                1  1  0,   2  0  0,
PLAT911_ALERT_3_G Missing FCF Refl Between Thmin & STh/L=    0.600        174 Report
                2 12  0,   4  0  0,   4  2  0,   4 10  0,   5  3  0,   5  5  0,
                6  0  0,   6  4  0,   6 12  0,   7  3  0,   7 11  0,   8  0  0,
                8  6  0,   9  3  0,   9  7  0,   9  9  0,  10  0  0,  10  6  0,
               10  8  0,  10 10  0,  12  0  0,  13  1  0,  14  0  0,  16  0  0,
              -16  4  1, -13  5  1, -13  9  1, -10  8  1,  -9  9  1,  -8  6  1,
                   (  144 More Missing: see the .ckf listing file)
PLAT912_ALERT_4_G Missing # of FCF Reflections Above STh/L=  0.600       2391 Note
PLAT929_ALERT_5_G No Weight Pars,Obs and Calc R1,wR2,S not Checked          ! Info
PLAT961_ALERT_5_G Dataset Contains no Negative Intensities .......     Please Check
PLAT969_ALERT_5_G The 'Henn et al.' R-Factor-gap value ..........      0.806 Note
              Predicted wR2: Based on SigI**2  4.94 or SHELX Weight  4.94
PLAT978_ALERT_2_G Number C-C Bonds with Positive Residual Density.          1 Info
PLAT980_ALERT_1_G No Anomalous Scattering Factors Found in CIF ...     Please Check
```

---

  0 **ALERT level A** = Most likely a serious problem - resolve or explain
  0 **ALERT level B** = A potentially serious problem, consider carefully
559 **ALERT level C** = Check. Ensure it is not caused by an omission or oversight
 20 **ALERT level G** = General information/check it is not something unexpected

557 ALERT type 1 CIF construction/syntax error, inconsistent or missing data
  2 ALERT type 2 Indicator that the structure model may be wrong or deficient
  5 ALERT type 3 Indicator that the structure quality may be low
 10 ALERT type 4 Improvement, methodology, query or suggestion
  5 ALERT type 5 Informative message, check

It is advisable to attempt to resolve as many as possible of the alerts in all categories. Often the minor alerts point to easily fixed oversights, errors and omissions in your CIF or refinement strategy, so attention to these fine details can be worthwhile. In order to resolve some of the more serious problems it may be necessary to carry out additional measurements or structure refinements. However, the purpose of your study may justify the reported deviations and the more serious of these should normally be commented upon in the discussion or experimental section of a paper or in the "special_details" fields of the CIF. checkCIF was carefully designed to identify outliers and unusual parameters, but every test has its limitations and alerts that are not important in a particular case may appear. Conversely, the absence of alerts does not guarantee there are no aspects of the results needing attention. It is up to the individual to critically assess their own results and, if necessary, seek expert advice.

Publication of your CIF in IUCr journals

A basic structural check has been run on your CIF. These basic checks will be run on all CIFs submitted for publication in IUCr journals (*Acta Crystallographica*, *Journal of Applied Crystallography*, *Journal of Synchrotron Radiation*); however, if you intend to submit to *Acta Crystallographica Section C* or *E* or *IUCrData*, you should make sure that full publication checks are run on the final version of your CIF prior to submission.

Publication of your CIF in other journals

Please refer to the *Notes for Authors* of the relevant journal for any special instructions relating to CIF submission.

PLATON version of 04/06/2025; check.def file version of 30/05/2025

Datablock Bisacetonitrile_arachno-decaborane12_HAR - ellipsoid plot

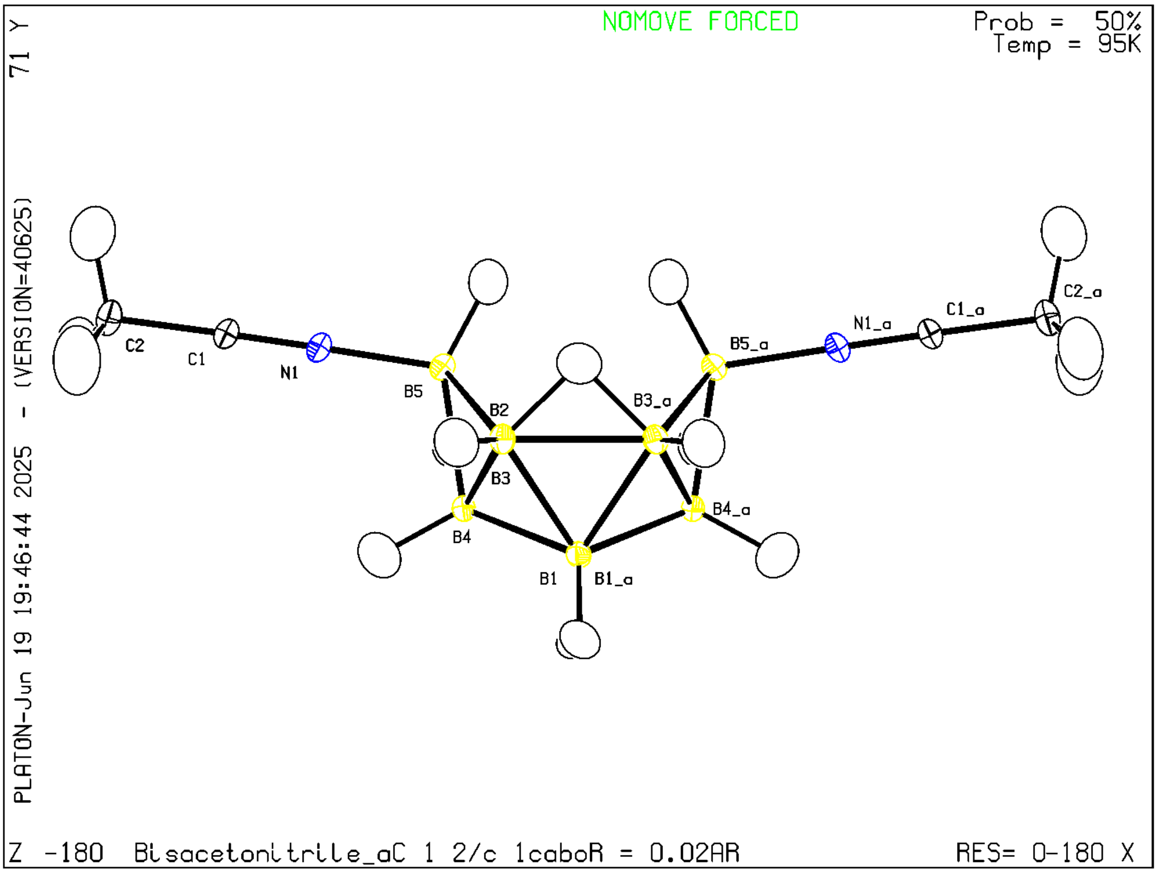

Supplement: Supplementary file 2 [file jz5c03918_si_002.zip › H_Bisacetonitrile arachno-decaborane(12)/Bis(acetonitrile)_arachno-decaborane(12)_HAR.pdf]
