# Supplementary material for: Periodic Hirshfeld Atom Refinement
Source: J Phys Chem Lett. 2026 Feb 27;17(11):3170–9. doi: 10.1021/acs.jpclett.5c03918 (PMC13007019; doi:10.1021/acs.jpclett.5c03918)

checkCIF/PLATON report

Structure factors have been supplied for datablock(s) NH3_6311

THIS REPORT IS FOR GUIDANCE ONLY. IF USED AS PART OF A REVIEW PROCEDURE FOR PUBLICATION, IT SHOULD NOT REPLACE THE EXPERTISE OF AN EXPERIENCED CRYSTALLOGRAPHIC REFEREE.

No syntax errors found. CIF dictionary Interpreting this report

Datablock: NH3_6311

Bond precision: = 0.0000 A Wavelength=0.71073

Cell:	a=5.1305(8)	b=5.1305(8)	c=5.1305(8)
	alpha=90	beta=90	gamma=90
Temperature:	160 K		

	Calculated	Reported
Volume	135.05(6)	135.05(4)
Space group	P 21 3	P 21 3
Hall group	P 2ac 2ab 3	P 2ac 2ab 3
Moiety formula	H3 N	H3 N
Sum formula	H3 N	H3 N
Mr	17.03	17.03
Dx,g cm-3	0.838	0.838
Z	4	4
Mu (mm-1)	0.064	0.064
F000	40.0	40.0
F000'	40.00	
h,k,lmax	7,7,7	6,6,7
Nref	134[85]	81
Tmin,Tmax		
Tmin'		

Correction method= Not given

Data completeness= 0.95/0.60 Theta(max)= 29.647

R(reflections)= 0.0090(81) wR2(reflections)= wR= 0.0088(81)

S = 0.961 Npar= 13

The following ALERTS were generated. Each ALERT has the format
test-name_ALERT_alert-type_alert-level.
Click on the hyperlinks for more details of the test.

Alert level B

```
PLAT049_ALERT_1_B Calculated Density Less Than 1.0 gcm-3 .........     0.8378 Check
              N
```

Author Response: This is a re-refinement of previously reported structure. ICSD code 84

Alert level C

```
REFLE01_ALERT_3_C  The _reflns_threshold_multiplier given is >= 4
            Premultiplier =    4.01
REFLE01_ALERT_3_C  The _reflns_threshold_multiplier given is >= 4
            Premultiplier =    4.01
PLAT089_ALERT_3_C Poor Data / Parameter Ratio (Zmax < 18) ........       6.15 Note
PLAT711_ALERT_1_C BOND    Unknown or Inconsistent Label .........          H2 Check
                    N           H2
PLAT711_ALERT_1_C BOND    Unknown or Inconsistent Label .........          H3 Check
                    N           H3
PLAT712_ALERT_1_C ANGLE   Unknown or Inconsistent Label .........          H2 Check
                    H1          N           H2
PLAT712_ALERT_1_C ANGLE   Unknown or Inconsistent Label .........          H3 Check
                    H1          N           H3
PLAT712_ALERT_1_C ANGLE   Unknown or Inconsistent Label .........          H2 Check
                    H2          N           H3
```

Alert level G

```
FORMU01_ALERT_2_G  There is a discrepancy between the atom counts in the
            _chemical_formula_sum and the formula from the _atom_site* data.
            Atom count from _chemical_formula_sum:H3 N1
            Atom count from the _atom_site data:  H9 N1
CELLZ01_ALERT_1_G Difference between formula and atom_site contents detected.
CELLZ01_ALERT_1_G ALERT: Large difference may be due to a
            symmetry error - see SYMMG tests
           From the CIF: _cell_formula_units_Z    4
           From the CIF: _chemical_formula_sum  H3 N
           TEST: Compare cell contents of formula and atom_site data

           atom    Z*formula  cif sites diff
           H         12.00     36.00  -24.00
           N          4.00      4.00    0.00
PLAT005_ALERT_5_G No Embedded Refinement Details Found  in the CIF     Please Do !
PLAT019_ALERT_1_G _diffrn_measured_fraction_theta_full/*_max < 1.0      0.884 Report
PLAT152_ALERT_1_G The Supplied and Calc. Volume s.u. Differ by ...          2 Units
PLAT802_ALERT_4_G CIF Input Record(s) with more than 80 Characters          1 Info
PLAT808_ALERT_5_G No Parseable SHELXL Style Weighting Scheme Found     Please Check
PLAT882_ALERT_1_G No Datum for _diffrn_reflns_av_unetI/netI ......     Please Do !
PLAT883_ALERT_1_G Absent Datum for _atom_sites_solution_primary ..     Please Do !
PLAT912_ALERT_4_G Missing # of FCF Reflections Above STh/L=  0.600          3 Note
```

```
PLAT929_ALERT_5_G No Weight Pars,Obs and Calc R1,wR2,S not Checked          ! Info
PLAT961_ALERT_5_G Dataset Contains no Negative Intensities .......     Please Check
PLAT969_ALERT_5_G The 'Henn et al.' R-Factor-gap value ..........      0.972 Note
              Predicted wR2: Based on SigI**2  1.85 or SHELX Weight  1.85
PLAT980_ALERT_1_G No Anomalous Scattering Factors Found in CIF ...     Please Check
```

```
   0 ALERT level A = Most likely a serious problem - resolve or explain
   1 ALERT level B = A potentially serious problem, consider carefully
   8 ALERT level C = Check. Ensure it is not caused by an omission or oversight
  15 ALERT level G = General information/check it is not something unexpected

  13 ALERT type 1 CIF construction/syntax error, inconsistent or missing data
   1 ALERT type 2 Indicator that the structure model may be wrong or deficient
   3 ALERT type 3 Indicator that the structure quality may be low
   2 ALERT type 4 Improvement, methodology, query or suggestion
   5 ALERT type 5 Informative message, check
```

It is advisable to attempt to resolve as many as possible of the alerts in all categories. Often the minor alerts point to easily fixed oversights, errors and omissions in your CIF or refinement strategy, so attention to these fine details can be worthwhile. In order to resolve some of the more serious problems it may be necessary to carry out additional measurements or structure refinements. However, the purpose of your study may justify the reported deviations and the more serious of these should normally be commented upon in the discussion or experimental section of a paper or in the "special_details" fields of the CIF. checkCIF was carefully designed to identify outliers and unusual parameters, but every test has its limitations and alerts that are not important in a particular case may appear. Conversely, the absence of alerts does not guarantee there are no aspects of the results needing attention. It is up to the individual to critically assess their own results and, if necessary, seek expert advice.

Publication of your CIF in IUCr journals

A basic structural check has been run on your CIF. These basic checks will be run on all CIFs submitted for publication in IUCr journals (*Acta Crystallographica*, *Journal of Applied Crystallography*, *Journal of Synchrotron Radiation*); however, if you intend to submit to *Acta Crystallographica Section C* or *E* or *IUCrData*, you should make sure that full publication checks are run on the final version of your CIF prior to submission.

Publication of your CIF in other journals

Please refer to the *Notes for Authors* of the relevant journal for any special instructions relating to CIF submission.

PLATON version of 04/06/2025; check.def file version of 30/05/2025

Datablock NH3_6311 - ellipsoid plot

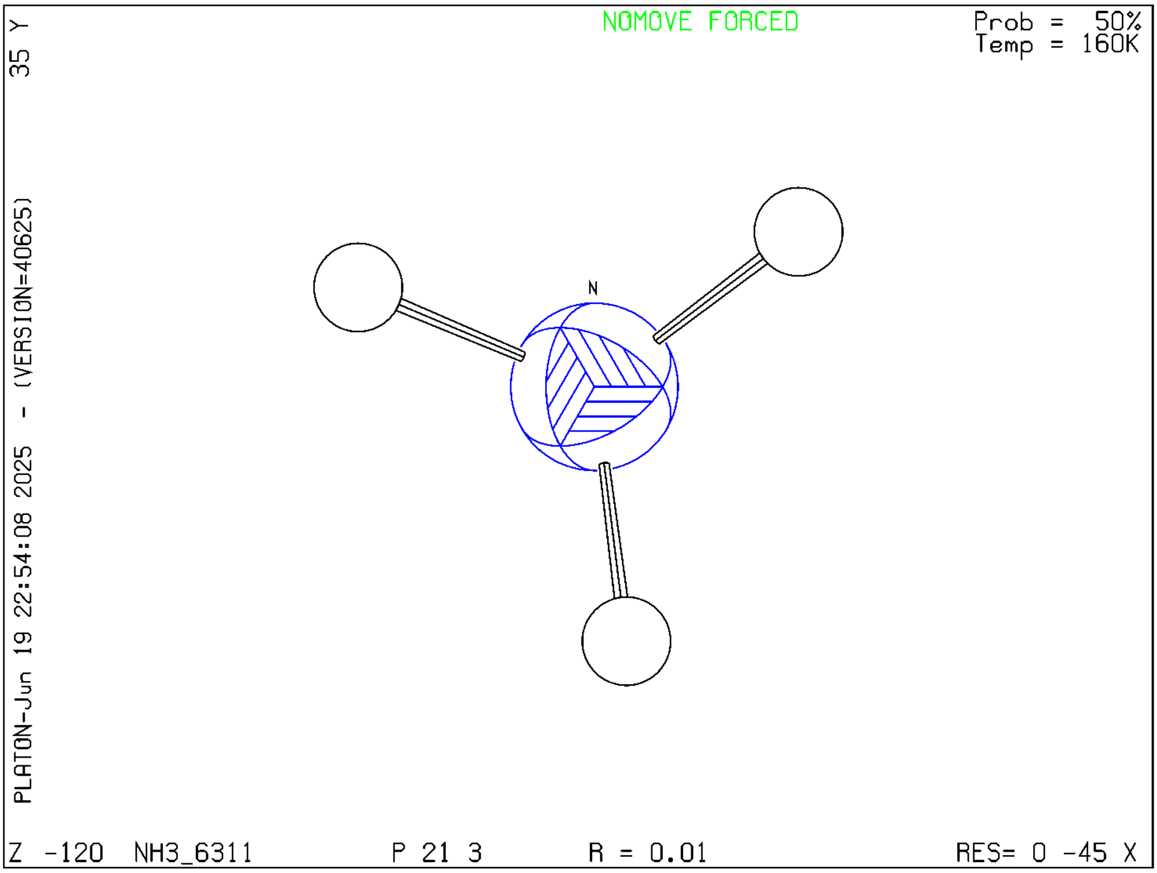

Supplement: Supplementary file 2 [file NH3_6311.pdf › A_ammonia/basis_dependancy/NH3_6311.pdf]
