# Supplementary material for: Periodic Hirshfeld Atom Refinement
Source: J Phys Chem Lett. 2026 Feb 27;17(11):3170–9. doi: 10.1021/acs.jpclett.5c03918 (PMC13007019; doi:10.1021/acs.jpclett.5c03918)

checkCIF/PLATON report

Structure factors have been supplied for datablock(s) Diborane_HAR

THIS REPORT IS FOR GUIDANCE ONLY. IF USED AS PART OF A REVIEW PROCEDURE FOR PUBLICATION, IT SHOULD NOT REPLACE THE EXPERTISE OF AN EXPERIENCED CRYSTALLOGRAPHIC REFEREE.

No syntax errors found. CIF dictionary Interpreting this report

Datablock: Diborane_HAR

Bond precision: B- B = 0.0030 A Wavelength=0.71073

Cell:	a=4.400(7)	b=6.487(7)	c=5.734(7)
	alpha=90	beta=105.35(5)	gamma=90
Temperature:	94 K		

	Calculated	Reported
Volume	157.8(4)	157.8(4)
Space group	P 21/n	P 1 21/n 1
Hall group	-P 2yn	-P 2yn
Moiety formula	B2 H6	B2 H6
Sum formula	B2 H6	B2 H6
Mr	27.67	27.67
Dx,g cm-3	0.582	0.582
Z	2	2
Mu (mm-1)	0.022	0.021
F000	32.0	32.0
F000'	32.00	
h,k,lmax	9,14,12	9,13,12
Nref	1831	889
Tmin,Tmax		
Tmin'		

Correction method= Not given

Data completeness= 0.486 Theta(max)= 52.382

R(reflections)= 0.0318(889) wR2(reflections)= wR= 0.0328(889)

S = 1.212 Npar= 37

The following ALERTS were generated. Each ALERT has the format
test-name_ALERT_alert-type_alert-level.
Click on the hyperlinks for more details of the test.

Alert level B

PLAT049_ALERT_1_B Calculated Density Less Than 1.0 gcm-3 .... 0.5823 Check
B1

Author Response: This structure consists of light atoms. This is a re-refinement of previously reported structure. ICSD code 1789586 (doi: 10.1002/zaac.200400104)

PLAT920_ALERT_1_B Theta(Max) in CIF and FCF Differ by .... 0.68 Degree

Author Response: Only the reflections satisfying _reflns_threshold_expression are shown in the structure factors data. None of the reflections in the range of 51.7 < theta < 52.382 degrees satisfy the criteria: F>0 and F/u(F)>4.0

Alert level C

ABSMU01_ALERT_1_C The ratio of given/expected absorption coefficient lies
outside the range 0.99 <> 1.01
Calculated value of mu = 0.022
Value of mu given = 0.021
REFLE01_ALERT_3_C The _reflns_threshold_multiplier given is >= 4
Premultiplier = 4.01
REFLE01_ALERT_3_C The _reflns_threshold_multiplier given is >= 4
Premultiplier = 4.01
PLAT148_ALERT_3_C s.u. on the a - Axis is (Too) Large .... 0.007 Ang.
PLAT148_ALERT_3_C s.u. on the b - Axis is (Too) Large .... 0.0070 Ang.
PLAT148_ALERT_3_C s.u. on the c - Axis is (Too) Large .... 0.007 Ang.
PLAT303_ALERT_2_C Full Occupancy Atom H1 with # Connections 2.00 Check
PLAT711_ALERT_1_C BOND Unknown or Inconsistent Label .... B2 Check
B1 B2
PLAT711_ALERT_1_C BOND Unknown or Inconsistent Label .... H3 Check
B1 H3
PLAT711_ALERT_1_C BOND Unknown or Inconsistent Label .... B2 Check
B2 H1
PLAT711_ALERT_1_C BOND Unknown or Inconsistent Label .... B2 Check
B2 H3
PLAT711_ALERT_1_C BOND Unknown or Inconsistent Label .... B2 Check
B2 H5
PLAT711_ALERT_1_C BOND Unknown or Inconsistent Label .... B2 Check
B2 H6
PLAT712_ALERT_1_C ANGLE Unknown or Inconsistent Label .... B2 Check
B1 B2 H1
PLAT712_ALERT_1_C ANGLE Unknown or Inconsistent Label .... B2 Check
B1 B2 H3
PLAT712_ALERT_1_C ANGLE Unknown or Inconsistent Label .... B2 Check
B1 B2 H5
PLAT712_ALERT_1_C ANGLE Unknown or Inconsistent Label .... B2 Check

```
                    B1          B2          H6
PLAT712_ALERT_1_C ANGLE   Unknown or Inconsistent Label .........         B2 Check
                    B2          B1          H1
PLAT712_ALERT_1_C ANGLE   Unknown or Inconsistent Label .........         B2 Check
                    B2          B1          H2
PLAT712_ALERT_1_C ANGLE   Unknown or Inconsistent Label .........         B2 Check
                    B2          B1          H3
PLAT712_ALERT_1_C ANGLE   Unknown or Inconsistent Label .........         B2 Check
                    B2          B1          H4
PLAT712_ALERT_1_C ANGLE   Unknown or Inconsistent Label .........         B2 Check
                    B1          H1          B2
PLAT712_ALERT_1_C ANGLE   Unknown or Inconsistent Label .........         H3 Check
                    B1          H3          B2
PLAT712_ALERT_1_C ANGLE   Unknown or Inconsistent Label .........         H3 Check
                    H1          B1          H3
PLAT712_ALERT_1_C ANGLE   Unknown or Inconsistent Label .........         B2 Check
                    H1          B2          H3
PLAT712_ALERT_1_C ANGLE   Unknown or Inconsistent Label .........         B2 Check
                    H1          B2          H5
PLAT712_ALERT_1_C ANGLE   Unknown or Inconsistent Label .........         B2 Check
                    H1          B2          H6
PLAT712_ALERT_1_C ANGLE   Unknown or Inconsistent Label .........         H3 Check
                    H2          B1          H3
PLAT712_ALERT_1_C ANGLE   Unknown or Inconsistent Label .........         H3 Check
                    H3          B1          H4
PLAT712_ALERT_1_C ANGLE   Unknown or Inconsistent Label .........         H3 Check
                    H3          B2          H5
PLAT712_ALERT_1_C ANGLE   Unknown or Inconsistent Label .........         H3 Check
                    H3          B2          H6
PLAT712_ALERT_1_C ANGLE   Unknown or Inconsistent Label .........         H5 Check
                    H5          B2          H6
PLAT713_ALERT_1_C TORSION Unknown or Inconsistent Label .........         B2 Check
                    B1          H1          B2          H3
PLAT713_ALERT_1_C TORSION Unknown or Inconsistent Label .........         B2 Check
                    B1          H1          B2          H5
PLAT713_ALERT_1_C TORSION Unknown or Inconsistent Label .........         B2 Check
                    B1          H1          B2          H6
PLAT713_ALERT_1_C TORSION Unknown or Inconsistent Label .........         H3 Check
                    B1          H3          B2          H1
PLAT713_ALERT_1_C TORSION Unknown or Inconsistent Label .........         H3 Check
                    B1          H3          B2          H5
PLAT713_ALERT_1_C TORSION Unknown or Inconsistent Label .........         H3 Check
                    B1          H3          B2          H6
PLAT713_ALERT_1_C TORSION Unknown or Inconsistent Label .........         B2 Check
                    B2          H1          B1          H3
PLAT713_ALERT_1_C TORSION Unknown or Inconsistent Label .........         B2 Check
                    B2          H1          B1          H4
PLAT713_ALERT_1_C TORSION Unknown or Inconsistent Label .........         B2 Check
                    B2          H3          B1          H4
PLAT713_ALERT_1_C TORSION Unknown or Inconsistent Label .........         B2 Check
                    H1          B1          B2          H3
PLAT713_ALERT_1_C TORSION Unknown or Inconsistent Label .........         B2 Check
                    H1          B1          B2          H5
PLAT713_ALERT_1_C TORSION Unknown or Inconsistent Label .........         B2 Check
                    H1          B1          B2          H6
PLAT713_ALERT_1_C TORSION Unknown or Inconsistent Label .........         B2 Check
                    H1          B2          B1          H2
```

```
PLAT713_ALERT_1_C TORSION Unknown or Inconsistent Label .........         B2 Check
                    H1          B2          B1          H3
PLAT713_ALERT_1_C TORSION Unknown or Inconsistent Label .........         B2 Check
                    H1          B2          B1          H4
PLAT713_ALERT_1_C TORSION Unknown or Inconsistent Label .........         B2 Check
                    H2          B1          B2          H3
PLAT713_ALERT_1_C TORSION Unknown or Inconsistent Label .........         B2 Check
                    H2          B1          B2          H5
PLAT713_ALERT_1_C TORSION Unknown or Inconsistent Label .........         B2 Check
                    H2          B1          B2          H6
PLAT713_ALERT_1_C TORSION Unknown or Inconsistent Label .........         H3 Check
                    H3          B1          B2          H5
PLAT713_ALERT_1_C TORSION Unknown or Inconsistent Label .........         H3 Check
                    H3          B1          B2          H6
PLAT713_ALERT_1_C TORSION Unknown or Inconsistent Label .........         H3 Check
                    H3          B2          B1          H4
PLAT713_ALERT_1_C TORSION Unknown or Inconsistent Label .........         B2 Check
                    H4          B1          B2          H5
PLAT713_ALERT_1_C TORSION Unknown or Inconsistent Label .........         B2 Check
                    H4          B1          B2          H6
PLAT713_ALERT_1_C TORSION Unknown or Inconsistent Label .........         H3 Check
                    H1          B1          H3          B2
PLAT713_ALERT_1_C TORSION Unknown or Inconsistent Label .........         B2 Check
                    H2          B1          H1          B2
PLAT713_ALERT_1_C TORSION Unknown or Inconsistent Label .........         H3 Check
                    H2          B1          H3          B2
PLAT992_ALERT_5_C Repd & Actual _reflns_number_gt Values Differ by         19 Check
```

Alert level G

```
FORMU01_ALERT_2_G  There is a discrepancy between the atom counts in the
            _chemical_formula_sum and the formula from the _atom_site* data.
            Atom count from _chemical_formula_sum:H6 B2
            Atom count from the _atom_site data:  H12 B4
CELLZ01_ALERT_1_G Difference between formula and atom_site contents detected.
CELLZ01_ALERT_1_G ALERT: Large difference may be due to a
            symmetry error - see SYMMG tests
           From the CIF: _cell_formula_units_Z    2
           From the CIF: _chemical_formula_sum  B2 H6
           TEST: Compare cell contents of formula and atom_site data

           atom    Z*formula  cif sites diff
           B          4.00      8.00   -4.00
           H         12.00     24.00  -12.00
PLAT005_ALERT_5_G No Embedded Refinement Details Found  in the CIF     Please Do !
PLAT410_ALERT_2_G Short Intra H...H Contact  H1       ..H2       .       2.01 Ang.
                                              -x,1-y,1-z  =      2_566 Check
PLAT410_ALERT_2_G Short Intra H...H Contact  H1       ..H4       .       1.99 Ang.
                                              -x,1-y,1-z  =      2_566 Check
PLAT808_ALERT_5_G No Parseable SHELXL Style Weighting Scheme Found     Please Check
PLAT881_ALERT_1_G No Datum for _diffrn_reflns_av_R_equivalents ...     Please Do !
PLAT883_ALERT_1_G Absent Datum for _atom_sites_solution_primary ..     Please Do !
PLAT911_ALERT_3_G Missing FCF Refl Between Thmin & STh/L=    0.600         33 Report
                 2  1  0,   4  1  0,   4  3  0,   3  4  0,   2  5  0,   0  6  0,
                 1  6  0,   2  6  0,   3  6  0,   2  7  0,   1  4  1,   0  5  1,
                 2  5  1,  -3  6  1,  -1  6  1,  -2  1  2,   1  1  2,  -4  4  2,
                -3  5  2,  -2  6  2,   2  6  2,  -1  7  2,   1  7  2,  -2  2  3,
```

```
              -1  6  3,   1  6  3,   2  1  4,  -2  6  4,   0  6  4,  -3  4  5,
                  (    3 More Missing: see the .ckf listing file)
PLAT912_ALERT_4_G Missing # of FCF Reflections Above STh/L=  0.600        850 Note
PLAT929_ALERT_5_G No Weight Pars,Obs and Calc R1,wR2,S not Checked          ! Info
PLAT961_ALERT_5_G Dataset Contains no Negative Intensities .......     Please Check
PLAT969_ALERT_5_G The 'Henn et al.' R-Factor-gap value ..........      1.263 Note
             Predicted wR2: Based on SigI**2  5.26 or SHELX Weight  5.26
PLAT980_ALERT_1_G No Anomalous Scattering Factors Found in CIF ...     Please Check
```

```
   0 ALERT level A = Most likely a serious problem - resolve or explain
   2 ALERT level B = A potentially serious problem, consider carefully
  59 ALERT level C = Check. Ensure it is not caused by an omission or oversight
  15 ALERT level G = General information/check it is not something unexpected

  59 ALERT type 1 CIF construction/syntax error, inconsistent or missing data
   4 ALERT type 2 Indicator that the structure model may be wrong or deficient
   6 ALERT type 3 Indicator that the structure quality may be low
   1 ALERT type 4 Improvement, methodology, query or suggestion
   6 ALERT type 5 Informative message, check
```

It is advisable to attempt to resolve as many as possible of the alerts in all categories. Often the minor alerts point to easily fixed oversights, errors and omissions in your CIF or refinement strategy, so attention to these fine details can be worthwhile. In order to resolve some of the more serious problems it may be necessary to carry out additional measurements or structure refinements. However, the purpose of your study may justify the reported deviations and the more serious of these should normally be commented upon in the discussion or experimental section of a paper or in the "special_details" fields of the CIF. checkCIF was carefully designed to identify outliers and unusual parameters, but every test has its limitations and alerts that are not important in a particular case may appear. Conversely, the absence of alerts does not guarantee there are no aspects of the results needing attention. It is up to the individual to critically assess their own results and, if necessary, seek expert advice.

Publication of your CIF in IUCr journals

A basic structural check has been run on your CIF. These basic checks will be run on all CIFs submitted for publication in IUCr journals (*Acta Crystallographica*, *Journal of Applied Crystallography*, *Journal of Synchrotron Radiation*); however, if you intend to submit to *Acta Crystallographica Section C* or *E* or *IUCrData*, you should make sure that full publication checks are run on the final version of your CIF prior to submission.

Publication of your CIF in other journals

Please refer to the *Notes for Authors* of the relevant journal for any special instructions relating to CIF submission.

PLATON version of 04/06/2025; check.def file version of 30/05/2025

Datablock Diborane_HAR - ellipsoid plot

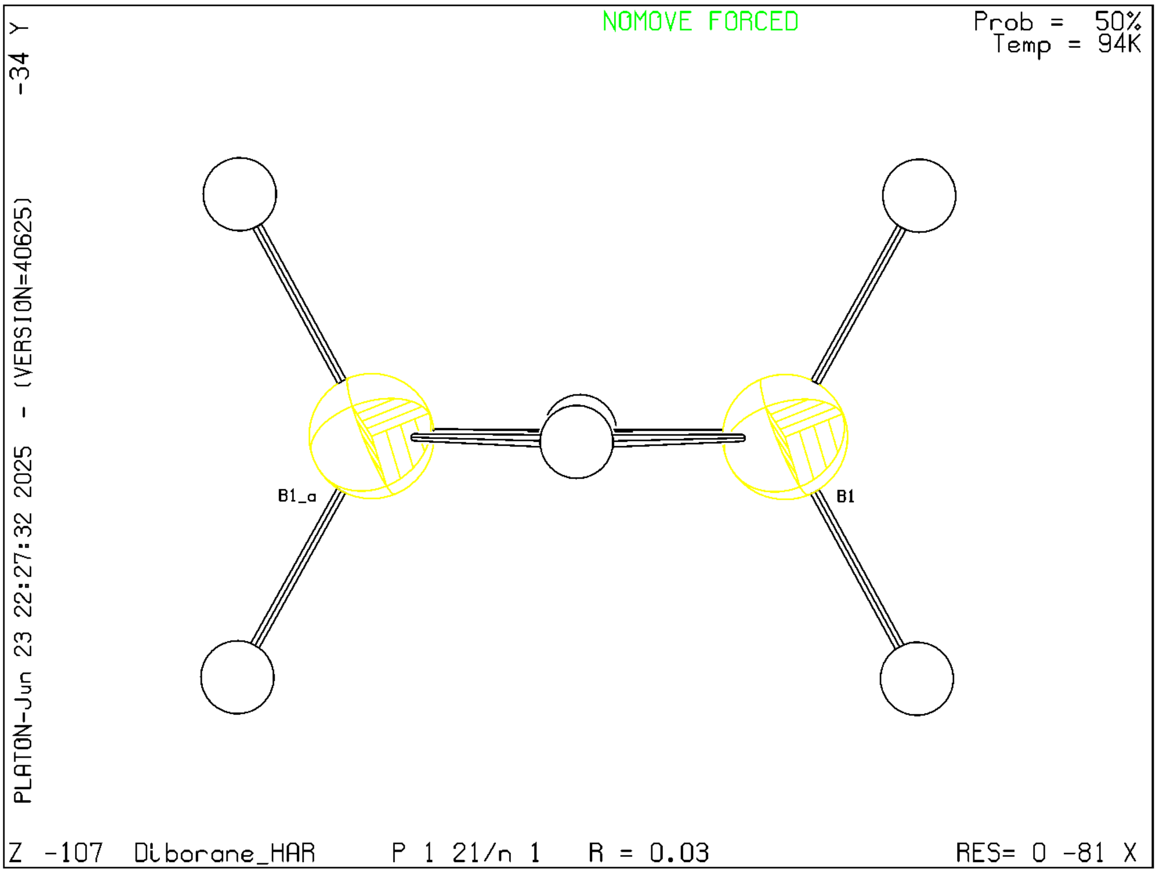

Supplement: Supplementary file 2 [file Diborane(6)_HAR.pdf › B_Diborane(6)/Diborane(6)_HAR.pdf]
