# Supplementary material for: Periodic Hirshfeld Atom Refinement
Source: J Phys Chem Lett. 2026 Feb 27;17(11):3170–9. doi: 10.1021/acs.jpclett.5c03918 (PMC13007019; doi:10.1021/acs.jpclett.5c03918)

checkCIF/PLATON report

Structure factors have been supplied for datablock(s) Hydrazine_borane_HAR

THIS REPORT IS FOR GUIDANCE ONLY. IF USED AS PART OF A REVIEW PROCEDURE FOR PUBLICATION, IT SHOULD NOT REPLACE THE EXPERTISE OF AN EXPERIENCED CRYSTALLOGRAPHIC REFEREE.

No syntax errors found. CIF dictionary Interpreting this report

Datablock: Hydrazine_borane_HAR

Bond precision: N- N = 0.0003 A Wavelength=0.71073

Cell:	a=12.974(2)	b=5.0702(10)	c=9.5069(10)
	alpha=90	beta=90	gamma=90
Temperature:	95 K		

	Calculated	Reported
Volume	625.37(17)	625.37(17)
Space group	P b c n	P b c n
Hall group	-P 2n 2ab	-P 2n 2ab
Moiety formula	B H7 N2	B H7 N2
Sum formula	B H7 N2	B H7 N2
Mr	45.89	45.88
Dx,g cm-3	0.975	0.975
Z	8	8
Mu (mm-1)	0.064	0.064
F000	208.0	208.0
F000’	208.01	
h,k,lmax	33,13,24	33,13,24
Nref	5827	4559
Tmin,Tmax	0.981,0.987	
Tmin’	0.981	

Correction method= Not given

Data completeness= 0.782 Theta(max)= 68.104

R(reflections)= 0.0220(4559) wR2(reflections)= wR= 0.0245(4559)

S = 1.405 Npar= 91

The following ALERTS were generated. Each ALERT has the format
test-name_ALERT_alert-type_alert-level.
Click on the hyperlinks for more details of the test.

Alert level B

```
PLAT049_ALERT_1_B Calculated Density Less Than 1.0 gcm-3 .........     0.9747 Check
```

Author Response: This structure consists of many light atoms

```
PLAT094_ALERT_2_B Ratio of Maximum / Minimum Residual Density ....       5.39 Report
```

Author Response: The N-N bond is poorly modeled.

Alert level C

```
DIFMX02_ALERT_1_C  The maximum difference density is > 0.1*ZMAX*0.75
            The relevant atom site should be identified.
REFLE01_ALERT_3_C  The _reflns_threshold_multiplier given is >= 4
            Premultiplier =    4.01
REFLE01_ALERT_3_C  The _reflns_threshold_multiplier given is >= 4
            Premultiplier =    4.01
PLAT097_ALERT_2_C Large Reported Max.  (Positive) Residual Density       0.55 eA-3
PLAT992_ALERT_5_C Repd & Actual _reflns_number_gt Values Differ by         19 Check
```

Alert level G

```
PLAT005_ALERT_5_G No Embedded Refinement Details Found  in the CIF     Please Do !
PLAT415_ALERT_2_G Short Inter D-H..H-X       H4       ..H5       .       2.05 Ang.
                                              1-x,1-y,1-z  =      2_666 Check
PLAT808_ALERT_5_G No Parseable SHELXL Style Weighting Scheme Found     Please Check
PLAT883_ALERT_1_G Absent Datum for _atom_sites_solution_primary ..     Please Do !
PLAT911_ALERT_3_G Missing FCF Refl Between Thmin & STh/L=    0.600          39 Report
                1  5  1,    9  4  1,   10  2  1,   11  4  1,    6  5  2,   10  2  2,
               12  2  2,   13  1  2,   13  3  2,   14  2  2,    1  5  3,    7  4  3,
                9  4  3,    5  2  4,   10  2  4,   13  2  4,    1  2  5,    9  4  5,
               13  2  5,    7  2  6,    9  2  6,   10  2  6,   11  2  6,    0  4  7,
                3  4  7,   11  1  7,    0  2  8,    2  0  8,    2  2  8,    4  2  8,
                   (    9 More Missing: see the .ckf listing file)
PLAT912_ALERT_4_G Missing # of FCF Reflections Above STh/L=  0.600       1229 Note
PLAT929_ALERT_5_G No Weight Pars,Obs and Calc R1,wR2,S not Checked          ! Info
PLAT961_ALERT_5_G Dataset Contains no Negative Intensities .......     Please Check
PLAT969_ALERT_5_G The 'Henn et al.' R-Factor-gap value ..........      1.400 Note
              Predicted wR2: Based on SigI**2  3.48 or SHELX Weight  3.48
PLAT980_ALERT_1_G No Anomalous Scattering Factors Found in CIF ...     Please Check
```

0 **ALERT level A** = Most likely a serious problem - resolve or explain
2 **ALERT level B** = A potentially serious problem, consider carefully
5 **ALERT level C** = Check. Ensure it is not caused by an omission or oversight
10 **ALERT level G** = General information/check it is not something unexpected

```
4 ALERT type 1 CIF construction/syntax error, inconsistent or missing data
3 ALERT type 2 Indicator that the structure model may be wrong or deficient
3 ALERT type 3 Indicator that the structure quality may be low
1 ALERT type 4 Improvement, methodology, query or suggestion
6 ALERT type 5 Informative message, check
```

It is advisable to attempt to resolve as many as possible of the alerts in all categories. Often the minor alerts point to easily fixed oversights, errors and omissions in your CIF or refinement strategy, so attention to these fine details can be worthwhile. In order to resolve some of the more serious problems it may be necessary to carry out additional measurements or structure refinements. However, the purpose of your study may justify the reported deviations and the more serious of these should normally be commented upon in the discussion or experimental section of a paper or in the "special_details" fields of the CIF. checkCIF was carefully designed to identify outliers and unusual parameters, but every test has its limitations and alerts that are not important in a particular case may appear. Conversely, the absence of alerts does not guarantee there are no aspects of the results needing attention. It is up to the individual to critically assess their own results and, if necessary, seek expert advice.

Publication of your CIF in IUCr journals

A basic structural check has been run on your CIF. These basic checks will be run on all CIFs submitted for publication in IUCr journals (*Acta Crystallographica*, *Journal of Applied Crystallography*, *Journal of Synchrotron Radiation*); however, if you intend to submit to *Acta Crystallographica Section C* or *E* or *IUCrData*, you should make sure that full publication checks are run on the final version of your CIF prior to submission.

Publication of your CIF in other journals

Please refer to the *Notes for Authors* of the relevant journal for any special instructions relating to CIF submission.

PLATON version of 04/06/2025; check.def file version of 30/05/2025

Datablock Hydrazine_borane_HAR - ellipsoid plot

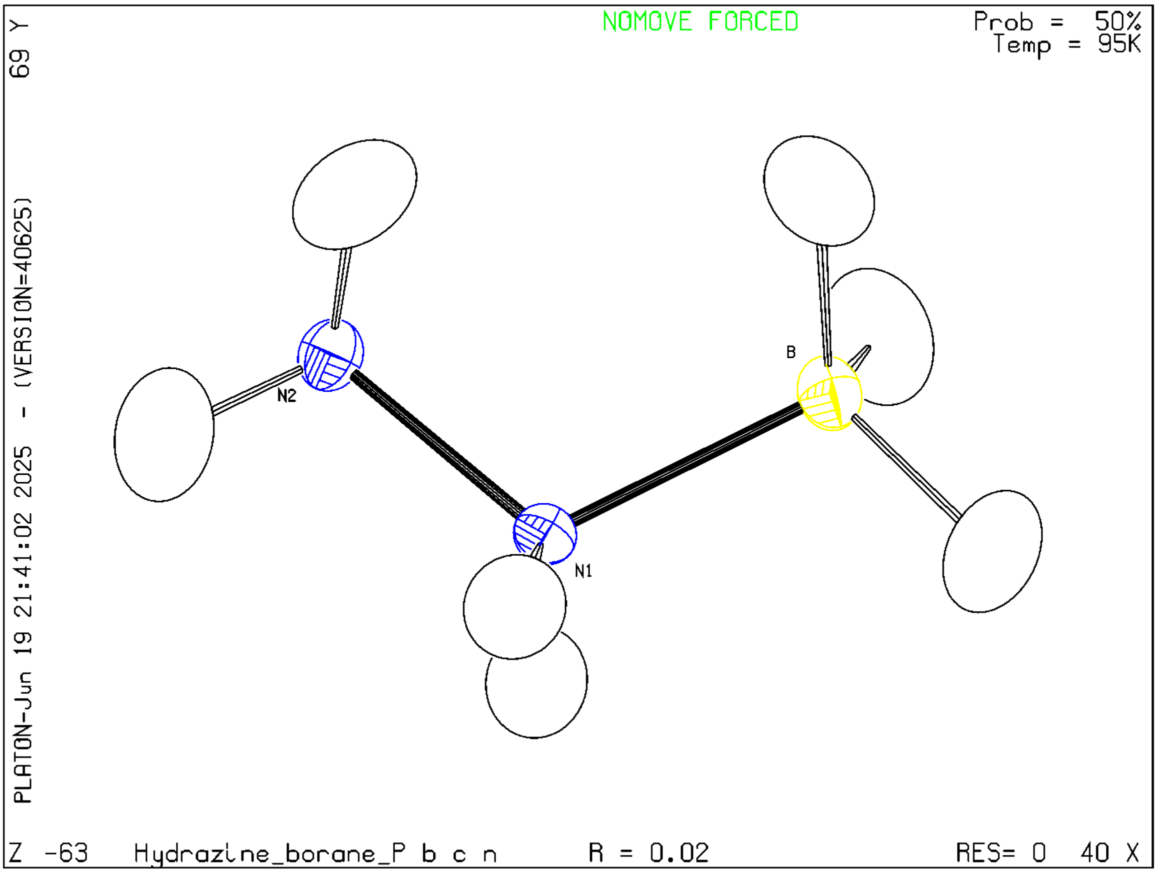

Supplement: Supplementary file 2 [file Hydrazine_borane_HAR.pdf › D_Hydrazine_borane/Hydrazine_borane_HAR.pdf]
