# Supplementary material for: Periodic Hirshfeld Atom Refinement
Source: J Phys Chem Lett. 2026 Feb 27;17(11):3170–9. doi: 10.1021/acs.jpclett.5c03918 (PMC13007019; doi:10.1021/acs.jpclett.5c03918)

checkCIF/PLATON report

Structure factors have been supplied for datablock(s) Bisammonium_closo-hexaborate6_HAR

THIS REPORT IS FOR GUIDANCE ONLY. IF USED AS PART OF A REVIEW PROCEDURE FOR PUBLICATION, IT SHOULD NOT REPLACE THE EXPERTISE OF AN EXPERIENCED CRYSTALLOGRAPHIC REFEREE.

No syntax errors found.   CIF dictionary   Interpreting this report

Datablock: Bisammonium_closo-hexaborate6_HAR

```
Bond precision:   B- B = 0.0002 A          Wavelength=0.71073

Cell:                a=9.0998(4)      b=9.0998(4)      c=9.0998(4)
                     alpha=90         beta=90          gamma=90
Temperature:         100 K
```

	Calculated	Reported
Volume	753.52(10)	753.52(6)
Space group	F m -3 m	F m -3 m
Hall group	-F 4 2 3	-F 4 2 3
Moiety formula	B12 H12, H16 N4	2(N H4), B6 H6
Sum formula	B12 H28 N4	B6 H14 N2
Mr	213.98	106.99
Dx,g cm-3	0.943	0.943
Z	2	4
Mu (mm-1)	0.046	0.046
F000	232.0	232.0
F000’	232.00	
h,k,lmax	24,24,24	13,17,24
Nref	450	403
Tmin,Tmax		0.710,1.000
Tmin’		

```
Correction method= # Reported T Limits: Tmin=0.710 Tmax=1.000
AbsCorr = MULTI-SCAN

Data completeness= 0.896           Theta(max)= 73.530

                                                      wR2(reflections)=
R(reflections)= 0.0180( 403)                          wR= 0.0239( 403)

S = 1.469                  Npar= 11
```

The following ALERTS were generated. Each ALERT has the format
test-name_ALERT_alert-type_alert-level.
Click on the hyperlinks for more details of the test.

Alert level B

```
PLAT049_ALERT_1_B Calculated Density Less Than 1.0 gcm-3 .........     0.9431 Check
              B1
```

Author Response: This structure consists of many light atoms

Alert level C

```
ABSMU01_ALERT_1_C  The ratio of given/expected absorption coefficient lies
               outside the range 0.99 <> 1.01
            Calculated value of mu =     0.046
            Value of mu given      =     0.046
REFLE01_ALERT_3_C  The _reflns_threshold_multiplier given is >= 4
            Premultiplier =    4.01
REFLE01_ALERT_3_C  The _reflns_threshold_multiplier given is >= 4
            Premultiplier =    4.01
PLAT041_ALERT_1_C Calc. and Reported SumFormula    Strings  Differ     Please Check
              Calc: B12 H28 N4
              Rep.: B6 H14 N2
PLAT042_ALERT_1_C Calc. and Reported MoietyFormula Strings  Differ     Please Check
              Calc: B12 H12, H16 N4
              Rep.: 2(N H4), B6 H6
PLAT053_ALERT_1_C Minimum Crystal Dimension Missing (or Error) ...     Please Check
PLAT054_ALERT_1_C Medium  Crystal Dimension Missing (or Error) ...     Please Check
PLAT055_ALERT_1_C Maximum Crystal Dimension Missing (or Error) ...     Please Check
PLAT202_ALERT_3_C Isotropic non-H Atoms in Anion/Solvent .........          1 Check
              N
PLAT420_ALERT_2_C D-H Bond Without Acceptor  N        --H1A      .     Please Check
PLAT711_ALERT_1_C BOND    Unknown or Inconsistent Label ..........        H2A Check
                    N           H2A
PLAT711_ALERT_1_C BOND    Unknown or Inconsistent Label ..........        H3A Check
                    N           H3A
PLAT711_ALERT_1_C BOND    Unknown or Inconsistent Label ..........        H4A Check
                    N           H4A
PLAT711_ALERT_1_C BOND    Unknown or Inconsistent Label ..........         B2 Check
                    B1          B2
PLAT711_ALERT_1_C BOND    Unknown or Inconsistent Label ..........         B3 Check
                    B1          B3
PLAT711_ALERT_1_C BOND    Unknown or Inconsistent Label ..........         B5 Check
                    B1          B5
PLAT711_ALERT_1_C BOND    Unknown or Inconsistent Label ..........         B6 Check
                    B1          B6
PLAT711_ALERT_1_C BOND    Unknown or Inconsistent Label ..........         B2 Check
                    B2          B3
PLAT711_ALERT_1_C BOND    Unknown or Inconsistent Label ..........         B2 Check
                    B2          B4
PLAT711_ALERT_1_C BOND    Unknown or Inconsistent Label ..........         B2 Check
                    B2          B5
```

```
PLAT711_ALERT_1_C BOND    Unknown or Inconsistent Label .........          B3 Check
                    B3          B4
PLAT711_ALERT_1_C BOND    Unknown or Inconsistent Label .........          B3 Check
                    B3          B6
PLAT711_ALERT_1_C BOND    Unknown or Inconsistent Label .........          B4 Check
                    B4          B5
PLAT711_ALERT_1_C BOND    Unknown or Inconsistent Label .........          B4 Check
                    B4          B6
PLAT711_ALERT_1_C BOND    Unknown or Inconsistent Label .........          B5 Check
                    B5          B6
PLAT711_ALERT_1_C BOND    Unknown or Inconsistent Label .........          B2 Check
                    B2          H2
PLAT711_ALERT_1_C BOND    Unknown or Inconsistent Label .........          B3 Check
                    B3          H3
PLAT711_ALERT_1_C BOND    Unknown or Inconsistent Label .........          B4 Check
                    B4          H4
PLAT711_ALERT_1_C BOND    Unknown or Inconsistent Label .........          B5 Check
                    B5          H5
PLAT711_ALERT_1_C BOND    Unknown or Inconsistent Label .........          B6 Check
                    B6          H6
PLAT712_ALERT_1_C ANGLE   Unknown or Inconsistent Label .........          B2 Check
                    B1          B2          B3
PLAT712_ALERT_1_C ANGLE   Unknown or Inconsistent Label .........          B2 Check
                    B1          B2          B4
PLAT712_ALERT_1_C ANGLE   Unknown or Inconsistent Label .........          B2 Check
                    B1          B2          B5
PLAT712_ALERT_1_C ANGLE   Unknown or Inconsistent Label .........          B3 Check
                    B1          B3          B2
PLAT712_ALERT_1_C ANGLE   Unknown or Inconsistent Label .........          B3 Check
                    B1          B3          B4
PLAT712_ALERT_1_C ANGLE   Unknown or Inconsistent Label .........          B3 Check
                    B1          B3          B6
PLAT712_ALERT_1_C ANGLE   Unknown or Inconsistent Label .........          B5 Check
                    B1          B5          B2
PLAT712_ALERT_1_C ANGLE   Unknown or Inconsistent Label .........          B5 Check
                    B1          B5          B4
PLAT712_ALERT_1_C ANGLE   Unknown or Inconsistent Label .........          B5 Check
                    B1          B5          B6
PLAT712_ALERT_1_C ANGLE   Unknown or Inconsistent Label .........          B6 Check
                    B1          B6          B3
PLAT712_ALERT_1_C ANGLE   Unknown or Inconsistent Label .........          B6 Check
                    B1          B6          B4
PLAT712_ALERT_1_C ANGLE   Unknown or Inconsistent Label .........          B6 Check
                    B1          B6          B5
PLAT712_ALERT_1_C ANGLE   Unknown or Inconsistent Label .........          B2 Check
                    B2          B1          B3
PLAT712_ALERT_1_C ANGLE   Unknown or Inconsistent Label .........          B2 Check
                    B2          B1          B5
PLAT712_ALERT_1_C ANGLE   Unknown or Inconsistent Label .........          B2 Check
                    B2          B1          B6
PLAT712_ALERT_1_C ANGLE   Unknown or Inconsistent Label .........          B2 Check
                    B2          B3          B4
PLAT712_ALERT_1_C ANGLE   Unknown or Inconsistent Label .........          B2 Check
                    B2          B3          B6
PLAT712_ALERT_1_C ANGLE   Unknown or Inconsistent Label .........          B2 Check
                    B2          B4          B3
PLAT712_ALERT_1_C ANGLE   Unknown or Inconsistent Label .........          B2 Check
```

```
                    B2          B4          B5
PLAT712_ALERT_1_C ANGLE   Unknown or Inconsistent Label .........         B2 Check
                    B2          B4          B6
PLAT712_ALERT_1_C ANGLE   Unknown or Inconsistent Label .........         B2 Check
                    B2          B5          B4
PLAT712_ALERT_1_C ANGLE   Unknown or Inconsistent Label .........         B2 Check
                    B2          B5          B6
PLAT712_ALERT_1_C ANGLE   Unknown or Inconsistent Label .........         B3 Check
                    B3          B1          B5
PLAT712_ALERT_1_C ANGLE   Unknown or Inconsistent Label .........         B3 Check
                    B3          B1          B6
PLAT712_ALERT_1_C ANGLE   Unknown or Inconsistent Label .........         B3 Check
                    B3          B2          B4
PLAT712_ALERT_1_C ANGLE   Unknown or Inconsistent Label .........         B3 Check
                    B3          B2          B5
PLAT712_ALERT_1_C ANGLE   Unknown or Inconsistent Label .........         B3 Check
                    B3          B4          B5
PLAT712_ALERT_1_C ANGLE   Unknown or Inconsistent Label .........         B3 Check
                    B3          B4          B6
PLAT712_ALERT_1_C ANGLE   Unknown or Inconsistent Label .........         B3 Check
                    B3          B6          B4
PLAT712_ALERT_1_C ANGLE   Unknown or Inconsistent Label .........         B3 Check
                    B3          B6          B5
PLAT712_ALERT_1_C ANGLE   Unknown or Inconsistent Label .........         B4 Check
                    B4          B2          B5
PLAT712_ALERT_1_C ANGLE   Unknown or Inconsistent Label .........         B4 Check
                    B4          B3          B6
PLAT712_ALERT_1_C ANGLE   Unknown or Inconsistent Label .........         B4 Check
                    B4          B5          B6
PLAT712_ALERT_1_C ANGLE   Unknown or Inconsistent Label .........         B4 Check
                    B4          B6          B5
PLAT712_ALERT_1_C ANGLE   Unknown or Inconsistent Label .........         B5 Check
                    B5          B1          B6
PLAT712_ALERT_1_C ANGLE   Unknown or Inconsistent Label .........         B5 Check
                    B5          B4          B6
PLAT712_ALERT_1_C ANGLE   Unknown or Inconsistent Label .........         B2 Check
                    B1          B2          H2
PLAT712_ALERT_1_C ANGLE   Unknown or Inconsistent Label .........         B3 Check
                    B1          B3          H3
PLAT712_ALERT_1_C ANGLE   Unknown or Inconsistent Label .........         B5 Check
                    B1          B5          H5
PLAT712_ALERT_1_C ANGLE   Unknown or Inconsistent Label .........         B6 Check
                    B1          B6          H6
PLAT712_ALERT_1_C ANGLE   Unknown or Inconsistent Label .........         B2 Check
                    B2          B1          H1
PLAT712_ALERT_1_C ANGLE   Unknown or Inconsistent Label .........         B2 Check
                    B2          B3          H3
PLAT712_ALERT_1_C ANGLE   Unknown or Inconsistent Label .........         B2 Check
                    B2          B4          H4
PLAT712_ALERT_1_C ANGLE   Unknown or Inconsistent Label .........         B2 Check
                    B2          B5          H5
PLAT712_ALERT_1_C ANGLE   Unknown or Inconsistent Label .........         B3 Check
                    B3          B1          H1
PLAT712_ALERT_1_C ANGLE   Unknown or Inconsistent Label .........         B3 Check
                    B3          B2          H2
PLAT712_ALERT_1_C ANGLE   Unknown or Inconsistent Label .........         B3 Check
                    B3          B4          H4
```

```
PLAT712_ALERT_1_C ANGLE   Unknown or Inconsistent Label .........          B3 Check
                   B3          B6          H6
PLAT712_ALERT_1_C ANGLE   Unknown or Inconsistent Label .........          B4 Check
                   B4          B2          H2
PLAT712_ALERT_1_C ANGLE   Unknown or Inconsistent Label .........          B4 Check
                   B4          B3          H3
PLAT712_ALERT_1_C ANGLE   Unknown or Inconsistent Label .........          B4 Check
                   B4          B5          H5
PLAT712_ALERT_1_C ANGLE   Unknown or Inconsistent Label .........          B4 Check
                   B4          B6          H6
PLAT712_ALERT_1_C ANGLE   Unknown or Inconsistent Label .........          B5 Check
                   B5          B1          H1
PLAT712_ALERT_1_C ANGLE   Unknown or Inconsistent Label .........          B5 Check
                   B5          B2          H2
PLAT712_ALERT_1_C ANGLE   Unknown or Inconsistent Label .........          B5 Check
                   B5          B4          H4
PLAT712_ALERT_1_C ANGLE   Unknown or Inconsistent Label .........          B5 Check
                   B5          B6          H6
PLAT712_ALERT_1_C ANGLE   Unknown or Inconsistent Label .........          B6 Check
                   B6          B1          H1
PLAT712_ALERT_1_C ANGLE   Unknown or Inconsistent Label .........          B6 Check
                   B6          B3          H3
PLAT712_ALERT_1_C ANGLE   Unknown or Inconsistent Label .........          B6 Check
                   B6          B4          H4
PLAT712_ALERT_1_C ANGLE   Unknown or Inconsistent Label .........          B6 Check
                   B6          B5          H5
PLAT712_ALERT_1_C ANGLE   Unknown or Inconsistent Label .........         H2A Check
                   H1A         N           H2A
PLAT712_ALERT_1_C ANGLE   Unknown or Inconsistent Label .........         H3A Check
                   H1A         N           H3A
PLAT712_ALERT_1_C ANGLE   Unknown or Inconsistent Label .........         H4A Check
                   H1A         N           H4A
PLAT712_ALERT_1_C ANGLE   Unknown or Inconsistent Label .........         H2A Check
                   H2A         N           H3A
PLAT712_ALERT_1_C ANGLE   Unknown or Inconsistent Label .........         H2A Check
                   H2A         N           H4A
PLAT712_ALERT_1_C ANGLE   Unknown or Inconsistent Label .........         H3A Check
                   H3A         N           H4A
PLAT713_ALERT_1_C TORSION Unknown or Inconsistent Label .........          B2 Check
                   B1          B2          B3          B4
PLAT713_ALERT_1_C TORSION Unknown or Inconsistent Label .........          B2 Check
                   B1          B2          B3          B6
PLAT713_ALERT_1_C TORSION Unknown or Inconsistent Label .........          B2 Check
                   B1          B2          B4          B3
PLAT713_ALERT_1_C TORSION Unknown or Inconsistent Label .........          B2 Check
                   B1          B2          B4          B5
PLAT713_ALERT_1_C TORSION Unknown or Inconsistent Label .........          B2 Check
                   B1          B2          B4          B6
PLAT713_ALERT_1_C TORSION Unknown or Inconsistent Label .........          B2 Check
                   B1          B2          B5          B4
PLAT713_ALERT_1_C TORSION Unknown or Inconsistent Label .........          B2 Check
                   B1          B2          B5          B6
PLAT713_ALERT_1_C TORSION Unknown or Inconsistent Label .........          B3 Check
                   B1          B3          B2          B4
PLAT713_ALERT_1_C TORSION Unknown or Inconsistent Label .........          B3 Check
                   B1          B3          B2          B5
PLAT713_ALERT_1_C TORSION Unknown or Inconsistent Label .........          B3 Check
```

```
                   B1          B3          B4          B2
PLAT713_ALERT_1_C TORSION Unknown or Inconsistent Label ..........        B3 Check
                   B1          B3          B4          B5
PLAT713_ALERT_1_C TORSION Unknown or Inconsistent Label ..........        B3 Check
                   B1          B3          B4          B6
PLAT713_ALERT_1_C TORSION Unknown or Inconsistent Label ..........        B3 Check
                   B1          B3          B6          B4
PLAT713_ALERT_1_C TORSION Unknown or Inconsistent Label ..........        B3 Check
                   B1          B3          B6          B5
PLAT713_ALERT_1_C TORSION Unknown or Inconsistent Label ..........        B5 Check
                   B1          B5          B2          B3
PLAT713_ALERT_1_C TORSION Unknown or Inconsistent Label ..........        B5 Check
                   B1          B5          B2          B4
PLAT713_ALERT_1_C TORSION Unknown or Inconsistent Label ..........        B5 Check
                   B1          B5          B4          B2
PLAT713_ALERT_1_C TORSION Unknown or Inconsistent Label ..........        B5 Check
                   B1          B5          B4          B3
PLAT713_ALERT_1_C TORSION Unknown or Inconsistent Label ..........        B5 Check
                   B1          B5          B4          B6
PLAT713_ALERT_1_C TORSION Unknown or Inconsistent Label ..........        B5 Check
                   B1          B5          B6          B3
PLAT713_ALERT_1_C TORSION Unknown or Inconsistent Label ..........        B5 Check
                   B1          B5          B6          B4
PLAT713_ALERT_1_C TORSION Unknown or Inconsistent Label ..........        B6 Check
                   B1          B6          B3          B2
PLAT713_ALERT_1_C TORSION Unknown or Inconsistent Label ..........        B6 Check
                   B1          B6          B3          B4
PLAT713_ALERT_1_C TORSION Unknown or Inconsistent Label ..........        B6 Check
                   B1          B6          B4          B2
PLAT713_ALERT_1_C TORSION Unknown or Inconsistent Label ..........        B6 Check
                   B1          B6          B4          B3
PLAT713_ALERT_1_C TORSION Unknown or Inconsistent Label ..........        B6 Check
                   B1          B6          B4          B5
PLAT713_ALERT_1_C TORSION Unknown or Inconsistent Label ..........        B6 Check
                   B1          B6          B5          B2
PLAT713_ALERT_1_C TORSION Unknown or Inconsistent Label ..........        B6 Check
                   B1          B6          B5          B4
PLAT713_ALERT_1_C TORSION Unknown or Inconsistent Label ..........        B2 Check
                   B2          B1          B3          B4
PLAT713_ALERT_1_C TORSION Unknown or Inconsistent Label ..........        B2 Check
                   B2          B1          B3          B6
PLAT713_ALERT_1_C TORSION Unknown or Inconsistent Label ..........        B2 Check
                   B2          B1          B5          B4
PLAT713_ALERT_1_C TORSION Unknown or Inconsistent Label ..........        B2 Check
                   B2          B1          B5          B6
PLAT713_ALERT_1_C TORSION Unknown or Inconsistent Label ..........        B2 Check
                   B2          B1          B6          B3
PLAT713_ALERT_1_C TORSION Unknown or Inconsistent Label ..........        B2 Check
                   B2          B1          B6          B4
PLAT713_ALERT_1_C TORSION Unknown or Inconsistent Label ..........        B2 Check
                   B2          B1          B6          B5
PLAT713_ALERT_1_C TORSION Unknown or Inconsistent Label ..........        B2 Check
                   B2          B3          B1          B5
PLAT713_ALERT_1_C TORSION Unknown or Inconsistent Label ..........        B2 Check
                   B2          B3          B1          B6
PLAT713_ALERT_1_C TORSION Unknown or Inconsistent Label ..........        B2 Check
                   B2          B3          B4          B5
```

```
PLAT713_ALERT_1_C TORSION Unknown or Inconsistent Label ..........         B2 Check
                   B2          B3          B4          B6
PLAT713_ALERT_1_C TORSION Unknown or Inconsistent Label ..........         B2 Check
                   B2          B3          B6          B4
PLAT713_ALERT_1_C TORSION Unknown or Inconsistent Label ..........         B2 Check
                   B2          B3          B6          B5
PLAT713_ALERT_1_C TORSION Unknown or Inconsistent Label ..........         B2 Check
                   B2          B4          B3          B6
PLAT713_ALERT_1_C TORSION Unknown or Inconsistent Label ..........         B2 Check
                   B2          B4          B5          B6
PLAT713_ALERT_1_C TORSION Unknown or Inconsistent Label ..........         B2 Check
                   B2          B4          B6          B3
PLAT713_ALERT_1_C TORSION Unknown or Inconsistent Label ..........         B2 Check
                   B2          B4          B6          B5
PLAT713_ALERT_1_C TORSION Unknown or Inconsistent Label ..........         B2 Check
                   B2          B5          B1          B3
PLAT713_ALERT_1_C TORSION Unknown or Inconsistent Label ..........         B2 Check
                   B2          B5          B1          B6
PLAT713_ALERT_1_C TORSION Unknown or Inconsistent Label ..........         B2 Check
                   B2          B5          B4          B3
PLAT713_ALERT_1_C TORSION Unknown or Inconsistent Label ..........         B2 Check
                   B2          B5          B4          B6
PLAT713_ALERT_1_C TORSION Unknown or Inconsistent Label ..........         B2 Check
                   B2          B5          B6          B3
PLAT713_ALERT_1_C TORSION Unknown or Inconsistent Label ..........         B2 Check
                   B2          B5          B6          B4
PLAT713_ALERT_1_C TORSION Unknown or Inconsistent Label ..........         B3 Check
                   B3          B1          B2          B4
PLAT713_ALERT_1_C TORSION Unknown or Inconsistent Label ..........         B3 Check
                   B3          B1          B2          B5
PLAT713_ALERT_1_C TORSION Unknown or Inconsistent Label ..........         B3 Check
                   B3          B1          B5          B4
PLAT713_ALERT_1_C TORSION Unknown or Inconsistent Label ..........         B3 Check
                   B3          B1          B5          B6
PLAT713_ALERT_1_C TORSION Unknown or Inconsistent Label ..........         B3 Check
                   B3          B1          B6          B4
PLAT713_ALERT_1_C TORSION Unknown or Inconsistent Label ..........         B3 Check
                   B3          B1          B6          B5
PLAT713_ALERT_1_C TORSION Unknown or Inconsistent Label ..........         B3 Check
                   B3          B2          B1          B5
PLAT713_ALERT_1_C TORSION Unknown or Inconsistent Label ..........         B3 Check
                   B3          B2          B1          B6
PLAT713_ALERT_1_C TORSION Unknown or Inconsistent Label ..........         B3 Check
                   B3          B2          B4          B5
PLAT713_ALERT_1_C TORSION Unknown or Inconsistent Label ..........         B3 Check
                   B3          B2          B4          B6
PLAT713_ALERT_1_C TORSION Unknown or Inconsistent Label ..........         B3 Check
                   B3          B2          B5          B4
PLAT713_ALERT_1_C TORSION Unknown or Inconsistent Label ..........         B3 Check
                   B3          B2          B5          B6
PLAT713_ALERT_1_C TORSION Unknown or Inconsistent Label ..........         B3 Check
                   B3          B4          B2          B5
PLAT713_ALERT_1_C TORSION Unknown or Inconsistent Label ..........         B3 Check
                   B3          B4          B5          B6
PLAT713_ALERT_1_C TORSION Unknown or Inconsistent Label ..........         B3 Check
                   B3          B4          B6          B5
PLAT713_ALERT_1_C TORSION Unknown or Inconsistent Label ..........         B3 Check
```

```
                   B3          B6          B1          B5
PLAT713_ALERT_1_C TORSION Unknown or Inconsistent Label ..........         B3 Check
                   B3          B6          B4          B5
PLAT713_ALERT_1_C TORSION Unknown or Inconsistent Label ..........         B3 Check
                   B3          B6          B5          B4
PLAT713_ALERT_1_C TORSION Unknown or Inconsistent Label ..........         B4 Check
                   B4          B2          B1          B5
PLAT713_ALERT_1_C TORSION Unknown or Inconsistent Label ..........         B4 Check
                   B4          B2          B1          B6
PLAT713_ALERT_1_C TORSION Unknown or Inconsistent Label ..........         B4 Check
                   B4          B2          B3          B6
PLAT713_ALERT_1_C TORSION Unknown or Inconsistent Label ..........         B4 Check
                   B4          B2          B5          B6
PLAT713_ALERT_1_C TORSION Unknown or Inconsistent Label ..........         B4 Check
                   B4          B3          B1          B5
PLAT713_ALERT_1_C TORSION Unknown or Inconsistent Label ..........         B4 Check
                   B4          B3          B1          B6
PLAT713_ALERT_1_C TORSION Unknown or Inconsistent Label ..........         B4 Check
                   B4          B3          B2          B5
PLAT713_ALERT_1_C TORSION Unknown or Inconsistent Label ..........         B4 Check
                   B4          B3          B6          B5
PLAT713_ALERT_1_C TORSION Unknown or Inconsistent Label ..........         B4 Check
                   B4          B5          B1          B6
PLAT713_ALERT_1_C TORSION Unknown or Inconsistent Label ..........         B4 Check
                   B4          B6          B1          B5
PLAT713_ALERT_1_C TORSION Unknown or Inconsistent Label ..........         B5 Check
                   B5          B1          B3          B6
PLAT713_ALERT_1_C TORSION Unknown or Inconsistent Label ..........         B5 Check
                   B5          B2          B1          B6
PLAT713_ALERT_1_C TORSION Unknown or Inconsistent Label ..........         B5 Check
                   B5          B2          B3          B6
PLAT713_ALERT_1_C TORSION Unknown or Inconsistent Label ..........         B5 Check
                   B5          B2          B4          B6
PLAT713_ALERT_1_C TORSION Unknown or Inconsistent Label ..........         B5 Check
                   B5          B4          B3          B6
PLAT713_ALERT_1_C TORSION Unknown or Inconsistent Label ..........         B2 Check
                   B1          B2          B3          H3
PLAT713_ALERT_1_C TORSION Unknown or Inconsistent Label ..........         B2 Check
                   B1          B2          B4          H4
PLAT713_ALERT_1_C TORSION Unknown or Inconsistent Label ..........         B2 Check
                   B1          B2          B5          H5
PLAT713_ALERT_1_C TORSION Unknown or Inconsistent Label ..........         B3 Check
                   B1          B3          B2          H2
PLAT713_ALERT_1_C TORSION Unknown or Inconsistent Label ..........         B3 Check
                   B1          B3          B4          H4
PLAT713_ALERT_1_C TORSION Unknown or Inconsistent Label ..........         B3 Check
                   B1          B3          B6          H6
PLAT713_ALERT_1_C TORSION Unknown or Inconsistent Label ..........         B5 Check
                   B1          B5          B2          H2
PLAT713_ALERT_1_C TORSION Unknown or Inconsistent Label ..........         B5 Check
                   B1          B5          B4          H4
PLAT713_ALERT_1_C TORSION Unknown or Inconsistent Label ..........         B5 Check
                   B1          B5          B6          H6
PLAT713_ALERT_1_C TORSION Unknown or Inconsistent Label ..........         B6 Check
                   B1          B6          B3          H3
PLAT713_ALERT_1_C TORSION Unknown or Inconsistent Label ..........         B6 Check
                   B1          B6          B4          H4
```

```
PLAT713_ALERT_1_C TORSION Unknown or Inconsistent Label ..........          B6 Check
                    B1          B6          B5          H5
PLAT713_ALERT_1_C TORSION Unknown or Inconsistent Label ..........          B2 Check
                    B2          B1          B3          H3
PLAT713_ALERT_1_C TORSION Unknown or Inconsistent Label ..........          B2 Check
                    B2          B1          B5          H5
PLAT713_ALERT_1_C TORSION Unknown or Inconsistent Label ..........          B2 Check
                    B2          B1          B6          H6
PLAT713_ALERT_1_C TORSION Unknown or Inconsistent Label ..........          B2 Check
                    B2          B3          B4          H4
PLAT713_ALERT_1_C TORSION Unknown or Inconsistent Label ..........          B2 Check
                    B2          B3          B6          H6
PLAT713_ALERT_1_C TORSION Unknown or Inconsistent Label ..........          B2 Check
                    B2          B4          B3          H3
PLAT713_ALERT_1_C TORSION Unknown or Inconsistent Label ..........          B2 Check
                    B2          B4          B5          H5
PLAT713_ALERT_1_C TORSION Unknown or Inconsistent Label ..........          B2 Check
                    B2          B4          B6          H6
PLAT713_ALERT_1_C TORSION Unknown or Inconsistent Label ..........          B2 Check
                    B2          B5          B4          H4
PLAT713_ALERT_1_C TORSION Unknown or Inconsistent Label ..........          B2 Check
                    B2          B5          B6          H6
PLAT713_ALERT_1_C TORSION Unknown or Inconsistent Label ..........          B3 Check
                    B3          B1          B2          H2
PLAT713_ALERT_1_C TORSION Unknown or Inconsistent Label ..........          B3 Check
                    B3          B1          B5          H5
PLAT713_ALERT_1_C TORSION Unknown or Inconsistent Label ..........          B3 Check
                    B3          B1          B6          H6
PLAT713_ALERT_1_C TORSION Unknown or Inconsistent Label ..........          B3 Check
                    B3          B2          B4          H4
PLAT713_ALERT_1_C TORSION Unknown or Inconsistent Label ..........          B3 Check
                    B3          B2          B5          H5
PLAT713_ALERT_1_C TORSION Unknown or Inconsistent Label ..........          B3 Check
                    B3          B4          B2          H2
PLAT713_ALERT_1_C TORSION Unknown or Inconsistent Label ..........          B3 Check
                    B3          B4          B5          H5
PLAT713_ALERT_1_C TORSION Unknown or Inconsistent Label ..........          B3 Check
                    B3          B4          B6          H6
PLAT713_ALERT_1_C TORSION Unknown or Inconsistent Label ..........          B3 Check
                    B3          B6          B4          H4
PLAT713_ALERT_1_C TORSION Unknown or Inconsistent Label ..........          B3 Check
                    B3          B6          B5          H5
PLAT713_ALERT_1_C TORSION Unknown or Inconsistent Label ..........          B4 Check
                    B4          B2          B3          H3
PLAT713_ALERT_1_C TORSION Unknown or Inconsistent Label ..........          B4 Check
                    B4          B2          B5          H5
PLAT713_ALERT_1_C TORSION Unknown or Inconsistent Label ..........          B4 Check
                    B4          B3          B2          H2
PLAT713_ALERT_1_C TORSION Unknown or Inconsistent Label ..........          B4 Check
                    B4          B3          B6          H6
PLAT713_ALERT_1_C TORSION Unknown or Inconsistent Label ..........          B4 Check
                    B4          B5          B2          H2
PLAT713_ALERT_1_C TORSION Unknown or Inconsistent Label ..........          B4 Check
                    B4          B5          B6          H6
PLAT713_ALERT_1_C TORSION Unknown or Inconsistent Label ..........          B4 Check
                    B4          B6          B3          H3
PLAT713_ALERT_1_C TORSION Unknown or Inconsistent Label ..........          B4 Check
```

```
                   B4          B6          B5          H5
PLAT713_ALERT_1_C TORSION Unknown or Inconsistent Label ..........         B5 Check
                   B5          B1          B2          H2
PLAT713_ALERT_1_C TORSION Unknown or Inconsistent Label ..........         B5 Check
                   B5          B1          B3          H3
PLAT713_ALERT_1_C TORSION Unknown or Inconsistent Label ..........         B5 Check
                   B5          B1          B6          H6
PLAT713_ALERT_1_C TORSION Unknown or Inconsistent Label ..........         B5 Check
                   B5          B2          B3          H3
PLAT713_ALERT_1_C TORSION Unknown or Inconsistent Label ..........         B5 Check
                   B5          B2          B4          H4
PLAT713_ALERT_1_C TORSION Unknown or Inconsistent Label ..........         B5 Check
                   B5          B4          B2          H2
PLAT713_ALERT_1_C TORSION Unknown or Inconsistent Label ..........         B5 Check
                   B5          B4          B3          H3
PLAT713_ALERT_1_C TORSION Unknown or Inconsistent Label ..........         B5 Check
                   B5          B4          B6          H6
PLAT713_ALERT_1_C TORSION Unknown or Inconsistent Label ..........         B5 Check
                   B5          B6          B3          H3
PLAT713_ALERT_1_C TORSION Unknown or Inconsistent Label ..........         B5 Check
                   B5          B6          B4          H4
PLAT713_ALERT_1_C TORSION Unknown or Inconsistent Label ..........         B6 Check
                   B6          B1          B2          H2
PLAT713_ALERT_1_C TORSION Unknown or Inconsistent Label ..........         B6 Check
                   B6          B1          B3          H3
PLAT713_ALERT_1_C TORSION Unknown or Inconsistent Label ..........         B6 Check
                   B6          B1          B5          H5
PLAT713_ALERT_1_C TORSION Unknown or Inconsistent Label ..........         B6 Check
                   B6          B3          B2          H2
PLAT713_ALERT_1_C TORSION Unknown or Inconsistent Label ..........         B6 Check
                   B6          B3          B4          H4
PLAT713_ALERT_1_C TORSION Unknown or Inconsistent Label ..........         B6 Check
                   B6          B4          B2          H2
PLAT713_ALERT_1_C TORSION Unknown or Inconsistent Label ..........         B6 Check
                   B6          B4          B3          H3
PLAT713_ALERT_1_C TORSION Unknown or Inconsistent Label ..........         B6 Check
                   B6          B4          B5          H5
PLAT713_ALERT_1_C TORSION Unknown or Inconsistent Label ..........         B6 Check
                   B6          B5          B2          H2
PLAT713_ALERT_1_C TORSION Unknown or Inconsistent Label ..........         B6 Check
                   B6          B5          B4          H4
PLAT713_ALERT_1_C TORSION Unknown or Inconsistent Label ..........         B2 Check
                   H1          B1          B2          B3
PLAT713_ALERT_1_C TORSION Unknown or Inconsistent Label ..........         B2 Check
                   H1          B1          B2          B4
PLAT713_ALERT_1_C TORSION Unknown or Inconsistent Label ..........         B2 Check
                   H1          B1          B2          B5
PLAT713_ALERT_1_C TORSION Unknown or Inconsistent Label ..........         B3 Check
                   H1          B1          B3          B2
PLAT713_ALERT_1_C TORSION Unknown or Inconsistent Label ..........         B3 Check
                   H1          B1          B3          B4
PLAT713_ALERT_1_C TORSION Unknown or Inconsistent Label ..........         B3 Check
                   H1          B1          B3          B6
PLAT713_ALERT_1_C TORSION Unknown or Inconsistent Label ..........         B5 Check
                   H1          B1          B5          B2
PLAT713_ALERT_1_C TORSION Unknown or Inconsistent Label ..........         B5 Check
                   H1          B1          B5          B4
```

```
PLAT713_ALERT_1_C TORSION Unknown or Inconsistent Label ..........         B5 Check
                    H1          B1          B5          B6
PLAT713_ALERT_1_C TORSION Unknown or Inconsistent Label ..........         B6 Check
                    H1          B1          B6          B3
PLAT713_ALERT_1_C TORSION Unknown or Inconsistent Label ..........         B6 Check
                    H1          B1          B6          B4
PLAT713_ALERT_1_C TORSION Unknown or Inconsistent Label ..........         B6 Check
                    H1          B1          B6          B5
PLAT713_ALERT_1_C TORSION Unknown or Inconsistent Label ..........         B2 Check
                    H1          B1          B2          H2
PLAT713_ALERT_1_C TORSION Unknown or Inconsistent Label ..........         B3 Check
                    H1          B1          B3          H3
PLAT713_ALERT_1_C TORSION Unknown or Inconsistent Label ..........         B5 Check
                    H1          B1          B5          H5
PLAT713_ALERT_1_C TORSION Unknown or Inconsistent Label ..........         B6 Check
                    H1          B1          B6          H6
PLAT713_ALERT_1_C TORSION Unknown or Inconsistent Label ..........         H2 Check
                    H2          B2          B3          H3
PLAT713_ALERT_1_C TORSION Unknown or Inconsistent Label ..........         H2 Check
                    H2          B2          B4          H4
PLAT713_ALERT_1_C TORSION Unknown or Inconsistent Label ..........         H2 Check
                    H2          B2          B5          H5
PLAT713_ALERT_1_C TORSION Unknown or Inconsistent Label ..........         H3 Check
                    H3          B3          B4          H4
PLAT713_ALERT_1_C TORSION Unknown or Inconsistent Label ..........         H3 Check
                    H3          B3          B6          H6
PLAT713_ALERT_1_C TORSION Unknown or Inconsistent Label ..........         H4 Check
                    H4          B4          B5          H5
PLAT713_ALERT_1_C TORSION Unknown or Inconsistent Label ..........         H4 Check
                    H4          B4          B6          H6
PLAT713_ALERT_1_C TORSION Unknown or Inconsistent Label ..........         H5 Check
                    H5          B5          B6          H6
```

Alert level G

```
FORMU01_ALERT_2_G  There is a discrepancy between the atom counts in the
            _chemical_formula_sum and the formula from the _atom_site* data.
            Atom count from _chemical_formula_sum:H14 B6 N2
            Atom count from the _atom_site data:  H68 B36 N2
CELLZ01_ALERT_1_G Difference between formula and atom_site contents detected.
CELLZ01_ALERT_1_G ALERT: Large difference may be due to a
            symmetry error - see SYMMG tests
           From the CIF: _cell_formula_units_Z    4
           From the CIF: _chemical_formula_sum  B6 H14 N2
           TEST: Compare cell contents of formula and atom_site data

           atom    Z*formula  cif sites diff
           B         24.00    144.00 -120.00
           H         56.00    272.00 -216.00
           N          8.00      8.00    0.00
PLAT005_ALERT_5_G No Embedded Refinement Details Found  in the CIF     Please Do !
PLAT045_ALERT_1_G Calculated and Reported Z Differ by a Factor ...      0.500 Check
PLAT152_ALERT_1_G The Supplied and Calc. Volume s.u. Differ by ...          4 Units
PLAT304_ALERT_4_G Non-Integer Number of Atoms in ..... (Resd    1)       8.25 Check
PLAT304_ALERT_4_G Non-Integer Number of Atoms in ..... (Resd    2)       3.96 Check
PLAT432_ALERT_2_G Short Inter X...Y Contact  B1       ..B1       .       2.45 Ang.
                                                  1-x,1-y,z  =     33_665 Check
```

```
PLAT808_ALERT_5_G No Parseable SHELXL Style Weighting Scheme Found     Please Check
PLAT883_ALERT_1_G Absent Datum for _atom_sites_solution_primary ..     Please Do !
PLAT912_ALERT_4_G Missing # of FCF Reflections Above STh/L=  0.600         44 Note
PLAT929_ALERT_5_G No Weight Pars,Obs and Calc R1,wR2,S not Checked          ! Info
PLAT961_ALERT_5_G Dataset Contains no Negative Intensities .......     Please Check
PLAT969_ALERT_5_G The 'Henn et al.' R-Factor-gap value ..........      1.494 Note
              Predicted wR2: Based on SigI**2  3.25 or SHELX Weight  3.25
PLAT980_ALERT_1_G No Anomalous Scattering Factors Found in CIF ...     Please Check
```

```
   0 ALERT level A = Most likely a serious problem - resolve or explain
   1 ALERT level B = A potentially serious problem, consider carefully
 264 ALERT level C = Check. Ensure it is not caused by an omission or oversight
  16 ALERT level G = General information/check it is not something unexpected

 267 ALERT type 1 CIF construction/syntax error, inconsistent or missing data
   3 ALERT type 2 Indicator that the structure model may be wrong or deficient
   3 ALERT type 3 Indicator that the structure quality may be low
   3 ALERT type 4 Improvement, methodology, query or suggestion
   5 ALERT type 5 Informative message, check
```

It is advisable to attempt to resolve as many as possible of the alerts in all categories. Often the minor alerts point to easily fixed oversights, errors and omissions in your CIF or refinement strategy, so attention to these fine details can be worthwhile. In order to resolve some of the more serious problems it may be necessary to carry out additional measurements or structure refinements. However, the purpose of your study may justify the reported deviations and the more serious of these should normally be commented upon in the discussion or experimental section of a paper or in the "special_details" fields of the CIF. checkCIF was carefully designed to identify outliers and unusual parameters, but every test has its limitations and alerts that are not important in a particular case may appear. Conversely, the absence of alerts does not guarantee there are no aspects of the results needing attention. It is up to the individual to critically assess their own results and, if necessary, seek expert advice.

Publication of your CIF in IUCr journals

A basic structural check has been run on your CIF. These basic checks will be run on all CIFs submitted for publication in IUCr journals (*Acta Crystallographica*, *Journal of Applied Crystallography*, *Journal of Synchrotron Radiation*); however, if you intend to submit to *Acta Crystallographica Section C* or *E* or *IUCrData*, you should make sure that full publication checks are run on the final version of your CIF prior to submission.

Publication of your CIF in other journals

Please refer to the *Notes for Authors* of the relevant journal for any special instructions relating to CIF submission.

PLATON version of 04/06/2025; check.def file version of 30/05/2025

Datablock Bisammonium_closo-hexaborate6_HAR - ellipsoid plot

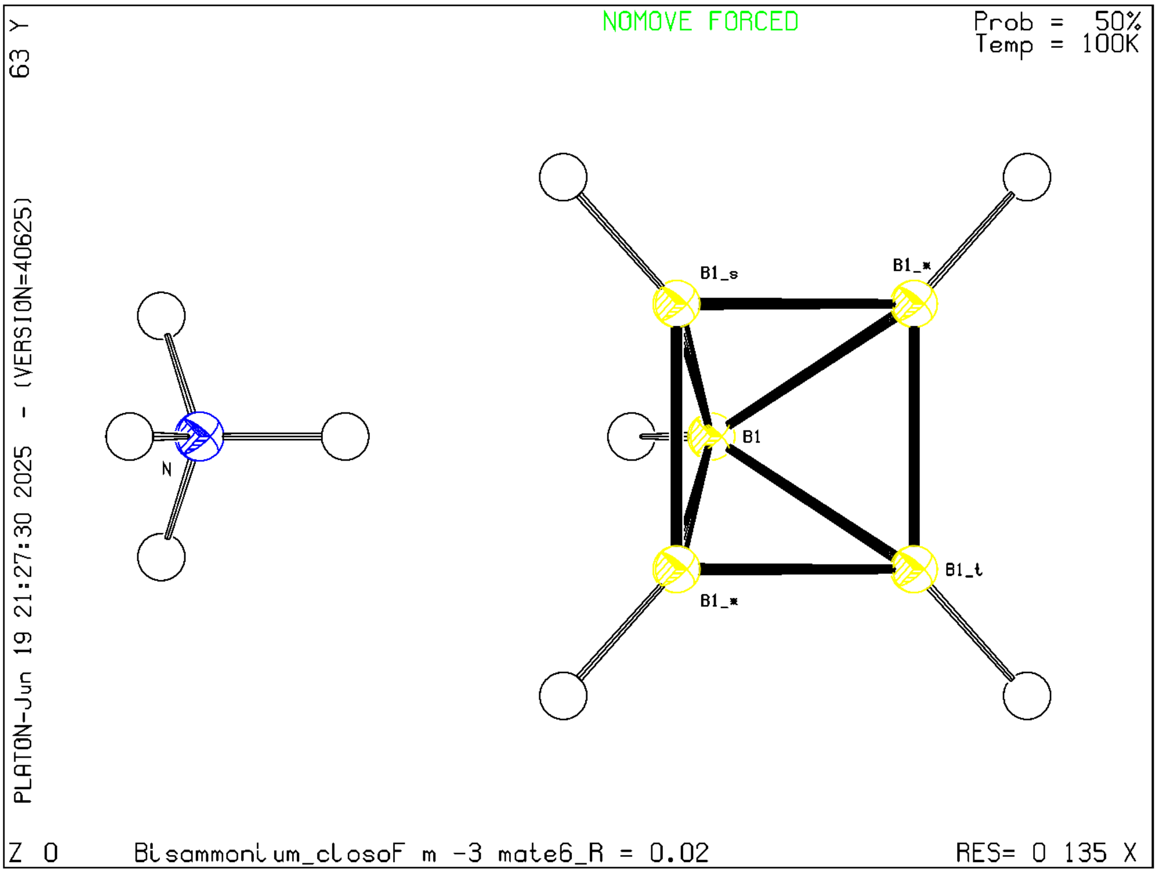

Supplement: Supplementary file 2 [file Bis(ammonium)_closo-hexaborate(6)_HAR.pdf › E_Bis(ammonium)_closo-hexaborate(6)/Bis(ammonium)_closo-hexaborate(6)_HAR.pdf]
