# Supplementary material for: Periodic Hirshfeld Atom Refinement
Source: J Phys Chem Lett. 2026 Feb 27;17(11):3170–9. doi: 10.1021/acs.jpclett.5c03918 (PMC13007019; doi:10.1021/acs.jpclett.5c03918)

checkCIF/PLATON report

Structure factors have been supplied for datablock(s) Bisammonia_arachno-decaborane12_HAR

THIS REPORT IS FOR GUIDANCE ONLY. IF USED AS PART OF A REVIEW PROCEDURE FOR PUBLICATION, IT SHOULD NOT REPLACE THE EXPERTISE OF AN EXPERIENCED CRYSTALLOGRAPHIC REFEREE.

No syntax errors found. CIF dictionary Interpreting this report

Datablock: Bisammonia_arachno-decaborane12_HAR

Bond precision: N- B = 0.0008 A Wavelength=0.51660

Cell:	a=17.860(4)	b=7.3160(15)	c=7.1770(14)
	alpha=90	beta=90	gamma=90
Temperature:	9 K		

	Calculated	Reported
Volume	937.8(3)	937.8(3)
Space group	P n m a	P n m a
Hall group	-P 2ac 2n	-P 2ac 2n
Moiety formula	B10 H18 N2	B10 H18 N2
Sum formula	B10 H18 N2	B10 H18 N2
Mr	154.26	154.27
Dx,g cm-3	1.093	1.093
Z	4	4
Mu (mm-1)	0.032	0.027
F000	328.0	328.0
F000'	327.93	
h,k,lmax	35,14,14	35,14,14
Nref	4138	1830
Tmin,Tmax	0.994,0.996	
Tmin'	0.992	

Correction method= Not given

Data completeness= 0.442 Theta(max)= 31.100

R(reflections)= 0.0232(1830) wR2(reflections)= wR= 0.0256(1830)

S = 0.364 Npar= 154

The following ALERTS were generated. Each ALERT has the format
test-name_ALERT_alert-type_alert-level.
Click on the hyperlinks for more details of the test.

Alert level A

```
GOODF01_ALERT_2_A  The least squares goodness of fit parameter lies
            outside the range 0.40 <> 6.00
            Goodness of fit given =      0.364
```

Author Response: Refer to Stefan Mebs, et al., Inorganic Chemistry 2011 50(1), 90-103 (DOI: 10.1021/ic1013158)

Alert level C

```
CELLK01_ALERT_1_C  Check that the cell measurement temperature is in Kelvin.
            Value of measurement temperature given =     9.000
REFLE01_ALERT_3_C  The _reflns_threshold_multiplier given is >= 4
            Premultiplier =    4.01
REFLE01_ALERT_3_C  The _reflns_threshold_multiplier given is >= 4
            Premultiplier =    4.01
RINTA01_ALERT_3_C  The value of Rint is greater than 0.12
            Rint given   0.152
PLAT303_ALERT_2_C Full Occupancy Atom H7        with # Connections       2.00 Check
PLAT415_ALERT_2_C Short Inter D-H..H-X       H3        ..H10      .       2.14 Ang.
                                                x,y,-1+z   =      1_554 Check
PLAT415_ALERT_2_C Short Inter D-H..H-X       H3        ..H6       .       2.14 Ang.
                                        3/2-x,1-y,-1/2+z   =      3_664 Check
PLAT415_ALERT_2_C Short Inter D-H..H-X       H3        ..H10      .       2.14 Ang.
                                            x,3/2-y,-1+z   =      8_564 Check
PLAT420_ALERT_2_C D-H Bond Without Acceptor  N2       --H11       .     Please Check
PLAT420_ALERT_2_C D-H Bond Without Acceptor  N2       --H12       .     Please Check
PLAT711_ALERT_1_C BOND    Unknown or Inconsistent Label .........        H13 Check
                    N1          H13
PLAT711_ALERT_1_C BOND    Unknown or Inconsistent Label .........        H14 Check
                    N2          H14
PLAT711_ALERT_1_C BOND    Unknown or Inconsistent Label .........         B8 Check
                    B1          B8
PLAT711_ALERT_1_C BOND    Unknown or Inconsistent Label .........         B9 Check
                    B1          B9
PLAT711_ALERT_1_C BOND    Unknown or Inconsistent Label .........         B9 Check
                    B2          B9
PLAT711_ALERT_1_C BOND    Unknown or Inconsistent Label .........         B8 Check
                    B3          B8
PLAT711_ALERT_1_C BOND    Unknown or Inconsistent Label .........        B10 Check
                    B3          B10
PLAT711_ALERT_1_C BOND    Unknown or Inconsistent Label .........         B8 Check
                    B4          B8
PLAT711_ALERT_1_C BOND    Unknown or Inconsistent Label .........         B9 Check
                    B4          B9
PLAT711_ALERT_1_C BOND    Unknown or Inconsistent Label .........         B8 Check
                    B5          B8
PLAT711_ALERT_1_C BOND    Unknown or Inconsistent Label .........        B10 Check
```

```
                   B5          B10
PLAT711_ALERT_1_C BOND    Unknown or Inconsistent Label .........        B10 Check
                   B6          B10
PLAT711_ALERT_1_C BOND    Unknown or Inconsistent Label .........         B9 Check
                   B7          B9
PLAT711_ALERT_1_C BOND    Unknown or Inconsistent Label .........         B8 Check
                   B8          B10
PLAT711_ALERT_1_C BOND    Unknown or Inconsistent Label .........        H15 Check
                   B5          H15
PLAT711_ALERT_1_C BOND    Unknown or Inconsistent Label .........         B8 Check
                   B8          H16
PLAT711_ALERT_1_C BOND    Unknown or Inconsistent Label .........         B9 Check
                   B9          H7
PLAT711_ALERT_1_C BOND    Unknown or Inconsistent Label .........         B9 Check
                   B9          H17
PLAT711_ALERT_1_C BOND    Unknown or Inconsistent Label .........        B10 Check
                   B10         H15
PLAT711_ALERT_1_C BOND    Unknown or Inconsistent Label .........        B10 Check
                   B10         H18
PLAT712_ALERT_1_C ANGLE   Unknown or Inconsistent Label .........        B10 Check
                   N1          B6          B10
PLAT712_ALERT_1_C ANGLE   Unknown or Inconsistent Label .........         B9 Check
                   N2          B7          B9
PLAT712_ALERT_1_C ANGLE   Unknown or Inconsistent Label .........        H13 Check
                   B6          N1          H13
PLAT712_ALERT_1_C ANGLE   Unknown or Inconsistent Label .........        H14 Check
                   B7          N2          H14
PLAT712_ALERT_1_C ANGLE   Unknown or Inconsistent Label .........         B8 Check
                   B1          B4          B8
PLAT712_ALERT_1_C ANGLE   Unknown or Inconsistent Label .........         B9 Check
                   B1          B4          B9
PLAT712_ALERT_1_C ANGLE   Unknown or Inconsistent Label .........         B8 Check
                   B1          B5          B8
PLAT712_ALERT_1_C ANGLE   Unknown or Inconsistent Label .........        B10 Check
                   B1          B5          B10
PLAT712_ALERT_1_C ANGLE   Unknown or Inconsistent Label .........         B9 Check
                   B1          B7          B9
PLAT712_ALERT_1_C ANGLE   Unknown or Inconsistent Label .........         B8 Check
                   B1          B8          B3
PLAT712_ALERT_1_C ANGLE   Unknown or Inconsistent Label .........         B8 Check
                   B1          B8          B4
PLAT712_ALERT_1_C ANGLE   Unknown or Inconsistent Label .........         B8 Check
                   B1          B8          B5
PLAT712_ALERT_1_C ANGLE   Unknown or Inconsistent Label .........         B8 Check
                   B1          B8          B10
PLAT712_ALERT_1_C ANGLE   Unknown or Inconsistent Label .........         B9 Check
                   B1          B9          B2
PLAT712_ALERT_1_C ANGLE   Unknown or Inconsistent Label .........         B9 Check
                   B1          B9          B4
PLAT712_ALERT_1_C ANGLE   Unknown or Inconsistent Label .........         B9 Check
                   B1          B9          B7
PLAT712_ALERT_1_C ANGLE   Unknown or Inconsistent Label .........         B8 Check
                   B2          B3          B8
PLAT712_ALERT_1_C ANGLE   Unknown or Inconsistent Label .........        B10 Check
                   B2          B3          B10
PLAT712_ALERT_1_C ANGLE   Unknown or Inconsistent Label .........         B8 Check
                   B2          B4          B8
```

PLAT712_ALERT_1_C ANGLE Unknown or Inconsistent Label .... B9 Check
B2 B4 B9
PLAT712_ALERT_1_C ANGLE Unknown or Inconsistent Label .... B10 Check
B2 B6 B10
PLAT712_ALERT_1_C ANGLE Unknown or Inconsistent Label .... B9 Check
B2 B9 B4
PLAT712_ALERT_1_C ANGLE Unknown or Inconsistent Label .... B9 Check
B2 B9 B7
PLAT712_ALERT_1_C ANGLE Unknown or Inconsistent Label .... B9 Check
B3 B2 B9
PLAT712_ALERT_1_C ANGLE Unknown or Inconsistent Label .... B8 Check
B3 B4 B8
PLAT712_ALERT_1_C ANGLE Unknown or Inconsistent Label .... B9 Check
B3 B4 B9
PLAT712_ALERT_1_C ANGLE Unknown or Inconsistent Label .... B10 Check
B3 B6 B10
PLAT712_ALERT_1_C ANGLE Unknown or Inconsistent Label .... B8 Check
B3 B8 B4
PLAT712_ALERT_1_C ANGLE Unknown or Inconsistent Label .... B8 Check
B3 B8 B5
PLAT712_ALERT_1_C ANGLE Unknown or Inconsistent Label .... B8 Check
B3 B8 B10
PLAT712_ALERT_1_C ANGLE Unknown or Inconsistent Label .... B10 Check
B3 B10 B5
PLAT712_ALERT_1_C ANGLE Unknown or Inconsistent Label .... B10 Check
B3 B10 B6
PLAT712_ALERT_1_C ANGLE Unknown or Inconsistent Label .... B10 Check
B3 B10 B8
PLAT712_ALERT_1_C ANGLE Unknown or Inconsistent Label .... B8 Check
B4 B1 B8
PLAT712_ALERT_1_C ANGLE Unknown or Inconsistent Label .... B9 Check
B4 B1 B9
PLAT712_ALERT_1_C ANGLE Unknown or Inconsistent Label .... B9 Check
B4 B2 B9
PLAT712_ALERT_1_C ANGLE Unknown or Inconsistent Label .... B8 Check
B4 B3 B8
PLAT712_ALERT_1_C ANGLE Unknown or Inconsistent Label .... B10 Check
B4 B3 B10
PLAT712_ALERT_1_C ANGLE Unknown or Inconsistent Label .... B8 Check
B4 B8 B5
PLAT712_ALERT_1_C ANGLE Unknown or Inconsistent Label .... B8 Check
B4 B8 B10
PLAT712_ALERT_1_C ANGLE Unknown or Inconsistent Label .... B9 Check
B4 B9 B7
PLAT712_ALERT_1_C ANGLE Unknown or Inconsistent Label .... B8 Check
B5 B1 B8
PLAT712_ALERT_1_C ANGLE Unknown or Inconsistent Label .... B9 Check
B5 B1 B9
PLAT712_ALERT_1_C ANGLE Unknown or Inconsistent Label .... B9 Check
B5 B7 B9
PLAT712_ALERT_1_C ANGLE Unknown or Inconsistent Label .... B8 Check
B5 B8 B10
PLAT712_ALERT_1_C ANGLE Unknown or Inconsistent Label .... B10 Check
B5 B10 B6
PLAT712_ALERT_1_C ANGLE Unknown or Inconsistent Label .... B10 Check
B5 B10 B8
PLAT712_ALERT_1_C ANGLE Unknown or Inconsistent Label .... B9 Check

```
                    B6          B2          B9
PLAT712_ALERT_1_C ANGLE   Unknown or Inconsistent Label .........          B8 Check
                    B6          B3          B8
PLAT712_ALERT_1_C ANGLE   Unknown or Inconsistent Label .........         B10 Check
                    B6          B3          B10
PLAT712_ALERT_1_C ANGLE   Unknown or Inconsistent Label .........         B10 Check
                    B6          B10         B8
PLAT712_ALERT_1_C ANGLE   Unknown or Inconsistent Label .........          B8 Check
                    B7          B1          B8
PLAT712_ALERT_1_C ANGLE   Unknown or Inconsistent Label .........          B9 Check
                    B7          B1          B9
PLAT712_ALERT_1_C ANGLE   Unknown or Inconsistent Label .........          B8 Check
                    B7          B5          B8
PLAT712_ALERT_1_C ANGLE   Unknown or Inconsistent Label .........         B10 Check
                    B7          B5          B10
PLAT712_ALERT_1_C ANGLE   Unknown or Inconsistent Label .........          B8 Check
                    B8          B1          B9
PLAT712_ALERT_1_C ANGLE   Unknown or Inconsistent Label .........          B8 Check
                    B8          B3          B10
PLAT712_ALERT_1_C ANGLE   Unknown or Inconsistent Label .........          B8 Check
                    B8          B4          B9
PLAT712_ALERT_1_C ANGLE   Unknown or Inconsistent Label .........          B8 Check
                    B8          B5          B10
PLAT712_ALERT_1_C ANGLE   Unknown or Inconsistent Label .........         H15 Check
                    B1          B5          H15
PLAT712_ALERT_1_C ANGLE   Unknown or Inconsistent Label .........          B8 Check
                    B1          B8          H16
PLAT712_ALERT_1_C ANGLE   Unknown or Inconsistent Label .........          B9 Check
                    B1          B9          H7
PLAT712_ALERT_1_C ANGLE   Unknown or Inconsistent Label .........          B9 Check
                    B1          B9          H17
PLAT712_ALERT_1_C ANGLE   Unknown or Inconsistent Label .........          B9 Check
                    B2          B9          H7
PLAT712_ALERT_1_C ANGLE   Unknown or Inconsistent Label .........          B9 Check
                    B2          B9          H17
PLAT712_ALERT_1_C ANGLE   Unknown or Inconsistent Label .........          B8 Check
                    B3          B8          H16
PLAT712_ALERT_1_C ANGLE   Unknown or Inconsistent Label .........         B10 Check
                    B3          B10         H15
PLAT712_ALERT_1_C ANGLE   Unknown or Inconsistent Label .........         B10 Check
                    B3          B10         H18
PLAT712_ALERT_1_C ANGLE   Unknown or Inconsistent Label .........          B8 Check
                    B4          B8          H16
PLAT712_ALERT_1_C ANGLE   Unknown or Inconsistent Label .........          B9 Check
                    B4          B9          H7
PLAT712_ALERT_1_C ANGLE   Unknown or Inconsistent Label .........          B9 Check
                    B4          B9          H17
PLAT712_ALERT_1_C ANGLE   Unknown or Inconsistent Label .........          B8 Check
                    B5          B8          H16
PLAT712_ALERT_1_C ANGLE   Unknown or Inconsistent Label .........         B10 Check
                    B5          B10         H15
PLAT712_ALERT_1_C ANGLE   Unknown or Inconsistent Label .........         B10 Check
                    B5          B10         H18
PLAT712_ALERT_1_C ANGLE   Unknown or Inconsistent Label .........         B10 Check
                    B6          B10         H15
PLAT712_ALERT_1_C ANGLE   Unknown or Inconsistent Label .........         B10 Check
                    B6          B10         H18
```

```
PLAT712_ALERT_1_C ANGLE   Unknown or Inconsistent Label .........         H15 Check
                    B7          B5          H15
PLAT712_ALERT_1_C ANGLE   Unknown or Inconsistent Label .........          B9 Check
                    B7          B9          H7
PLAT712_ALERT_1_C ANGLE   Unknown or Inconsistent Label .........          B9 Check
                    B7          B9          H17
PLAT712_ALERT_1_C ANGLE   Unknown or Inconsistent Label .........          B8 Check
                    B8          B1          H5
PLAT712_ALERT_1_C ANGLE   Unknown or Inconsistent Label .........          B8 Check
                    B8          B3          H1
PLAT712_ALERT_1_C ANGLE   Unknown or Inconsistent Label .........          B8 Check
                    B8          B4          H3
PLAT712_ALERT_1_C ANGLE   Unknown or Inconsistent Label .........          B8 Check
                    B8          B5          H2
PLAT712_ALERT_1_C ANGLE   Unknown or Inconsistent Label .........          B8 Check
                    B8          B5          H15
PLAT712_ALERT_1_C ANGLE   Unknown or Inconsistent Label .........          B8 Check
                    B8          B10         H15
PLAT712_ALERT_1_C ANGLE   Unknown or Inconsistent Label .........          B8 Check
                    B8          B10         H18
PLAT712_ALERT_1_C ANGLE   Unknown or Inconsistent Label .........          B9 Check
                    B9          B1          H5
PLAT712_ALERT_1_C ANGLE   Unknown or Inconsistent Label .........          B9 Check
                    B9          B2          H7
PLAT712_ALERT_1_C ANGLE   Unknown or Inconsistent Label .........          B9 Check
                    B9          B2          H8
PLAT712_ALERT_1_C ANGLE   Unknown or Inconsistent Label .........          B9 Check
                    B9          B4          H3
PLAT712_ALERT_1_C ANGLE   Unknown or Inconsistent Label .........          B9 Check
                    B9          B7          H4
PLAT712_ALERT_1_C ANGLE   Unknown or Inconsistent Label .........         B10 Check
                    B10         B3          H1
PLAT712_ALERT_1_C ANGLE   Unknown or Inconsistent Label .........         B10 Check
                    B10         B5          H2
PLAT712_ALERT_1_C ANGLE   Unknown or Inconsistent Label .........         B10 Check
                    B10         B5          H15
PLAT712_ALERT_1_C ANGLE   Unknown or Inconsistent Label .........         B10 Check
                    B10         B6          H9
PLAT712_ALERT_1_C ANGLE   Unknown or Inconsistent Label .........         B10 Check
                    B10         B8          H16
PLAT712_ALERT_1_C ANGLE   Unknown or Inconsistent Label .........          B9 Check
                    B2          H7          B9
PLAT712_ALERT_1_C ANGLE   Unknown or Inconsistent Label .........         H15 Check
                    B5          H15         B10
PLAT712_ALERT_1_C ANGLE   Unknown or Inconsistent Label .........         H13 Check
                    H6          N1          H13
PLAT712_ALERT_1_C ANGLE   Unknown or Inconsistent Label .........         H13 Check
                    H10         N1          H13
PLAT712_ALERT_1_C ANGLE   Unknown or Inconsistent Label .........         H14 Check
                    H11         N2          H14
PLAT712_ALERT_1_C ANGLE   Unknown or Inconsistent Label .........         H14 Check
                    H12         N2          H14
PLAT712_ALERT_1_C ANGLE   Unknown or Inconsistent Label .........         H15 Check
                    H2          B5          H15
PLAT712_ALERT_1_C ANGLE   Unknown or Inconsistent Label .........          B9 Check
                    H7          B9          H17
PLAT712_ALERT_1_C ANGLE   Unknown or Inconsistent Label .........         H15 Check
```

```
                    H15         B10         H18
PLAT713_ALERT_1_C TORSION Unknown or Inconsistent Label ..........         B9 Check
                    N1          B6          B2          B9
PLAT713_ALERT_1_C TORSION Unknown or Inconsistent Label ..........         B8 Check
                    N1          B6          B3          B8
PLAT713_ALERT_1_C TORSION Unknown or Inconsistent Label ..........        B10 Check
                    N1          B6          B3          B10
PLAT713_ALERT_1_C TORSION Unknown or Inconsistent Label ..........        B10 Check
                    N1          B6          B10         B3
PLAT713_ALERT_1_C TORSION Unknown or Inconsistent Label ..........        B10 Check
                    N1          B6          B10         B5
PLAT713_ALERT_1_C TORSION Unknown or Inconsistent Label ..........        B10 Check
                    N1          B6          B10         B8
PLAT713_ALERT_1_C TORSION Unknown or Inconsistent Label ..........         B8 Check
                    N2          B7          B1          B8
PLAT713_ALERT_1_C TORSION Unknown or Inconsistent Label ..........         B9 Check
                    N2          B7          B1          B9
PLAT713_ALERT_1_C TORSION Unknown or Inconsistent Label ..........         B8 Check
                    N2          B7          B5          B8
PLAT713_ALERT_1_C TORSION Unknown or Inconsistent Label ..........        B10 Check
                    N2          B7          B5          B10
PLAT713_ALERT_1_C TORSION Unknown or Inconsistent Label ..........         B9 Check
                    N2          B7          B9          B1
PLAT713_ALERT_1_C TORSION Unknown or Inconsistent Label ..........         B9 Check
                    N2          B7          B9          B2
PLAT713_ALERT_1_C TORSION Unknown or Inconsistent Label ..........         B9 Check
                    N2          B7          B9          B4
PLAT713_ALERT_1_C TORSION Unknown or Inconsistent Label ..........        B10 Check
                    N1          B6          B10         H15
PLAT713_ALERT_1_C TORSION Unknown or Inconsistent Label ..........        B10 Check
                    N1          B6          B10         H18
PLAT713_ALERT_1_C TORSION Unknown or Inconsistent Label ..........        H15 Check
                    N2          B7          B5          H15
PLAT713_ALERT_1_C TORSION Unknown or Inconsistent Label ..........         B9 Check
                    N2          B7          B9          H7
PLAT713_ALERT_1_C TORSION Unknown or Inconsistent Label ..........         B9 Check
                    N2          B7          B9          H17
PLAT713_ALERT_1_C TORSION Unknown or Inconsistent Label ..........        H14 Check
                    B1          B7          N2          H14
PLAT713_ALERT_1_C TORSION Unknown or Inconsistent Label ..........        H13 Check
                    B2          B6          N1          H13
PLAT713_ALERT_1_C TORSION Unknown or Inconsistent Label ..........        H13 Check
                    B3          B6          N1          H13
PLAT713_ALERT_1_C TORSION Unknown or Inconsistent Label ..........        H14 Check
                    B5          B7          N2          H14
PLAT713_ALERT_1_C TORSION Unknown or Inconsistent Label ..........         B9 Check
                    B1          B4          B2          B9
PLAT713_ALERT_1_C TORSION Unknown or Inconsistent Label ..........         B8 Check
                    B1          B4          B3          B8
PLAT713_ALERT_1_C TORSION Unknown or Inconsistent Label ..........        B10 Check
                    B1          B4          B3          B10
PLAT713_ALERT_1_C TORSION Unknown or Inconsistent Label ..........         B8 Check
                    B1          B4          B8          B3
PLAT713_ALERT_1_C TORSION Unknown or Inconsistent Label ..........         B8 Check
                    B1          B4          B8          B5
PLAT713_ALERT_1_C TORSION Unknown or Inconsistent Label ..........         B8 Check
                    B1          B4          B8          B10
```

```
PLAT713_ALERT_1_C TORSION Unknown or Inconsistent Label ..........          B9 Check
                    B1          B4          B9          B2
PLAT713_ALERT_1_C TORSION Unknown or Inconsistent Label ..........          B9 Check
                    B1          B4          B9          B7
PLAT713_ALERT_1_C TORSION Unknown or Inconsistent Label ..........          B9 Check
                    B1          B5          B7          B9
PLAT713_ALERT_1_C TORSION Unknown or Inconsistent Label ..........          B8 Check
                    B1          B5          B8          B3
PLAT713_ALERT_1_C TORSION Unknown or Inconsistent Label ..........          B8 Check
                    B1          B5          B8          B4
PLAT713_ALERT_1_C TORSION Unknown or Inconsistent Label ..........          B8 Check
                    B1          B5          B8          B10
PLAT713_ALERT_1_C TORSION Unknown or Inconsistent Label ..........         B10 Check
                    B1          B5          B10         B3
PLAT713_ALERT_1_C TORSION Unknown or Inconsistent Label ..........         B10 Check
                    B1          B5          B10         B6
PLAT713_ALERT_1_C TORSION Unknown or Inconsistent Label ..........         B10 Check
                    B1          B5          B10         B8
PLAT713_ALERT_1_C TORSION Unknown or Inconsistent Label ..........          B8 Check
                    B1          B7          B5          B8
PLAT713_ALERT_1_C TORSION Unknown or Inconsistent Label ..........         B10 Check
                    B1          B7          B5          B10
PLAT713_ALERT_1_C TORSION Unknown or Inconsistent Label ..........          B9 Check
                    B1          B7          B9          B2
PLAT713_ALERT_1_C TORSION Unknown or Inconsistent Label ..........          B9 Check
                    B1          B7          B9          B4
PLAT713_ALERT_1_C TORSION Unknown or Inconsistent Label ..........          B8 Check
                    B1          B8          B3          B2
PLAT713_ALERT_1_C TORSION Unknown or Inconsistent Label ..........          B8 Check
                    B1          B8          B3          B4
PLAT713_ALERT_1_C TORSION Unknown or Inconsistent Label ..........          B8 Check
                    B1          B8          B3          B6
PLAT713_ALERT_1_C TORSION Unknown or Inconsistent Label ..........          B8 Check
                    B1          B8          B3          B10
PLAT713_ALERT_1_C TORSION Unknown or Inconsistent Label ..........          B8 Check
                    B1          B8          B4          B2
PLAT713_ALERT_1_C TORSION Unknown or Inconsistent Label ..........          B8 Check
                    B1          B8          B4          B3
PLAT713_ALERT_1_C TORSION Unknown or Inconsistent Label ..........          B8 Check
                    B1          B8          B4          B9
PLAT713_ALERT_1_C TORSION Unknown or Inconsistent Label ..........          B8 Check
                    B1          B8          B5          B7
PLAT713_ALERT_1_C TORSION Unknown or Inconsistent Label ..........          B8 Check
                    B1          B8          B5          B10
PLAT713_ALERT_1_C TORSION Unknown or Inconsistent Label ..........          B8 Check
                    B1          B8          B10         B3
PLAT713_ALERT_1_C TORSION Unknown or Inconsistent Label ..........          B8 Check
                    B1          B8          B10         B5
PLAT713_ALERT_1_C TORSION Unknown or Inconsistent Label ..........          B8 Check
                    B1          B8          B10         B6
PLAT713_ALERT_1_C TORSION Unknown or Inconsistent Label ..........          B9 Check
                    B1          B9          B2          B3
PLAT713_ALERT_1_C TORSION Unknown or Inconsistent Label ..........          B9 Check
                    B1          B9          B2          B4
PLAT713_ALERT_1_C TORSION Unknown or Inconsistent Label ..........          B9 Check
                    B1          B9          B2          B6
PLAT713_ALERT_1_C TORSION Unknown or Inconsistent Label ..........          B9 Check
```

```
                   B1          B9          B4          B2
PLAT713_ALERT_1_C TORSION Unknown or Inconsistent Label ..........          B9 Check
                   B1          B9          B4          B3
PLAT713_ALERT_1_C TORSION Unknown or Inconsistent Label ..........          B9 Check
                   B1          B9          B4          B8
PLAT713_ALERT_1_C TORSION Unknown or Inconsistent Label ..........          B9 Check
                   B1          B9          B7          B5
PLAT713_ALERT_1_C TORSION Unknown or Inconsistent Label ..........          B8 Check
                   B2          B3          B4          B8
PLAT713_ALERT_1_C TORSION Unknown or Inconsistent Label ..........          B9 Check
                   B2          B3          B4          B9
PLAT713_ALERT_1_C TORSION Unknown or Inconsistent Label ..........         B10 Check
                   B2          B3          B6          B10
PLAT713_ALERT_1_C TORSION Unknown or Inconsistent Label ..........          B8 Check
                   B2          B3          B8          B4
PLAT713_ALERT_1_C TORSION Unknown or Inconsistent Label ..........          B8 Check
                   B2          B3          B8          B5
PLAT713_ALERT_1_C TORSION Unknown or Inconsistent Label ..........          B8 Check
                   B2          B3          B8          B10
PLAT713_ALERT_1_C TORSION Unknown or Inconsistent Label ..........         B10 Check
                   B2          B3          B10         B5
PLAT713_ALERT_1_C TORSION Unknown or Inconsistent Label ..........         B10 Check
                   B2          B3          B10         B6
PLAT713_ALERT_1_C TORSION Unknown or Inconsistent Label ..........         B10 Check
                   B2          B3          B10         B8
PLAT713_ALERT_1_C TORSION Unknown or Inconsistent Label ..........          B8 Check
                   B2          B4          B1          B8
PLAT713_ALERT_1_C TORSION Unknown or Inconsistent Label ..........          B9 Check
                   B2          B4          B1          B9
PLAT713_ALERT_1_C TORSION Unknown or Inconsistent Label ..........          B8 Check
                   B2          B4          B3          B8
PLAT713_ALERT_1_C TORSION Unknown or Inconsistent Label ..........         B10 Check
                   B2          B4          B3          B10
PLAT713_ALERT_1_C TORSION Unknown or Inconsistent Label ..........          B8 Check
                   B2          B4          B8          B3
PLAT713_ALERT_1_C TORSION Unknown or Inconsistent Label ..........          B8 Check
                   B2          B4          B8          B5
PLAT713_ALERT_1_C TORSION Unknown or Inconsistent Label ..........          B8 Check
                   B2          B4          B8          B10
PLAT713_ALERT_1_C TORSION Unknown or Inconsistent Label ..........          B9 Check
                   B2          B4          B9          B7
PLAT713_ALERT_1_C TORSION Unknown or Inconsistent Label ..........          B8 Check
                   B2          B6          B3          B8
PLAT713_ALERT_1_C TORSION Unknown or Inconsistent Label ..........         B10 Check
                   B2          B6          B3          B10
PLAT713_ALERT_1_C TORSION Unknown or Inconsistent Label ..........         B10 Check
                   B2          B6          B10         B3
PLAT713_ALERT_1_C TORSION Unknown or Inconsistent Label ..........         B10 Check
                   B2          B6          B10         B5
PLAT713_ALERT_1_C TORSION Unknown or Inconsistent Label ..........         B10 Check
                   B2          B6          B10         B8
PLAT713_ALERT_1_C TORSION Unknown or Inconsistent Label ..........          B9 Check
                   B2          B9          B1          B4
PLAT713_ALERT_1_C TORSION Unknown or Inconsistent Label ..........          B9 Check
                   B2          B9          B1          B5
PLAT713_ALERT_1_C TORSION Unknown or Inconsistent Label ..........          B9 Check
                   B2          B9          B1          B7
```

```
PLAT713_ALERT_1_C TORSION Unknown or Inconsistent Label ..........          B9 Check
                    B2          B9          B1          B8
PLAT713_ALERT_1_C TORSION Unknown or Inconsistent Label ..........          B9 Check
                    B2          B9          B4          B3
PLAT713_ALERT_1_C TORSION Unknown or Inconsistent Label ..........          B9 Check
                    B2          B9          B4          B8
PLAT713_ALERT_1_C TORSION Unknown or Inconsistent Label ..........          B9 Check
                    B2          B9          B7          B5
PLAT713_ALERT_1_C TORSION Unknown or Inconsistent Label ..........          B8 Check
                    B3          B2          B4          B8
PLAT713_ALERT_1_C TORSION Unknown or Inconsistent Label ..........          B9 Check
                    B3          B2          B4          B9
PLAT713_ALERT_1_C TORSION Unknown or Inconsistent Label ..........         B10 Check
                    B3          B2          B6          B10
PLAT713_ALERT_1_C TORSION Unknown or Inconsistent Label ..........          B9 Check
                    B3          B2          B9          B4
PLAT713_ALERT_1_C TORSION Unknown or Inconsistent Label ..........          B9 Check
                    B3          B2          B9          B7
PLAT713_ALERT_1_C TORSION Unknown or Inconsistent Label ..........          B8 Check
                    B3          B4          B1          B8
PLAT713_ALERT_1_C TORSION Unknown or Inconsistent Label ..........          B9 Check
                    B3          B4          B1          B9
PLAT713_ALERT_1_C TORSION Unknown or Inconsistent Label ..........          B9 Check
                    B3          B4          B2          B9
PLAT713_ALERT_1_C TORSION Unknown or Inconsistent Label ..........          B8 Check
                    B3          B4          B8          B5
PLAT713_ALERT_1_C TORSION Unknown or Inconsistent Label ..........          B8 Check
                    B3          B4          B8          B10
PLAT713_ALERT_1_C TORSION Unknown or Inconsistent Label ..........          B9 Check
                    B3          B4          B9          B7
PLAT713_ALERT_1_C TORSION Unknown or Inconsistent Label ..........          B9 Check
                    B3          B6          B2          B9
PLAT713_ALERT_1_C TORSION Unknown or Inconsistent Label ..........         B10 Check
                    B3          B6          B10         B5
PLAT713_ALERT_1_C TORSION Unknown or Inconsistent Label ..........         B10 Check
                    B3          B6          B10         B8
PLAT713_ALERT_1_C TORSION Unknown or Inconsistent Label ..........          B8 Check
                    B3          B8          B1          B4
PLAT713_ALERT_1_C TORSION Unknown or Inconsistent Label ..........          B8 Check
                    B3          B8          B1          B5
PLAT713_ALERT_1_C TORSION Unknown or Inconsistent Label ..........          B8 Check
                    B3          B8          B1          B7
PLAT713_ALERT_1_C TORSION Unknown or Inconsistent Label ..........          B8 Check
                    B3          B8          B1          B9
PLAT713_ALERT_1_C TORSION Unknown or Inconsistent Label ..........          B8 Check
                    B3          B8          B4          B9
PLAT713_ALERT_1_C TORSION Unknown or Inconsistent Label ..........          B8 Check
                    B3          B8          B5          B7
PLAT713_ALERT_1_C TORSION Unknown or Inconsistent Label ..........          B8 Check
                    B3          B8          B5          B10
PLAT713_ALERT_1_C TORSION Unknown or Inconsistent Label ..........          B8 Check
                    B3          B8          B10         B5
PLAT713_ALERT_1_C TORSION Unknown or Inconsistent Label ..........          B8 Check
                    B3          B8          B10         B6
PLAT713_ALERT_1_C TORSION Unknown or Inconsistent Label ..........         B10 Check
                    B3          B10         B5          B7
PLAT713_ALERT_1_C TORSION Unknown or Inconsistent Label ..........         B10 Check
```

```
                    B3          B10         B5          B8
PLAT713_ALERT_1_C TORSION Unknown or Inconsistent Label ..........        B10 Check
                    B3          B10         B8          B4
PLAT713_ALERT_1_C TORSION Unknown or Inconsistent Label ..........        B10 Check
                    B3          B10         B8          B5
PLAT713_ALERT_1_C TORSION Unknown or Inconsistent Label ..........         B8 Check
                    B4          B1          B5          B8
PLAT713_ALERT_1_C TORSION Unknown or Inconsistent Label ..........        B10 Check
                    B4          B1          B5          B10
PLAT713_ALERT_1_C TORSION Unknown or Inconsistent Label ..........         B9 Check
                    B4          B1          B7          B9
PLAT713_ALERT_1_C TORSION Unknown or Inconsistent Label ..........         B8 Check
                    B4          B1          B8          B5
PLAT713_ALERT_1_C TORSION Unknown or Inconsistent Label ..........         B8 Check
                    B4          B1          B8          B10
PLAT713_ALERT_1_C TORSION Unknown or Inconsistent Label ..........         B9 Check
                    B4          B1          B9          B7
PLAT713_ALERT_1_C TORSION Unknown or Inconsistent Label ..........         B8 Check
                    B4          B2          B3          B8
PLAT713_ALERT_1_C TORSION Unknown or Inconsistent Label ..........        B10 Check
                    B4          B2          B3          B10
PLAT713_ALERT_1_C TORSION Unknown or Inconsistent Label ..........        B10 Check
                    B4          B2          B6          B10
PLAT713_ALERT_1_C TORSION Unknown or Inconsistent Label ..........         B9 Check
                    B4          B2          B9          B7
PLAT713_ALERT_1_C TORSION Unknown or Inconsistent Label ..........         B9 Check
                    B4          B3          B2          B9
PLAT713_ALERT_1_C TORSION Unknown or Inconsistent Label ..........        B10 Check
                    B4          B3          B6          B10
PLAT713_ALERT_1_C TORSION Unknown or Inconsistent Label ..........         B8 Check
                    B4          B3          B8          B5
PLAT713_ALERT_1_C TORSION Unknown or Inconsistent Label ..........         B8 Check
                    B4          B3          B8          B10
PLAT713_ALERT_1_C TORSION Unknown or Inconsistent Label ..........        B10 Check
                    B4          B3          B10         B5
PLAT713_ALERT_1_C TORSION Unknown or Inconsistent Label ..........        B10 Check
                    B4          B3          B10         B6
PLAT713_ALERT_1_C TORSION Unknown or Inconsistent Label ..........        B10 Check
                    B4          B3          B10         B8
PLAT713_ALERT_1_C TORSION Unknown or Inconsistent Label ..........         B8 Check
                    B4          B8          B1          B5
PLAT713_ALERT_1_C TORSION Unknown or Inconsistent Label ..........         B8 Check
                    B4          B8          B1          B7
PLAT713_ALERT_1_C TORSION Unknown or Inconsistent Label ..........         B8 Check
                    B4          B8          B1          B9
PLAT713_ALERT_1_C TORSION Unknown or Inconsistent Label ..........         B8 Check
                    B4          B8          B3          B6
PLAT713_ALERT_1_C TORSION Unknown or Inconsistent Label ..........         B8 Check
                    B4          B8          B3          B10
PLAT713_ALERT_1_C TORSION Unknown or Inconsistent Label ..........         B8 Check
                    B4          B8          B5          B7
PLAT713_ALERT_1_C TORSION Unknown or Inconsistent Label ..........         B8 Check
                    B4          B8          B5          B10
PLAT713_ALERT_1_C TORSION Unknown or Inconsistent Label ..........         B8 Check
                    B4          B8          B10         B5
PLAT713_ALERT_1_C TORSION Unknown or Inconsistent Label ..........         B8 Check
                    B4          B8          B10         B6
```

```
PLAT713_ALERT_1_C TORSION Unknown or Inconsistent Label ..........          B9 Check
                    B4          B9          B1          B5
PLAT713_ALERT_1_C TORSION Unknown or Inconsistent Label ..........          B9 Check
                    B4          B9          B1          B7
PLAT713_ALERT_1_C TORSION Unknown or Inconsistent Label ..........          B9 Check
                    B4          B9          B1          B8
PLAT713_ALERT_1_C TORSION Unknown or Inconsistent Label ..........          B9 Check
                    B4          B9          B2          B6
PLAT713_ALERT_1_C TORSION Unknown or Inconsistent Label ..........          B9 Check
                    B4          B9          B7          B5
PLAT713_ALERT_1_C TORSION Unknown or Inconsistent Label ..........          B8 Check
                    B5          B1          B4          B8
PLAT713_ALERT_1_C TORSION Unknown or Inconsistent Label ..........          B9 Check
                    B5          B1          B4          B9
PLAT713_ALERT_1_C TORSION Unknown or Inconsistent Label ..........          B9 Check
                    B5          B1          B7          B9
PLAT713_ALERT_1_C TORSION Unknown or Inconsistent Label ..........          B8 Check
                    B5          B1          B8          B10
PLAT713_ALERT_1_C TORSION Unknown or Inconsistent Label ..........          B9 Check
                    B5          B1          B9          B7
PLAT713_ALERT_1_C TORSION Unknown or Inconsistent Label ..........          B8 Check
                    B5          B7          B1          B8
PLAT713_ALERT_1_C TORSION Unknown or Inconsistent Label ..........          B9 Check
                    B5          B7          B1          B9
PLAT713_ALERT_1_C TORSION Unknown or Inconsistent Label ..........          B8 Check
                    B5          B8          B1          B7
PLAT713_ALERT_1_C TORSION Unknown or Inconsistent Label ..........          B8 Check
                    B5          B8          B1          B9
PLAT713_ALERT_1_C TORSION Unknown or Inconsistent Label ..........          B8 Check
                    B5          B8          B3          B6
PLAT713_ALERT_1_C TORSION Unknown or Inconsistent Label ..........          B8 Check
                    B5          B8          B3          B10
PLAT713_ALERT_1_C TORSION Unknown or Inconsistent Label ..........          B8 Check
                    B5          B8          B4          B9
PLAT713_ALERT_1_C TORSION Unknown or Inconsistent Label ..........          B8 Check
                    B5          B8          B10         B6
PLAT713_ALERT_1_C TORSION Unknown or Inconsistent Label ..........         B10 Check
                    B5          B10         B3          B6
PLAT713_ALERT_1_C TORSION Unknown or Inconsistent Label ..........         B10 Check
                    B5          B10         B3          B8
PLAT713_ALERT_1_C TORSION Unknown or Inconsistent Label ..........          B8 Check
                    B6          B2          B3          B8
PLAT713_ALERT_1_C TORSION Unknown or Inconsistent Label ..........         B10 Check
                    B6          B2          B3          B10
PLAT713_ALERT_1_C TORSION Unknown or Inconsistent Label ..........          B8 Check
                    B6          B2          B4          B8
PLAT713_ALERT_1_C TORSION Unknown or Inconsistent Label ..........          B9 Check
                    B6          B2          B4          B9
PLAT713_ALERT_1_C TORSION Unknown or Inconsistent Label ..........          B9 Check
                    B6          B2          B9          B7
PLAT713_ALERT_1_C TORSION Unknown or Inconsistent Label ..........          B9 Check
                    B6          B3          B2          B9
PLAT713_ALERT_1_C TORSION Unknown or Inconsistent Label ..........          B8 Check
                    B6          B3          B4          B8
PLAT713_ALERT_1_C TORSION Unknown or Inconsistent Label ..........          B9 Check
                    B6          B3          B4          B9
PLAT713_ALERT_1_C TORSION Unknown or Inconsistent Label ..........          B8 Check
```

```
                   B6          B3          B8          B10
PLAT713_ALERT_1_C TORSION Unknown or Inconsistent Label ..........        B10 Check
                   B6          B3          B10         B8
PLAT713_ALERT_1_C TORSION Unknown or Inconsistent Label ..........        B10 Check
                   B6          B10         B3          B8
PLAT713_ALERT_1_C TORSION Unknown or Inconsistent Label ..........        B10 Check
                   B6          B10         B5          B7
PLAT713_ALERT_1_C TORSION Unknown or Inconsistent Label ..........        B10 Check
                   B6          B10         B5          B8
PLAT713_ALERT_1_C TORSION Unknown or Inconsistent Label ..........         B8 Check
                   B7          B1          B4          B8
PLAT713_ALERT_1_C TORSION Unknown or Inconsistent Label ..........         B9 Check
                   B7          B1          B4          B9
PLAT713_ALERT_1_C TORSION Unknown or Inconsistent Label ..........         B8 Check
                   B7          B1          B5          B8
PLAT713_ALERT_1_C TORSION Unknown or Inconsistent Label ..........        B10 Check
                   B7          B1          B5          B10
PLAT713_ALERT_1_C TORSION Unknown or Inconsistent Label ..........         B8 Check
                   B7          B1          B8          B10
PLAT713_ALERT_1_C TORSION Unknown or Inconsistent Label ..........         B8 Check
                   B7          B5          B1          B8
PLAT713_ALERT_1_C TORSION Unknown or Inconsistent Label ..........         B9 Check
                   B7          B5          B1          B9
PLAT713_ALERT_1_C TORSION Unknown or Inconsistent Label ..........         B8 Check
                   B7          B5          B8          B10
PLAT713_ALERT_1_C TORSION Unknown or Inconsistent Label ..........        B10 Check
                   B7          B5          B10         B8
PLAT713_ALERT_1_C TORSION Unknown or Inconsistent Label ..........         B9 Check
                   B7          B9          B1          B8
PLAT713_ALERT_1_C TORSION Unknown or Inconsistent Label ..........         B9 Check
                   B7          B9          B4          B8
PLAT713_ALERT_1_C TORSION Unknown or Inconsistent Label ..........         B8 Check
                   B8          B1          B4          B9
PLAT713_ALERT_1_C TORSION Unknown or Inconsistent Label ..........         B8 Check
                   B8          B1          B5          B10
PLAT713_ALERT_1_C TORSION Unknown or Inconsistent Label ..........         B8 Check
                   B8          B1          B7          B9
PLAT713_ALERT_1_C TORSION Unknown or Inconsistent Label ..........         B8 Check
                   B8          B3          B2          B9
PLAT713_ALERT_1_C TORSION Unknown or Inconsistent Label ..........         B8 Check
                   B8          B3          B4          B9
PLAT713_ALERT_1_C TORSION Unknown or Inconsistent Label ..........         B8 Check
                   B8          B3          B6          B10
PLAT713_ALERT_1_C TORSION Unknown or Inconsistent Label ..........         B8 Check
                   B8          B4          B1          B9
PLAT713_ALERT_1_C TORSION Unknown or Inconsistent Label ..........         B8 Check
                   B8          B4          B2          B9
PLAT713_ALERT_1_C TORSION Unknown or Inconsistent Label ..........         B8 Check
                   B8          B4          B3          B10
PLAT713_ALERT_1_C TORSION Unknown or Inconsistent Label ..........         B8 Check
                   B8          B5          B1          B9
PLAT713_ALERT_1_C TORSION Unknown or Inconsistent Label ..........         B8 Check
                   B8          B5          B7          B9
PLAT713_ALERT_1_C TORSION Unknown or Inconsistent Label ..........         B9 Check
                   B9          B1          B5          B10
PLAT713_ALERT_1_C TORSION Unknown or Inconsistent Label ..........         B9 Check
                   B9          B1          B8          B10
```

```
PLAT713_ALERT_1_C TORSION Unknown or Inconsistent Label ..........        B9 Check
                    B9          B2          B3          B10
PLAT713_ALERT_1_C TORSION Unknown or Inconsistent Label ..........        B9 Check
                    B9          B2          B6          B10
PLAT713_ALERT_1_C TORSION Unknown or Inconsistent Label ..........        B9 Check
                    B9          B4          B3          B10
PLAT713_ALERT_1_C TORSION Unknown or Inconsistent Label ..........        B9 Check
                    B9          B4          B8          B10
PLAT713_ALERT_1_C TORSION Unknown or Inconsistent Label ..........        B9 Check
                    B9          B7          B5          B10
PLAT713_ALERT_1_C TORSION Unknown or Inconsistent Label ..........        B8 Check
                    B1          B4          B8          H16
PLAT713_ALERT_1_C TORSION Unknown or Inconsistent Label ..........        B9 Check
                    B1          B4          B9          H7
PLAT713_ALERT_1_C TORSION Unknown or Inconsistent Label ..........        B9 Check
                    B1          B4          B9          H17
PLAT713_ALERT_1_C TORSION Unknown or Inconsistent Label ..........        B8 Check
                    B1          B5          B8          H16
PLAT713_ALERT_1_C TORSION Unknown or Inconsistent Label ..........       B10 Check
                    B1          B5          B10         H15
PLAT713_ALERT_1_C TORSION Unknown or Inconsistent Label ..........       B10 Check
                    B1          B5          B10         H18
PLAT713_ALERT_1_C TORSION Unknown or Inconsistent Label ..........       H15 Check
                    B1          B7          B5          H15
PLAT713_ALERT_1_C TORSION Unknown or Inconsistent Label ..........        B9 Check
                    B1          B7          B9          H7
PLAT713_ALERT_1_C TORSION Unknown or Inconsistent Label ..........        B9 Check
                    B1          B7          B9          H17
PLAT713_ALERT_1_C TORSION Unknown or Inconsistent Label ..........        B8 Check
                    B1          B8          B3          H1
PLAT713_ALERT_1_C TORSION Unknown or Inconsistent Label ..........        B8 Check
                    B1          B8          B4          H3
PLAT713_ALERT_1_C TORSION Unknown or Inconsistent Label ..........        B8 Check
                    B1          B8          B5          H2
PLAT713_ALERT_1_C TORSION Unknown or Inconsistent Label ..........        B8 Check
                    B1          B8          B5          H15
PLAT713_ALERT_1_C TORSION Unknown or Inconsistent Label ..........        B8 Check
                    B1          B8          B10         H15
PLAT713_ALERT_1_C TORSION Unknown or Inconsistent Label ..........        B8 Check
                    B1          B8          B10         H18
PLAT713_ALERT_1_C TORSION Unknown or Inconsistent Label ..........        B9 Check
                    B1          B9          B2          H7
PLAT713_ALERT_1_C TORSION Unknown or Inconsistent Label ..........        B9 Check
                    B1          B9          B2          H8
PLAT713_ALERT_1_C TORSION Unknown or Inconsistent Label ..........        B9 Check
                    B1          B9          B4          H3
PLAT713_ALERT_1_C TORSION Unknown or Inconsistent Label ..........        B9 Check
                    B1          B9          B7          H4
PLAT713_ALERT_1_C TORSION Unknown or Inconsistent Label ..........        B8 Check
                    B2          B3          B8          H16
PLAT713_ALERT_1_C TORSION Unknown or Inconsistent Label ..........       B10 Check
                    B2          B3          B10         H15
PLAT713_ALERT_1_C TORSION Unknown or Inconsistent Label ..........       B10 Check
                    B2          B3          B10         H18
PLAT713_ALERT_1_C TORSION Unknown or Inconsistent Label ..........        B8 Check
                    B2          B4          B8          H16
PLAT713_ALERT_1_C TORSION Unknown or Inconsistent Label ..........        B9 Check
```

```
                    B2          B4          B9          H7
PLAT713_ALERT_1_C TORSION Unknown or Inconsistent Label ..........          B9 Check
                    B2          B4          B9          H17
PLAT713_ALERT_1_C TORSION Unknown or Inconsistent Label ..........         B10 Check
                    B2          B6          B10         H15
PLAT713_ALERT_1_C TORSION Unknown or Inconsistent Label ..........         B10 Check
                    B2          B6          B10         H18
PLAT713_ALERT_1_C TORSION Unknown or Inconsistent Label ..........          B9 Check
                    B2          B9          B1          H5
PLAT713_ALERT_1_C TORSION Unknown or Inconsistent Label ..........          B9 Check
                    B2          B9          B4          H3
PLAT713_ALERT_1_C TORSION Unknown or Inconsistent Label ..........          B9 Check
                    B2          B9          B7          H4
PLAT713_ALERT_1_C TORSION Unknown or Inconsistent Label ..........          B9 Check
                    B3          B2          B9          H7
PLAT713_ALERT_1_C TORSION Unknown or Inconsistent Label ..........          B9 Check
                    B3          B2          B9          H17
PLAT713_ALERT_1_C TORSION Unknown or Inconsistent Label ..........          B8 Check
                    B3          B4          B8          H16
PLAT713_ALERT_1_C TORSION Unknown or Inconsistent Label ..........          B9 Check
                    B3          B4          B9          H7
PLAT713_ALERT_1_C TORSION Unknown or Inconsistent Label ..........          B9 Check
                    B3          B4          B9          H17
PLAT713_ALERT_1_C TORSION Unknown or Inconsistent Label ..........         B10 Check
                    B3          B6          B10         H15
PLAT713_ALERT_1_C TORSION Unknown or Inconsistent Label ..........         B10 Check
                    B3          B6          B10         H18
PLAT713_ALERT_1_C TORSION Unknown or Inconsistent Label ..........          B8 Check
                    B3          B8          B1          H5
PLAT713_ALERT_1_C TORSION Unknown or Inconsistent Label ..........          B8 Check
                    B3          B8          B4          H3
PLAT713_ALERT_1_C TORSION Unknown or Inconsistent Label ..........          B8 Check
                    B3          B8          B5          H2
PLAT713_ALERT_1_C TORSION Unknown or Inconsistent Label ..........          B8 Check
                    B3          B8          B5          H15
PLAT713_ALERT_1_C TORSION Unknown or Inconsistent Label ..........          B8 Check
                    B3          B8          B10         H15
PLAT713_ALERT_1_C TORSION Unknown or Inconsistent Label ..........          B8 Check
                    B3          B8          B10         H18
PLAT713_ALERT_1_C TORSION Unknown or Inconsistent Label ..........         B10 Check
                    B3          B10         B5          H2
PLAT713_ALERT_1_C TORSION Unknown or Inconsistent Label ..........         B10 Check
                    B3          B10         B5          H15
PLAT713_ALERT_1_C TORSION Unknown or Inconsistent Label ..........         B10 Check
                    B3          B10         B6          H9
PLAT713_ALERT_1_C TORSION Unknown or Inconsistent Label ..........         B10 Check
                    B3          B10         B8          H16
PLAT713_ALERT_1_C TORSION Unknown or Inconsistent Label ..........         H15 Check
                    B4          B1          B5          H15
PLAT713_ALERT_1_C TORSION Unknown or Inconsistent Label ..........          B8 Check
                    B4          B1          B8          H16
PLAT713_ALERT_1_C TORSION Unknown or Inconsistent Label ..........          B9 Check
                    B4          B1          B9          H7
PLAT713_ALERT_1_C TORSION Unknown or Inconsistent Label ..........          B9 Check
                    B4          B1          B9          H17
PLAT713_ALERT_1_C TORSION Unknown or Inconsistent Label ..........          B9 Check
                    B4          B2          B9          H7
```

```
PLAT713_ALERT_1_C TORSION Unknown or Inconsistent Label ..........          B9 Check
                    B4           B2           B9           H17
PLAT713_ALERT_1_C TORSION Unknown or Inconsistent Label ..........          B8 Check
                    B4           B3           B8           H16
PLAT713_ALERT_1_C TORSION Unknown or Inconsistent Label ..........         B10 Check
                    B4           B3           B10          H15
PLAT713_ALERT_1_C TORSION Unknown or Inconsistent Label ..........         B10 Check
                    B4           B3           B10          H18
PLAT713_ALERT_1_C TORSION Unknown or Inconsistent Label ..........          B8 Check
                    B4           B8           B1           H5
PLAT713_ALERT_1_C TORSION Unknown or Inconsistent Label ..........          B8 Check
                    B4           B8           B3           H1
PLAT713_ALERT_1_C TORSION Unknown or Inconsistent Label ..........          B8 Check
                    B4           B8           B5           H2
PLAT713_ALERT_1_C TORSION Unknown or Inconsistent Label ..........          B8 Check
                    B4           B8           B5           H15
PLAT713_ALERT_1_C TORSION Unknown or Inconsistent Label ..........          B8 Check
                    B4           B8           B10          H15
PLAT713_ALERT_1_C TORSION Unknown or Inconsistent Label ..........          B8 Check
                    B4           B8           B10          H18
PLAT713_ALERT_1_C TORSION Unknown or Inconsistent Label ..........          B9 Check
                    B4           B9           B1           H5
PLAT713_ALERT_1_C TORSION Unknown or Inconsistent Label ..........          B9 Check
                    B4           B9           B2           H7
PLAT713_ALERT_1_C TORSION Unknown or Inconsistent Label ..........          B9 Check
                    B4           B9           B2           H8
PLAT713_ALERT_1_C TORSION Unknown or Inconsistent Label ..........          B9 Check
                    B4           B9           B7           H4
PLAT713_ALERT_1_C TORSION Unknown or Inconsistent Label ..........          B8 Check
                    B5           B1           B8           H16
PLAT713_ALERT_1_C TORSION Unknown or Inconsistent Label ..........          B9 Check
                    B5           B1           B9           H7
PLAT713_ALERT_1_C TORSION Unknown or Inconsistent Label ..........          B9 Check
                    B5           B1           B9           H17
PLAT713_ALERT_1_C TORSION Unknown or Inconsistent Label ..........          B9 Check
                    B5           B7           B9           H7
PLAT713_ALERT_1_C TORSION Unknown or Inconsistent Label ..........          B9 Check
                    B5           B7           B9           H17
PLAT713_ALERT_1_C TORSION Unknown or Inconsistent Label ..........          B8 Check
                    B5           B8           B1           H5
PLAT713_ALERT_1_C TORSION Unknown or Inconsistent Label ..........          B8 Check
                    B5           B8           B3           H1
PLAT713_ALERT_1_C TORSION Unknown or Inconsistent Label ..........          B8 Check
                    B5           B8           B4           H3
PLAT713_ALERT_1_C TORSION Unknown or Inconsistent Label ..........          B8 Check
                    B5           B8           B10          H15
PLAT713_ALERT_1_C TORSION Unknown or Inconsistent Label ..........          B8 Check
                    B5           B8           B10          H18
PLAT713_ALERT_1_C TORSION Unknown or Inconsistent Label ..........         B10 Check
                    B5           B10          B3           H1
PLAT713_ALERT_1_C TORSION Unknown or Inconsistent Label ..........         B10 Check
                    B5           B10          B6           H9
PLAT713_ALERT_1_C TORSION Unknown or Inconsistent Label ..........         B10 Check
                    B5           B10          B8           H16
PLAT713_ALERT_1_C TORSION Unknown or Inconsistent Label ..........          B9 Check
                    B6           B2           B9           H7
PLAT713_ALERT_1_C TORSION Unknown or Inconsistent Label ..........          B9 Check
```

```
                   B6          B2          B9          H17
PLAT713_ALERT_1_C TORSION Unknown or Inconsistent Label ..........         B8 Check
                   B6          B3          B8          H16
PLAT713_ALERT_1_C TORSION Unknown or Inconsistent Label ..........        B10 Check
                   B6          B3          B10         H15
PLAT713_ALERT_1_C TORSION Unknown or Inconsistent Label ..........        B10 Check
                   B6          B3          B10         H18
PLAT713_ALERT_1_C TORSION Unknown or Inconsistent Label ..........        B10 Check
                   B6          B10         B3          H1
PLAT713_ALERT_1_C TORSION Unknown or Inconsistent Label ..........        B10 Check
                   B6          B10         B5          H2
PLAT713_ALERT_1_C TORSION Unknown or Inconsistent Label ..........        B10 Check
                   B6          B10         B5          H15
PLAT713_ALERT_1_C TORSION Unknown or Inconsistent Label ..........        B10 Check
                   B6          B10         B8          H16
PLAT713_ALERT_1_C TORSION Unknown or Inconsistent Label ..........        H15 Check
                   B7          B1          B5          H15
PLAT713_ALERT_1_C TORSION Unknown or Inconsistent Label ..........         B8 Check
                   B7          B1          B8          H16
PLAT713_ALERT_1_C TORSION Unknown or Inconsistent Label ..........         B9 Check
                   B7          B1          B9          H7
PLAT713_ALERT_1_C TORSION Unknown or Inconsistent Label ..........         B9 Check
                   B7          B1          B9          H17
PLAT713_ALERT_1_C TORSION Unknown or Inconsistent Label ..........         B8 Check
                   B7          B5          B8          H16
PLAT713_ALERT_1_C TORSION Unknown or Inconsistent Label ..........        B10 Check
                   B7          B5          B10         H15
PLAT713_ALERT_1_C TORSION Unknown or Inconsistent Label ..........        B10 Check
                   B7          B5          B10         H18
PLAT713_ALERT_1_C TORSION Unknown or Inconsistent Label ..........         B9 Check
                   B7          B9          B1          H5
PLAT713_ALERT_1_C TORSION Unknown or Inconsistent Label ..........         B9 Check
                   B7          B9          B2          H7
PLAT713_ALERT_1_C TORSION Unknown or Inconsistent Label ..........         B9 Check
                   B7          B9          B2          H8
PLAT713_ALERT_1_C TORSION Unknown or Inconsistent Label ..........         B9 Check
                   B7          B9          B4          H3
PLAT713_ALERT_1_C TORSION Unknown or Inconsistent Label ..........         B8 Check
                   B8          B1          B5          H15
PLAT713_ALERT_1_C TORSION Unknown or Inconsistent Label ..........         B8 Check
                   B8          B1          B9          H17
PLAT713_ALERT_1_C TORSION Unknown or Inconsistent Label ..........         B8 Check
                   B8          B3          B10         H15
PLAT713_ALERT_1_C TORSION Unknown or Inconsistent Label ..........         B8 Check
                   B8          B3          B10         H18
PLAT713_ALERT_1_C TORSION Unknown or Inconsistent Label ..........         B8 Check
                   B8          B4          B9          H17
PLAT713_ALERT_1_C TORSION Unknown or Inconsistent Label ..........         B8 Check
                   B8          B5          B10         H15
PLAT713_ALERT_1_C TORSION Unknown or Inconsistent Label ..........         B8 Check
                   B8          B5          B10         H18
PLAT713_ALERT_1_C TORSION Unknown or Inconsistent Label ..........         B8 Check
                   B8          B10         B5          H15
PLAT713_ALERT_1_C TORSION Unknown or Inconsistent Label ..........         B9 Check
                   B9          B1          B5          H15
PLAT713_ALERT_1_C TORSION Unknown or Inconsistent Label ..........         B9 Check
                   B9          B1          B8          H16
```

```
PLAT713_ALERT_1_C TORSION Unknown or Inconsistent Label ..........         B9 Check
                    B9          B4          B8          H16
PLAT713_ALERT_1_C TORSION Unknown or Inconsistent Label ..........         B9 Check
                    B9          B7          B5          H15
PLAT713_ALERT_1_C TORSION Unknown or Inconsistent Label ..........        B10 Check
                    B10         B3          B8          H16
PLAT713_ALERT_1_C TORSION Unknown or Inconsistent Label ..........        B10 Check
                    B10         B5          B8          H16
PLAT713_ALERT_1_C TORSION Unknown or Inconsistent Label ..........        B10 Check
                    B10         B8          B5          H15
PLAT713_ALERT_1_C TORSION Unknown or Inconsistent Label ..........        H15 Check
                    B1          B5          H15         B10
PLAT713_ALERT_1_C TORSION Unknown or Inconsistent Label ..........         B9 Check
                    B1          B9          H7          B2
PLAT713_ALERT_1_C TORSION Unknown or Inconsistent Label ..........         B9 Check
                    B3          B2          H7          B9
PLAT713_ALERT_1_C TORSION Unknown or Inconsistent Label ..........        B10 Check
                    B3          B10         H15         B5
PLAT713_ALERT_1_C TORSION Unknown or Inconsistent Label ..........         B9 Check
                    B4          B2          H7          B9
PLAT713_ALERT_1_C TORSION Unknown or Inconsistent Label ..........         B9 Check
                    B6          B2          H7          B9
PLAT713_ALERT_1_C TORSION Unknown or Inconsistent Label ..........        H15 Check
                    B7          B5          H15         B10
PLAT713_ALERT_1_C TORSION Unknown or Inconsistent Label ..........         B8 Check
                    B8          B5          H15         B10
PLAT713_ALERT_1_C TORSION Unknown or Inconsistent Label ..........         B9 Check
                    B2          H7          B9          B4
PLAT713_ALERT_1_C TORSION Unknown or Inconsistent Label ..........         B9 Check
                    B2          H7          B9          B7
PLAT713_ALERT_1_C TORSION Unknown or Inconsistent Label ..........        H15 Check
                    B5          H15         B10         B6
PLAT713_ALERT_1_C TORSION Unknown or Inconsistent Label ..........        H15 Check
                    B5          H15         B10         B8
PLAT713_ALERT_1_C TORSION Unknown or Inconsistent Label ..........         B9 Check
                    B2          H7          B9          H17
PLAT713_ALERT_1_C TORSION Unknown or Inconsistent Label ..........        H15 Check
                    B5          H15         B10         H18
PLAT713_ALERT_1_C TORSION Unknown or Inconsistent Label ..........        B10 Check
                    H6          N1          B6          B10
PLAT713_ALERT_1_C TORSION Unknown or Inconsistent Label ..........        B10 Check
                    H10         N1          B6          B10
PLAT713_ALERT_1_C TORSION Unknown or Inconsistent Label ..........         B9 Check
                    H11         N2          B7          B9
PLAT713_ALERT_1_C TORSION Unknown or Inconsistent Label ..........         B9 Check
                    H12         N2          B7          B9
PLAT713_ALERT_1_C TORSION Unknown or Inconsistent Label ..........        H13 Check
                    H13         N1          B6          B10
PLAT713_ALERT_1_C TORSION Unknown or Inconsistent Label ..........        H14 Check
                    H14         N2          B7          B9
PLAT713_ALERT_1_C TORSION Unknown or Inconsistent Label ..........        H14 Check
                    H4          B7          N2          H14
PLAT713_ALERT_1_C TORSION Unknown or Inconsistent Label ..........        H13 Check
                    H9          B6          N1          H13
PLAT713_ALERT_1_C TORSION Unknown or Inconsistent Label ..........         B9 Check
                    H1          B3          B2          B9
PLAT713_ALERT_1_C TORSION Unknown or Inconsistent Label ..........         B8 Check
```

```
                   H1          B3          B4          B8
PLAT713_ALERT_1_C TORSION Unknown or Inconsistent Label ..........         B9 Check
                   H1          B3          B4          B9
PLAT713_ALERT_1_C TORSION Unknown or Inconsistent Label ..........        B10 Check
                   H1          B3          B6          B10
PLAT713_ALERT_1_C TORSION Unknown or Inconsistent Label ..........         B8 Check
                   H1          B3          B8          B10
PLAT713_ALERT_1_C TORSION Unknown or Inconsistent Label ..........        B10 Check
                   H1          B3          B10         B8
PLAT713_ALERT_1_C TORSION Unknown or Inconsistent Label ..........         B8 Check
                   H2          B5          B1          B8
PLAT713_ALERT_1_C TORSION Unknown or Inconsistent Label ..........         B9 Check
                   H2          B5          B1          B9
PLAT713_ALERT_1_C TORSION Unknown or Inconsistent Label ..........         B9 Check
                   H2          B5          B7          B9
PLAT713_ALERT_1_C TORSION Unknown or Inconsistent Label ..........         B8 Check
                   H2          B5          B8          B10
PLAT713_ALERT_1_C TORSION Unknown or Inconsistent Label ..........        B10 Check
                   H2          B5          B10         B8
PLAT713_ALERT_1_C TORSION Unknown or Inconsistent Label ..........         B8 Check
                   H3          B4          B1          B8
PLAT713_ALERT_1_C TORSION Unknown or Inconsistent Label ..........         B9 Check
                   H3          B4          B1          B9
PLAT713_ALERT_1_C TORSION Unknown or Inconsistent Label ..........         B9 Check
                   H3          B4          B2          B9
PLAT713_ALERT_1_C TORSION Unknown or Inconsistent Label ..........         B8 Check
                   H3          B4          B3          B8
PLAT713_ALERT_1_C TORSION Unknown or Inconsistent Label ..........        B10 Check
                   H3          B4          B3          B10
PLAT713_ALERT_1_C TORSION Unknown or Inconsistent Label ..........         B8 Check
                   H3          B4          B8          B10
PLAT713_ALERT_1_C TORSION Unknown or Inconsistent Label ..........         B8 Check
                   H4          B7          B1          B8
PLAT713_ALERT_1_C TORSION Unknown or Inconsistent Label ..........         B9 Check
                   H4          B7          B1          B9
PLAT713_ALERT_1_C TORSION Unknown or Inconsistent Label ..........         B8 Check
                   H4          B7          B5          B8
PLAT713_ALERT_1_C TORSION Unknown or Inconsistent Label ..........        B10 Check
                   H4          B7          B5          B10
PLAT713_ALERT_1_C TORSION Unknown or Inconsistent Label ..........         B8 Check
                   H5          B1          B4          B8
PLAT713_ALERT_1_C TORSION Unknown or Inconsistent Label ..........         B9 Check
                   H5          B1          B4          B9
PLAT713_ALERT_1_C TORSION Unknown or Inconsistent Label ..........         B8 Check
                   H5          B1          B5          B8
PLAT713_ALERT_1_C TORSION Unknown or Inconsistent Label ..........        B10 Check
                   H5          B1          B5          B10
PLAT713_ALERT_1_C TORSION Unknown or Inconsistent Label ..........         B9 Check
                   H5          B1          B7          B9
PLAT713_ALERT_1_C TORSION Unknown or Inconsistent Label ..........         B8 Check
                   H5          B1          B8          B10
PLAT713_ALERT_1_C TORSION Unknown or Inconsistent Label ..........         B8 Check
                   H7          B2          B3          B8
PLAT713_ALERT_1_C TORSION Unknown or Inconsistent Label ..........        B10 Check
                   H7          B2          B3          B10
PLAT713_ALERT_1_C TORSION Unknown or Inconsistent Label ..........         B8 Check
                   H7          B2          B4          B8
```

```
PLAT713_ALERT_1_C TORSION Unknown or Inconsistent Label ..........         B9 Check
                   H7          B2          B4          B9
PLAT713_ALERT_1_C TORSION Unknown or Inconsistent Label ..........        B10 Check
                   H7          B2          B6          B10
PLAT713_ALERT_1_C TORSION Unknown or Inconsistent Label ..........         B9 Check
                   H7          B9          B1          B8
PLAT713_ALERT_1_C TORSION Unknown or Inconsistent Label ..........         B9 Check
                   H7          B9          B4          B8
PLAT713_ALERT_1_C TORSION Unknown or Inconsistent Label ..........         B8 Check
                   H8          B2          B3          B8
PLAT713_ALERT_1_C TORSION Unknown or Inconsistent Label ..........        B10 Check
                   H8          B2          B3          B10
PLAT713_ALERT_1_C TORSION Unknown or Inconsistent Label ..........         B8 Check
                   H8          B2          B4          B8
PLAT713_ALERT_1_C TORSION Unknown or Inconsistent Label ..........         B9 Check
                   H8          B2          B4          B9
PLAT713_ALERT_1_C TORSION Unknown or Inconsistent Label ..........        B10 Check
                   H8          B2          B6          B10
PLAT713_ALERT_1_C TORSION Unknown or Inconsistent Label ..........         B9 Check
                   H9          B6          B2          B9
PLAT713_ALERT_1_C TORSION Unknown or Inconsistent Label ..........         B8 Check
                   H9          B6          B3          B8
PLAT713_ALERT_1_C TORSION Unknown or Inconsistent Label ..........        B10 Check
                   H9          B6          B3          B10
PLAT713_ALERT_1_C TORSION Unknown or Inconsistent Label ..........        B10 Check
                   H9          B6          B10         B8
PLAT713_ALERT_1_C TORSION Unknown or Inconsistent Label ..........         B8 Check
                   H1          B3          B8          H16
PLAT713_ALERT_1_C TORSION Unknown or Inconsistent Label ..........        B10 Check
                   H1          B3          B10         H15
PLAT713_ALERT_1_C TORSION Unknown or Inconsistent Label ..........        B10 Check
                   H1          B3          B10         H18
PLAT713_ALERT_1_C TORSION Unknown or Inconsistent Label ..........         B8 Check
                   H2          B5          B8          H16
PLAT713_ALERT_1_C TORSION Unknown or Inconsistent Label ..........        B10 Check
                   H2          B5          B10         H15
PLAT713_ALERT_1_C TORSION Unknown or Inconsistent Label ..........        B10 Check
                   H2          B5          B10         H18
PLAT713_ALERT_1_C TORSION Unknown or Inconsistent Label ..........         B8 Check
                   H3          B4          B8          H16
PLAT713_ALERT_1_C TORSION Unknown or Inconsistent Label ..........         B9 Check
                   H3          B4          B9          H7
PLAT713_ALERT_1_C TORSION Unknown or Inconsistent Label ..........         B9 Check
                   H3          B4          B9          H17
PLAT713_ALERT_1_C TORSION Unknown or Inconsistent Label ..........        H15 Check
                   H4          B7          B5          H15
PLAT713_ALERT_1_C TORSION Unknown or Inconsistent Label ..........         B9 Check
                   H4          B7          B9          H7
PLAT713_ALERT_1_C TORSION Unknown or Inconsistent Label ..........         B9 Check
                   H4          B7          B9          H17
PLAT713_ALERT_1_C TORSION Unknown or Inconsistent Label ..........        H15 Check
                   H5          B1          B5          H15
PLAT713_ALERT_1_C TORSION Unknown or Inconsistent Label ..........         B8 Check
                   H5          B1          B8          H16
PLAT713_ALERT_1_C TORSION Unknown or Inconsistent Label ..........         B9 Check
                   H5          B1          B9          H7
PLAT713_ALERT_1_C TORSION Unknown or Inconsistent Label ..........         B9 Check
```

```
                    H5          B1          B9          H17
PLAT713_ALERT_1_C TORSION Unknown or Inconsistent Label .........         B9 Check
                    H7          B2          B9          H17
PLAT713_ALERT_1_C TORSION Unknown or Inconsistent Label .........         B9 Check
                    H7          B9          B2          H8
PLAT713_ALERT_1_C TORSION Unknown or Inconsistent Label .........         B9 Check
                    H8          B2          B9          H17
PLAT713_ALERT_1_C TORSION Unknown or Inconsistent Label .........        B10 Check
                    H9          B6          B10         H15
PLAT713_ALERT_1_C TORSION Unknown or Inconsistent Label .........        B10 Check
                    H9          B6          B10         H18
PLAT713_ALERT_1_C TORSION Unknown or Inconsistent Label .........        H15 Check
                    H15         B5          B8          H16
PLAT713_ALERT_1_C TORSION Unknown or Inconsistent Label .........        H15 Check
                    H15         B5          B10         H18
PLAT713_ALERT_1_C TORSION Unknown or Inconsistent Label .........        H15 Check
                    H15         B10         B8          H16
PLAT713_ALERT_1_C TORSION Unknown or Inconsistent Label .........        H16 Check
                    H16         B8          B10         H18
PLAT713_ALERT_1_C TORSION Unknown or Inconsistent Label .........        H15 Check
                    H2          B5          H15         B10
PLAT713_ALERT_1_C TORSION Unknown or Inconsistent Label .........         B9 Check
                    H8          B2          H7          B9
PLAT910_ALERT_3_C Missing FCF Reflection(s) Below Theta(Min)[Deg]=       3.01 Note
                2  0  0,   2  1  0,   0  1  1,   1  0  1,   2  0  1,
PLAT913_ALERT_3_C Missing # of Very Strong Reflections in FCF ....          7 Note
                0  2  0,   0  4  0,   2  1  0,   4  1  0,  14  0  0,   0  1  1,
                0  0  2,
```

Alert level G

```
FORMU01_ALERT_2_G  There is a discrepancy between the atom counts in the
            _chemical_formula_sum and the formula from the _atom_site* data.
            Atom count from _chemical_formula_sum:H18 B10 N2
            Atom count from the _atom_site data:  H30 B16 N2
ABSMU01_ALERT_1_G  Calculation of _exptl_absorpt_correction_mu
                not performed for this radiation type.
CELLZ01_ALERT_1_G Difference between formula and atom_site contents detected.
CELLZ01_ALERT_1_G ALERT: Large difference may be due to a
            symmetry error - see SYMMG tests
           From the CIF: _cell_formula_units_Z    4
           From the CIF: _chemical_formula_sum  B10 H18 N2
           TEST: Compare cell contents of formula and atom_site data

           atom    Z*formula  cif sites diff
           B         40.00     64.00  -24.00
           H         72.00    120.00  -48.00
           N          8.00      8.00    0.00
PLAT005_ALERT_5_G No Embedded Refinement Details Found  in the CIF     Please Do !
PLAT020_ALERT_3_G The Value of Rint is Greater Than 0.12 ........      0.152 Report
PLAT180_ALERT_4_G Check Cell Rounding: # of Values Ending with 0 =          3 Note
PLAT410_ALERT_2_G Short Intra H...H Contact  H7       ..H9       .       2.14 Ang.
                                                    x,y,z  =      1_555 Check
PLAT808_ALERT_5_G No Parseable SHELXL Style Weighting Scheme Found     Please Check
PLAT883_ALERT_1_G Absent Datum for _atom_sites_solution_primary ..     Please Do !
PLAT911_ALERT_3_G Missing FCF Refl Between Thmin & STh/L=    0.600        399 Report
                0  2  0,   0  4  0,   0  6  0,   0  8  0,   2  2  0,   2  3  0,
```

```
                 2  4  0,   2  5  0,   2  6  0,   2  7  0,   2  8  0,   4  0  0,
                 4  1  0,   4  2  0,   4  3  0,   4  4  0,   4  5  0,   4  6  0,
                 4  7  0,   4  8  0,   6  0  0,   6  1  0,   6  2  0,   6  3  0,
                 6  4  0,   6  5  0,   6  6  0,   6  7  0,   6  8  0,   8  0  0,
                    (  369 More Missing: see the .ckf listing file)
PLAT912_ALERT_4_G Missing # of FCF Reflections Above STh/L=  0.600       1902 Note
PLAT929_ALERT_5_G No Weight Pars,Obs and Calc R1,wR2,S not Checked          ! Info
PLAT961_ALERT_5_G Dataset Contains no Negative Intensities ......     Please Check
PLAT969_ALERT_5_G The 'Henn et al.' R-Factor-gap value ..........      0.373 Note
             Predicted wR2: Based on SigI**2 14.07 or SHELX Weight 14.07
PLAT980_ALERT_1_G No Anomalous Scattering Factors Found in CIF ...     Please Check
```

```
   1 ALERT level A = Most likely a serious problem - resolve or explain
   0 ALERT level B = A potentially serious problem, consider carefully
 547 ALERT level C = Check. Ensure it is not caused by an omission or oversight
  16 ALERT level G = General information/check it is not something unexpected

 541 ALERT type 1 CIF construction/syntax error, inconsistent or missing data
   9 ALERT type 2 Indicator that the structure model may be wrong or deficient
   7 ALERT type 3 Indicator that the structure quality may be low
   2 ALERT type 4 Improvement, methodology, query or suggestion
   5 ALERT type 5 Informative message, check
```

It is advisable to attempt to resolve as many as possible of the alerts in all categories. Often the minor alerts point to easily fixed oversights, errors and omissions in your CIF or refinement strategy, so attention to these fine details can be worthwhile. In order to resolve some of the more serious problems it may be necessary to carry out additional measurements or structure refinements. However, the purpose of your study may justify the reported deviations and the more serious of these should normally be commented upon in the discussion or experimental section of a paper or in the "special_details" fields of the CIF. checkCIF was carefully designed to identify outliers and unusual parameters, but every test has its limitations and alerts that are not important in a particular case may appear. Conversely, the absence of alerts does not guarantee there are no aspects of the results needing attention. It is up to the individual to critically assess their own results and, if necessary, seek expert advice.

Publication of your CIF in IUCr journals

A basic structural check has been run on your CIF. These basic checks will be run on all CIFs submitted for publication in IUCr journals (*Acta Crystallographica*, *Journal of Applied Crystallography*, *Journal of Synchrotron Radiation*); however, if you intend to submit to *Acta Crystallographica Section C* or *E* or *IUCrData*, you should make sure that full publication checks are run on the final version of your CIF prior to submission.

Publication of your CIF in other journals

Please refer to the *Notes for Authors* of the relevant journal for any special instructions relating to CIF submission.

PLATON version of 04/06/2025; check.def file version of 30/05/2025

Datablock Bisammonia_arachno-decaborane12_HAR - ellipsoid plot

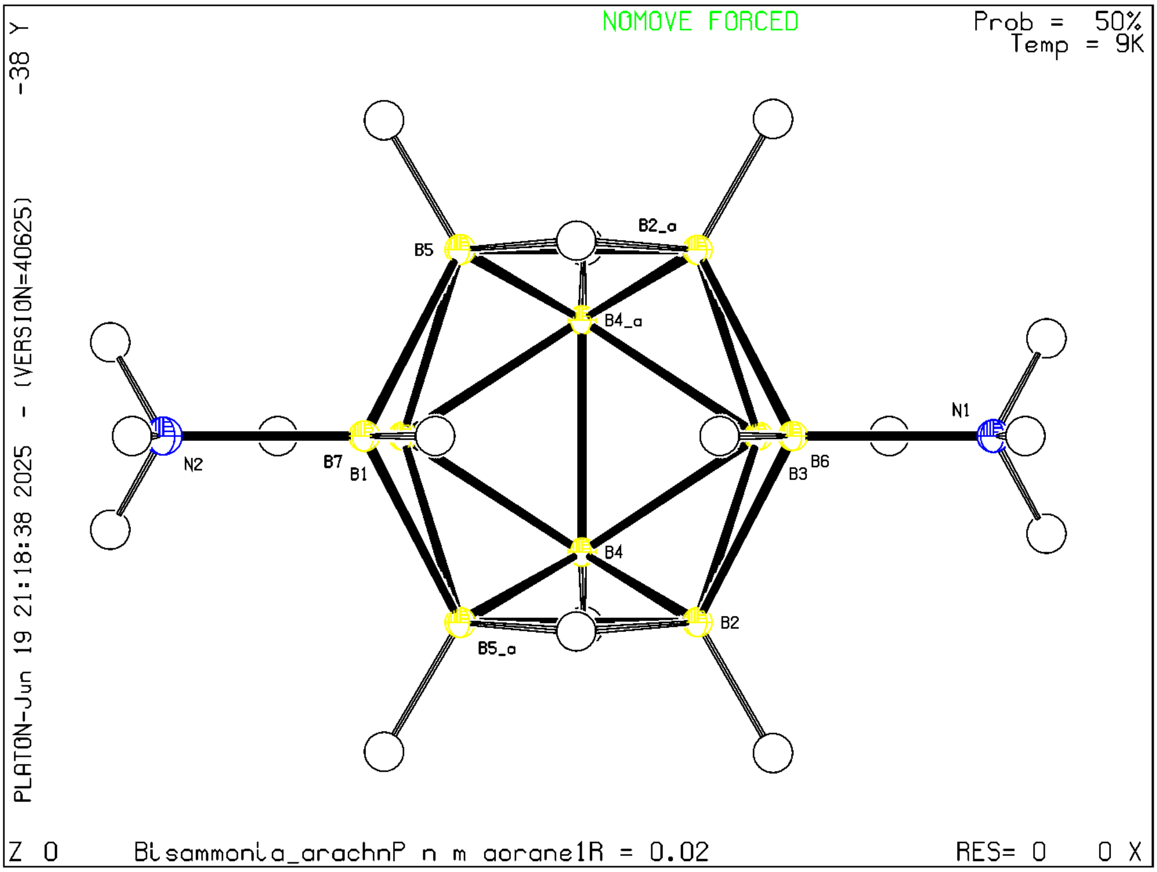

Supplement: Supplementary file 2 [file Bis(ammonia)_arachno-decaborane(12)_HAR.pdf › G_Bisammonia_arachno-decaborane(12)/Bis(ammonia)_arachno-decaborane(12)_HAR.pdf]
