# Supplementary material for: Periodic Hirshfeld Atom Refinement
Source: J Phys Chem Lett. 2026 Feb 27;17(11):3170–9. doi: 10.1021/acs.jpclett.5c03918 (PMC13007019; doi:10.1021/acs.jpclett.5c03918)

checkCIF/PLATON report

Structure factors have been supplied for datablock(s) Bislutidinium_closo-dodecaborate12__IAM

THIS REPORT IS FOR GUIDANCE ONLY. IF USED AS PART OF A REVIEW PROCEDURE FOR PUBLICATION, IT SHOULD NOT REPLACE THE EXPERTISE OF AN EXPERIENCED CRYSTALLOGRAPHIC REFEREE.

No syntax errors found. CIF dictionary Interpreting this report

Datablock: Bislutidinium_closo-dodecaborate12__IAM

```
Bond precision:   C-C = 0.0008 A           Wavelength=0.56000

Cell:               a=9.920(2)      b=9.1900(19)        c=11.841(2)
                    alpha=90        beta=95.88(3)       gamma=90
Temperature:        100 K
```

	Calculated	Reported
Volume	1073.8(4)	1073.8(4)
Space group	P 21/n	P 1 21/n 1
Hall group	-P 2yn	-P 2yn
Moiety formula	B12 H12, 2(C7 H10 N)	2(C7 H10 N), B12 H12
Sum formula	C14 H32 B12 N2	B12 C14 H32 N2
Mr	358.14	358.14
Dx,g cm-3	1.108	1.108
Z	2	2
Mu (mm-1)	0.038	0.038
F000	380.0	380.0
F000’	379.98	
h,k,lmax	23,21,27	22,21,27
Nref	14030	7599
Tmin,Tmax	0.989,0.992	
Tmin’	0.989	

```
Correction method= Not given

Data completeness= 0.542           Theta(max)= 40.510

                                                       wR2(reflections)=
R(reflections)= 0.0442( 7599)                          wR= 0.0611( 7599)

S = 0.859                  Npar= 191
```

The following ALERTS were generated. Each ALERT has the format
test-name_ALERT_alert-type_alert-level.
Click on the hyperlinks for more details of the test.

Alert level B

```
PLAT097_ALERT_2_B Large Reported Max.  (Positive) Residual Density       0.81 eA-3
```

Author Response: C-C bonds are poorly modeled in the IAM.

```
PLAT703_ALERT_1_B Torsion Calc    -0.23(9), Rep     0.00(8), Dev..       2.56 Sigma
              C1  -N   -C2  -C3    1_555  1_555  1_555  1_555      #   11 Check
```

Author Response: Very small torsion angles are regarded as 0 degree. For example, the torsion angle for C1-N-C2-C3 is equal to arccos(0.9999918). Considering the significant digit in the cif file, the value 0.9999918 was rounded to be 1.0000, resulting in arccos(1.0) = 0 deg.

```
PLAT703_ALERT_1_B Torsion Calc     0.14(6), Rep     0.00(5), Dev..       2.33 Sigma
              B3  -B4  -B1  -B6    1_555  1_555  1_555  1_555      #  183 Check
```

Author Response: Very small torsion angles are regarded as 0 degree. For example, the torsion angle for C1-N-C2-C3 is equal to arccos(0.9999918). Considering the significant digit in the cif file, the value 0.9999918 was rounded to be 1.0000, resulting in arccos(1.0) = 0 deg.

```
PLAT703_ALERT_1_B Torsion Calc    -0.13(6), Rep     0.00(5), Dev..       2.17 Sigma
              B4  -B1  -B6  -B5    1_555  1_555  1_555  1_555      #  231 Check
```

Author Response: Very small torsion angles are regarded as 0 degree. For example, the torsion angle for C1-N-C2-C3 is equal to arccos(0.9999918). Considering the significant digit in the cif file, the value 0.9999918 was rounded to be 1.0000, resulting in arccos(1.0) = 0 deg.

Alert level C

```
DIFMX02_ALERT_1_C  The maximum difference density is > 0.1*ZMAX*0.75
            The relevant atom site should be identified.
REFLE01_ALERT_3_C  The _reflns_threshold_multiplier given is >= 4
            Premultiplier =    4.01
REFLE01_ALERT_3_C  The _reflns_threshold_multiplier given is >= 4
            Premultiplier =    4.01
PLAT041_ALERT_1_C Calc. and Reported SumFormula    Strings  Differ     Please Check
              Calc: C14 H32 B12 N2
              Rep.: B12 C14 H32 N2
PLAT042_ALERT_1_C Calc. and Reported MoietyFormula Strings  Differ     Please Check
              Calc: B12 H12, 2(C7 H10 N)
```

```
            Rep.: 2(C7 H10 N), B12 H12
PLAT094_ALERT_2_C Ratio of Maximum / Minimum Residual Density ....       3.06 Report
PLAT703_ALERT_1_C Torsion Calc    -0.10(7), Rep     0.00(5), Dev..       1.43 Sigma
              B1  -B4  -B3  -B5    1_555  1_555  1_555  1_555      #   53 Check
```

Author Response: Very small torsion angles are regarded as 0 degree. For example, the torsion angle for C1-N-C2-C3 is equal to arccos(0.9999918). Considering the significant digit in the cif file, the value 0.9999918 was rounded to be 1.0000, resulting in arccos(1.0) = 0 deg.

```
PLAT703_ALERT_1_C Torsion Calc     0.07(6), Rep     0.00(5), Dev..       1.17 Sigma
              B1  -B6  -B5  -B3    1_555  1_555  1_555  1_555      #   67 Check
```

Author Response: Very small torsion angles are regarded as 0 degree. For example, the torsion angle for C1-N-C2-C3 is equal to arccos(0.9999918). Considering the significant digit in the cif file, the value 0.9999918 was rounded to be 1.0000, resulting in arccos(1.0) = 0 deg.

```
PLAT711_ALERT_1_C BOND    Unknown or Inconsistent Label .........          B7 Check
                    B1          B7
PLAT711_ALERT_1_C BOND    Unknown or Inconsistent Label .........          B8 Check
                    B1          B8
PLAT711_ALERT_1_C BOND    Unknown or Inconsistent Label .........          B9 Check
                    B3          B9
PLAT711_ALERT_1_C BOND    Unknown or Inconsistent Label .........         B10 Check
                    B3          B10
PLAT711_ALERT_1_C BOND    Unknown or Inconsistent Label .........          B8 Check
                    B4          B8
PLAT711_ALERT_1_C BOND    Unknown or Inconsistent Label .........         B10 Check
                    B4          B10
PLAT711_ALERT_1_C BOND    Unknown or Inconsistent Label .........          B9 Check
                    B5          B9
PLAT711_ALERT_1_C BOND    Unknown or Inconsistent Label .........         B11 Check
                    B5          B11
PLAT711_ALERT_1_C BOND    Unknown or Inconsistent Label .........          B7 Check
                    B6          B7
PLAT711_ALERT_1_C BOND    Unknown or Inconsistent Label .........         B11 Check
                    B6          B11
PLAT711_ALERT_1_C BOND    Unknown or Inconsistent Label .........          B7 Check
                    B7          B8
PLAT711_ALERT_1_C BOND    Unknown or Inconsistent Label .........          B7 Check
                    B7          B11
PLAT711_ALERT_1_C BOND    Unknown or Inconsistent Label .........          B7 Check
                    B7          B12
PLAT711_ALERT_1_C BOND    Unknown or Inconsistent Label .........          B8 Check
                    B8          B10
PLAT711_ALERT_1_C BOND    Unknown or Inconsistent Label .........          B8 Check
                    B8          B12
PLAT711_ALERT_1_C BOND    Unknown or Inconsistent Label .........          B9 Check
                    B9          B10
PLAT711_ALERT_1_C BOND    Unknown or Inconsistent Label .........          B9 Check
                    B9          B11
PLAT711_ALERT_1_C BOND    Unknown or Inconsistent Label .........          B9 Check
```

```
                    B9          B12
PLAT711_ALERT_1_C BOND    Unknown or Inconsistent Label .........        B10 Check
                    B10         B12
PLAT711_ALERT_1_C BOND    Unknown or Inconsistent Label .........        B11 Check
                    B11         B12
PLAT711_ALERT_1_C BOND    Unknown or Inconsistent Label .........         B7 Check
                    B7          H17
PLAT711_ALERT_1_C BOND    Unknown or Inconsistent Label .........         B8 Check
                    B8          H18
PLAT711_ALERT_1_C BOND    Unknown or Inconsistent Label .........         B9 Check
                    B9          H19
PLAT711_ALERT_1_C BOND    Unknown or Inconsistent Label .........        B10 Check
                    B10         H20
PLAT711_ALERT_1_C BOND    Unknown or Inconsistent Label .........        B11 Check
                    B11         H21
PLAT711_ALERT_1_C BOND    Unknown or Inconsistent Label .........        B12 Check
                    B12         H22
PLAT712_ALERT_1_C ANGLE   Unknown or Inconsistent Label .........         B8 Check
                    B1          B4          B8
PLAT712_ALERT_1_C ANGLE   Unknown or Inconsistent Label .........        B10 Check
                    B1          B4          B10
PLAT712_ALERT_1_C ANGLE   Unknown or Inconsistent Label .........         B7 Check
                    B1          B6          B7
PLAT712_ALERT_1_C ANGLE   Unknown or Inconsistent Label .........        B11 Check
                    B1          B6          B11
PLAT712_ALERT_1_C ANGLE   Unknown or Inconsistent Label .........         B7 Check
                    B1          B7          B6
PLAT712_ALERT_1_C ANGLE   Unknown or Inconsistent Label .........         B7 Check
                    B1          B7          B8
PLAT712_ALERT_1_C ANGLE   Unknown or Inconsistent Label .........         B7 Check
                    B1          B7          B11
PLAT712_ALERT_1_C ANGLE   Unknown or Inconsistent Label .........         B7 Check
                    B1          B7          B12
PLAT712_ALERT_1_C ANGLE   Unknown or Inconsistent Label .........         B8 Check
                    B1          B8          B4
PLAT712_ALERT_1_C ANGLE   Unknown or Inconsistent Label .........         B8 Check
                    B1          B8          B7
PLAT712_ALERT_1_C ANGLE   Unknown or Inconsistent Label .........         B8 Check
                    B1          B8          B10
PLAT712_ALERT_1_C ANGLE   Unknown or Inconsistent Label .........         B8 Check
                    B1          B8          B12
PLAT712_ALERT_1_C ANGLE   Unknown or Inconsistent Label .........         B7 Check
                    B2          B1          B7
PLAT712_ALERT_1_C ANGLE   Unknown or Inconsistent Label .........         B8 Check
                    B2          B1          B8
PLAT712_ALERT_1_C ANGLE   Unknown or Inconsistent Label .........         B9 Check
                    B2          B3          B9
PLAT712_ALERT_1_C ANGLE   Unknown or Inconsistent Label .........        B10 Check
                    B2          B3          B10
PLAT712_ALERT_1_C ANGLE   Unknown or Inconsistent Label .........         B8 Check
                    B2          B4          B8
PLAT712_ALERT_1_C ANGLE   Unknown or Inconsistent Label .........        B10 Check
                    B2          B4          B10
PLAT712_ALERT_1_C ANGLE   Unknown or Inconsistent Label .........         B9 Check
                    B2          B5          B9
PLAT712_ALERT_1_C ANGLE   Unknown or Inconsistent Label .........        B11 Check
                    B2          B5          B11
```

```
PLAT712_ALERT_1_C ANGLE   Unknown or Inconsistent Label .........          B7 Check
                    B2          B6          B7
PLAT712_ALERT_1_C ANGLE   Unknown or Inconsistent Label .........         B11 Check
                    B2          B6          B11
PLAT712_ALERT_1_C ANGLE   Unknown or Inconsistent Label .........          B8 Check
                    B3          B4          B8
PLAT712_ALERT_1_C ANGLE   Unknown or Inconsistent Label .........         B10 Check
                    B3          B4          B10
PLAT712_ALERT_1_C ANGLE   Unknown or Inconsistent Label .........          B9 Check
                    B3          B5          B9
PLAT712_ALERT_1_C ANGLE   Unknown or Inconsistent Label .........         B11 Check
                    B3          B5          B11
PLAT712_ALERT_1_C ANGLE   Unknown or Inconsistent Label .........          B9 Check
                    B3          B9          B5
PLAT712_ALERT_1_C ANGLE   Unknown or Inconsistent Label .........          B9 Check
                    B3          B9          B10
PLAT712_ALERT_1_C ANGLE   Unknown or Inconsistent Label .........          B9 Check
                    B3          B9          B11
PLAT712_ALERT_1_C ANGLE   Unknown or Inconsistent Label .........          B9 Check
                    B3          B9          B12
PLAT712_ALERT_1_C ANGLE   Unknown or Inconsistent Label .........         B10 Check
                    B3          B10         B4
PLAT712_ALERT_1_C ANGLE   Unknown or Inconsistent Label .........         B10 Check
                    B3          B10         B8
PLAT712_ALERT_1_C ANGLE   Unknown or Inconsistent Label .........         B10 Check
                    B3          B10         B9
PLAT712_ALERT_1_C ANGLE   Unknown or Inconsistent Label .........         B10 Check
                    B3          B10         B12
PLAT712_ALERT_1_C ANGLE   Unknown or Inconsistent Label .........          B7 Check
                    B4          B1          B7
PLAT712_ALERT_1_C ANGLE   Unknown or Inconsistent Label .........          B8 Check
                    B4          B1          B8
PLAT712_ALERT_1_C ANGLE   Unknown or Inconsistent Label .........          B9 Check
                    B4          B3          B9
PLAT712_ALERT_1_C ANGLE   Unknown or Inconsistent Label .........         B10 Check
                    B4          B3          B10
PLAT712_ALERT_1_C ANGLE   Unknown or Inconsistent Label .........          B8 Check
                    B4          B8          B7
PLAT712_ALERT_1_C ANGLE   Unknown or Inconsistent Label .........          B8 Check
                    B4          B8          B10
PLAT712_ALERT_1_C ANGLE   Unknown or Inconsistent Label .........          B8 Check
                    B4          B8          B12
PLAT712_ALERT_1_C ANGLE   Unknown or Inconsistent Label .........         B10 Check
                    B4          B10         B8
PLAT712_ALERT_1_C ANGLE   Unknown or Inconsistent Label .........         B10 Check
                    B4          B10         B9
PLAT712_ALERT_1_C ANGLE   Unknown or Inconsistent Label .........         B10 Check
                    B4          B10         B12
PLAT712_ALERT_1_C ANGLE   Unknown or Inconsistent Label .........          B9 Check
                    B5          B3          B9
PLAT712_ALERT_1_C ANGLE   Unknown or Inconsistent Label .........         B10 Check
                    B5          B3          B10
PLAT712_ALERT_1_C ANGLE   Unknown or Inconsistent Label .........          B7 Check
                    B5          B6          B7
PLAT712_ALERT_1_C ANGLE   Unknown or Inconsistent Label .........         B11 Check
                    B5          B6          B11
PLAT712_ALERT_1_C ANGLE   Unknown or Inconsistent Label .........          B9 Check
```

```
                    B5          B9          B10
PLAT712_ALERT_1_C ANGLE   Unknown or Inconsistent Label ..........         B9 Check
                    B5          B9          B11
PLAT712_ALERT_1_C ANGLE   Unknown or Inconsistent Label ..........         B9 Check
                    B5          B9          B12
PLAT712_ALERT_1_C ANGLE   Unknown or Inconsistent Label ..........        B11 Check
                    B5          B11         B6
PLAT712_ALERT_1_C ANGLE   Unknown or Inconsistent Label ..........        B11 Check
                    B5          B11         B7
PLAT712_ALERT_1_C ANGLE   Unknown or Inconsistent Label ..........        B11 Check
                    B5          B11         B9
PLAT712_ALERT_1_C ANGLE   Unknown or Inconsistent Label ..........        B11 Check
                    B5          B11         B12
PLAT712_ALERT_1_C ANGLE   Unknown or Inconsistent Label ..........         B7 Check
                    B6          B1          B7
PLAT712_ALERT_1_C ANGLE   Unknown or Inconsistent Label ..........         B8 Check
                    B6          B1          B8
PLAT712_ALERT_1_C ANGLE   Unknown or Inconsistent Label ..........         B9 Check
                    B6          B5          B9
PLAT712_ALERT_1_C ANGLE   Unknown or Inconsistent Label ..........        B11 Check
                    B6          B5          B11
PLAT712_ALERT_1_C ANGLE   Unknown or Inconsistent Label ..........         B7 Check
                    B6          B7          B8
PLAT712_ALERT_1_C ANGLE   Unknown or Inconsistent Label ..........         B7 Check
                    B6          B7          B11
PLAT712_ALERT_1_C ANGLE   Unknown or Inconsistent Label ..........         B7 Check
                    B6          B7          B12
PLAT712_ALERT_1_C ANGLE   Unknown or Inconsistent Label ..........        B11 Check
                    B6          B11         B7
PLAT712_ALERT_1_C ANGLE   Unknown or Inconsistent Label ..........        B11 Check
                    B6          B11         B9
PLAT712_ALERT_1_C ANGLE   Unknown or Inconsistent Label ..........        B11 Check
                    B6          B11         B12
PLAT712_ALERT_1_C ANGLE   Unknown or Inconsistent Label ..........         B7 Check
                    B7          B1          B8
PLAT712_ALERT_1_C ANGLE   Unknown or Inconsistent Label ..........         B7 Check
                    B7          B6          B11
PLAT712_ALERT_1_C ANGLE   Unknown or Inconsistent Label ..........         B7 Check
                    B7          B8          B10
PLAT712_ALERT_1_C ANGLE   Unknown or Inconsistent Label ..........         B7 Check
                    B7          B8          B12
PLAT712_ALERT_1_C ANGLE   Unknown or Inconsistent Label ..........         B7 Check
                    B7          B11         B9
PLAT712_ALERT_1_C ANGLE   Unknown or Inconsistent Label ..........         B7 Check
                    B7          B11         B12
PLAT712_ALERT_1_C ANGLE   Unknown or Inconsistent Label ..........         B7 Check
                    B7          B12         B8
PLAT712_ALERT_1_C ANGLE   Unknown or Inconsistent Label ..........         B7 Check
                    B7          B12         B9
PLAT712_ALERT_1_C ANGLE   Unknown or Inconsistent Label ..........         B7 Check
                    B7          B12         B10
PLAT712_ALERT_1_C ANGLE   Unknown or Inconsistent Label ..........         B7 Check
                    B7          B12         B11
PLAT712_ALERT_1_C ANGLE   Unknown or Inconsistent Label ..........         B8 Check
                    B8          B4          B10
PLAT712_ALERT_1_C ANGLE   Unknown or Inconsistent Label ..........         B8 Check
                    B8          B7          B11
```

```
PLAT712_ALERT_1_C ANGLE   Unknown or Inconsistent Label .........          B8 Check
                   B8          B7          B12
PLAT712_ALERT_1_C ANGLE   Unknown or Inconsistent Label .........          B8 Check
                   B8          B10         B9
PLAT712_ALERT_1_C ANGLE   Unknown or Inconsistent Label .........          B8 Check
                   B8          B10         B12
PLAT712_ALERT_1_C ANGLE   Unknown or Inconsistent Label .........          B8 Check
                   B8          B12         B9
PLAT712_ALERT_1_C ANGLE   Unknown or Inconsistent Label .........          B8 Check
                   B8          B12         B10
PLAT712_ALERT_1_C ANGLE   Unknown or Inconsistent Label .........          B8 Check
                   B8          B12         B11
PLAT712_ALERT_1_C ANGLE   Unknown or Inconsistent Label .........          B9 Check
                   B9          B3          B10
PLAT712_ALERT_1_C ANGLE   Unknown or Inconsistent Label .........          B9 Check
                   B9          B5          B11
PLAT712_ALERT_1_C ANGLE   Unknown or Inconsistent Label .........          B9 Check
                   B9          B10         B12
PLAT712_ALERT_1_C ANGLE   Unknown or Inconsistent Label .........          B9 Check
                   B9          B11         B12
PLAT712_ALERT_1_C ANGLE   Unknown or Inconsistent Label .........          B9 Check
                   B9          B12         B10
PLAT712_ALERT_1_C ANGLE   Unknown or Inconsistent Label .........          B9 Check
                   B9          B12         B11
PLAT712_ALERT_1_C ANGLE   Unknown or Inconsistent Label .........         B10 Check
                   B10         B8          B12
PLAT712_ALERT_1_C ANGLE   Unknown or Inconsistent Label .........         B10 Check
                   B10         B9          B11
PLAT712_ALERT_1_C ANGLE   Unknown or Inconsistent Label .........         B10 Check
                   B10         B9          B12
PLAT712_ALERT_1_C ANGLE   Unknown or Inconsistent Label .........         B10 Check
                   B10         B12         B11
PLAT712_ALERT_1_C ANGLE   Unknown or Inconsistent Label .........         B11 Check
                   B11         B7          B12
PLAT712_ALERT_1_C ANGLE   Unknown or Inconsistent Label .........         B11 Check
                   B11         B9          B12
PLAT712_ALERT_1_C ANGLE   Unknown or Inconsistent Label .........          B7 Check
                   B1          B7          H17
PLAT712_ALERT_1_C ANGLE   Unknown or Inconsistent Label .........          B8 Check
                   B1          B8          H18
PLAT712_ALERT_1_C ANGLE   Unknown or Inconsistent Label .........          B9 Check
                   B3          B9          H19
PLAT712_ALERT_1_C ANGLE   Unknown or Inconsistent Label .........         B10 Check
                   B3          B10         H20
PLAT712_ALERT_1_C ANGLE   Unknown or Inconsistent Label .........          B8 Check
                   B4          B8          H18
PLAT712_ALERT_1_C ANGLE   Unknown or Inconsistent Label .........         B10 Check
                   B4          B10         H20
PLAT712_ALERT_1_C ANGLE   Unknown or Inconsistent Label .........          B9 Check
                   B5          B9          H19
PLAT712_ALERT_1_C ANGLE   Unknown or Inconsistent Label .........         B11 Check
                   B5          B11         H21
PLAT712_ALERT_1_C ANGLE   Unknown or Inconsistent Label .........          B7 Check
                   B6          B7          H17
PLAT712_ALERT_1_C ANGLE   Unknown or Inconsistent Label .........         B11 Check
                   B6          B11         H21
PLAT712_ALERT_1_C ANGLE   Unknown or Inconsistent Label .........          B7 Check
```

```
                   B7          B1          H13
PLAT712_ALERT_1_C ANGLE   Unknown or Inconsistent Label .........          B7 Check
                   B7          B6          H15
PLAT712_ALERT_1_C ANGLE   Unknown or Inconsistent Label .........          B7 Check
                   B7          B8          H18
PLAT712_ALERT_1_C ANGLE   Unknown or Inconsistent Label .........          B7 Check
                   B7          B11         H21
PLAT712_ALERT_1_C ANGLE   Unknown or Inconsistent Label .........          B7 Check
                   B7          B12         H22
PLAT712_ALERT_1_C ANGLE   Unknown or Inconsistent Label .........          B8 Check
                   B8          B1          H13
PLAT712_ALERT_1_C ANGLE   Unknown or Inconsistent Label .........          B8 Check
                   B8          B4          H12
PLAT712_ALERT_1_C ANGLE   Unknown or Inconsistent Label .........          B8 Check
                   B8          B7          H17
PLAT712_ALERT_1_C ANGLE   Unknown or Inconsistent Label .........          B8 Check
                   B8          B10         H20
PLAT712_ALERT_1_C ANGLE   Unknown or Inconsistent Label .........          B8 Check
                   B8          B12         H22
PLAT712_ALERT_1_C ANGLE   Unknown or Inconsistent Label .........          B9 Check
                   B9          B3          H16
PLAT712_ALERT_1_C ANGLE   Unknown or Inconsistent Label .........          B9 Check
                   B9          B5          H11
PLAT712_ALERT_1_C ANGLE   Unknown or Inconsistent Label .........          B9 Check
                   B9          B10         H20
PLAT712_ALERT_1_C ANGLE   Unknown or Inconsistent Label .........          B9 Check
                   B9          B11         H21
PLAT712_ALERT_1_C ANGLE   Unknown or Inconsistent Label .........          B9 Check
                   B9          B12         H22
PLAT712_ALERT_1_C ANGLE   Unknown or Inconsistent Label .........         B10 Check
                   B10         B3          H16
PLAT712_ALERT_1_C ANGLE   Unknown or Inconsistent Label .........         B10 Check
                   B10         B4          H12
PLAT712_ALERT_1_C ANGLE   Unknown or Inconsistent Label .........         B10 Check
                   B10         B8          H18
PLAT712_ALERT_1_C ANGLE   Unknown or Inconsistent Label .........         B10 Check
                   B10         B9          H19
PLAT712_ALERT_1_C ANGLE   Unknown or Inconsistent Label .........         B10 Check
                   B10         B12         H22
PLAT712_ALERT_1_C ANGLE   Unknown or Inconsistent Label .........         B11 Check
                   B11         B5          H11
PLAT712_ALERT_1_C ANGLE   Unknown or Inconsistent Label .........         B11 Check
                   B11         B6          H15
PLAT712_ALERT_1_C ANGLE   Unknown or Inconsistent Label .........         B11 Check
                   B11         B7          H17
PLAT712_ALERT_1_C ANGLE   Unknown or Inconsistent Label .........         B11 Check
                   B11         B9          H19
PLAT712_ALERT_1_C ANGLE   Unknown or Inconsistent Label .........         B11 Check
                   B11         B12         H22
PLAT712_ALERT_1_C ANGLE   Unknown or Inconsistent Label .........         B12 Check
                   B12         B7          H17
PLAT712_ALERT_1_C ANGLE   Unknown or Inconsistent Label .........         B12 Check
                   B12         B8          H18
PLAT712_ALERT_1_C ANGLE   Unknown or Inconsistent Label .........         B12 Check
                   B12         B9          H19
PLAT712_ALERT_1_C ANGLE   Unknown or Inconsistent Label .........         B12 Check
                   B12         B10         H20
```

```
PLAT712_ALERT_1_C ANGLE   Unknown or Inconsistent Label ..........        B12 Check
                    B12         B11         H21
PLAT713_ALERT_1_C TORSION Unknown or Inconsistent Label ..........         B9 Check
                    B1          B2          B3          B9
PLAT713_ALERT_1_C TORSION Unknown or Inconsistent Label ..........        B10 Check
                    B1          B2          B3          B10
PLAT713_ALERT_1_C TORSION Unknown or Inconsistent Label ..........         B8 Check
                    B1          B2          B4          B8
PLAT713_ALERT_1_C TORSION Unknown or Inconsistent Label ..........        B10 Check
                    B1          B2          B4          B10
PLAT713_ALERT_1_C TORSION Unknown or Inconsistent Label ..........         B9 Check
                    B1          B2          B5          B9
PLAT713_ALERT_1_C TORSION Unknown or Inconsistent Label ..........        B11 Check
                    B1          B2          B5          B11
PLAT713_ALERT_1_C TORSION Unknown or Inconsistent Label ..........         B7 Check
                    B1          B2          B6          B7
PLAT713_ALERT_1_C TORSION Unknown or Inconsistent Label ..........        B11 Check
                    B1          B2          B6          B11
PLAT713_ALERT_1_C TORSION Unknown or Inconsistent Label ..........         B9 Check
                    B1          B4          B3          B9
PLAT713_ALERT_1_C TORSION Unknown or Inconsistent Label ..........        B10 Check
                    B1          B4          B3          B10
PLAT713_ALERT_1_C TORSION Unknown or Inconsistent Label ..........         B8 Check
                    B1          B4          B8          B7
PLAT713_ALERT_1_C TORSION Unknown or Inconsistent Label ..........         B8 Check
                    B1          B4          B8          B10
PLAT713_ALERT_1_C TORSION Unknown or Inconsistent Label ..........         B8 Check
                    B1          B4          B8          B12
PLAT713_ALERT_1_C TORSION Unknown or Inconsistent Label ..........        B10 Check
                    B1          B4          B10         B3
PLAT713_ALERT_1_C TORSION Unknown or Inconsistent Label ..........        B10 Check
                    B1          B4          B10         B8
PLAT713_ALERT_1_C TORSION Unknown or Inconsistent Label ..........        B10 Check
                    B1          B4          B10         B9
PLAT713_ALERT_1_C TORSION Unknown or Inconsistent Label ..........        B10 Check
                    B1          B4          B10         B12
PLAT713_ALERT_1_C TORSION Unknown or Inconsistent Label ..........         B9 Check
                    B1          B6          B5          B9
PLAT713_ALERT_1_C TORSION Unknown or Inconsistent Label ..........        B11 Check
                    B1          B6          B5          B11
PLAT713_ALERT_1_C TORSION Unknown or Inconsistent Label ..........         B7 Check
                    B1          B6          B7          B8
PLAT713_ALERT_1_C TORSION Unknown or Inconsistent Label ..........         B7 Check
                    B1          B6          B7          B11
PLAT713_ALERT_1_C TORSION Unknown or Inconsistent Label ..........         B7 Check
                    B1          B6          B7          B12
PLAT713_ALERT_1_C TORSION Unknown or Inconsistent Label ..........        B11 Check
                    B1          B6          B11         B5
PLAT713_ALERT_1_C TORSION Unknown or Inconsistent Label ..........        B11 Check
                    B1          B6          B11         B7
PLAT713_ALERT_1_C TORSION Unknown or Inconsistent Label ..........        B11 Check
                    B1          B6          B11         B9
PLAT713_ALERT_1_C TORSION Unknown or Inconsistent Label ..........        B11 Check
                    B1          B6          B11         B12
PLAT713_ALERT_1_C TORSION Unknown or Inconsistent Label ..........         B7 Check
                    B1          B7          B6          B2
PLAT713_ALERT_1_C TORSION Unknown or Inconsistent Label ..........         B7 Check
```

```
                    B1          B7          B6          B5
PLAT713_ALERT_1_C TORSION Unknown or Inconsistent Label ..........          B7 Check
                    B1          B7          B6          B11
PLAT713_ALERT_1_C TORSION Unknown or Inconsistent Label ..........          B7 Check
                    B1          B7          B8          B4
PLAT713_ALERT_1_C TORSION Unknown or Inconsistent Label ..........          B7 Check
                    B1          B7          B8          B10
PLAT713_ALERT_1_C TORSION Unknown or Inconsistent Label ..........          B7 Check
                    B1          B7          B8          B12
PLAT713_ALERT_1_C TORSION Unknown or Inconsistent Label ..........          B7 Check
                    B1          B7          B11         B5
PLAT713_ALERT_1_C TORSION Unknown or Inconsistent Label ..........          B7 Check
                    B1          B7          B11         B6
PLAT713_ALERT_1_C TORSION Unknown or Inconsistent Label ..........          B7 Check
                    B1          B7          B11         B9
PLAT713_ALERT_1_C TORSION Unknown or Inconsistent Label ..........          B7 Check
                    B1          B7          B11         B12
PLAT713_ALERT_1_C TORSION Unknown or Inconsistent Label ..........          B7 Check
                    B1          B7          B12         B8
PLAT713_ALERT_1_C TORSION Unknown or Inconsistent Label ..........          B7 Check
                    B1          B7          B12         B9
PLAT713_ALERT_1_C TORSION Unknown or Inconsistent Label ..........          B7 Check
                    B1          B7          B12         B10
PLAT713_ALERT_1_C TORSION Unknown or Inconsistent Label ..........          B7 Check
                    B1          B7          B12         B11
PLAT713_ALERT_1_C TORSION Unknown or Inconsistent Label ..........          B8 Check
                    B1          B8          B4          B2
PLAT713_ALERT_1_C TORSION Unknown or Inconsistent Label ..........          B8 Check
                    B1          B8          B4          B3
PLAT713_ALERT_1_C TORSION Unknown or Inconsistent Label ..........          B8 Check
                    B1          B8          B4          B10
PLAT713_ALERT_1_C TORSION Unknown or Inconsistent Label ..........          B8 Check
                    B1          B8          B7          B6
PLAT713_ALERT_1_C TORSION Unknown or Inconsistent Label ..........          B8 Check
                    B1          B8          B7          B11
PLAT713_ALERT_1_C TORSION Unknown or Inconsistent Label ..........          B8 Check
                    B1          B8          B7          B12
PLAT713_ALERT_1_C TORSION Unknown or Inconsistent Label ..........          B8 Check
                    B1          B8          B10         B3
PLAT713_ALERT_1_C TORSION Unknown or Inconsistent Label ..........          B8 Check
                    B1          B8          B10         B4
PLAT713_ALERT_1_C TORSION Unknown or Inconsistent Label ..........          B8 Check
                    B1          B8          B10         B9
PLAT713_ALERT_1_C TORSION Unknown or Inconsistent Label ..........          B8 Check
                    B1          B8          B10         B12
PLAT713_ALERT_1_C TORSION Unknown or Inconsistent Label ..........          B8 Check
                    B1          B8          B12         B7
PLAT713_ALERT_1_C TORSION Unknown or Inconsistent Label ..........          B8 Check
                    B1          B8          B12         B9
PLAT713_ALERT_1_C TORSION Unknown or Inconsistent Label ..........          B8 Check
                    B1          B8          B12         B10
PLAT713_ALERT_1_C TORSION Unknown or Inconsistent Label ..........          B8 Check
                    B1          B8          B12         B11
PLAT713_ALERT_1_C TORSION Unknown or Inconsistent Label ..........          B8 Check
                    B2          B1          B4          B8
PLAT713_ALERT_1_C TORSION Unknown or Inconsistent Label ..........         B10 Check
                    B2          B1          B4          B10
```

```
PLAT713_ALERT_1_C TORSION Unknown or Inconsistent Label ..........         B7 Check
                   B2          B1          B6          B7
PLAT713_ALERT_1_C TORSION Unknown or Inconsistent Label ..........        B11 Check
                   B2          B1          B6          B11
PLAT713_ALERT_1_C TORSION Unknown or Inconsistent Label ..........         B7 Check
                   B2          B1          B7          B6
PLAT713_ALERT_1_C TORSION Unknown or Inconsistent Label ..........         B7 Check
                   B2          B1          B7          B8
PLAT713_ALERT_1_C TORSION Unknown or Inconsistent Label ..........         B7 Check
                   B2          B1          B7          B11
PLAT713_ALERT_1_C TORSION Unknown or Inconsistent Label ..........         B7 Check
                   B2          B1          B7          B12
PLAT713_ALERT_1_C TORSION Unknown or Inconsistent Label ..........         B8 Check
                   B2          B1          B8          B4
PLAT713_ALERT_1_C TORSION Unknown or Inconsistent Label ..........         B8 Check
                   B2          B1          B8          B7
PLAT713_ALERT_1_C TORSION Unknown or Inconsistent Label ..........         B8 Check
                   B2          B1          B8          B10
PLAT713_ALERT_1_C TORSION Unknown or Inconsistent Label ..........         B8 Check
                   B2          B1          B8          B12
PLAT713_ALERT_1_C TORSION Unknown or Inconsistent Label ..........         B8 Check
                   B2          B3          B4          B8
PLAT713_ALERT_1_C TORSION Unknown or Inconsistent Label ..........        B10 Check
                   B2          B3          B4          B10
PLAT713_ALERT_1_C TORSION Unknown or Inconsistent Label ..........         B9 Check
                   B2          B3          B5          B9
PLAT713_ALERT_1_C TORSION Unknown or Inconsistent Label ..........        B11 Check
                   B2          B3          B5          B11
PLAT713_ALERT_1_C TORSION Unknown or Inconsistent Label ..........         B9 Check
                   B2          B3          B9          B5
PLAT713_ALERT_1_C TORSION Unknown or Inconsistent Label ..........         B9 Check
                   B2          B3          B9          B10
PLAT713_ALERT_1_C TORSION Unknown or Inconsistent Label ..........         B9 Check
                   B2          B3          B9          B11
PLAT713_ALERT_1_C TORSION Unknown or Inconsistent Label ..........         B9 Check
                   B2          B3          B9          B12
PLAT713_ALERT_1_C TORSION Unknown or Inconsistent Label ..........        B10 Check
                   B2          B3          B10         B4
PLAT713_ALERT_1_C TORSION Unknown or Inconsistent Label ..........        B10 Check
                   B2          B3          B10         B8
PLAT713_ALERT_1_C TORSION Unknown or Inconsistent Label ..........        B10 Check
                   B2          B3          B10         B9
PLAT713_ALERT_1_C TORSION Unknown or Inconsistent Label ..........        B10 Check
                   B2          B3          B10         B12
PLAT713_ALERT_1_C TORSION Unknown or Inconsistent Label ..........         B7 Check
                   B2          B4          B1          B7
PLAT713_ALERT_1_C TORSION Unknown or Inconsistent Label ..........         B8 Check
                   B2          B4          B1          B8
PLAT713_ALERT_1_C TORSION Unknown or Inconsistent Label ..........         B9 Check
                   B2          B4          B3          B9
PLAT713_ALERT_1_C TORSION Unknown or Inconsistent Label ..........        B10 Check
                   B2          B4          B3          B10
PLAT713_ALERT_1_C TORSION Unknown or Inconsistent Label ..........         B8 Check
                   B2          B4          B8          B7
PLAT713_ALERT_1_C TORSION Unknown or Inconsistent Label ..........         B8 Check
                   B2          B4          B8          B10
PLAT713_ALERT_1_C TORSION Unknown or Inconsistent Label ..........         B8 Check
```

```
                    B2          B4          B8          B12
PLAT713_ALERT_1_C TORSION Unknown or Inconsistent Label ..........        B10 Check
                    B2          B4          B10         B3
PLAT713_ALERT_1_C TORSION Unknown or Inconsistent Label ..........        B10 Check
                    B2          B4          B10         B8
PLAT713_ALERT_1_C TORSION Unknown or Inconsistent Label ..........        B10 Check
                    B2          B4          B10         B9
PLAT713_ALERT_1_C TORSION Unknown or Inconsistent Label ..........        B10 Check
                    B2          B4          B10         B12
PLAT713_ALERT_1_C TORSION Unknown or Inconsistent Label ..........         B9 Check
                    B2          B5          B3          B9
PLAT713_ALERT_1_C TORSION Unknown or Inconsistent Label ..........        B10 Check
                    B2          B5          B3          B10
PLAT713_ALERT_1_C TORSION Unknown or Inconsistent Label ..........         B7 Check
                    B2          B5          B6          B7
PLAT713_ALERT_1_C TORSION Unknown or Inconsistent Label ..........        B11 Check
                    B2          B5          B6          B11
PLAT713_ALERT_1_C TORSION Unknown or Inconsistent Label ..........         B9 Check
                    B2          B5          B9          B3
PLAT713_ALERT_1_C TORSION Unknown or Inconsistent Label ..........         B9 Check
                    B2          B5          B9          B10
PLAT713_ALERT_1_C TORSION Unknown or Inconsistent Label ..........         B9 Check
                    B2          B5          B9          B11
PLAT713_ALERT_1_C TORSION Unknown or Inconsistent Label ..........         B9 Check
                    B2          B5          B9          B12
PLAT713_ALERT_1_C TORSION Unknown or Inconsistent Label ..........        B11 Check
                    B2          B5          B11         B6
PLAT713_ALERT_1_C TORSION Unknown or Inconsistent Label ..........        B11 Check
                    B2          B5          B11         B7
PLAT713_ALERT_1_C TORSION Unknown or Inconsistent Label ..........        B11 Check
                    B2          B5          B11         B9
PLAT713_ALERT_1_C TORSION Unknown or Inconsistent Label ..........        B11 Check
                    B2          B5          B11         B12
PLAT713_ALERT_1_C TORSION Unknown or Inconsistent Label ..........         B7 Check
                    B2          B6          B1          B7
PLAT713_ALERT_1_C TORSION Unknown or Inconsistent Label ..........         B8 Check
                    B2          B6          B1          B8
PLAT713_ALERT_1_C TORSION Unknown or Inconsistent Label ..........         B9 Check
                    B2          B6          B5          B9
PLAT713_ALERT_1_C TORSION Unknown or Inconsistent Label ..........        B11 Check
                    B2          B6          B5          B11
PLAT713_ALERT_1_C TORSION Unknown or Inconsistent Label ..........         B7 Check
                    B2          B6          B7          B8
PLAT713_ALERT_1_C TORSION Unknown or Inconsistent Label ..........         B7 Check
                    B2          B6          B7          B11
PLAT713_ALERT_1_C TORSION Unknown or Inconsistent Label ..........         B7 Check
                    B2          B6          B7          B12
PLAT713_ALERT_1_C TORSION Unknown or Inconsistent Label ..........        B11 Check
                    B2          B6          B11         B5
PLAT713_ALERT_1_C TORSION Unknown or Inconsistent Label ..........        B11 Check
                    B2          B6          B11         B7
PLAT713_ALERT_1_C TORSION Unknown or Inconsistent Label ..........        B11 Check
                    B2          B6          B11         B9
PLAT713_ALERT_1_C TORSION Unknown or Inconsistent Label ..........        B11 Check
                    B2          B6          B11         B12
PLAT713_ALERT_1_C TORSION Unknown or Inconsistent Label ..........         B7 Check
                    B3          B2          B1          B7
```

```
PLAT713_ALERT_1_C TORSION Unknown or Inconsistent Label ..........          B8 Check
                    B3          B2          B1          B8
PLAT713_ALERT_1_C TORSION Unknown or Inconsistent Label ..........          B8 Check
                    B3          B2          B4          B8
PLAT713_ALERT_1_C TORSION Unknown or Inconsistent Label ..........         B10 Check
                    B3          B2          B4          B10
PLAT713_ALERT_1_C TORSION Unknown or Inconsistent Label ..........          B9 Check
                    B3          B2          B5          B9
PLAT713_ALERT_1_C TORSION Unknown or Inconsistent Label ..........         B11 Check
                    B3          B2          B5          B11
PLAT713_ALERT_1_C TORSION Unknown or Inconsistent Label ..........          B7 Check
                    B3          B2          B6          B7
PLAT713_ALERT_1_C TORSION Unknown or Inconsistent Label ..........         B11 Check
                    B3          B2          B6          B11
PLAT713_ALERT_1_C TORSION Unknown or Inconsistent Label ..........          B7 Check
                    B3          B4          B1          B7
PLAT713_ALERT_1_C TORSION Unknown or Inconsistent Label ..........          B8 Check
                    B3          B4          B1          B8
PLAT713_ALERT_1_C TORSION Unknown or Inconsistent Label ..........          B8 Check
                    B3          B4          B8          B7
PLAT713_ALERT_1_C TORSION Unknown or Inconsistent Label ..........          B8 Check
                    B3          B4          B8          B10
PLAT713_ALERT_1_C TORSION Unknown or Inconsistent Label ..........          B8 Check
                    B3          B4          B8          B12
PLAT713_ALERT_1_C TORSION Unknown or Inconsistent Label ..........         B10 Check
                    B3          B4          B10         B8
PLAT713_ALERT_1_C TORSION Unknown or Inconsistent Label ..........         B10 Check
                    B3          B4          B10         B9
PLAT713_ALERT_1_C TORSION Unknown or Inconsistent Label ..........         B10 Check
                    B3          B4          B10         B12
PLAT713_ALERT_1_C TORSION Unknown or Inconsistent Label ..........          B7 Check
                    B3          B5          B6          B7
PLAT713_ALERT_1_C TORSION Unknown or Inconsistent Label ..........         B11 Check
                    B3          B5          B6          B11
PLAT713_ALERT_1_C TORSION Unknown or Inconsistent Label ..........          B9 Check
                    B3          B5          B9          B10
PLAT713_ALERT_1_C TORSION Unknown or Inconsistent Label ..........          B9 Check
                    B3          B5          B9          B11
PLAT713_ALERT_1_C TORSION Unknown or Inconsistent Label ..........          B9 Check
                    B3          B5          B9          B12
PLAT713_ALERT_1_C TORSION Unknown or Inconsistent Label ..........         B11 Check
                    B3          B5          B11         B6
PLAT713_ALERT_1_C TORSION Unknown or Inconsistent Label ..........         B11 Check
                    B3          B5          B11         B7
PLAT713_ALERT_1_C TORSION Unknown or Inconsistent Label ..........         B11 Check
                    B3          B5          B11         B9
PLAT713_ALERT_1_C TORSION Unknown or Inconsistent Label ..........         B11 Check
                    B3          B5          B11         B12
PLAT713_ALERT_1_C TORSION Unknown or Inconsistent Label ..........          B9 Check
                    B3          B9          B5          B6
PLAT713_ALERT_1_C TORSION Unknown or Inconsistent Label ..........          B9 Check
                    B3          B9          B5          B11
PLAT713_ALERT_1_C TORSION Unknown or Inconsistent Label ..........          B9 Check
                    B3          B9          B10         B4
PLAT713_ALERT_1_C TORSION Unknown or Inconsistent Label ..........          B9 Check
                    B3          B9          B10         B8
PLAT713_ALERT_1_C TORSION Unknown or Inconsistent Label ..........          B9 Check
```

```
                   B3          B9          B10         B12
PLAT713_ALERT_1_C TORSION Unknown or Inconsistent Label ..........         B9 Check
                   B3          B9          B11         B5
PLAT713_ALERT_1_C TORSION Unknown or Inconsistent Label ..........         B9 Check
                   B3          B9          B11         B6
PLAT713_ALERT_1_C TORSION Unknown or Inconsistent Label ..........         B9 Check
                   B3          B9          B11         B7
PLAT713_ALERT_1_C TORSION Unknown or Inconsistent Label ..........         B9 Check
                   B3          B9          B11         B12
PLAT713_ALERT_1_C TORSION Unknown or Inconsistent Label ..........         B9 Check
                   B3          B9          B12         B7
PLAT713_ALERT_1_C TORSION Unknown or Inconsistent Label ..........         B9 Check
                   B3          B9          B12         B8
PLAT713_ALERT_1_C TORSION Unknown or Inconsistent Label ..........         B9 Check
                   B3          B9          B12         B10
PLAT713_ALERT_1_C TORSION Unknown or Inconsistent Label ..........         B9 Check
                   B3          B9          B12         B11
PLAT713_ALERT_1_C TORSION Unknown or Inconsistent Label ..........        B10 Check
                   B3          B10         B4          B8
PLAT713_ALERT_1_C TORSION Unknown or Inconsistent Label ..........        B10 Check
                   B3          B10         B8          B4
PLAT713_ALERT_1_C TORSION Unknown or Inconsistent Label ..........        B10 Check
                   B3          B10         B8          B7
PLAT713_ALERT_1_C TORSION Unknown or Inconsistent Label ..........        B10 Check
                   B3          B10         B8          B12
PLAT713_ALERT_1_C TORSION Unknown or Inconsistent Label ..........        B10 Check
                   B3          B10         B9          B5
PLAT713_ALERT_1_C TORSION Unknown or Inconsistent Label ..........        B10 Check
                   B3          B10         B9          B11
PLAT713_ALERT_1_C TORSION Unknown or Inconsistent Label ..........        B10 Check
                   B3          B10         B9          B12
PLAT713_ALERT_1_C TORSION Unknown or Inconsistent Label ..........        B10 Check
                   B3          B10         B12         B7
PLAT713_ALERT_1_C TORSION Unknown or Inconsistent Label ..........        B10 Check
                   B3          B10         B12         B8
PLAT713_ALERT_1_C TORSION Unknown or Inconsistent Label ..........        B10 Check
                   B3          B10         B12         B9
PLAT713_ALERT_1_C TORSION Unknown or Inconsistent Label ..........        B10 Check
                   B3          B10         B12         B11
PLAT713_ALERT_1_C TORSION Unknown or Inconsistent Label ..........         B7 Check
                   B4          B1          B6          B7
PLAT713_ALERT_1_C TORSION Unknown or Inconsistent Label ..........        B11 Check
                   B4          B1          B6          B11
PLAT713_ALERT_1_C TORSION Unknown or Inconsistent Label ..........         B7 Check
                   B4          B1          B7          B6
PLAT713_ALERT_1_C TORSION Unknown or Inconsistent Label ..........         B7 Check
                   B4          B1          B7          B8
PLAT713_ALERT_1_C TORSION Unknown or Inconsistent Label ..........         B7 Check
                   B4          B1          B7          B11
PLAT713_ALERT_1_C TORSION Unknown or Inconsistent Label ..........         B7 Check
                   B4          B1          B7          B12
PLAT713_ALERT_1_C TORSION Unknown or Inconsistent Label ..........         B8 Check
                   B4          B1          B8          B7
PLAT713_ALERT_1_C TORSION Unknown or Inconsistent Label ..........         B8 Check
                   B4          B1          B8          B10
PLAT713_ALERT_1_C TORSION Unknown or Inconsistent Label ..........         B8 Check
                   B4          B1          B8          B12
```

```
PLAT713_ALERT_1_C TORSION Unknown or Inconsistent Label ..........         B7 Check
                    B4          B2          B1          B7
PLAT713_ALERT_1_C TORSION Unknown or Inconsistent Label ..........         B8 Check
                    B4          B2          B1          B8
PLAT713_ALERT_1_C TORSION Unknown or Inconsistent Label ..........         B9 Check
                    B4          B2          B3          B9
PLAT713_ALERT_1_C TORSION Unknown or Inconsistent Label ..........        B10 Check
                    B4          B2          B3          B10
PLAT713_ALERT_1_C TORSION Unknown or Inconsistent Label ..........         B9 Check
                    B4          B2          B5          B9
PLAT713_ALERT_1_C TORSION Unknown or Inconsistent Label ..........        B11 Check
                    B4          B2          B5          B11
PLAT713_ALERT_1_C TORSION Unknown or Inconsistent Label ..........         B7 Check
                    B4          B2          B6          B7
PLAT713_ALERT_1_C TORSION Unknown or Inconsistent Label ..........        B11 Check
                    B4          B2          B6          B11
PLAT713_ALERT_1_C TORSION Unknown or Inconsistent Label ..........         B9 Check
                    B4          B3          B5          B9
PLAT713_ALERT_1_C TORSION Unknown or Inconsistent Label ..........        B11 Check
                    B4          B3          B5          B11
PLAT713_ALERT_1_C TORSION Unknown or Inconsistent Label ..........         B9 Check
                    B4          B3          B9          B5
PLAT713_ALERT_1_C TORSION Unknown or Inconsistent Label ..........         B9 Check
                    B4          B3          B9          B10
PLAT713_ALERT_1_C TORSION Unknown or Inconsistent Label ..........         B9 Check
                    B4          B3          B9          B11
PLAT713_ALERT_1_C TORSION Unknown or Inconsistent Label ..........         B9 Check
                    B4          B3          B9          B12
PLAT713_ALERT_1_C TORSION Unknown or Inconsistent Label ..........        B10 Check
                    B4          B3          B10         B8
PLAT713_ALERT_1_C TORSION Unknown or Inconsistent Label ..........        B10 Check
                    B4          B3          B10         B9
PLAT713_ALERT_1_C TORSION Unknown or Inconsistent Label ..........        B10 Check
                    B4          B3          B10         B12
PLAT713_ALERT_1_C TORSION Unknown or Inconsistent Label ..........         B8 Check
                    B4          B8          B1          B6
PLAT713_ALERT_1_C TORSION Unknown or Inconsistent Label ..........         B8 Check
                    B4          B8          B1          B7
PLAT713_ALERT_1_C TORSION Unknown or Inconsistent Label ..........         B8 Check
                    B4          B8          B7          B6
PLAT713_ALERT_1_C TORSION Unknown or Inconsistent Label ..........         B8 Check
                    B4          B8          B7          B11
PLAT713_ALERT_1_C TORSION Unknown or Inconsistent Label ..........         B8 Check
                    B4          B8          B7          B12
PLAT713_ALERT_1_C TORSION Unknown or Inconsistent Label ..........         B8 Check
                    B4          B8          B10         B9
PLAT713_ALERT_1_C TORSION Unknown or Inconsistent Label ..........         B8 Check
                    B4          B8          B10         B12
PLAT713_ALERT_1_C TORSION Unknown or Inconsistent Label ..........         B8 Check
                    B4          B8          B12         B7
PLAT713_ALERT_1_C TORSION Unknown or Inconsistent Label ..........         B8 Check
                    B4          B8          B12         B9
PLAT713_ALERT_1_C TORSION Unknown or Inconsistent Label ..........         B8 Check
                    B4          B8          B12         B10
PLAT713_ALERT_1_C TORSION Unknown or Inconsistent Label ..........         B8 Check
                    B4          B8          B12         B11
PLAT713_ALERT_1_C TORSION Unknown or Inconsistent Label ..........        B10 Check
```

```
                    B4          B10         B3          B5
PLAT713_ALERT_1_C TORSION Unknown or Inconsistent Label ..........        B10 Check
                    B4          B10         B3          B9
PLAT713_ALERT_1_C TORSION Unknown or Inconsistent Label ..........        B10 Check
                    B4          B10         B8          B7
PLAT713_ALERT_1_C TORSION Unknown or Inconsistent Label ..........        B10 Check
                    B4          B10         B8          B12
PLAT713_ALERT_1_C TORSION Unknown or Inconsistent Label ..........        B10 Check
                    B4          B10         B9          B5
PLAT713_ALERT_1_C TORSION Unknown or Inconsistent Label ..........        B10 Check
                    B4          B10         B9          B11
PLAT713_ALERT_1_C TORSION Unknown or Inconsistent Label ..........        B10 Check
                    B4          B10         B9          B12
PLAT713_ALERT_1_C TORSION Unknown or Inconsistent Label ..........        B10 Check
                    B4          B10         B12         B7
PLAT713_ALERT_1_C TORSION Unknown or Inconsistent Label ..........        B10 Check
                    B4          B10         B12         B8
PLAT713_ALERT_1_C TORSION Unknown or Inconsistent Label ..........        B10 Check
                    B4          B10         B12         B9
PLAT713_ALERT_1_C TORSION Unknown or Inconsistent Label ..........        B10 Check
                    B4          B10         B12         B11
PLAT713_ALERT_1_C TORSION Unknown or Inconsistent Label ..........         B7 Check
                    B5          B2          B1          B7
PLAT713_ALERT_1_C TORSION Unknown or Inconsistent Label ..........         B8 Check
                    B5          B2          B1          B8
PLAT713_ALERT_1_C TORSION Unknown or Inconsistent Label ..........         B9 Check
                    B5          B2          B3          B9
PLAT713_ALERT_1_C TORSION Unknown or Inconsistent Label ..........        B10 Check
                    B5          B2          B3          B10
PLAT713_ALERT_1_C TORSION Unknown or Inconsistent Label ..........         B8 Check
                    B5          B2          B4          B8
PLAT713_ALERT_1_C TORSION Unknown or Inconsistent Label ..........        B10 Check
                    B5          B2          B4          B10
PLAT713_ALERT_1_C TORSION Unknown or Inconsistent Label ..........         B7 Check
                    B5          B2          B6          B7
PLAT713_ALERT_1_C TORSION Unknown or Inconsistent Label ..........        B11 Check
                    B5          B2          B6          B11
PLAT713_ALERT_1_C TORSION Unknown or Inconsistent Label ..........         B8 Check
                    B5          B3          B4          B8
PLAT713_ALERT_1_C TORSION Unknown or Inconsistent Label ..........        B10 Check
                    B5          B3          B4          B10
PLAT713_ALERT_1_C TORSION Unknown or Inconsistent Label ..........         B9 Check
                    B5          B3          B9          B10
PLAT713_ALERT_1_C TORSION Unknown or Inconsistent Label ..........         B9 Check
                    B5          B3          B9          B11
PLAT713_ALERT_1_C TORSION Unknown or Inconsistent Label ..........         B9 Check
                    B5          B3          B9          B12
PLAT713_ALERT_1_C TORSION Unknown or Inconsistent Label ..........        B10 Check
                    B5          B3          B10         B8
PLAT713_ALERT_1_C TORSION Unknown or Inconsistent Label ..........        B10 Check
                    B5          B3          B10         B9
PLAT713_ALERT_1_C TORSION Unknown or Inconsistent Label ..........        B10 Check
                    B5          B3          B10         B12
PLAT713_ALERT_1_C TORSION Unknown or Inconsistent Label ..........         B7 Check
                    B5          B6          B1          B7
PLAT713_ALERT_1_C TORSION Unknown or Inconsistent Label ..........         B8 Check
                    B5          B6          B1          B8
```

```
PLAT713_ALERT_1_C TORSION Unknown or Inconsistent Label ..........         B7 Check
                    B5          B6          B7          B8
PLAT713_ALERT_1_C TORSION Unknown or Inconsistent Label ..........         B7 Check
                    B5          B6          B7          B11
PLAT713_ALERT_1_C TORSION Unknown or Inconsistent Label ..........         B7 Check
                    B5          B6          B7          B12
PLAT713_ALERT_1_C TORSION Unknown or Inconsistent Label ..........        B11 Check
                    B5          B6          B11         B7
PLAT713_ALERT_1_C TORSION Unknown or Inconsistent Label ..........        B11 Check
                    B5          B6          B11         B9
PLAT713_ALERT_1_C TORSION Unknown or Inconsistent Label ..........        B11 Check
                    B5          B6          B11         B12
PLAT713_ALERT_1_C TORSION Unknown or Inconsistent Label ..........         B9 Check
                    B5          B9          B3          B10
PLAT713_ALERT_1_C TORSION Unknown or Inconsistent Label ..........         B9 Check
                    B5          B9          B10         B8
PLAT713_ALERT_1_C TORSION Unknown or Inconsistent Label ..........         B9 Check
                    B5          B9          B10         B12
PLAT713_ALERT_1_C TORSION Unknown or Inconsistent Label ..........         B9 Check
                    B5          B9          B11         B6
PLAT713_ALERT_1_C TORSION Unknown or Inconsistent Label ..........         B9 Check
                    B5          B9          B11         B7
PLAT713_ALERT_1_C TORSION Unknown or Inconsistent Label ..........         B9 Check
                    B5          B9          B11         B12
PLAT713_ALERT_1_C TORSION Unknown or Inconsistent Label ..........         B9 Check
                    B5          B9          B12         B7
PLAT713_ALERT_1_C TORSION Unknown or Inconsistent Label ..........         B9 Check
                    B5          B9          B12         B8
PLAT713_ALERT_1_C TORSION Unknown or Inconsistent Label ..........         B9 Check
                    B5          B9          B12         B10
PLAT713_ALERT_1_C TORSION Unknown or Inconsistent Label ..........         B9 Check
                    B5          B9          B12         B11
PLAT713_ALERT_1_C TORSION Unknown or Inconsistent Label ..........        B11 Check
                    B5          B11         B6          B7
PLAT713_ALERT_1_C TORSION Unknown or Inconsistent Label ..........        B11 Check
                    B5          B11         B7          B6
PLAT713_ALERT_1_C TORSION Unknown or Inconsistent Label ..........        B11 Check
                    B5          B11         B7          B8
PLAT713_ALERT_1_C TORSION Unknown or Inconsistent Label ..........        B11 Check
                    B5          B11         B7          B12
PLAT713_ALERT_1_C TORSION Unknown or Inconsistent Label ..........        B11 Check
                    B5          B11         B9          B10
PLAT713_ALERT_1_C TORSION Unknown or Inconsistent Label ..........        B11 Check
                    B5          B11         B9          B12
PLAT713_ALERT_1_C TORSION Unknown or Inconsistent Label ..........        B11 Check
                    B5          B11         B12         B7
PLAT713_ALERT_1_C TORSION Unknown or Inconsistent Label ..........        B11 Check
                    B5          B11         B12         B8
PLAT713_ALERT_1_C TORSION Unknown or Inconsistent Label ..........        B11 Check
                    B5          B11         B12         B9
PLAT713_ALERT_1_C TORSION Unknown or Inconsistent Label ..........        B11 Check
                    B5          B11         B12         B10
PLAT713_ALERT_1_C TORSION Unknown or Inconsistent Label ..........         B8 Check
                    B6          B1          B4          B8
PLAT713_ALERT_1_C TORSION Unknown or Inconsistent Label ..........        B10 Check
                    B6          B1          B4          B10
PLAT713_ALERT_1_C TORSION Unknown or Inconsistent Label ..........         B7 Check
```

```
                    B6          B1          B7          B8
PLAT713_ALERT_1_C TORSION Unknown or Inconsistent Label ..........          B7 Check
                    B6          B1          B7          B11
PLAT713_ALERT_1_C TORSION Unknown or Inconsistent Label ..........          B7 Check
                    B6          B1          B7          B12
PLAT713_ALERT_1_C TORSION Unknown or Inconsistent Label ..........          B8 Check
                    B6          B1          B8          B7
PLAT713_ALERT_1_C TORSION Unknown or Inconsistent Label ..........          B8 Check
                    B6          B1          B8          B10
PLAT713_ALERT_1_C TORSION Unknown or Inconsistent Label ..........          B8 Check
                    B6          B1          B8          B12
PLAT713_ALERT_1_C TORSION Unknown or Inconsistent Label ..........          B7 Check
                    B6          B2          B1          B7
PLAT713_ALERT_1_C TORSION Unknown or Inconsistent Label ..........          B8 Check
                    B6          B2          B1          B8
PLAT713_ALERT_1_C TORSION Unknown or Inconsistent Label ..........          B9 Check
                    B6          B2          B3          B9
PLAT713_ALERT_1_C TORSION Unknown or Inconsistent Label ..........         B10 Check
                    B6          B2          B3          B10
PLAT713_ALERT_1_C TORSION Unknown or Inconsistent Label ..........          B8 Check
                    B6          B2          B4          B8
PLAT713_ALERT_1_C TORSION Unknown or Inconsistent Label ..........         B10 Check
                    B6          B2          B4          B10
PLAT713_ALERT_1_C TORSION Unknown or Inconsistent Label ..........          B9 Check
                    B6          B2          B5          B9
PLAT713_ALERT_1_C TORSION Unknown or Inconsistent Label ..........         B11 Check
                    B6          B2          B5          B11
PLAT713_ALERT_1_C TORSION Unknown or Inconsistent Label ..........          B9 Check
                    B6          B5          B3          B9
PLAT713_ALERT_1_C TORSION Unknown or Inconsistent Label ..........         B10 Check
                    B6          B5          B3          B10
PLAT713_ALERT_1_C TORSION Unknown or Inconsistent Label ..........          B9 Check
                    B6          B5          B9          B10
PLAT713_ALERT_1_C TORSION Unknown or Inconsistent Label ..........          B9 Check
                    B6          B5          B9          B11
PLAT713_ALERT_1_C TORSION Unknown or Inconsistent Label ..........          B9 Check
                    B6          B5          B9          B12
PLAT713_ALERT_1_C TORSION Unknown or Inconsistent Label ..........         B11 Check
                    B6          B5          B11         B7
PLAT713_ALERT_1_C TORSION Unknown or Inconsistent Label ..........         B11 Check
                    B6          B5          B11         B9
PLAT713_ALERT_1_C TORSION Unknown or Inconsistent Label ..........         B11 Check
                    B6          B5          B11         B12
PLAT713_ALERT_1_C TORSION Unknown or Inconsistent Label ..........          B7 Check
                    B6          B7          B1          B8
PLAT713_ALERT_1_C TORSION Unknown or Inconsistent Label ..........          B7 Check
                    B6          B7          B8          B10
PLAT713_ALERT_1_C TORSION Unknown or Inconsistent Label ..........          B7 Check
                    B6          B7          B8          B12
PLAT713_ALERT_1_C TORSION Unknown or Inconsistent Label ..........          B7 Check
                    B6          B7          B11         B9
PLAT713_ALERT_1_C TORSION Unknown or Inconsistent Label ..........          B7 Check
                    B6          B7          B11         B12
PLAT713_ALERT_1_C TORSION Unknown or Inconsistent Label ..........          B7 Check
                    B6          B7          B12         B8
PLAT713_ALERT_1_C TORSION Unknown or Inconsistent Label ..........          B7 Check
                    B6          B7          B12         B9
```

```
PLAT713_ALERT_1_C TORSION Unknown or Inconsistent Label ..........         B7 Check
                    B6          B7          B12         B10
PLAT713_ALERT_1_C TORSION Unknown or Inconsistent Label ..........         B7 Check
                    B6          B7          B12         B11
PLAT713_ALERT_1_C TORSION Unknown or Inconsistent Label ..........        B11 Check
                    B6          B11         B5          B9
PLAT713_ALERT_1_C TORSION Unknown or Inconsistent Label ..........        B11 Check
                    B6          B11         B7          B8
PLAT713_ALERT_1_C TORSION Unknown or Inconsistent Label ..........        B11 Check
                    B6          B11         B7          B12
PLAT713_ALERT_1_C TORSION Unknown or Inconsistent Label ..........        B11 Check
                    B6          B11         B9          B10
PLAT713_ALERT_1_C TORSION Unknown or Inconsistent Label ..........        B11 Check
                    B6          B11         B9          B12
PLAT713_ALERT_1_C TORSION Unknown or Inconsistent Label ..........        B11 Check
                    B6          B11         B12         B7
PLAT713_ALERT_1_C TORSION Unknown or Inconsistent Label ..........        B11 Check
                    B6          B11         B12         B8
PLAT713_ALERT_1_C TORSION Unknown or Inconsistent Label ..........        B11 Check
                    B6          B11         B12         B9
PLAT713_ALERT_1_C TORSION Unknown or Inconsistent Label ..........        B11 Check
                    B6          B11         B12         B10
PLAT713_ALERT_1_C TORSION Unknown or Inconsistent Label ..........         B7 Check
                    B7          B1          B4          B8
PLAT713_ALERT_1_C TORSION Unknown or Inconsistent Label ..........         B7 Check
                    B7          B1          B4          B10
PLAT713_ALERT_1_C TORSION Unknown or Inconsistent Label ..........         B7 Check
                    B7          B1          B6          B11
PLAT713_ALERT_1_C TORSION Unknown or Inconsistent Label ..........         B7 Check
                    B7          B1          B8          B10
PLAT713_ALERT_1_C TORSION Unknown or Inconsistent Label ..........         B7 Check
                    B7          B1          B8          B12
PLAT713_ALERT_1_C TORSION Unknown or Inconsistent Label ..........         B7 Check
                    B7          B6          B1          B8
PLAT713_ALERT_1_C TORSION Unknown or Inconsistent Label ..........         B7 Check
                    B7          B6          B5          B9
PLAT713_ALERT_1_C TORSION Unknown or Inconsistent Label ..........         B7 Check
                    B7          B6          B5          B11
PLAT713_ALERT_1_C TORSION Unknown or Inconsistent Label ..........         B7 Check
                    B7          B6          B11         B9
PLAT713_ALERT_1_C TORSION Unknown or Inconsistent Label ..........         B7 Check
                    B7          B6          B11         B12
PLAT713_ALERT_1_C TORSION Unknown or Inconsistent Label ..........         B7 Check
                    B7          B8          B4          B10
PLAT713_ALERT_1_C TORSION Unknown or Inconsistent Label ..........         B7 Check
                    B7          B8          B10         B9
PLAT713_ALERT_1_C TORSION Unknown or Inconsistent Label ..........         B7 Check
                    B7          B8          B10         B12
PLAT713_ALERT_1_C TORSION Unknown or Inconsistent Label ..........         B7 Check
                    B7          B8          B12         B9
PLAT713_ALERT_1_C TORSION Unknown or Inconsistent Label ..........         B7 Check
                    B7          B8          B12         B10
PLAT713_ALERT_1_C TORSION Unknown or Inconsistent Label ..........         B7 Check
                    B7          B8          B12         B11
PLAT713_ALERT_1_C TORSION Unknown or Inconsistent Label ..........         B7 Check
                    B7          B11         B5          B9
PLAT713_ALERT_1_C TORSION Unknown or Inconsistent Label ..........         B7 Check
```

```
                    B7          B11         B9          B10
PLAT713_ALERT_1_C TORSION Unknown or Inconsistent Label ..........         B7 Check
                    B7          B11         B9          B12
PLAT713_ALERT_1_C TORSION Unknown or Inconsistent Label ..........         B7 Check
                    B7          B11         B12         B8
PLAT713_ALERT_1_C TORSION Unknown or Inconsistent Label ..........         B7 Check
                    B7          B11         B12         B9
PLAT713_ALERT_1_C TORSION Unknown or Inconsistent Label ..........         B7 Check
                    B7          B11         B12         B10
PLAT713_ALERT_1_C TORSION Unknown or Inconsistent Label ..........         B7 Check
                    B7          B12         B8          B10
PLAT713_ALERT_1_C TORSION Unknown or Inconsistent Label ..........         B7 Check
                    B7          B12         B9          B10
PLAT713_ALERT_1_C TORSION Unknown or Inconsistent Label ..........         B7 Check
                    B7          B12         B9          B11
PLAT713_ALERT_1_C TORSION Unknown or Inconsistent Label ..........         B7 Check
                    B7          B12         B10         B8
PLAT713_ALERT_1_C TORSION Unknown or Inconsistent Label ..........         B7 Check
                    B7          B12         B10         B9
PLAT713_ALERT_1_C TORSION Unknown or Inconsistent Label ..........         B7 Check
                    B7          B12         B11         B9
PLAT713_ALERT_1_C TORSION Unknown or Inconsistent Label ..........         B8 Check
                    B8          B1          B4          B10
PLAT713_ALERT_1_C TORSION Unknown or Inconsistent Label ..........         B8 Check
                    B8          B1          B6          B11
PLAT713_ALERT_1_C TORSION Unknown or Inconsistent Label ..........         B8 Check
                    B8          B1          B7          B11
PLAT713_ALERT_1_C TORSION Unknown or Inconsistent Label ..........         B8 Check
                    B8          B1          B7          B12
PLAT713_ALERT_1_C TORSION Unknown or Inconsistent Label ..........         B8 Check
                    B8          B4          B3          B9
PLAT713_ALERT_1_C TORSION Unknown or Inconsistent Label ..........         B8 Check
                    B8          B4          B3          B10
PLAT713_ALERT_1_C TORSION Unknown or Inconsistent Label ..........         B8 Check
                    B8          B4          B10         B9
PLAT713_ALERT_1_C TORSION Unknown or Inconsistent Label ..........         B8 Check
                    B8          B4          B10         B12
PLAT713_ALERT_1_C TORSION Unknown or Inconsistent Label ..........         B8 Check
                    B8          B7          B6          B11
PLAT713_ALERT_1_C TORSION Unknown or Inconsistent Label ..........         B8 Check
                    B8          B7          B11         B9
PLAT713_ALERT_1_C TORSION Unknown or Inconsistent Label ..........         B8 Check
                    B8          B7          B11         B12
PLAT713_ALERT_1_C TORSION Unknown or Inconsistent Label ..........         B8 Check
                    B8          B7          B12         B9
PLAT713_ALERT_1_C TORSION Unknown or Inconsistent Label ..........         B8 Check
                    B8          B7          B12         B10
PLAT713_ALERT_1_C TORSION Unknown or Inconsistent Label ..........         B8 Check
                    B8          B7          B12         B11
PLAT713_ALERT_1_C TORSION Unknown or Inconsistent Label ..........         B8 Check
                    B8          B10         B3          B9
PLAT713_ALERT_1_C TORSION Unknown or Inconsistent Label ..........         B8 Check
                    B8          B10         B9          B11
PLAT713_ALERT_1_C TORSION Unknown or Inconsistent Label ..........         B8 Check
                    B8          B10         B9          B12
PLAT713_ALERT_1_C TORSION Unknown or Inconsistent Label ..........         B8 Check
                    B8          B10         B12         B9
```

```
PLAT713_ALERT_1_C TORSION Unknown or Inconsistent Label ..........          B8 Check
                    B8          B10         B12         B11
PLAT713_ALERT_1_C TORSION Unknown or Inconsistent Label ..........          B8 Check
                    B8          B12         B7          B11
PLAT713_ALERT_1_C TORSION Unknown or Inconsistent Label ..........          B8 Check
                    B8          B12         B9          B10
PLAT713_ALERT_1_C TORSION Unknown or Inconsistent Label ..........          B8 Check
                    B8          B12         B9          B11
PLAT713_ALERT_1_C TORSION Unknown or Inconsistent Label ..........          B8 Check
                    B8          B12         B10         B9
PLAT713_ALERT_1_C TORSION Unknown or Inconsistent Label ..........          B8 Check
                    B8          B12         B11         B9
PLAT713_ALERT_1_C TORSION Unknown or Inconsistent Label ..........          B9 Check
                    B9          B3          B4          B10
PLAT713_ALERT_1_C TORSION Unknown or Inconsistent Label ..........          B9 Check
                    B9          B3          B5          B11
PLAT713_ALERT_1_C TORSION Unknown or Inconsistent Label ..........          B9 Check
                    B9          B3          B10         B12
PLAT713_ALERT_1_C TORSION Unknown or Inconsistent Label ..........          B9 Check
                    B9          B5          B3          B10
PLAT713_ALERT_1_C TORSION Unknown or Inconsistent Label ..........          B9 Check
                    B9          B5          B6          B11
PLAT713_ALERT_1_C TORSION Unknown or Inconsistent Label ..........          B9 Check
                    B9          B5          B11         B12
PLAT713_ALERT_1_C TORSION Unknown or Inconsistent Label ..........          B9 Check
                    B9          B10         B8          B12
PLAT713_ALERT_1_C TORSION Unknown or Inconsistent Label ..........          B9 Check
                    B9          B10         B12         B11
PLAT713_ALERT_1_C TORSION Unknown or Inconsistent Label ..........          B9 Check
                    B9          B11         B7          B12
PLAT713_ALERT_1_C TORSION Unknown or Inconsistent Label ..........          B9 Check
                    B9          B11         B12         B10
PLAT713_ALERT_1_C TORSION Unknown or Inconsistent Label ..........          B9 Check
                    B9          B12         B7          B11
PLAT713_ALERT_1_C TORSION Unknown or Inconsistent Label ..........          B9 Check
                    B9          B12         B8          B10
PLAT713_ALERT_1_C TORSION Unknown or Inconsistent Label ..........         B10 Check
                    B10         B3          B5          B11
PLAT713_ALERT_1_C TORSION Unknown or Inconsistent Label ..........         B10 Check
                    B10         B3          B9          B11
PLAT713_ALERT_1_C TORSION Unknown or Inconsistent Label ..........         B10 Check
                    B10         B3          B9          B12
PLAT713_ALERT_1_C TORSION Unknown or Inconsistent Label ..........         B10 Check
                    B10         B4          B8          B12
PLAT713_ALERT_1_C TORSION Unknown or Inconsistent Label ..........         B10 Check
                    B10         B8          B7          B11
PLAT713_ALERT_1_C TORSION Unknown or Inconsistent Label ..........         B10 Check
                    B10         B8          B7          B12
PLAT713_ALERT_1_C TORSION Unknown or Inconsistent Label ..........         B10 Check
                    B10         B8          B12         B11
PLAT713_ALERT_1_C TORSION Unknown or Inconsistent Label ..........         B10 Check
                    B10         B9          B5          B11
PLAT713_ALERT_1_C TORSION Unknown or Inconsistent Label ..........         B10 Check
                    B10         B9          B11         B12
PLAT713_ALERT_1_C TORSION Unknown or Inconsistent Label ..........         B10 Check
                    B10         B9          B12         B11
PLAT713_ALERT_1_C TORSION Unknown or Inconsistent Label ..........         B10 Check
```

```
                  B10         B12         B7          B11
PLAT713_ALERT_1_C TORSION Unknown or Inconsistent Label ..........        B10 Check
                  B10         B12         B9          B11
PLAT713_ALERT_1_C TORSION Unknown or Inconsistent Label ..........        B11 Check
                  B11         B5          B9          B12
PLAT713_ALERT_1_C TORSION Unknown or Inconsistent Label ..........        B11 Check
                  B11         B6          B7          B12
PLAT713_ALERT_1_C TORSION Unknown or Inconsistent Label ..........        B11 Check
                  B11         B7          B8          B12
PLAT713_ALERT_1_C TORSION Unknown or Inconsistent Label ..........        B11 Check
                  B11         B9          B10         B12
PLAT713_ALERT_1_C TORSION Unknown or Inconsistent Label ..........         B8 Check
                  B1          B4          B8          H18
PLAT713_ALERT_1_C TORSION Unknown or Inconsistent Label ..........        B10 Check
                  B1          B4          B10         H20
PLAT713_ALERT_1_C TORSION Unknown or Inconsistent Label ..........         B7 Check
                  B1          B6          B7          H17
PLAT713_ALERT_1_C TORSION Unknown or Inconsistent Label ..........        B11 Check
                  B1          B6          B11         H21
PLAT713_ALERT_1_C TORSION Unknown or Inconsistent Label ..........         B7 Check
                  B1          B7          B6          H15
PLAT713_ALERT_1_C TORSION Unknown or Inconsistent Label ..........         B7 Check
                  B1          B7          B8          H18
PLAT713_ALERT_1_C TORSION Unknown or Inconsistent Label ..........         B7 Check
                  B1          B7          B11         H21
PLAT713_ALERT_1_C TORSION Unknown or Inconsistent Label ..........         B7 Check
                  B1          B7          B12         H22
PLAT713_ALERT_1_C TORSION Unknown or Inconsistent Label ..........         B8 Check
                  B1          B8          B4          H12
PLAT713_ALERT_1_C TORSION Unknown or Inconsistent Label ..........         B8 Check
                  B1          B8          B7          H17
PLAT713_ALERT_1_C TORSION Unknown or Inconsistent Label ..........         B8 Check
                  B1          B8          B10         H20
PLAT713_ALERT_1_C TORSION Unknown or Inconsistent Label ..........         B8 Check
                  B1          B8          B12         H22
PLAT713_ALERT_1_C TORSION Unknown or Inconsistent Label ..........         B7 Check
                  B2          B1          B7          H17
PLAT713_ALERT_1_C TORSION Unknown or Inconsistent Label ..........         B8 Check
                  B2          B1          B8          H18
PLAT713_ALERT_1_C TORSION Unknown or Inconsistent Label ..........         B9 Check
                  B2          B3          B9          H19
PLAT713_ALERT_1_C TORSION Unknown or Inconsistent Label ..........        B10 Check
                  B2          B3          B10         H20
PLAT713_ALERT_1_C TORSION Unknown or Inconsistent Label ..........         B8 Check
                  B2          B4          B8          H18
PLAT713_ALERT_1_C TORSION Unknown or Inconsistent Label ..........        B10 Check
                  B2          B4          B10         H20
PLAT713_ALERT_1_C TORSION Unknown or Inconsistent Label ..........         B9 Check
                  B2          B5          B9          H19
PLAT713_ALERT_1_C TORSION Unknown or Inconsistent Label ..........        B11 Check
                  B2          B5          B11         H21
PLAT713_ALERT_1_C TORSION Unknown or Inconsistent Label ..........         B7 Check
                  B2          B6          B7          H17
PLAT713_ALERT_1_C TORSION Unknown or Inconsistent Label ..........        B11 Check
                  B2          B6          B11         H21
PLAT713_ALERT_1_C TORSION Unknown or Inconsistent Label ..........         B8 Check
                  B3          B4          B8          H18
```

```
PLAT713_ALERT_1_C TORSION Unknown or Inconsistent Label ..........        B10 Check
                   B3          B4          B10         H20
PLAT713_ALERT_1_C TORSION Unknown or Inconsistent Label ..........         B9 Check
                   B3          B5          B9          H19
PLAT713_ALERT_1_C TORSION Unknown or Inconsistent Label ..........        B11 Check
                   B3          B5          B11         H21
PLAT713_ALERT_1_C TORSION Unknown or Inconsistent Label ..........         B9 Check
                   B3          B9          B5          H11
PLAT713_ALERT_1_C TORSION Unknown or Inconsistent Label ..........         B9 Check
                   B3          B9          B10         H20
PLAT713_ALERT_1_C TORSION Unknown or Inconsistent Label ..........         B9 Check
                   B3          B9          B11         H21
PLAT713_ALERT_1_C TORSION Unknown or Inconsistent Label ..........         B9 Check
                   B3          B9          B12         H22
PLAT713_ALERT_1_C TORSION Unknown or Inconsistent Label ..........        B10 Check
                   B3          B10         B4          H12
PLAT713_ALERT_1_C TORSION Unknown or Inconsistent Label ..........        B10 Check
                   B3          B10         B8          H18
PLAT713_ALERT_1_C TORSION Unknown or Inconsistent Label ..........        B10 Check
                   B3          B10         B9          H19
PLAT713_ALERT_1_C TORSION Unknown or Inconsistent Label ..........        B10 Check
                   B3          B10         B12         H22
PLAT713_ALERT_1_C TORSION Unknown or Inconsistent Label ..........         B7 Check
                   B4          B1          B7          H17
PLAT713_ALERT_1_C TORSION Unknown or Inconsistent Label ..........         B8 Check
                   B4          B1          B8          H18
PLAT713_ALERT_1_C TORSION Unknown or Inconsistent Label ..........         B9 Check
                   B4          B3          B9          H19
PLAT713_ALERT_1_C TORSION Unknown or Inconsistent Label ..........        B10 Check
                   B4          B3          B10         H20
PLAT713_ALERT_1_C TORSION Unknown or Inconsistent Label ..........         B8 Check
                   B4          B8          B1          H13
PLAT713_ALERT_1_C TORSION Unknown or Inconsistent Label ..........         B8 Check
                   B4          B8          B7          H17
PLAT713_ALERT_1_C TORSION Unknown or Inconsistent Label ..........         B8 Check
                   B4          B8          B10         H20
PLAT713_ALERT_1_C TORSION Unknown or Inconsistent Label ..........         B8 Check
                   B4          B8          B12         H22
PLAT713_ALERT_1_C TORSION Unknown or Inconsistent Label ..........        B10 Check
                   B4          B10         B3          H16
PLAT713_ALERT_1_C TORSION Unknown or Inconsistent Label ..........        B10 Check
                   B4          B10         B8          H18
PLAT713_ALERT_1_C TORSION Unknown or Inconsistent Label ..........        B10 Check
                   B4          B10         B9          H19
PLAT713_ALERT_1_C TORSION Unknown or Inconsistent Label ..........        B10 Check
                   B4          B10         B12         H22
PLAT713_ALERT_1_C TORSION Unknown or Inconsistent Label ..........         B9 Check
                   B5          B3          B9          H19
PLAT713_ALERT_1_C TORSION Unknown or Inconsistent Label ..........        B10 Check
                   B5          B3          B10         H20
PLAT713_ALERT_1_C TORSION Unknown or Inconsistent Label ..........         B7 Check
                   B5          B6          B7          H17
PLAT713_ALERT_1_C TORSION Unknown or Inconsistent Label ..........        B11 Check
                   B5          B6          B11         H21
PLAT713_ALERT_1_C TORSION Unknown or Inconsistent Label ..........         B9 Check
                   B5          B9          B3          H16
PLAT713_ALERT_1_C TORSION Unknown or Inconsistent Label ..........         B9 Check
```

```
                    B5          B9          B10         H20
PLAT713_ALERT_1_C TORSION Unknown or Inconsistent Label ..........          B9 Check
                    B5          B9          B11         H21
PLAT713_ALERT_1_C TORSION Unknown or Inconsistent Label ..........          B9 Check
                    B5          B9          B12         H22
PLAT713_ALERT_1_C TORSION Unknown or Inconsistent Label ..........         B11 Check
                    B5          B11         B6          H15
PLAT713_ALERT_1_C TORSION Unknown or Inconsistent Label ..........         B11 Check
                    B5          B11         B7          H17
PLAT713_ALERT_1_C TORSION Unknown or Inconsistent Label ..........         B11 Check
                    B5          B11         B9          H19
PLAT713_ALERT_1_C TORSION Unknown or Inconsistent Label ..........         B11 Check
                    B5          B11         B12         H22
PLAT713_ALERT_1_C TORSION Unknown or Inconsistent Label ..........          B7 Check
                    B6          B1          B7          H17
PLAT713_ALERT_1_C TORSION Unknown or Inconsistent Label ..........          B8 Check
                    B6          B1          B8          H18
PLAT713_ALERT_1_C TORSION Unknown or Inconsistent Label ..........          B9 Check
                    B6          B5          B9          H19
PLAT713_ALERT_1_C TORSION Unknown or Inconsistent Label ..........         B11 Check
                    B6          B5          B11         H21
PLAT713_ALERT_1_C TORSION Unknown or Inconsistent Label ..........          B7 Check
                    B6          B7          B1          H13
PLAT713_ALERT_1_C TORSION Unknown or Inconsistent Label ..........          B7 Check
                    B6          B7          B8          H18
PLAT713_ALERT_1_C TORSION Unknown or Inconsistent Label ..........          B7 Check
                    B6          B7          B11         H21
PLAT713_ALERT_1_C TORSION Unknown or Inconsistent Label ..........          B7 Check
                    B6          B7          B12         H22
PLAT713_ALERT_1_C TORSION Unknown or Inconsistent Label ..........         B11 Check
                    B6          B11         B5          H11
PLAT713_ALERT_1_C TORSION Unknown or Inconsistent Label ..........         B11 Check
                    B6          B11         B7          H17
PLAT713_ALERT_1_C TORSION Unknown or Inconsistent Label ..........         B11 Check
                    B6          B11         B9          H19
PLAT713_ALERT_1_C TORSION Unknown or Inconsistent Label ..........         B11 Check
                    B6          B11         B12         H22
PLAT713_ALERT_1_C TORSION Unknown or Inconsistent Label ..........          B7 Check
                    B7          B1          B8          H18
PLAT713_ALERT_1_C TORSION Unknown or Inconsistent Label ..........          B7 Check
                    B7          B6          B11         H21
PLAT713_ALERT_1_C TORSION Unknown or Inconsistent Label ..........          B7 Check
                    B7          B8          B10         H20
PLAT713_ALERT_1_C TORSION Unknown or Inconsistent Label ..........          B7 Check
                    B7          B8          B12         H22
PLAT713_ALERT_1_C TORSION Unknown or Inconsistent Label ..........          B7 Check
                    B7          B11         B9          H19
PLAT713_ALERT_1_C TORSION Unknown or Inconsistent Label ..........          B7 Check
                    B7          B11         B12         H22
PLAT713_ALERT_1_C TORSION Unknown or Inconsistent Label ..........          B7 Check
                    B7          B12         B8          H18
PLAT713_ALERT_1_C TORSION Unknown or Inconsistent Label ..........          B7 Check
                    B7          B12         B9          H19
PLAT713_ALERT_1_C TORSION Unknown or Inconsistent Label ..........          B7 Check
                    B7          B12         B10         H20
PLAT713_ALERT_1_C TORSION Unknown or Inconsistent Label ..........          B7 Check
                    B7          B12         B11         H21
```

```
PLAT713_ALERT_1_C TORSION Unknown or Inconsistent Label ..........        B8 Check
                   B8         B1          B7          H17
PLAT713_ALERT_1_C TORSION Unknown or Inconsistent Label ..........        B8 Check
                   B8         B4          B10         H20
PLAT713_ALERT_1_C TORSION Unknown or Inconsistent Label ..........        B8 Check
                   B8         B7          B11         H21
PLAT713_ALERT_1_C TORSION Unknown or Inconsistent Label ..........        B8 Check
                   B8         B7          B12         H22
PLAT713_ALERT_1_C TORSION Unknown or Inconsistent Label ..........        B8 Check
                   B8         B10         B9          H19
PLAT713_ALERT_1_C TORSION Unknown or Inconsistent Label ..........        B8 Check
                   B8         B10         B12         H22
PLAT713_ALERT_1_C TORSION Unknown or Inconsistent Label ..........        B8 Check
                   B8         B12         B7          H17
PLAT713_ALERT_1_C TORSION Unknown or Inconsistent Label ..........        B8 Check
                   B8         B12         B9          H19
PLAT713_ALERT_1_C TORSION Unknown or Inconsistent Label ..........        B8 Check
                   B8         B12         B10         H20
PLAT713_ALERT_1_C TORSION Unknown or Inconsistent Label ..........        B8 Check
                   B8         B12         B11         H21
PLAT713_ALERT_1_C TORSION Unknown or Inconsistent Label ..........        B9 Check
                   B9         B3          B10         H20
PLAT713_ALERT_1_C TORSION Unknown or Inconsistent Label ..........        B9 Check
                   B9         B5          B11         H21
PLAT713_ALERT_1_C TORSION Unknown or Inconsistent Label ..........        B9 Check
                   B9         B10         B8          H18
PLAT713_ALERT_1_C TORSION Unknown or Inconsistent Label ..........        B9 Check
                   B9         B10         B12         H22
PLAT713_ALERT_1_C TORSION Unknown or Inconsistent Label ..........        B9 Check
                   B9         B11         B7          H17
PLAT713_ALERT_1_C TORSION Unknown or Inconsistent Label ..........        B9 Check
                   B9         B11         B12         H22
PLAT713_ALERT_1_C TORSION Unknown or Inconsistent Label ..........        B9 Check
                   B9         B12         B7          H17
PLAT713_ALERT_1_C TORSION Unknown or Inconsistent Label ..........        B9 Check
                   B9         B12         B8          H18
PLAT713_ALERT_1_C TORSION Unknown or Inconsistent Label ..........        B9 Check
                   B9         B12         B10         H20
PLAT713_ALERT_1_C TORSION Unknown or Inconsistent Label ..........        B9 Check
                   B9         B12         B11         H21
PLAT713_ALERT_1_C TORSION Unknown or Inconsistent Label ..........       B10 Check
                   B10        B3          B9          H19
PLAT713_ALERT_1_C TORSION Unknown or Inconsistent Label ..........       B10 Check
                   B10        B4          B8          H18
PLAT713_ALERT_1_C TORSION Unknown or Inconsistent Label ..........       B10 Check
                   B10        B8          B7          H17
PLAT713_ALERT_1_C TORSION Unknown or Inconsistent Label ..........       B10 Check
                   B10        B8          B12         H22
PLAT713_ALERT_1_C TORSION Unknown or Inconsistent Label ..........       B10 Check
                   B10        B9          B11         H21
PLAT713_ALERT_1_C TORSION Unknown or Inconsistent Label ..........       B10 Check
                   B10        B9          B12         H22
PLAT713_ALERT_1_C TORSION Unknown or Inconsistent Label ..........       B10 Check
                   B10        B12         B7          H17
PLAT713_ALERT_1_C TORSION Unknown or Inconsistent Label ..........       B10 Check
                   B10        B12         B8          H18
PLAT713_ALERT_1_C TORSION Unknown or Inconsistent Label ..........       B10 Check
```

```
                   B10         B12         B9          H19
PLAT713_ALERT_1_C TORSION Unknown or Inconsistent Label ..........        B10 Check
                   B10         B12         B11         H21
PLAT713_ALERT_1_C TORSION Unknown or Inconsistent Label ..........        B11 Check
                   B11         B5          B9          H19
PLAT713_ALERT_1_C TORSION Unknown or Inconsistent Label ..........        B11 Check
                   B11         B6          B7          H17
PLAT713_ALERT_1_C TORSION Unknown or Inconsistent Label ..........        B11 Check
                   B11         B7          B8          H18
PLAT713_ALERT_1_C TORSION Unknown or Inconsistent Label ..........        B11 Check
                   B11         B7          B12         H22
PLAT713_ALERT_1_C TORSION Unknown or Inconsistent Label ..........        B11 Check
                   B11         B9          B10         H20
PLAT713_ALERT_1_C TORSION Unknown or Inconsistent Label ..........        B11 Check
                   B11         B9          B12         H22
PLAT713_ALERT_1_C TORSION Unknown or Inconsistent Label ..........        B11 Check
                   B11         B12         B7          H17
PLAT713_ALERT_1_C TORSION Unknown or Inconsistent Label ..........        B11 Check
                   B11         B12         B8          H18
PLAT713_ALERT_1_C TORSION Unknown or Inconsistent Label ..........        B11 Check
                   B11         B12         B9          H19
PLAT713_ALERT_1_C TORSION Unknown or Inconsistent Label ..........        B11 Check
                   B11         B12         B10         H20
PLAT713_ALERT_1_C TORSION Unknown or Inconsistent Label ..........        B12 Check
                   B12         B7          B8          H18
PLAT713_ALERT_1_C TORSION Unknown or Inconsistent Label ..........        B12 Check
                   B12         B7          B11         H21
PLAT713_ALERT_1_C TORSION Unknown or Inconsistent Label ..........        B12 Check
                   B12         B8          B10         H20
PLAT713_ALERT_1_C TORSION Unknown or Inconsistent Label ..........        B12 Check
                   B12         B9          B10         H20
PLAT713_ALERT_1_C TORSION Unknown or Inconsistent Label ..........        B12 Check
                   B12         B9          B11         H21
PLAT713_ALERT_1_C TORSION Unknown or Inconsistent Label ..........        B12 Check
                   B12         B10         B8          H18
PLAT713_ALERT_1_C TORSION Unknown or Inconsistent Label ..........        B12 Check
                   B12         B10         B9          H19
PLAT713_ALERT_1_C TORSION Unknown or Inconsistent Label ..........        B12 Check
                   B12         B11         B9          H19
PLAT713_ALERT_1_C TORSION Unknown or Inconsistent Label ..........         B9 Check
                   H11         B5          B3          B9
PLAT713_ALERT_1_C TORSION Unknown or Inconsistent Label ..........        B10 Check
                   H11         B5          B3          B10
PLAT713_ALERT_1_C TORSION Unknown or Inconsistent Label ..........         B7 Check
                   H11         B5          B6          B7
PLAT713_ALERT_1_C TORSION Unknown or Inconsistent Label ..........        B11 Check
                   H11         B5          B6          B11
PLAT713_ALERT_1_C TORSION Unknown or Inconsistent Label ..........         B9 Check
                   H11         B5          B9          B10
PLAT713_ALERT_1_C TORSION Unknown or Inconsistent Label ..........         B9 Check
                   H11         B5          B9          B11
PLAT713_ALERT_1_C TORSION Unknown or Inconsistent Label ..........         B9 Check
                   H11         B5          B9          B12
PLAT713_ALERT_1_C TORSION Unknown or Inconsistent Label ..........        B11 Check
                   H11         B5          B11         B7
PLAT713_ALERT_1_C TORSION Unknown or Inconsistent Label ..........        B11 Check
                   H11         B5          B11         B9
```

```
PLAT713_ALERT_1_C TORSION Unknown or Inconsistent Label ..........        B11 Check
                   H11         B5          B11         B12
PLAT713_ALERT_1_C TORSION Unknown or Inconsistent Label ..........         B7 Check
                   H12         B4          B1          B7
PLAT713_ALERT_1_C TORSION Unknown or Inconsistent Label ..........         B8 Check
                   H12         B4          B1          B8
PLAT713_ALERT_1_C TORSION Unknown or Inconsistent Label ..........         B9 Check
                   H12         B4          B3          B9
PLAT713_ALERT_1_C TORSION Unknown or Inconsistent Label ..........        B10 Check
                   H12         B4          B3          B10
PLAT713_ALERT_1_C TORSION Unknown or Inconsistent Label ..........         B8 Check
                   H12         B4          B8          B7
PLAT713_ALERT_1_C TORSION Unknown or Inconsistent Label ..........         B8 Check
                   H12         B4          B8          B10
PLAT713_ALERT_1_C TORSION Unknown or Inconsistent Label ..........         B8 Check
                   H12         B4          B8          B12
PLAT713_ALERT_1_C TORSION Unknown or Inconsistent Label ..........        B10 Check
                   H12         B4          B10         B8
PLAT713_ALERT_1_C TORSION Unknown or Inconsistent Label ..........        B10 Check
                   H12         B4          B10         B9
PLAT713_ALERT_1_C TORSION Unknown or Inconsistent Label ..........        B10 Check
                   H12         B4          B10         B12
PLAT713_ALERT_1_C TORSION Unknown or Inconsistent Label ..........         B8 Check
                   H13         B1          B4          B8
PLAT713_ALERT_1_C TORSION Unknown or Inconsistent Label ..........        B10 Check
                   H13         B1          B4          B10
PLAT713_ALERT_1_C TORSION Unknown or Inconsistent Label ..........         B7 Check
                   H13         B1          B6          B7
PLAT713_ALERT_1_C TORSION Unknown or Inconsistent Label ..........        B11 Check
                   H13         B1          B6          B11
PLAT713_ALERT_1_C TORSION Unknown or Inconsistent Label ..........         B7 Check
                   H13         B1          B7          B8
PLAT713_ALERT_1_C TORSION Unknown or Inconsistent Label ..........         B7 Check
                   H13         B1          B7          B11
PLAT713_ALERT_1_C TORSION Unknown or Inconsistent Label ..........         B7 Check
                   H13         B1          B7          B12
PLAT713_ALERT_1_C TORSION Unknown or Inconsistent Label ..........         B8 Check
                   H13         B1          B8          B7
PLAT713_ALERT_1_C TORSION Unknown or Inconsistent Label ..........         B8 Check
                   H13         B1          B8          B10
PLAT713_ALERT_1_C TORSION Unknown or Inconsistent Label ..........         B8 Check
                   H13         B1          B8          B12
PLAT713_ALERT_1_C TORSION Unknown or Inconsistent Label ..........         B7 Check
                   H14         B2          B1          B7
PLAT713_ALERT_1_C TORSION Unknown or Inconsistent Label ..........         B8 Check
                   H14         B2          B1          B8
PLAT713_ALERT_1_C TORSION Unknown or Inconsistent Label ..........         B9 Check
                   H14         B2          B3          B9
PLAT713_ALERT_1_C TORSION Unknown or Inconsistent Label ..........        B10 Check
                   H14         B2          B3          B10
PLAT713_ALERT_1_C TORSION Unknown or Inconsistent Label ..........         B8 Check
                   H14         B2          B4          B8
PLAT713_ALERT_1_C TORSION Unknown or Inconsistent Label ..........        B10 Check
                   H14         B2          B4          B10
PLAT713_ALERT_1_C TORSION Unknown or Inconsistent Label ..........         B9 Check
                   H14         B2          B5          B9
PLAT713_ALERT_1_C TORSION Unknown or Inconsistent Label ..........        B11 Check
```

```
                    H14         B2          B5          B11
PLAT713_ALERT_1_C TORSION Unknown or Inconsistent Label ..........          B7 Check
                    H14         B2          B6          B7
PLAT713_ALERT_1_C TORSION Unknown or Inconsistent Label ..........         B11 Check
                    H14         B2          B6          B11
PLAT713_ALERT_1_C TORSION Unknown or Inconsistent Label ..........          B7 Check
                    H15         B6          B1          B7
PLAT713_ALERT_1_C TORSION Unknown or Inconsistent Label ..........          B8 Check
                    H15         B6          B1          B8
PLAT713_ALERT_1_C TORSION Unknown or Inconsistent Label ..........          B9 Check
                    H15         B6          B5          B9
PLAT713_ALERT_1_C TORSION Unknown or Inconsistent Label ..........         B11 Check
                    H15         B6          B5          B11
PLAT713_ALERT_1_C TORSION Unknown or Inconsistent Label ..........          B7 Check
                    H15         B6          B7          B8
PLAT713_ALERT_1_C TORSION Unknown or Inconsistent Label ..........          B7 Check
                    H15         B6          B7          B11
PLAT713_ALERT_1_C TORSION Unknown or Inconsistent Label ..........          B7 Check
                    H15         B6          B7          B12
PLAT713_ALERT_1_C TORSION Unknown or Inconsistent Label ..........         B11 Check
                    H15         B6          B11         B7
PLAT713_ALERT_1_C TORSION Unknown or Inconsistent Label ..........         B11 Check
                    H15         B6          B11         B9
PLAT713_ALERT_1_C TORSION Unknown or Inconsistent Label ..........         B11 Check
                    H15         B6          B11         B12
PLAT713_ALERT_1_C TORSION Unknown or Inconsistent Label ..........          B8 Check
                    H16         B3          B4          B8
PLAT713_ALERT_1_C TORSION Unknown or Inconsistent Label ..........         B10 Check
                    H16         B3          B4          B10
PLAT713_ALERT_1_C TORSION Unknown or Inconsistent Label ..........          B9 Check
                    H16         B3          B5          B9
PLAT713_ALERT_1_C TORSION Unknown or Inconsistent Label ..........         B11 Check
                    H16         B3          B5          B11
PLAT713_ALERT_1_C TORSION Unknown or Inconsistent Label ..........          B9 Check
                    H16         B3          B9          B10
PLAT713_ALERT_1_C TORSION Unknown or Inconsistent Label ..........          B9 Check
                    H16         B3          B9          B11
PLAT713_ALERT_1_C TORSION Unknown or Inconsistent Label ..........          B9 Check
                    H16         B3          B9          B12
PLAT713_ALERT_1_C TORSION Unknown or Inconsistent Label ..........         B10 Check
                    H16         B3          B10         B8
PLAT713_ALERT_1_C TORSION Unknown or Inconsistent Label ..........         B10 Check
                    H16         B3          B10         B9
PLAT713_ALERT_1_C TORSION Unknown or Inconsistent Label ..........         B10 Check
                    H16         B3          B10         B12
PLAT713_ALERT_1_C TORSION Unknown or Inconsistent Label ..........         H17 Check
                    H17         B7          B8          B12
PLAT713_ALERT_1_C TORSION Unknown or Inconsistent Label ..........         H17 Check
                    H17         B7          B11         B12
PLAT713_ALERT_1_C TORSION Unknown or Inconsistent Label ..........          B9 Check
                    H11         B5          B9          H19
PLAT713_ALERT_1_C TORSION Unknown or Inconsistent Label ..........         B11 Check
                    H11         B5          B11         H21
PLAT713_ALERT_1_C TORSION Unknown or Inconsistent Label ..........          B8 Check
                    H12         B4          B8          H18
PLAT713_ALERT_1_C TORSION Unknown or Inconsistent Label ..........         B10 Check
                    H12         B4          B10         H20
```

```
PLAT713_ALERT_1_C TORSION Unknown or Inconsistent Label ..........          B7 Check
                    H13         B1          B7          H17
PLAT713_ALERT_1_C TORSION Unknown or Inconsistent Label ..........          B8 Check
                    H13         B1          B8          H18
PLAT713_ALERT_1_C TORSION Unknown or Inconsistent Label ..........          B7 Check
                    H15         B6          B7          H17
PLAT713_ALERT_1_C TORSION Unknown or Inconsistent Label ..........         B11 Check
                    H15         B6          B11         H21
PLAT713_ALERT_1_C TORSION Unknown or Inconsistent Label ..........          B9 Check
                    H16         B3          B9          H19
PLAT713_ALERT_1_C TORSION Unknown or Inconsistent Label ..........         B10 Check
                    H16         B3          B10         H20
PLAT713_ALERT_1_C TORSION Unknown or Inconsistent Label ..........         H17 Check
                    H17         B7          B8          H18
PLAT713_ALERT_1_C TORSION Unknown or Inconsistent Label ..........         H17 Check
                    H17         B7          B11         H21
PLAT713_ALERT_1_C TORSION Unknown or Inconsistent Label ..........         H17 Check
                    H17         B7          B12         H22
PLAT713_ALERT_1_C TORSION Unknown or Inconsistent Label ..........         H18 Check
                    H18         B8          B10         H20
PLAT713_ALERT_1_C TORSION Unknown or Inconsistent Label ..........         H18 Check
                    H18         B8          B12         H22
PLAT713_ALERT_1_C TORSION Unknown or Inconsistent Label ..........         H19 Check
                    H19         B9          B10         H20
PLAT713_ALERT_1_C TORSION Unknown or Inconsistent Label ..........         H19 Check
                    H19         B9          B11         H21
PLAT713_ALERT_1_C TORSION Unknown or Inconsistent Label ..........         H19 Check
                    H19         B9          B12         H22
PLAT713_ALERT_1_C TORSION Unknown or Inconsistent Label ..........         H20 Check
                    H20         B10         B12         H22
PLAT713_ALERT_1_C TORSION Unknown or Inconsistent Label ..........         H21 Check
                    H21         B11         B12         H22
PLAT913_ALERT_3_C Missing # of Very Strong Reflections in FCF ....          12 Note
                1  1  0,   1  2  0,   2  0  0,   2  2  0,  -2  1  1,  -1  1  1,
               -1  2  1,   1  2  1,   2  2  1,   0  0  2,   1  1  2,   2  2  2,
PLAT992_ALERT_5_C Repd & Actual _reflns_number_gt Values Differ by          11 Check
```

Alert level G

```
ABSMU01_ALERT_1_G  Calculation of _exptl_absorpt_correction_mu
                not performed for this radiation type.
PLAT005_ALERT_5_G No Embedded Refinement Details Found  in the CIF      Please Do !
PLAT415_ALERT_2_G Short Inter D-H..H-X       H4       ..H11       .        1.93 Ang.
                                                1-x,1-y,2-z  =      2_667 Check
PLAT808_ALERT_5_G No Parseable SHELXL Style Weighting Scheme Found      Please Check
PLAT883_ALERT_1_G Absent Datum for _atom_sites_solution_primary ..      Please Do !
PLAT910_ALERT_3_G Missing FCF Reflection(s) Below Theta(Min)[Deg]=        2.21 Note
               -1  0  1,
PLAT911_ALERT_3_G Missing FCF Refl Between Thmin & STh/L=    0.600         236 Report
                0  6  0,   0  8  0,   1  1  0,   1  2  0,   2  0  0,   2  2  0,
                6  7  0,   8  5  0,   9  6  0,  10  0  0,  10  1  0,  10  4  0,
               11  3  0, -11  2  1, -10  2  1,  -8  4  1,  -8  7  1,  -8  8  1,
               -7  6  1,  -7  7  1,  -6  8  1,  -5  9  1,  -5 10  1,  -4  6  1,
               -4 10  1,  -3  0  1,  -3  9  1,  -2  1  1,  -1  1  1,  -1  2  1,
                    (  206 More Missing: see the .ckf listing file)
PLAT912_ALERT_4_G Missing # of FCF Reflections Above STh/L=  0.600        6197 Note
PLAT929_ALERT_5_G No Weight Pars,Obs and Calc R1,wR2,S not Checked           ! Info
```

```
PLAT961_ALERT_5_G Dataset Contains no Negative Intensities .......     Please Check
PLAT969_ALERT_5_G The 'Henn et al.' R-Factor-gap value ..........      0.855 Note
              Predicted wR2: Based on SigI**2 14.22 or SHELX Weight 14.22
PLAT978_ALERT_2_G Number C-C Bonds with Positive Residual Density.          6 Info
PLAT980_ALERT_1_G No Anomalous Scattering Factors Found in CIF ...     Please Check
```

```
   0 ALERT level A = Most likely a serious problem - resolve or explain
   4 ALERT level B = A potentially serious problem, consider carefully
 756 ALERT level C = Check. Ensure it is not caused by an omission or oversight
  13 ALERT level G = General information/check it is not something unexpected

 757 ALERT type 1 CIF construction/syntax error, inconsistent or missing data
   4 ALERT type 2 Indicator that the structure model may be wrong or deficient
   5 ALERT type 3 Indicator that the structure quality may be low
   1 ALERT type 4 Improvement, methodology, query or suggestion
   6 ALERT type 5 Informative message, check
```

It is advisable to attempt to resolve as many as possible of the alerts in all categories. Often the minor alerts point to easily fixed oversights, errors and omissions in your CIF or refinement strategy, so attention to these fine details can be worthwhile. In order to resolve some of the more serious problems it may be necessary to carry out additional measurements or structure refinements. However, the purpose of your study may justify the reported deviations and the more serious of these should normally be commented upon in the discussion or experimental section of a paper or in the "special_details" fields of the CIF. checkCIF was carefully designed to identify outliers and unusual parameters, but every test has its limitations and alerts that are not important in a particular case may appear. Conversely, the absence of alerts does not guarantee there are no aspects of the results needing attention. It is up to the individual to critically assess their own results and, if necessary, seek expert advice.

Publication of your CIF in IUCr journals

A basic structural check has been run on your CIF. These basic checks will be run on all CIFs submitted for publication in IUCr journals (*Acta Crystallographica*, *Journal of Applied Crystallography*, *Journal of Synchrotron Radiation*); however, if you intend to submit to *Acta Crystallographica Section C* or *E* or *IUCrData*, you should make sure that full publication checks are run on the final version of your CIF prior to submission.

Publication of your CIF in other journals

Please refer to the *Notes for Authors* of the relevant journal for any special instructions relating to CIF submission.

PLATON version of 04/06/2025; check.def file version of 30/05/2025

Datablock Bislutidinium_closo-dodecaborate12__IAM - ellipsoid plot

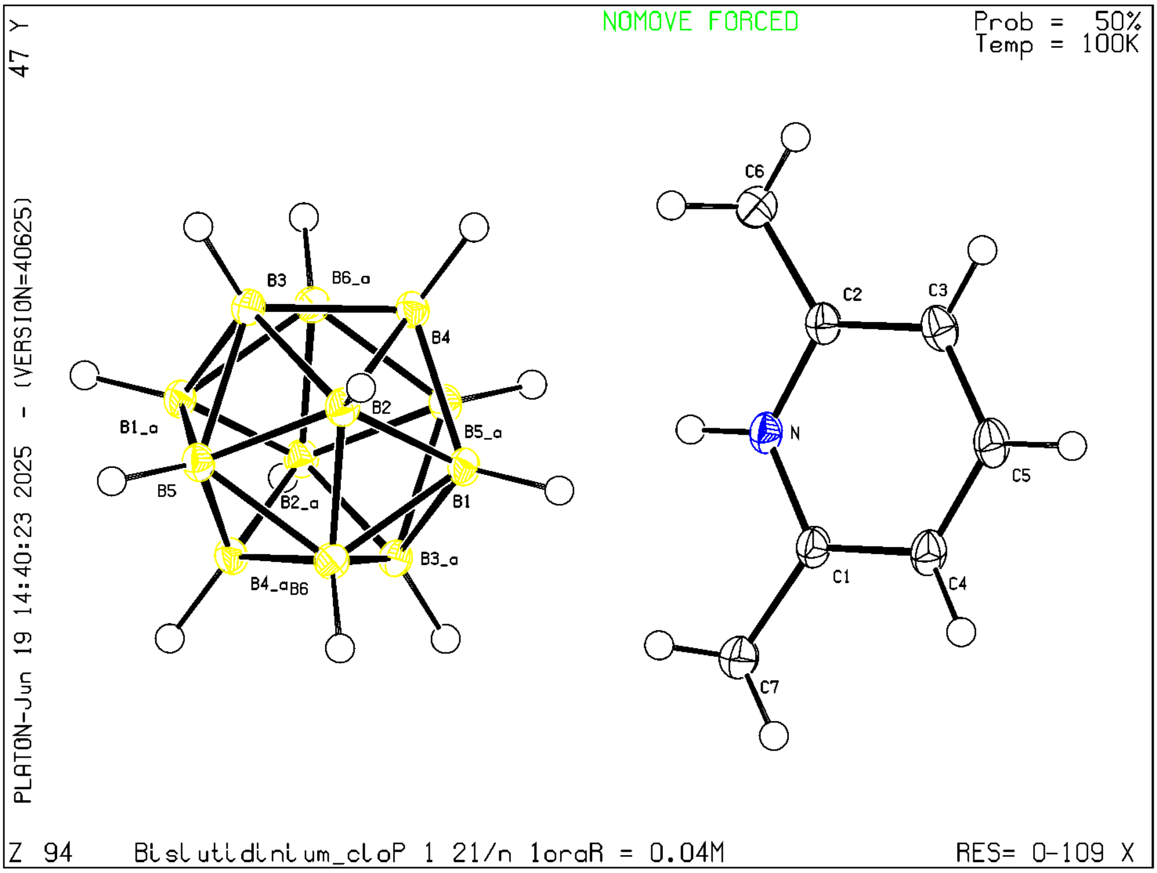

Supplement: Supplementary file 2 [file Bis(2,6-lutidinium)_closo-dodecaborate(12)__IAM.pdf › I_Bis26Luti_closo-dodecaborane(12)/Bis(2,6-lutidinium)_closo-dodecaborate(12)__IAM.pdf]
