# Supplementary material for: Periodic Hirshfeld Atom Refinement
Source: J Phys Chem Lett. 2026 Feb 27;17(11):3170–9. doi: 10.1021/acs.jpclett.5c03918 (PMC13007019; doi:10.1021/acs.jpclett.5c03918)

checkCIF/PLATON report

Structure factors have been supplied for datablock(s) HMA-Mg_pHAR

THIS REPORT IS FOR GUIDANCE ONLY. IF USED AS PART OF A REVIEW PROCEDURE FOR PUBLICATION, IT SHOULD NOT REPLACE THE EXPERTISE OF AN EXPERIENCED CRYSTALLOGRAPHIC REFEREE.

No syntax errors found. CIF dictionary Interpreting this report

Datablock: HMA-Mg_pHAR

```
Bond precision:   C-C = 0.0003 A           Wavelength=0.35320

Cell:              a=10.195(2)     b=11.759(2)         c=6.6206(13)
                   alpha=90        beta=103.67(3)      gamma=90
Temperature:       15 K

                 Calculated                       Reported
Volume           771.2(3)                         771.2(3)
Space group      P 21/c                           P 1 21/c 1
Hall group       -P 2ybc                          -P 2ybc
Moiety formula   2(C4 H3 O4), H12 Mg O6           2(C4 H3 O4), H12 Mg O6
Sum formula      C8 H18 Mg O14                    C8 H18 Mg O14
Mr               362.53                           362.54
Dx,g cm-3        1.561                            1.561
Z                2                                2
Mu (mm-1)        0.049                            0.049
F000             380.0                            380.0
F000'            379.96
h,k,lmax         25,29,16                         25,29,16
Nref             12628                            11647
Tmin,Tmax        0.993,0.995
Tmin'            0.993

Correction method= Not given

Data completeness= 0.922           Theta(max)= 26.199

                                                        wR2(reflections)=
R(reflections)= 0.0162( 11647)                          wR= 0.0172(
                                                        11647)
S = 3.141                  Npar= 187
```

The following ALERTS were generated. Each ALERT has the format
test-name_ALERT_alert-type_alert-level.
Click on the hyperlinks for more details of the test.

Alert level A

```
PLAT925_ALERT_1_A The Reported and Calculated Rho(max) Differ by .       3.89 eA-3
```

Author Response: The pHAR involves non-spherical scattering factors, so the atom- in-molecule (AIM) model is not applicable.

```
PLAT971_ALERT_2_A Check Calcd Resid. Dens.  0.24Ang From O7              4.54 eA-3
```

Author Response: The pHAR involves non-spherical scattering factors, so the atom- in-molecule (AIM) model is not applicable.

```
PLAT971_ALERT_2_A Check Calcd Resid. Dens.  0.31Ang From O7              4.53 eA-3
```

Author Response: The pHAR involves non-spherical scattering factors, so the atom- in-molecule (AIM) model is not applicable.

```
PLAT971_ALERT_2_A Check Calcd Resid. Dens.  0.31Ang From O7              4.53 eA-3
```

Author Response: The pHAR involves non-spherical scattering factors, so the atom- in-molecule (AIM) model is not applicable.

Alert level C

```
CELLK01_ALERT_1_C  Check that the cell measurement temperature is in Kelvin.
            Value of measurement temperature given =    14.900
GOODF01_ALERT_2_C  The least squares goodness of fit parameter lies
            outside the range 0.80 <> 2.00
            Goodness of fit given =      3.141
REFLE01_ALERT_3_C  The _reflns_threshold_multiplier given is >= 4
            Premultiplier =    4.01
REFLE01_ALERT_3_C  The _reflns_threshold_multiplier given is >= 4
            Premultiplier =    4.01
PLAT202_ALERT_3_C Isotropic non-H Atoms in Anion/Solvent ........          1 Check
              O7
PLAT250_ALERT_2_C Large U3/U1 Ratio for <U(i,j)> Tensor(Resd    1)        2.2 Note
PLAT711_ALERT_1_C BOND    Unknown or Inconsistent Label .........         O6 Check
                    MG          O6
PLAT711_ALERT_1_C BOND    Unknown or Inconsistent Label .........         O9 Check
                    MG          O9
PLAT711_ALERT_1_C BOND    Unknown or Inconsistent Label .........        O10 Check
                    MG          O10
PLAT711_ALERT_1_C BOND    Unknown or Inconsistent Label .........         O6 Check
                    O6          H8
```

```
PLAT711_ALERT_1_C BOND    Unknown or Inconsistent Label .........          O6 Check
                   O6          H9
PLAT711_ALERT_1_C BOND    Unknown or Inconsistent Label .........          O9 Check
                   O9          H12
PLAT711_ALERT_1_C BOND    Unknown or Inconsistent Label .........          O9 Check
                   O9          H13
PLAT711_ALERT_1_C BOND    Unknown or Inconsistent Label .........         O10 Check
                   O10         H14
PLAT711_ALERT_1_C BOND    Unknown or Inconsistent Label .........         O10 Check
                   O10         H15
PLAT712_ALERT_1_C ANGLE   Unknown or Inconsistent Label .........          O6 Check
                   MG          O6          H8
PLAT712_ALERT_1_C ANGLE   Unknown or Inconsistent Label .........          O6 Check
                   MG          O6          H9
PLAT712_ALERT_1_C ANGLE   Unknown or Inconsistent Label .........          O9 Check
                   MG          O9          H12
PLAT712_ALERT_1_C ANGLE   Unknown or Inconsistent Label .........          O9 Check
                   MG          O9          H13
PLAT712_ALERT_1_C ANGLE   Unknown or Inconsistent Label .........         O10 Check
                   MG          O10         H14
PLAT712_ALERT_1_C ANGLE   Unknown or Inconsistent Label .........         O10 Check
                   MG          O10         H15
PLAT712_ALERT_1_C ANGLE   Unknown or Inconsistent Label .........          O6 Check
                   O5          MG          O6
PLAT712_ALERT_1_C ANGLE   Unknown or Inconsistent Label .........          O9 Check
                   O5          MG          O9
PLAT712_ALERT_1_C ANGLE   Unknown or Inconsistent Label .........         O10 Check
                   O5          MG          O10
PLAT712_ALERT_1_C ANGLE   Unknown or Inconsistent Label .........          O6 Check
                   O6          MG          O7
PLAT712_ALERT_1_C ANGLE   Unknown or Inconsistent Label .........          O6 Check
                   O6          MG          O8
PLAT712_ALERT_1_C ANGLE   Unknown or Inconsistent Label .........          O6 Check
                   O6          MG          O9
PLAT712_ALERT_1_C ANGLE   Unknown or Inconsistent Label .........          O6 Check
                   O6          MG          O10
PLAT712_ALERT_1_C ANGLE   Unknown or Inconsistent Label .........          O9 Check
                   O7          MG          O9
PLAT712_ALERT_1_C ANGLE   Unknown or Inconsistent Label .........         O10 Check
                   O7          MG          O10
PLAT712_ALERT_1_C ANGLE   Unknown or Inconsistent Label .........          O9 Check
                   O8          MG          O9
PLAT712_ALERT_1_C ANGLE   Unknown or Inconsistent Label .........         O10 Check
                   O8          MG          O10
PLAT712_ALERT_1_C ANGLE   Unknown or Inconsistent Label .........          O9 Check
                   O9          MG          O10
PLAT712_ALERT_1_C ANGLE   Unknown or Inconsistent Label .........          H8 Check
                   H8          O6          H9
PLAT712_ALERT_1_C ANGLE   Unknown or Inconsistent Label .........         H12 Check
                   H12         O9          H13
PLAT712_ALERT_1_C ANGLE   Unknown or Inconsistent Label .........         H14 Check
                   H14         O10         H15
PLAT713_ALERT_1_C TORSION Unknown or Inconsistent Label .........          O6 Check
                   O5          MG          O6          H8
PLAT713_ALERT_1_C TORSION Unknown or Inconsistent Label .........          O6 Check
                   O5          MG          O6          H9
PLAT713_ALERT_1_C TORSION Unknown or Inconsistent Label .........          O9 Check
```

```
                    O5          MG          O9          H12
PLAT713_ALERT_1_C TORSION Unknown or Inconsistent Label ..........         O9 Check
                    O5          MG          O9          H13
PLAT713_ALERT_1_C TORSION Unknown or Inconsistent Label ..........        O10 Check
                    O5          MG          O10         H14
PLAT713_ALERT_1_C TORSION Unknown or Inconsistent Label ..........        O10 Check
                    O5          MG          O10         H15
PLAT713_ALERT_1_C TORSION Unknown or Inconsistent Label ..........         O6 Check
                    O6          MG          O5          H6
PLAT713_ALERT_1_C TORSION Unknown or Inconsistent Label ..........         O6 Check
                    O6          MG          O5          H7
PLAT713_ALERT_1_C TORSION Unknown or Inconsistent Label ..........         O6 Check
                    O6          MG          O7          H4
PLAT713_ALERT_1_C TORSION Unknown or Inconsistent Label ..........         O6 Check
                    O6          MG          O7          H10
PLAT713_ALERT_1_C TORSION Unknown or Inconsistent Label ..........         O6 Check
                    O6          MG          O8          H5
PLAT713_ALERT_1_C TORSION Unknown or Inconsistent Label ..........         O6 Check
                    O6          MG          O8          H11
PLAT713_ALERT_1_C TORSION Unknown or Inconsistent Label ..........         O6 Check
                    O6          MG          O9          H12
PLAT713_ALERT_1_C TORSION Unknown or Inconsistent Label ..........         O6 Check
                    O6          MG          O9          H13
PLAT713_ALERT_1_C TORSION Unknown or Inconsistent Label ..........         O6 Check
                    O6          MG          O10         H14
PLAT713_ALERT_1_C TORSION Unknown or Inconsistent Label ..........         O6 Check
                    O6          MG          O10         H15
PLAT713_ALERT_1_C TORSION Unknown or Inconsistent Label ..........         O6 Check
                    O7          MG          O6          H8
PLAT713_ALERT_1_C TORSION Unknown or Inconsistent Label ..........         O6 Check
                    O7          MG          O6          H9
PLAT713_ALERT_1_C TORSION Unknown or Inconsistent Label ..........         O9 Check
                    O7          MG          O9          H12
PLAT713_ALERT_1_C TORSION Unknown or Inconsistent Label ..........         O9 Check
                    O7          MG          O9          H13
PLAT713_ALERT_1_C TORSION Unknown or Inconsistent Label ..........        O10 Check
                    O7          MG          O10         H14
PLAT713_ALERT_1_C TORSION Unknown or Inconsistent Label ..........        O10 Check
                    O7          MG          O10         H15
PLAT713_ALERT_1_C TORSION Unknown or Inconsistent Label ..........         O6 Check
                    O8          MG          O6          H8
PLAT713_ALERT_1_C TORSION Unknown or Inconsistent Label ..........         O6 Check
                    O8          MG          O6          H9
PLAT713_ALERT_1_C TORSION Unknown or Inconsistent Label ..........         O9 Check
                    O8          MG          O9          H12
PLAT713_ALERT_1_C TORSION Unknown or Inconsistent Label ..........         O9 Check
                    O8          MG          O9          H13
PLAT713_ALERT_1_C TORSION Unknown or Inconsistent Label ..........        O10 Check
                    O8          MG          O10         H14
PLAT713_ALERT_1_C TORSION Unknown or Inconsistent Label ..........        O10 Check
                    O8          MG          O10         H15
PLAT713_ALERT_1_C TORSION Unknown or Inconsistent Label ..........         O9 Check
                    O9          MG          O5          H6
PLAT713_ALERT_1_C TORSION Unknown or Inconsistent Label ..........         O9 Check
                    O9          MG          O5          H7
PLAT713_ALERT_1_C TORSION Unknown or Inconsistent Label ..........         O9 Check
                    O9          MG          O6          H8
```

```
PLAT713_ALERT_1_C TORSION Unknown or Inconsistent Label .........         O9 Check
                   O9          MG          O6          H9
PLAT713_ALERT_1_C TORSION Unknown or Inconsistent Label .........         O9 Check
                   O9          MG          O7          H4
PLAT713_ALERT_1_C TORSION Unknown or Inconsistent Label .........         O9 Check
                   O9          MG          O7          H10
PLAT713_ALERT_1_C TORSION Unknown or Inconsistent Label .........         O9 Check
                   O9          MG          O8          H5
PLAT713_ALERT_1_C TORSION Unknown or Inconsistent Label .........         O9 Check
                   O9          MG          O8          H11
PLAT713_ALERT_1_C TORSION Unknown or Inconsistent Label .........         O9 Check
                   O9          MG          O10         H14
PLAT713_ALERT_1_C TORSION Unknown or Inconsistent Label .........         O9 Check
                   O9          MG          O10         H15
PLAT713_ALERT_1_C TORSION Unknown or Inconsistent Label .........        O10 Check
                   O10         MG          O5          H6
PLAT713_ALERT_1_C TORSION Unknown or Inconsistent Label .........        O10 Check
                   O10         MG          O5          H7
PLAT713_ALERT_1_C TORSION Unknown or Inconsistent Label .........        O10 Check
                   O10         MG          O6          H8
PLAT713_ALERT_1_C TORSION Unknown or Inconsistent Label .........        O10 Check
                   O10         MG          O6          H9
PLAT713_ALERT_1_C TORSION Unknown or Inconsistent Label .........        O10 Check
                   O10         MG          O7          H4
PLAT713_ALERT_1_C TORSION Unknown or Inconsistent Label .........        O10 Check
                   O10         MG          O7          H10
PLAT713_ALERT_1_C TORSION Unknown or Inconsistent Label .........        O10 Check
                   O10         MG          O8          H5
PLAT713_ALERT_1_C TORSION Unknown or Inconsistent Label .........        O10 Check
                   O10         MG          O8          H11
PLAT713_ALERT_1_C TORSION Unknown or Inconsistent Label .........        O10 Check
                   O10         MG          O9          H12
PLAT713_ALERT_1_C TORSION Unknown or Inconsistent Label .........        O10 Check
                   O10         MG          O9          H13
PLAT790_ALERT_4_C Centre of Gravity not Within Unit Cell: Resd.  #          1 Note
              C4 H3 O4
PLAT924_ALERT_1_C The Reported and Calculated Rho(min) Differ by .       1.54 eA-3
PLAT972_ALERT_2_C Check Calcd Resid. Dens.  0.25Ang From O7             -2.10 eA-3
PLAT972_ALERT_2_C Check Calcd Resid. Dens.  0.31Ang From O7             -2.10 eA-3
PLAT972_ALERT_2_C Check Calcd Resid. Dens.  0.31Ang From O7             -2.00 eA-3
PLAT972_ALERT_2_C Check Calcd Resid. Dens.  0.31Ang From O7             -2.00 eA-3
PLAT975_ALERT_2_C Check Calcd Resid. Dens.  0.43Ang From O8      .       0.84 eA-3
PLAT975_ALERT_2_C Check Calcd Resid. Dens.  0.41Ang From O5      .       0.74 eA-3
PLAT975_ALERT_2_C Check Calcd Resid. Dens.  0.56Ang From O7      .       0.63 eA-3
PLAT977_ALERT_2_C Check Negative Difference Density on H1        .      -0.32 eA-3
```

Alert level G

```
ABSMU01_ALERT_1_G  Calculation of _exptl_absorpt_correction_mu
                not performed for this radiation type.
PLAT005_ALERT_5_G No Embedded Refinement Details Found  in the CIF     Please Do !
PLAT092_ALERT_4_G Check: Wavelength Given is NOT Cu,Ga,Mo,Ag,In Ka    0.35320 Ang.
PLAT303_ALERT_2_G Full Occupancy Atom H1        with # Connections       2.00 Check
PLAT710_ALERT_4_G Delete 1-2-3 or 2-3-4 Linear Torsion Angle ... #         71 Do !
              O1  -H1  -O2  -C4    -35.00  4.00   1_555   1_555   1_555   1_555
PLAT710_ALERT_4_G Delete 1-2-3 or 2-3-4 Linear Torsion Angle ... #         72 Do !
              O2  -H1  -O1  -C1     34.00  4.00   1_555   1_555   1_555   1_555
```

```
PLAT808_ALERT_5_G No Parseable SHELXL Style Weighting Scheme Found     Please Check
PLAT883_ALERT_1_G Absent Datum for _atom_sites_solution_primary ..     Please Do !
PLAT911_ALERT_3_G Missing FCF Refl Between Thmin & STh/L=    0.600         10 Report
               10  0  0,  -1 11  2,  -1  4  3,   7  7  3,   7  1  4,   1  6  4,
               -3 11  4,  -8  8  5,   1 10  5,   3  1  6,
PLAT912_ALERT_4_G Missing # of FCF Reflections Above STh/L=  0.600        971 Note
PLAT929_ALERT_5_G No Weight Pars,Obs and Calc R1,wR2,S not Checked          ! Info
PLAT961_ALERT_5_G Dataset Contains no Negative Intensities .......     Please Check
PLAT969_ALERT_5_G The 'Henn et al.' R-Factor-gap value ..........       3.142 Note
              Predicted wR2: Based on SigI**2  1.10 or SHELX Weight  1.10
PLAT978_ALERT_2_G Number C-C Bonds with Positive Residual Density.          3 Info
PLAT980_ALERT_1_G No Anomalous Scattering Factors Found in CIF ...     Please Check
PLAT992_ALERT_5_G Repd & Actual _reflns_number_gt Values Differ by          5 Check
```

---

4 **ALERT level A** = Most likely a serious problem - resolve or explain
0 **ALERT level B** = A potentially serious problem, consider carefully
94 **ALERT level C** = Check. Ensure it is not caused by an omission or oversight
16 **ALERT level G** = General information/check it is not something unexpected

84 ALERT type 1 CIF construction/syntax error, inconsistent or missing data
15 ALERT type 2 Indicator that the structure model may be wrong or deficient
4 ALERT type 3 Indicator that the structure quality may be low
5 ALERT type 4 Improvement, methodology, query or suggestion
6 ALERT type 5 Informative message, check

It is advisable to attempt to resolve as many as possible of the alerts in all categories. Often the minor alerts point to easily fixed oversights, errors and omissions in your CIF or refinement strategy, so attention to these fine details can be worthwhile. In order to resolve some of the more serious problems it may be necessary to carry out additional measurements or structure refinements. However, the purpose of your study may justify the reported deviations and the more serious of these should normally be commented upon in the discussion or experimental section of a paper or in the "special_details" fields of the CIF. checkCIF was carefully designed to identify outliers and unusual parameters, but every test has its limitations and alerts that are not important in a particular case may appear. Conversely, the absence of alerts does not guarantee there are no aspects of the results needing attention. It is up to the individual to critically assess their own results and, if necessary, seek expert advice.

Publication of your CIF in IUCr journals

A basic structural check has been run on your CIF. These basic checks will be run on all CIFs submitted for publication in IUCr journals (*Acta Crystallographica*, *Journal of Applied Crystallography*, *Journal of Synchrotron Radiation*); however, if you intend to submit to *Acta Crystallographica Section C* or *E* or *IUCrData*, you should make sure that full publication checks are run on the final version of your CIF prior to submission.

Publication of your CIF in other journals

Please refer to the *Notes for Authors* of the relevant journal for any special instructions relating to CIF submission.

PLATON version of 04/06/2025; check.def file version of 30/05/2025

Datablock HMA-Mg_pHAR - ellipsoid plot

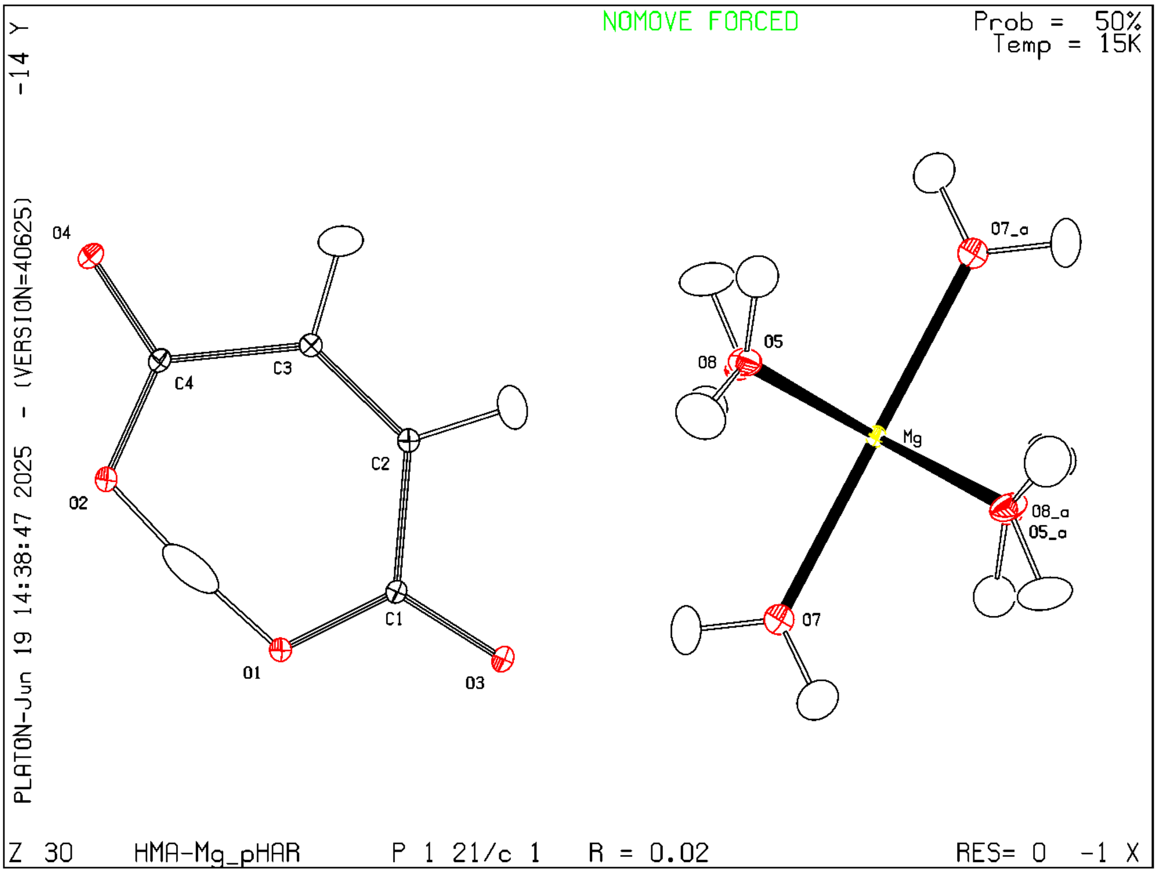

Supplement: Supplementary file 2 [file HMA-Mg_pHAR.pdf › Supple_HMA-Mg/HMA-Mg_pHAR.pdf]
