# Supplementary material for: Periodic Hirshfeld Atom Refinement
Source: J Phys Chem Lett. 2026 Feb 27;17(11):3170–9. doi: 10.1021/acs.jpclett.5c03918 (PMC13007019; doi:10.1021/acs.jpclett.5c03918)

checkCIF/PLATON report

Structure factors have been supplied for datablock(s) Xylitol_HAR

THIS REPORT IS FOR GUIDANCE ONLY. IF USED AS PART OF A REVIEW PROCEDURE FOR PUBLICATION, IT SHOULD NOT REPLACE THE EXPERTISE OF AN EXPERIENCED CRYSTALLOGRAPHIC REFEREE.

No syntax errors found. CIF dictionary Interpreting this report

Datablock: Xylitol_HAR

Bond precision: C-C = 0.0008 A Wavelength=0.71073

Cell:	a=8.264(4)	b=8.901(2)	c=8.9223(14)
	alpha=90	beta=90	gamma=90
Temperature:	122 K		

	Calculated	Reported
Volume	656.3(4)	656.3(4)
Space group	P 21 21 21	P 21 21 21
Hall group	P 2ac 2ab	P 2ac 2ab
Moiety formula	C5 H12 O5	C5 H12 O5
Sum formula	C5 H12 O5	C5 H12 O5
Mr	152.15	152.15
Dx,g cm-3	1.540	1.540
Z	4	4
Mu (mm-1)	0.139	0.139
F000	328.0	328.0
F000'	328.25	
h,k,lmax	20,21,21	20,21,21
Nref	9942[5483]	4715
Tmin,Tmax	0.950,0.965	
Tmin'	0.950	

Correction method= Not given

Data completeness= 0.86/0.47 Theta(max)= 59.940

R(reflections)= 0.0138(4715) wR2(reflections)= wR= 0.0128(4715)

S = 0.654 Npar= 199

The following ALERTS were generated. Each ALERT has the format
test-name_ALERT_alert-type_alert-level.
Click on the hyperlinks for more details of the test.

Alert level A

PLAT184_ALERT_1_A Missing _cell_measurement_theta_min Value .... Please Do !

Author Response: This is a re-refinement of deposited data CCDC 1432562 (XYLTOL03). These values were not reported by the original authors.

PLAT185_ALERT_1_A Missing _cell_measurement_theta_max Value .... Please Do !

Author Response: This is a re-refinement of deposited data CCDC 1432562 (XYLTOL03). These values were not reported by the original authors.

PLAT881_ALERT_1_A No Datum for _diffrn_reflns_av_R_equivalents ... Please Do !

Author Response: This is a re-refinement of deposited data CCDC 1432562 (XYLTOL03). These values were not reported by the original authors.

Alert level C

GOODF01_ALERT_2_C The least squares goodness of fit parameter lies
outside the range 0.80 <> 2.00
Goodness of fit given = 0.654
REFLE01_ALERT_3_C The _reflns_threshold_multiplier given is >= 4
Premultiplier = 4.01
REFLE01_ALERT_3_C The _reflns_threshold_multiplier given is >= 4
Premultiplier = 4.01

Alert level G

PLAT005_ALERT_5_G No Embedded Refinement Details Found in the CIF Please Do !
PLAT791_ALERT_4_G Model has Chirality at C2 (Sohncke SpGr) S Verify
PLAT791_ALERT_4_G Model has Chirality at C4 (Sohncke SpGr) R Verify
PLAT808_ALERT_5_G No Parseable SHELXL Style Weighting Scheme Found Please Check
PLAT883_ALERT_1_G Absent Datum for _atom_sites_solution_primary .. Please Do !
PLAT911_ALERT_3_G Missing FCF Refl Between Thmin & STh/L= 0.600 5 Report
8 5 0, 7 6 1, 0 7 3, 0 8 7, 2 0 10,
PLAT912_ALERT_4_G Missing # of FCF Reflections Above STh/L= 0.600 764 Note
PLAT929_ALERT_5_G No Weight Pars,Obs and Calc R1,wR2,S not Checked ! Info

```
PLAT961_ALERT_5_G Dataset Contains no Negative Intensities .......     Please Check
PLAT969_ALERT_5_G The 'Henn et al.' R-Factor-gap value ...........      0.656 Note
              Predicted wR2: Based on SigI**2  3.93 or SHELX Weight  3.93
PLAT978_ALERT_2_G Number C-C Bonds with Positive Residual Density.          4 Info
PLAT980_ALERT_1_G No Anomalous Scattering Factors Found in CIF ...     Please Check
```

```
   3 ALERT level A = Most likely a serious problem - resolve or explain
   0 ALERT level B = A potentially serious problem, consider carefully
   3 ALERT level C = Check. Ensure it is not caused by an omission or oversight
  12 ALERT level G = General information/check it is not something unexpected

   5 ALERT type 1 CIF construction/syntax error, inconsistent or missing data
   2 ALERT type 2 Indicator that the structure model may be wrong or deficient
   3 ALERT type 3 Indicator that the structure quality may be low
   3 ALERT type 4 Improvement, methodology, query or suggestion
   5 ALERT type 5 Informative message, check
```

It is advisable to attempt to resolve as many as possible of the alerts in all categories. Often the minor alerts point to easily fixed oversights, errors and omissions in your CIF or refinement strategy, so attention to these fine details can be worthwhile. It is up to the individual to critically assess their own results and, if necessary, seek expert advice.

PLATON version of 26/09/2025; check.def file version of 20/09/2025

Datablock Xylitol_HAR - ellipsoid plot

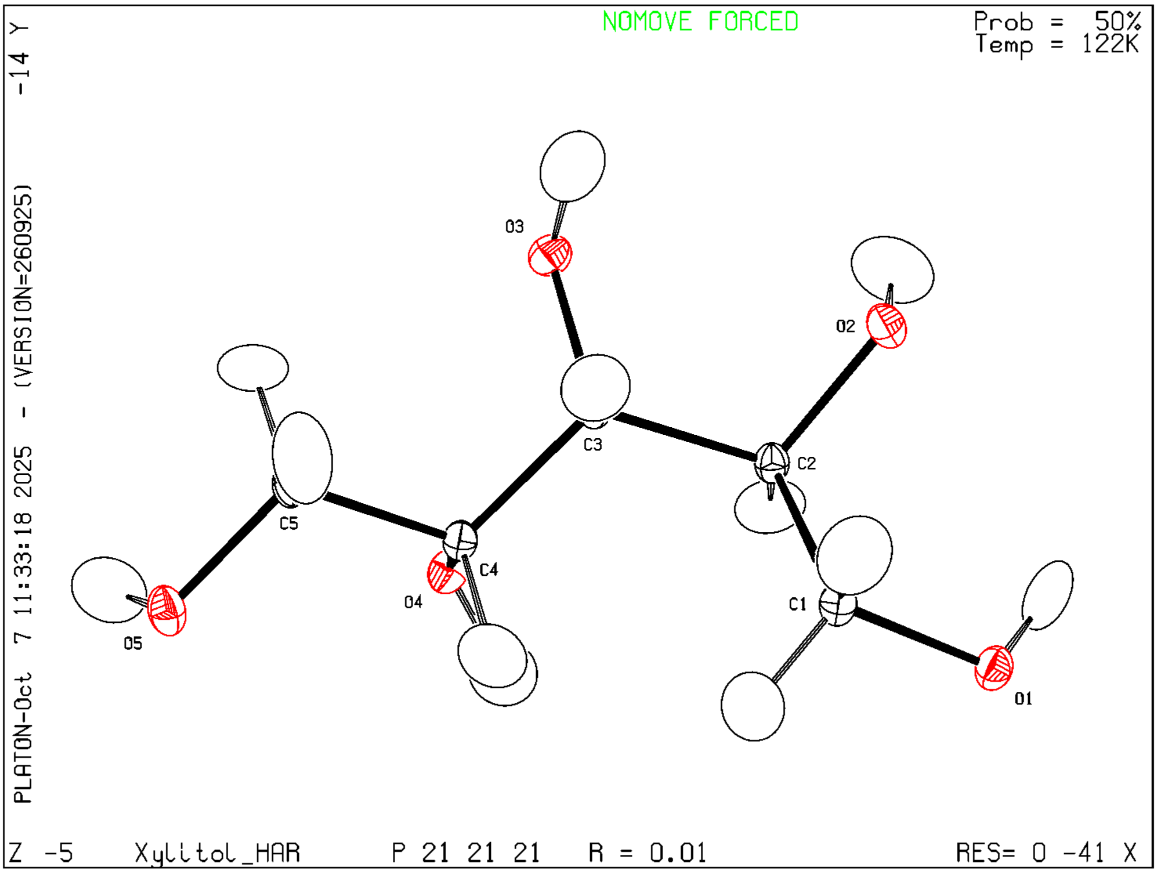

Supplement: Supplementary file 2 [file Xylitol_HAR.pdf › Supple_Xylitol/Xylitol_HAR.pdf]
